# Reproductive Hormone and Transcriptomic Responses of Pituitary Tissue in Anestrus Gilts Induced by Nutrient Restriction

**DOI:** 10.1371/journal.pone.0143219

**Published:** 2015-11-18

**Authors:** Shengyu Xu, Dingyue Wang, Dongsheng Zhou, Yan Lin, Lianqiang Che, Zhengfeng Fang, De Wu

**Affiliations:** Key Laboratory for Animal Disease Resistance Nutrition of the Ministry of Education, and Animal Nutrition Institute of Sichuan Agricultural University, Ya’an, P. R. China, 625014; Qingdao Agricultural University, CHINA

## Abstract

The onset of estrus is a critical sign of female sexual maturity. The pituitary plays a vital role in this process by the secretion of reproductive hormones. To investigate the effects of nutrient restriction on reproductive function and the underlying mechanisms involved, deep RNA sequencing of pituitary gland tissue was carried out to determine the differentially expressed genes (DEGs) between gilts in normal estrus, and gilts in which anestrus was induced by nutrient restriction. Gilts which had gone through two estrus cycles were fed a normal (CON, 2.86kg/d, n = 10) or nutrient restricted (NR, 1kg/d, n = 10) diet. The NR gilts experienced another three estrus cycles, but did not express estrus symptoms at the anticipated 6^th^ and 7^th^ cycles. Body weight gain in NR gilts was significantly decreased by nutrient restriction. Gilts were considered as anestrus when blood progesterone concentrations lower than 1.0 ng/mL from three consecutive blood samples were recorded. Circulating concentrations of progesterone (< 1.0 ng/mL vs. 2.1 ng/mL) and estradiol (208.6 ng/mL vs. 371.8 ng/mL) were significantly lower in the NR gilts than in the CON gilts. Between 5,360,000 and 5,370,000 sequence reads per sample from the CON and NR gilts’ pituitaries were obtained and mapped to the porcine genome. Analysis of read counts revealed 185 DEGs. Expression of selected genes was validated by the use of quantitative real-time RT-PCR. Bioinformatic analysis identified that the genes identified were enriched in the GO terms “neuroactive ligand-receptor interaction”, “GnRH signaling pathway” and “immune response system”. Our findings provide a new perspective for understanding the nutrient restriction-induced reproductive impairment at the pituitary transcriptional level, and how this is linked to hormone secretion. Moreover, the transcriptomic changes in anestrus gilts associated with nutrient restriction could be a resource for targeted studies of genes and pathways potentially involved in the regulation of reproductive function and animal health.

## Introduction

Throughout vertebrates the onset of estrus indicates that the female organism has reached sufficient sexual maturity to reproduce. Studies have shown that reproductive function in pigs is sensitive to the status of energy reserves suggesting that weight loss in humans by extreme dieting methods may have potential negative effects on fertility [1–3]. Nutritional factors are the strongest and most obvious environmental factors affecting the timing of puberty [4], and studies have revealed that nutrient restriction in post-pubertal gilts led to cessation of estrus [3]. However, in the pig industry, pre-pubertal gilts and weaned sows are always kept in good body condition without too much adipose tissue to allow the onset of puberty and estrus at the appropriate time.

Nutritional status, especially overall energy level, is important for the onset of estrus [1–3, 5]. Depressed luteinizing hormone (LH) and follicle stimulating hormone (FSH) secretion led to the inhibition of gonadal function and reproductive failure in nutrient restricted animals [2]. It is well known that estrus onset is a complex biological process which is initiated by hormonal signals from the brain to the gonads, involving the secretion of gonadotropin-releasing hormone (GnRH) by the hypothalamus [6], LH and FSH by the pituitary, and estradiol (E2) by the ovary. The coordinated actions of these hormones allow appropriate sexual organ development, pubertal development, and gametogenesis [7–8]. However, the underlying molecular and physiological mechanisms by which dietary factors influence reproductive function remain largely unknown. Notably, the secretion of LH and FSH by the pituitary directly regulates gonad function. Therefore, understanding the neuroendocrine control of LH and FSH secretion may provide insights into both normal reproduction and disorders of the pubertal process.

At the present time, it has become routine to obtain large-scale genetic data at the genomic and transcriptomic levels in pigs due to the development of high-throughput deep sequencing technologies. These approaches include Illumina’s RNA sequencing (RNA-seq) system, which employs whole transcriptome shotgun sequencing wherein mRNA or cDNA is mechanically fragmented, resulting in overlapping short fragments that cover the entire transcriptome. RNA-seq is powerful for unraveling transcriptome complexity, and for the identification of genes, splice variants, non-coding RNAs, and even novel transcriptional units [9–10].

The pig anestrus model is an important tool for the investigation of the influence of nutrition on estrus onset. Identification of differences in gene expression between normal and anestrus gilts could shed light on the underlying mechanisms. Therefore, using nutritionally-induced anestrus gilts, we assessed the hormonal secretion and transcriptomic responses of the pituitary by deep RNA sequencing. The mechanisms behind these nutrition-induced responses were investigated on a molecular basis.

## Materials and Methods

### Animals and diets

All experimental procedures were approved by the Animal Care and Use Committee of Sichuan Agricultural University. Twenty Landrace × Yorkshire crossbred gilts having experienced two estrus cycles were used in this study. Gilts with similar bodyweight (122 ± 4.5 kg) and age (230 ± 6 days) were allocated to two nutrition allowance levels and either fed a normal diet (control, CON, 2.86 kg/d) or a nutrient restricted diet (NR, 1 kg/d) [2] (n = 10/group). The CON gilts were fed 2.86 kg/d to meet the recommendations of the National Research Council (NRC, 1998), and NR gilts were fed 83% of the maintenance requirement as suggested by NRC (1998) [11]. Diets were formulated to meet NRC (1998) recommendations for nutritional requirements in breeding gilts (Table 1), containing 3.40 DE Mcal/kg and 14.3% of crude protein. Gilts were housed individually in a breeding facility where they were fed twice daily and were allowed to consume water *ad libitum*. The environmental temperature was controlled at 20–24°C. The condition of the animals was monitored and recorded daily.

**Table 1:** Diet composition and nutrient levels (90% Dry matter basis, %).

Ingredients (g/kg)		Calculated nutritive composition	
Corn	684.40	Digestible energy (Mcal/kg)	3.40
Soybean meal	159.50	Crude protein (%)	14.30
Whey bran	60.00	Starch (%)	47.30
Fish meal	20.00	Either extract (%)	7.10
Soybean oil	40.00	Soluble fiber (%)	1.62
Calcium Carbonate	10.00	Crude fiber (%)	2.46
Calcium hydrophosphate	6.80	Neutral Detergent Fiber (%)	10.05
Salt	4.00	Insoluble fiber (%)	10.50
Vitamin premix^a^	0.50	Lysine (%)	0.97
Trace mineral premix^b^	5.00	Calcium (g/kg)	9.20
Choline chloride	1.60	Phosphorous (g/kg)	6.70
Total	1000.00	Available phosphorous (g/kg)	4.60

Daily feeding intake in normal and restricted gilts were 2.86 kg/d and 1 kg/d, respectively.

^a^ Supplied per kilogram diet: Vitamin A, 16, 000 IU; Vitamin D_3_ 4, 000 IU; Vitamin E, 10 IU; Vitamin K, 2 mg; Vitamin B_1_, 0.8 mg; Vitamin B_2_, 6.4 mg; Vitamin B_12_, 0.012 mg; Niacin, 10 mg; Pantothenic, 10 mg; Folic acid, 0.2 mg.

^b^ Supplied per kilogram diet: Cu 13.5 mg, Fe 162 mg, Mn 54 mg, Zn 162 mg, I 0.55 mg, Se 0.4 mg.

### Nutritionally-induced anestrus definition and sample collection

The day of the second occurrence of standing heat in gilts was designated as day 0 (d 0). At d 0, the gilts in CON and NR groups were started on their respective feeding regimens. All gilts had fence-line contact with a mature boar to encourage pubertal estrus. Onset of estrus was detected by conducted by back pressure testing and examination of vulval characteristics twice daily, in the morning and afternoon, by an experienced stockperson. Gilts failed to express estrus until the 23rd day of the 6th estrus cycle (d 88) in the NR group. Blood samples (10 mL) were collected from the both groups of gilts every 3 days to determine the concentration of progesterone. When progesterone concentrations were below 1.0 ng/mL for three consecutive days in gilts who did not enter estrus, this was defined as nutritionally induced anestrus [12].

At d 107 (when standing heat in the 7^th^ estrus cycle was observed in the control group) both groups of gilts were anaesthetized with an intravenous injection of Zoletil 50 (0.1 mg/kg body weight; Virbac, Carros cedex, France), and were bled by exsanguination. Brains were immediately removed from the skull and excess tissues were removed. Within a few minutes after slaughter the pituitary gland was removed and flushed with ice-cold PBS, then rapidly frozen in liquid nitrogen. All the samples were maintained at −80°C until RNA analysis were performed.

### Determination of hormone concentrations

Blood samples were collected on d 0, and every 3 days from d 88 to d 107 by jugular venipuncture. Blood samples were centrifuged at 2400 g for 15 min to collect serum and stored at -20°C for future analysis of hormone concentrations. Circulating concentrations of E2 and progesterone (P) were determined using enzyme-linked immunosorbent assay kits (R&D Systems Inc., Minneapolis, MN, USA), according to the manufacturer’s recommendations. The limits for detection of E2 and P were 0.01 ng/mL, and 1.0 ng/mL, respectively. The intra- and inter-assay coefficients of variation were 6.6% and 7.5% for E2, and 5.3% and 6.4% for P.

### Total RNA extraction from pituitary

Total RNA was extracted with TRIzol reagent (Invitrogen, Carlsbad, CA, USA) from frozen tissues ground in liquid nitrogen. The quality and purity of RNA samples were checked by spectrophotometry (Beckman DU-800, Los Angeles, CA, USA) from the OD_260_:OD_280_ and denaturing agarose gel electrophoresis. All RNA samples were treated with DNase-I (TaKaRa Biotechnology Co., Ltd, Dalian, China). RNA integrity and concentration were evaluated using an Agilent 2100 Bioanalyzer (Agilent Technologies, Folsom, CA, USA).

### Library construction and Illumina sequencing

For RNA library construction and deep sequencing, equal quantities of RNA from four individual pig pituitaries which were fed a normal diet were pooled, and RNA samples from the four nutrient restricted pig pituitaries were pooled. Approximately 4 μg of RNA from each treatment group were submitted for sequencing. In brief, after RNA extraction, poly A-containing mRNAs were purified using oligo-dT-conjugated magnetic beads and fragmented into small pieces. Using these short fragments as templates, first- and second-strand cDNA were synthesized. These cDNA fragments were subjected to end repair, addition of a single ‘A’ base, and ligation of adapters. Products were subsequently purified and amplified by PCR to construct the final cDNA libraries. Finally, the raw image files from each of the libraries were collected by the Illumina HiSeqTM 2000 sequencing platform in BGI Shenzhen (http://en.genomics.cn/navigation/index.action).

### Analysis of sequencing data and detection of differentially expressed genes

All data is MIAME compliant. For the raw data, we filtered adaptor tags, low quality tags and tags with a copy number of 1 to obtain clean tags. Subsequently, the clean tags were classified according their copy number in the library, and the percentage of total clean tags was calculated and saturation of the library was analyzed. The *Sus scrofa* UniGene database (http://www.ncbi.nlm.nih.gov/UniGene/UGOrg.cgi?TAXID=9823, UniGene Build #42 *Sus scrofa*, Jun, 19th, 2012) was employed for sequence analysis. To compare the differentially expressed genes (DEGs) in the two treatments, a rigorous algorithm has been developed and described previously [13]. The *P*-value was used corresponding to a differential gene expression test at statistically significant levels [14]. Genes were deemed significantly differentially expressed with a *P*-value < 0.005, FDR ≤ 0.001 and an absolute value of log2-fold change ≥1 in sequence counts across libraries.

### Quantitative PCR (qPCR) analysis

To confirm the differential expression of genes revealed by RNA-Seq, the expression of genes in the pituitary, including Kiss-1, G protein-coupled receptor 54 (GPR54), FSH, LH, GnRHR and leptin receptor (Ob-R), were measured by qPCR. Complementary DNA (cDNA) was synthesized with random primers (Invitrogen, Carlsbad, CA, USA). Real-time PCR was used to quantify mRNA expression levels using the Sybr Green Kit (Qiagen, Valencia, CA, USA). Primers are listed in Table 2. Amplification was carried out in a final reaction volume of 12.5 μL containing 5 μL of Sybr green master mix, 0.5 μL of each primer (final concentration 0.25 μmol/L), 6 μL of diethyl pyrocarbonate (DEPC)-treated water and 0.5 μL of cDNA. All reactions were carried out in triplicate on a DNA Engine thermal cycler (PTC-0200, Chromo4 Real-Time Detector; Bio-Rad, Hercules, CA, USA) using thermal cycling conditions (denaturation for 10 min at 95°C, amplification for 45 cycles with denaturation at 95°C for 20 sec, annealing at 60°C for 10 sec, extension at 72°C for 9 sec followed by fluorescence acquisition at 60°C for 5 sec). To detect potential contamination, one reaction was performed with the cDNA replaced by water. Product sizes were verified by agarose gel electrophoresis and all products were sequenced to confirm their identity. β-actin was used as a housekeeping gene for each sample to verify the presence of cDNA and as an internal control to calculate the relative level of target gene expression using the 2^-△△Ct^ method [15].

**Table 2:** Primers used in real-time PCR analysis for detection of Kiss-1, GPR54, FSH, LH, GnRHR, Ob-R and β-actin.

Genes	Primer sequences	Size of amplified fragment(bp)	GenBank accession number
Kiss-1	F: GGGGGACCTCATCGTGCCAG	179	AB466320
	R: CGCCTGCCGCTTTCCGTA		
GPR54	F: GCTCTACTCTACCCCCTA	115	DQ459345
	R: CGGAAACACAGTCACATA		
FSH	F: GTGGTGTGCTGGCTATTGCTAC	117	NM_213875
	R: CCAGGTACTTTCACGGTCTCGTA		
LH	F: AGAGACTGCTGTTGTGGCTGCT	142	NM_214080
	R: CTGGTGGTAAAGGTGATGCAGAC		
GnRHR	F: AGCCAACCTGTTGGAGACTCTGAT	101	NM_214273
	R: AGCTGAGGACTTTGCAGAGGAACT		
Ob-R	F: ATTTCTGCTATCTGGCTATA	101	NM_001024587
	R: GTTTCACCACGGAATC		
β-actin	F: GGCCGCACCACTGGCATTGTCAT	104	DQ845171.1
	R: AGGTCCAGACGCAGGATGGCG		

GPR54: G protein-coupled receptor 54, FSH: follicle stimulating hormone, LH: luteinizing hormone, GnRHR: gonadotropin-releasing hormone receptor, Ob-R: leptin receptor.

### Statistical analysis

Data from gilts in the normal and nutrient-restricted groups were compared by *t* test and presented as means ± SEM. Differences were considered significant at *P* < 0.05. Functional classification of DEGs was conducted using the Gene Ontology atlas. Pathway analysis of DEGs was mainly based on the Kyoto Encyclopedia of Genes and Genomes (KEGG) database [16].

## Results

### Clinical features of nutrient restriction

In the present study, nutrient restricted gilts (n = 10) which already had experienced two estrus cycles failed to express estrus at the anticipated time of the 6^th^ and 7^th^ estrus (d 88 and d 107). The day of the second occurrence of standing heat was designated as d 0. As shown in Table 3, the NR gilts had a lower body weight than the normal-fed gilts at d 89 and d 107 (144.70 ± 7.68 kg vs. 100.15 ± 4.38 kg; 154.58 ± 2.32 kg vs. 97.80 ± 6.21 kg; *P* = 0.001). The back fat thickness decreased in the NR group compared with the CON group at d 89 and d 107 (16.50 ± 0.65 mm vs. 6.25 ± 0.25 mm, *P* = 0.004; 19.05 ± 1.92 mm vs. 5.55 ± 1.38 mm, *P* = 0.002).

**Table 3:** Body weight and backfat thickness in normal-fed or nutrient-restricted gilts.

Item		CON	NR
BW (kg)	d 0	122.40 ± 6.67	124.05 ± 5.87
	d 88	144.70 ± 7.68^a^	100.15 ± 4.38^b^
	d 107	154.58 ± 2.32^a^	97.80 ± 6.21^b^
P2 thickness(mm)	d 0	13.25 ± 0.75	13.75 ± 0.75
	d 88	16.50 ± 0.65^a^	6.25 ± 0.25^b^
	d 107	19.05 ± 1.92^a^	5.55 ± 1.38^b^

^a, b^ in the same line denotes the effects of timing on the changes of bodyweight and P2 thickness.

CON denotes normal-fed gilts (2.86 kg/d) and NR denotes nutrient restricted gilts (1 kg/d). The day of the second occurrence of standing heat was designated as d 0. *n* = 10.

### Circulating hormone concentrations

**Fig 1:**
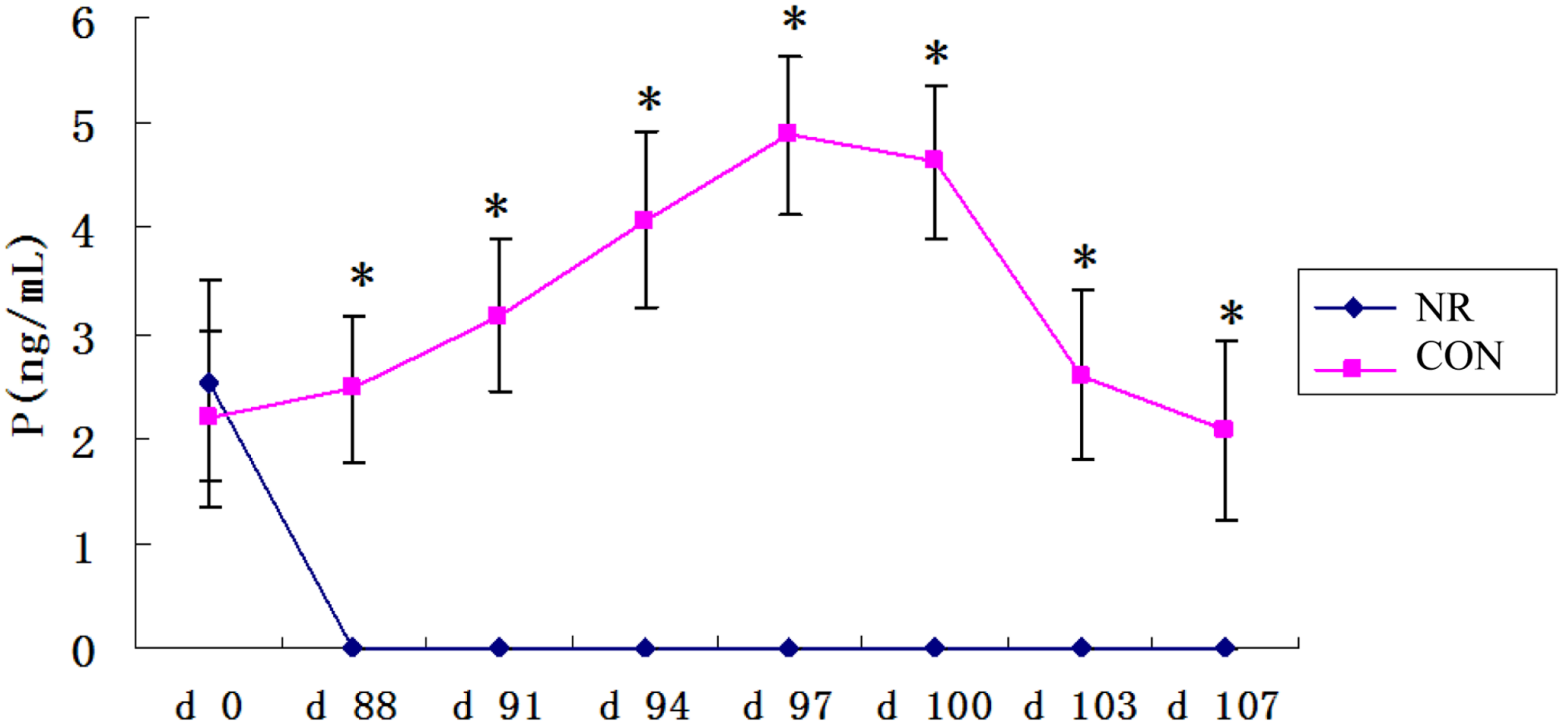
Concentrations of progesterone (P) in normal (CON) or nutrient-restricted (NR) gilts. CON denotes normally fed gilts (2.86 kg/d) and NR denotes nutrient restricted gilts (1 kg/d). The day of the second occurrence of standing heat was designated as d 0. P, progesterone; E2, estradiol. * denotes *P* < 0.05. n = 10.

The circulating concentrations of progesterone were determined at d 0 and every 3 days from d 88 to d 107. In the NR gilts progesterone concentrations were lower than 1.0 ng/mL and also beyond the limit of detection from d 88 to d 107. As shown in Fig 1, progesterone concentrations were significantly decreased in the NR gilts compared with the CON gilts group from d 88 to d 107 (< 1.0 ng/mL vs. > 2.0 ng/mL, *P* < 0.01). This was a reflection of nutritionally induced anestrus [17].

**Fig 2:**
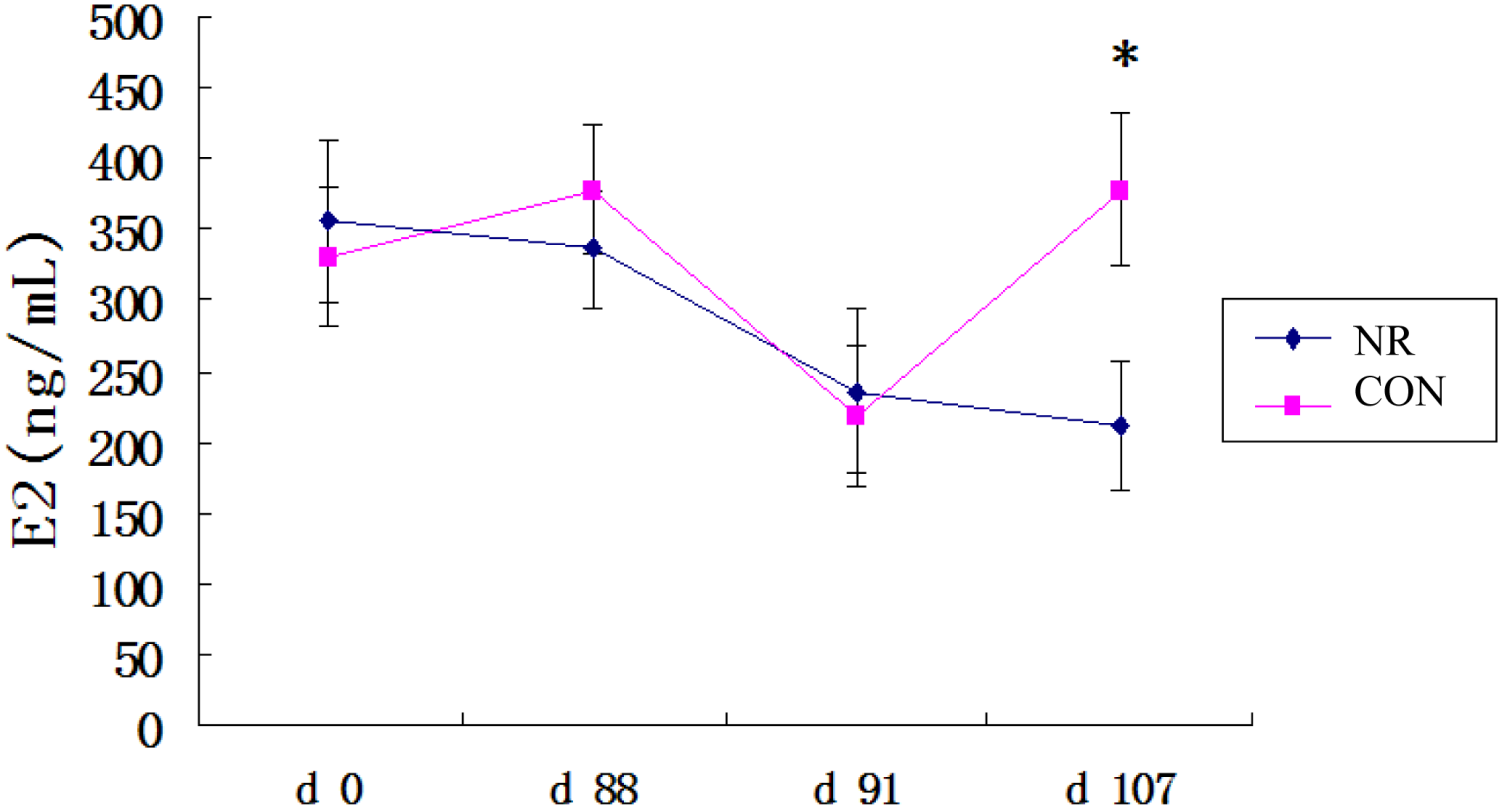
Concentrations of estradiol (E2) in normal (CON) or nutrient-restricted (NR) gilts. CON denotes normally fed gilts (2.86 kg/d) and NR denotes nutrient restricted gilts (1 kg/d). The day of the second occurrence of standing heat was designated as d 0. P, progesterone; E2, estradiol. * denotes *P* < 0.05. n = 10.

Circulating concentrations of E2 were determined at d 0, d 88, d 91 and d 107. E2 concentrations at d 0, d 88 and d 91 were not affected by nutrient restriction, with the exception of decreased E2 concentration in the NR gilts at d 107 compared with the CON group (208.6 ± 32.1 ng/mL vs. 371.8 ± 38.5 ng/mL, Fig 2).

### Summary of RNA-Seq data

To investigate the regulation of the response to nutrient restriction in gilts, we obtained the global gene expression profiles in pituitary tissues using RNA-seq. We sequenced two porcine pituitary RNA-seq libraries from CON and NR groups using parallel sequencing on the Illumina platform. Sequencing quality evaluation is shown in S1 Fig. A summary of sequencing tags and matched genes is shown in Table 4. RNA-seq yielded 7,187,315 and 7,221,064 raw reads from the CON and NR libraries, respectively. Low quality reads (i.e., tags containing only adaptors and ambiguously called bases) were removed, resulting in 7,160,026 and 7,193,730 clean tags, 7,147,897 and 7,180,408 of which were distinct (i.e., non-identical), from CON and NR gilts, respectively. These distinct clean tags were mapped to 5,358,288 and 5,369,164 genes in the *Sus scrofa* UniGene database for CON and NR libraries, respectively.

**Table 4:** RNA-Seq data summary and tag mapping to the UniGene transcript database.

RNA-Seq sample		CON	NR
Total tags (raw data)		7,187,315	7,221,064
Clean tags		7,160,026	7,193,730
Total distinct clean tags		7,147,897	7,180,408
Mapping to gene	TDCT	5,358,288	5,369,164
	DCT	4,224,291	4,138,639
Unambiguous Mapping	TDCT	3,494,005	3,320,515
	DCT	2,836,845	2,973,311
Unknown Tag		2,923,606	3,041,769

TDCT- Total distinct clean tag; DCT- distinct clean tag. CON denotes normal-fed gilts (2.86 kg/d) and NR denotes nutrient restricted gilts (1 kg/d).

### Identification of differentially expressed genes

To compare the global transcriptional changes between CON and NR pituitary tissues, we applied the same method as described previously [13] to identify DEGs from the normalized RNA-Seq data by pairwise comparisons between the two treatments. This showed that 36,244 genes were expressed differentially between the two treatments as shown in S1 Table. There were 185 genes with *P* values < 0.005, a false discovery rate (FDR) ≤ 0.001 and estimated absolute log2-fold change ≥ 1 in the pairwise comparisons (S2 Table). The NR pituitary samples showed 98 downregulated and 87 upregulated genes compared with CON (S2 Fig), according to statistical criteria for raw reads.

### Gene ontology and signaling pathway analysis of differentially expressed genes

The molecular function (MF), biological process (BP) and cellular component (CC) analysis based on gene ontology (GO) in the 185 identified DEGs are shown in S3 Table. GO analysis identified that the MF of “sequence-specific regulatory transcription factor site binding RNA polymerase II transcription factor activity” was significantly affected by nutrient restriction (*P* < 0.05, S3.1 Table). Interestingly, BP enrichment performed by two-sided Fisher’s exact test showed enrichment of processes involving “response to endogenous stimulus”, “response to hormone stimulus”, “humoral immune response mediated by circulating immunoglobulin” and “humoral immune response” as well as several others (*P* < 0.05, S3.2 Table). In addition, we found that the CON and NR gilts have differences in cellular components, including “extracellular region” and “extracellular region part” (*P* < 0.05, S3.3 Table).

The 185 DEGs could be grouped into 138 pathways based on analysis using the KEGG pathway database. These pathways and the DEGs involved are shown in S4 Table. The statistically significant signaling pathways included “neuroactive ligand-receptor interaction”, “complement and coagulation cascades”, “salivary secretion”, “cytokine-cytokine receptor interaction”, “GnRH signaling pathway”, “Jak-STAT signaling pathway”, “autoimmune thyroid disease”, “ECM-receptor interaction”, “taste transduction”, “calcium signaling pathway”, “MAPK signaling pathway”, “oocyte meiosis” and “progesterone-mediated oocyte maturation”.

### Validation of RNA-Seq data by qPCR

**Fig 3:**
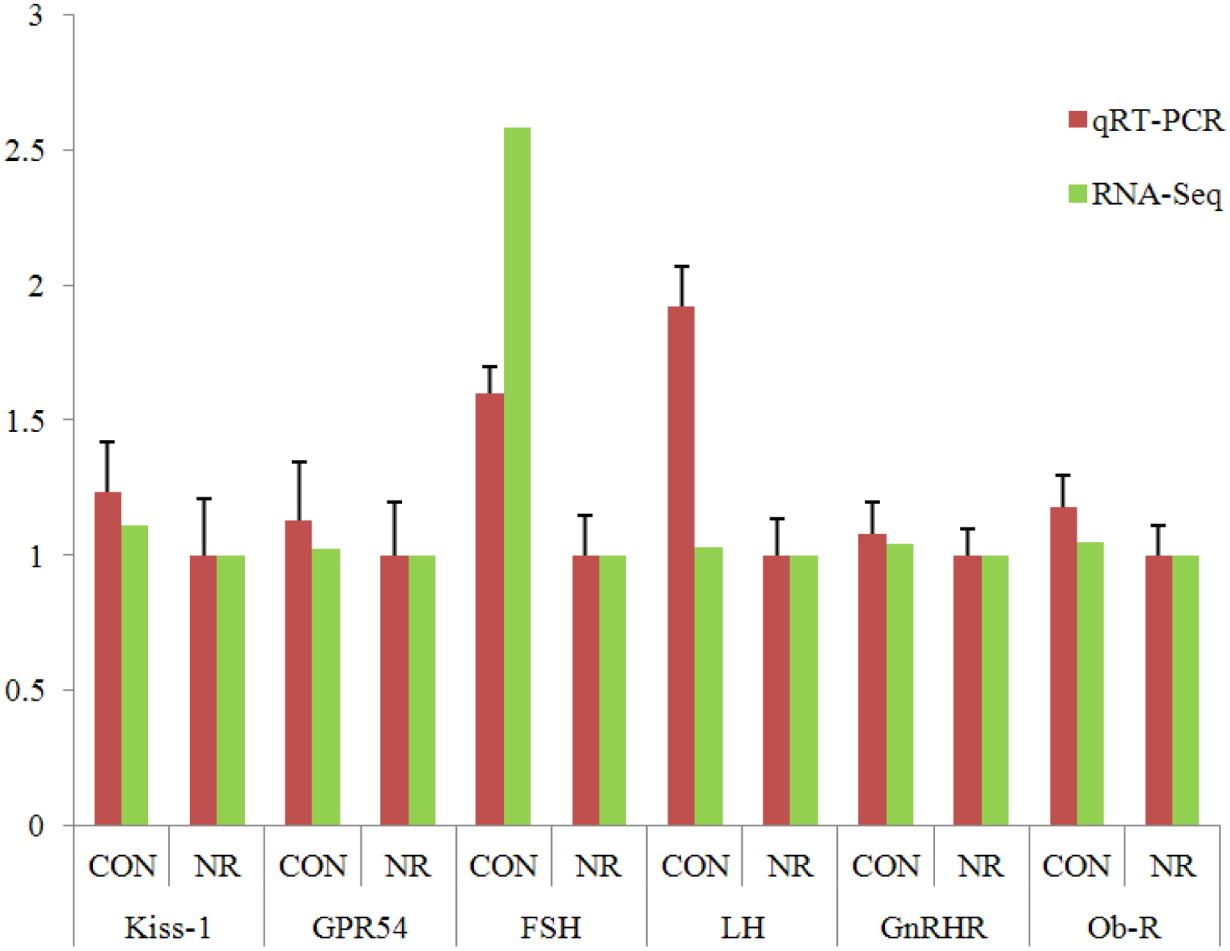
Validation of RNA-Seq data by qRT-PCR. Results are expressed as the target/reference ratio of each sample normalized by the target/reference ratio of the calibrator. β-actin was used as a reference gene. The vertical axis indicates the fold change of transcript abundance in CON gilts compared to the NR. For the NR sample, the fold change of transcript abundance relative to the NR sample equals one, by definition. qRT-PCR: the RNA samples from independent RNA extractions from six biological replicates; RNA-Seq: the results of deep RNA sequencing. Error bars represent standard error. CON denotes normally fed gilts (2.86 kg/d) and NR denotes nutrient restricted gilts (1 kg/d). GPR54: G protein-coupled receptor 54, FSH: follicle stimulating hormone, LH: luteinizing hormone, GnRHR: gonadotropin-releasing hormone receptor, Ob-R: leptin receptor.

To validate DEGs identified by RNA-seq, we used qRT-PCR to measure the expression of six genes. The genes were selected based on their involvement in neuroactive ligand-receptor interaction and GnRH signaling pathway (FSH, LH and GnRHR) or because they are important components of estrus onset (Kiss-1, GPR54 and Ob-R). Our results indicated that most of the genes investigated had expression levels which were consistent between RNA-Seq and qRT-PCR assays (Fig 3). There were higher transcript levels for FSH and LH in the CON group than in the NR group (*P* < 0.05). The expression of Kiss1, GPR54 and GnRHR were not affected by nutrient restriction.

## Discussion

The onset of estrus is the hallmark of reproductive maturity, and a sign that the female individual is sufficiently mature to produce offspring. It has been reported that nutritional factors are the major environmental influence on the timing of puberty [4]. However, until now, there has been no report of the genome-wide expression analysis of pituitaries in gilts where anestrus was induced by nutritional manipulation. Our study used RNA-seq to obtain new information on gene expression profiles in nutrient restricted and normally fed gilts.

In this study, the gilts were allowed to initially experience two estrus cycles. In the NR group, the nutrition allowance level was 1 kg/d, which is lower than the maintenance nutrient requirement by NRC (1998) [11]. Our results are in agreement with a previous study in gilts showing that nutrient restriction led to the loss of body fat reserves and eventual cessation of the estrus cycle [2]. Anestrus was observed at d 88 and d 107 in the NR group (anticipated time of 6^th^ and 7^th^ estrus). The concentrations of progesterone were below 1.0 ng/mL for the three consecutive blood samples after the point defined as nutritionally induced anestrus [12]. It is well known that E2 plays a key role in ovarian follicular development, oocyte maturation, ovulation and luteogenesis [18–20]. Also, it has been demonstrated that the production of E2 is the hallmark of pre-ovulatory follicular development in both animal models and in human studies [19, 21]. Our previous research showed that the nutrient restriction significantly suppressed ovary as determined by ovary weight, number of large ovarian follicles, follicular fluid weight, and corpus luteum number [3]. The lower concentration of E2 observed in the NR group suggests that pre-ovulatory follicular development was impaired by nutrient restriction.

To investigate the global gene expression profiles in the NR group pituitaries, we used the Illumina RNA-Seq system, a tag-based novel high-throughput transcriptome deep sequencing method. This was done using pooled biological replicates from four pigs from each group in order to obtain representative samples for deep sequencing analysis. We were able reach a sequencing depth of 7.19–7.22 million tags per library and found tags were mapped to around 5.4 million genes in the *Sus scrofa* UniGene database (Table 4). These results indicate the reliability of our RNA-seq methodology.

Based on GO analysis of the 185 DEGs identified in the NR gilts, multiple biological processes were significantly affected. As far as we know, the GO term “biological process response to hormone stimulus” is the most important in reproduction, which is the initiator of the estrus process [7]. However, the other effects of nutrient restriction are also likely to be important, and the DEG analysis showed massive changes in the transcript abundance of the terms “known developmental process”, “system development”, “cellular response to chemical stimulus”, and “humoral immune response genes”, which all may play a role in the ability of the animals to enter estrus. Our results may also provide further evidence for a relationship between nutrient restriction and the immune system.

Moreover, several components of the “neuroactive ligand-receptor interaction pathway”, including FSH, LH, thyroid stimulating hormone receptor (TSHR), growth hormone releasing hormone receptor (GHRHR), growth hormone (GH), prolactin (PRL) were altered by nutrient restriction. These genes not only play a vital role in reproduction and lactation [6, 7, 22, 23], but are also important in mammalian growth. For example, GH and TSHR are involved in the hypothalamus-pituitary-thyroid gland axis [24–26]. In this study, the transcription of FSH and LH were down regulated in pituitary tissue in the NR group (Fig 3). FSH stimulates the growth and recruitment of immature ovarian follicles in the ovary. In particular, FSH is the major survival factor that rescues the small antral follicles from apoptosis. LH is a larger protein hormone secreted into the general circulation from the anterior pituitary gland, and is essential for follicular development and oocyte maturation [27]. These observations may underlie the cessation of reproductive function in the nutritionally induced anestrus gilt as the results suggest the impaired secretion of the most important hormones in follicular development.

The GO terms “GnRH signaling pathway”, “oocyte meiosis” and “progesterone-mediated oocyte maturation” have well described roles in reproduction via DEGs including inositol 1,4,5-trisphosphate receptor type 1, calbindin 2 (CALM) and progesterone receptor [28, 29]. In the “complement and coagulation cascades pathway”, the coagulation factor XII (Hageman factor) was upregulated, and the complement component 3 (C3), C4, C7, complement component 1 and r subcomponent (C1R) were downregulated by nutrient restriction. These genes are important for organismal inflammation, prostaglandin biosynthesis, nitric oxide biosynthesis, and are also important for the immune functions [30]. The role of the Jak-STAT signaling pathway in growth, proliferation, development and immunity is well established in mammals [31–33]. Overall, of the significant signaling pathways, reproduction, immunity, and growth were the most vulnerable functions of the gilt under nutrient restriction conditions.

The Kiss1/GPR54 (G-protein-coupled receptor) system is considered as the gate keeper of estrus [34, 35]. This pathway plays a crucial role in the activity of GnRH neurons and in the timing of the onset of estrus in the hypothalamus [36, 37]. The pulsatile secretions of GnRH through activation of its receptor, GnRHR trigger the expression of FSH and LH in the anterior pituitary gland. However, we detected Kiss1 and GPR54 mRNA in the pituitary, but not in the hypothalamus. This could be the reason that Kiss1 and GPR54 expression in the pituitary is not regulated by nutrient restriction in this study. Although the expression of GnRHR was not affected by nutrient restriction, our previous research showed that the mRNA expression of Kiss-1, GPR54 and GnRH were decreased in the hypothalamic arcuate nucleus (ARC) and anteroventral periventricular nucleus (AVPV) in NR gilts [3]. This likely contributed to the lower expression of FSH and LH in the pituitary found in this study.

In conclusion, this study provides evidence at the transcriptional level to explain why postpubertal gilts terminate their reproductive functions in a nutrient restricted diet. From the GO and the KEGG analysis, it was also found that nutrient restriction had effects on immunity and growth in the gilts. Based on these results, humans should also be aware of potential risks to reproductive function and even overall health when we are on a diet.

## Supporting Information

S1 Fig Sequencing Quality Evaluation. A, pituitary sample from normally fed gilts (2.86 kg/d); B, pituitary sample from nutrient restricted gilts (1 kg/d).
(TIF)

S2 Fig Hierarchical clustering of log2FC (CON-VS-NR). The Genes shown in red were upregulated and those shown in green were downregulated in CON relative to NR gilts. See supplementary S2 Table for full details. CON denotes normally fed gilts (2.86 kg/d) and NR denotes nutrient restricted gilts (1 kg/d).
(TIF)

S1 File Pathway Map for S4 Table. (ZIP)

S1 Table Summary of differentially expressed genes identified between the control gilt (CON) and nutrient restriction gilt (NR) in the pituitary. CON denotes normally fed gilts (2.86 kg/d) and NR denotes nutrient restricted gilts (1 kg/d).
(XLSX)

S2 Table Differentially expressed genes between the control gilts (CON) and nutrient restriction gilts (NR) in the pituitary (filtered). CON denotes normally fed gilts (2.86 kg/d) and NR denotes nutrient restricted gilts (1 kg/d).
(XLSX)

S3 Table Gene Ontology Analysis of differentially expressed genes in the pituitary between the control (CON) and nutrient restricted (NR) gilts; S3.1 Table. Terms from the Function Ontology with p-value equal to or less than 1 (Terms for CON-versus-NR); S3.2 Table. Terms from the Process Ontology with p-value equal to or less than 1 (Terms for CON-VS-NR); S3.3 Table. Terms from the Component Ontology with p-value equal to or less than 1 (Terms for CON-VS-NR). CON denotes normally fed gilts (2.86 kg/d) and NR denotes nutrient restricted gilts (1 kg/d).
(XLSX)

S4 Table The result of Illumina sequencing for pathway analysis of differentially expressed genes based on the Kyoto Encyclopedia of Genes and Genomes (KEGG) database. The Genes shown in red box were upregulated and those shown in green box were downregulated in CON relative to NR gilts in the hyperlink pathway figure. CON denotes normally fed gilts (2.86 kg/d) and NR denotes nutrient restricted gilts (1 kg/d).
(DOCX)
