# Supplementary figures and images for: Reproductive Hormone and Transcriptomic Responses of Pituitary Tissue in Anestrus Gilts Induced by Nutrient Restriction (part 1 of 2)
Source: PLoS One. 2015 Nov 18;10(11):e0143219. doi: 10.1371/journal.pone.0143219 (PMC4651501; doi:10.1371/journal.pone.0143219)

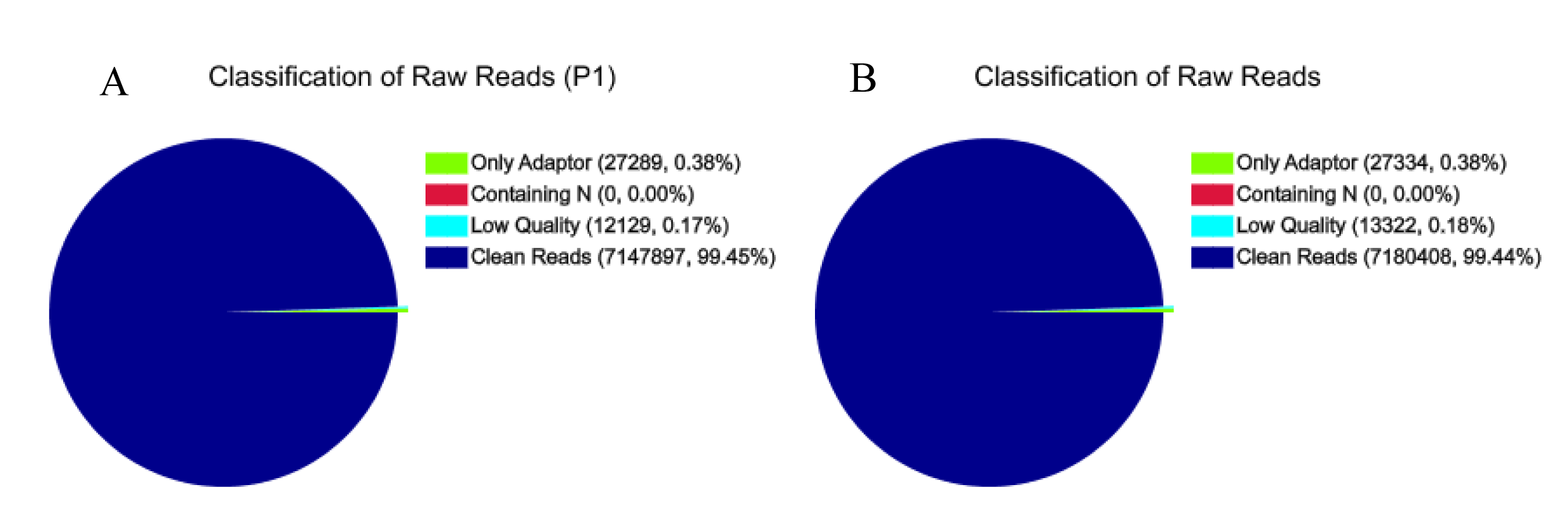

Supplement: S1 Fig — A, pituitary sample from normally fed gilts (2.86 kg/d); B, pituitary sample from nutrient restricted gilts (1 kg/d). (TIF) [file pone.0143219.s001.tif]

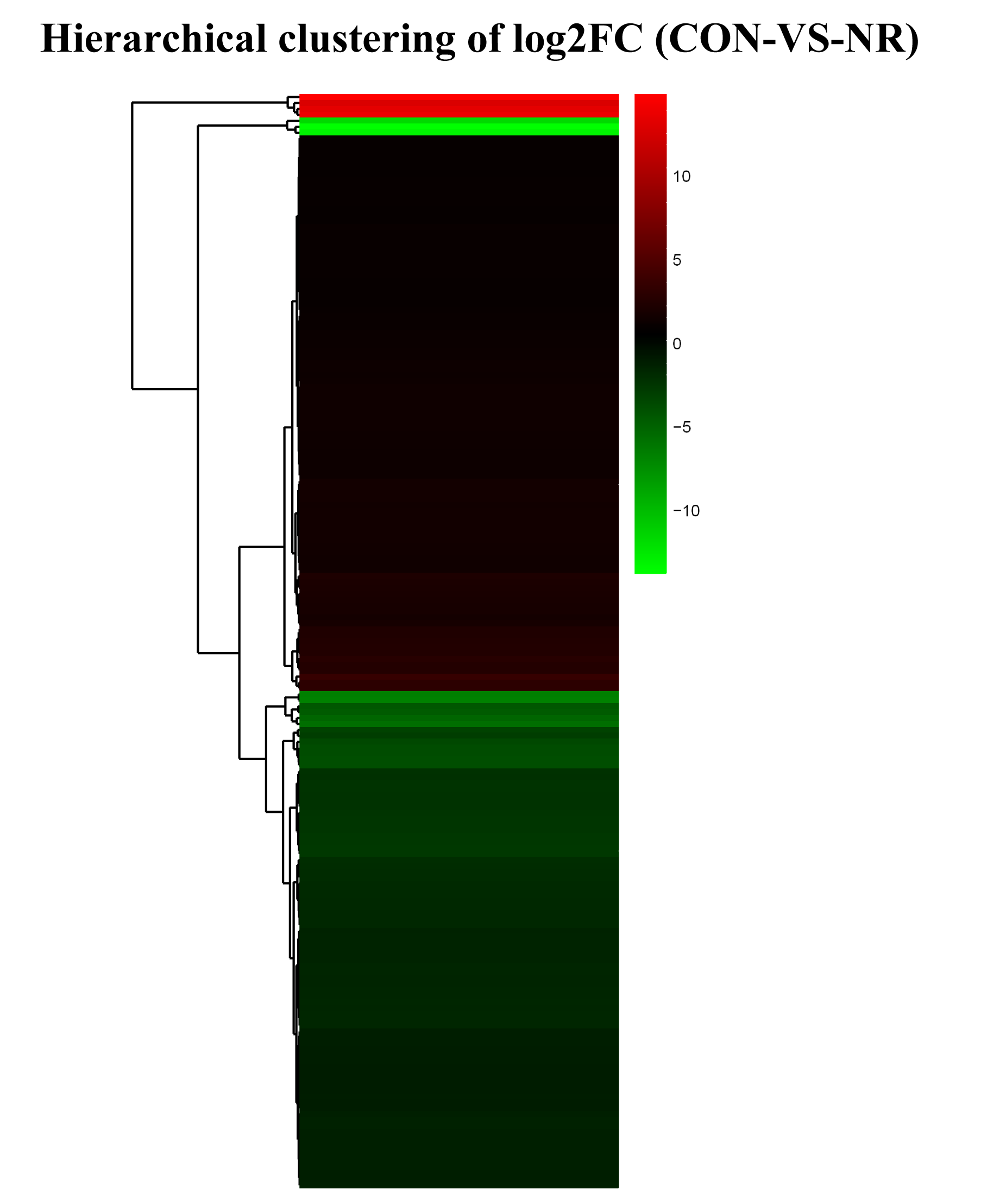

Supplement: S2 Fig — The Genes shown in red were upregulated and those shown in green were downregulated in CON relative to NR gilts. See supplementary S2 Table for full details. CON denotes normally fed gilts (2.86 kg/d) and NR denotes nutrient restricted gilts (1 kg/d). (TIF) [file pone.0143219.s002.tif]

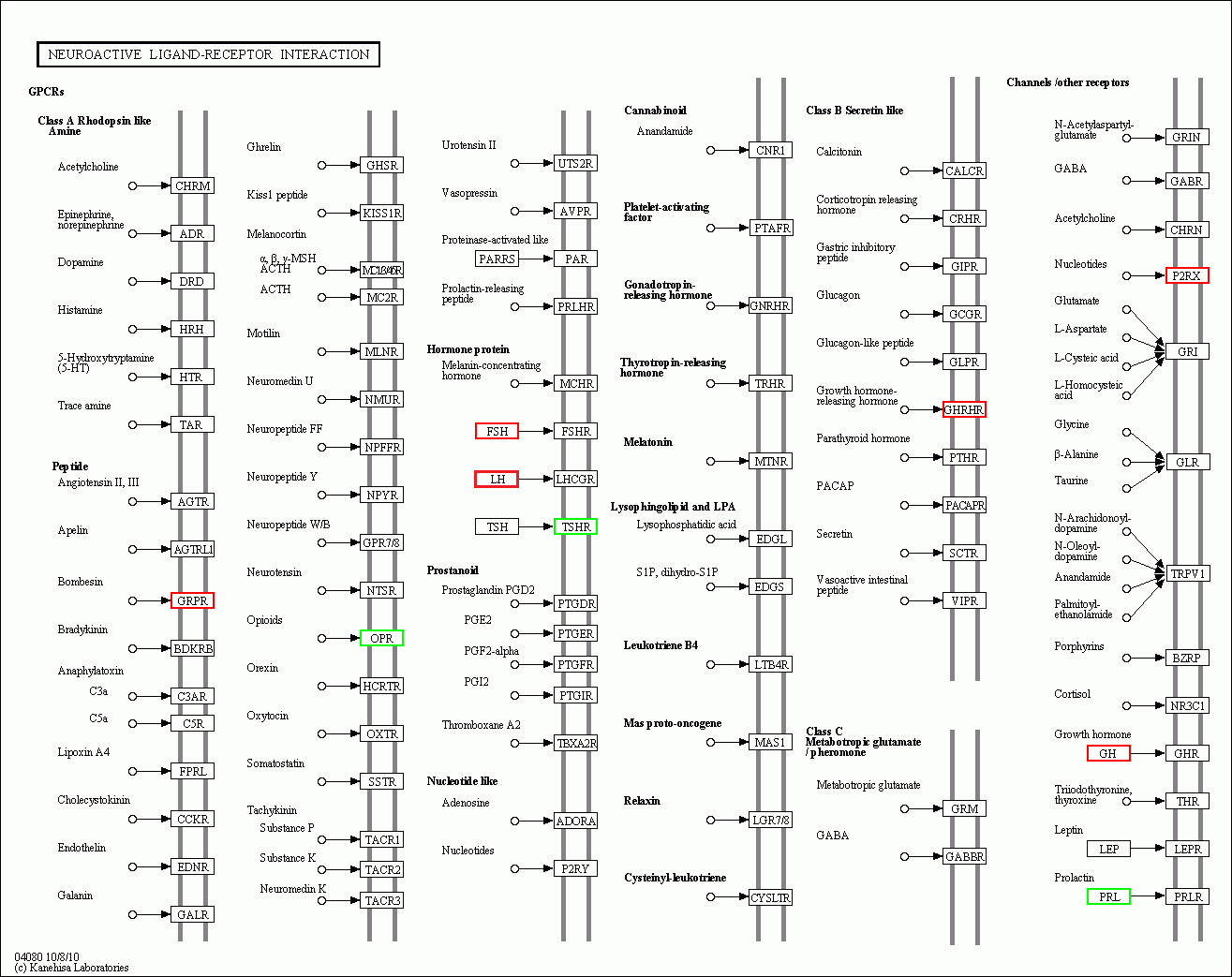

Supplement: S1 File — (ZIP) [file pone.0143219.s003.zip › pathway map/1 map04080.png]

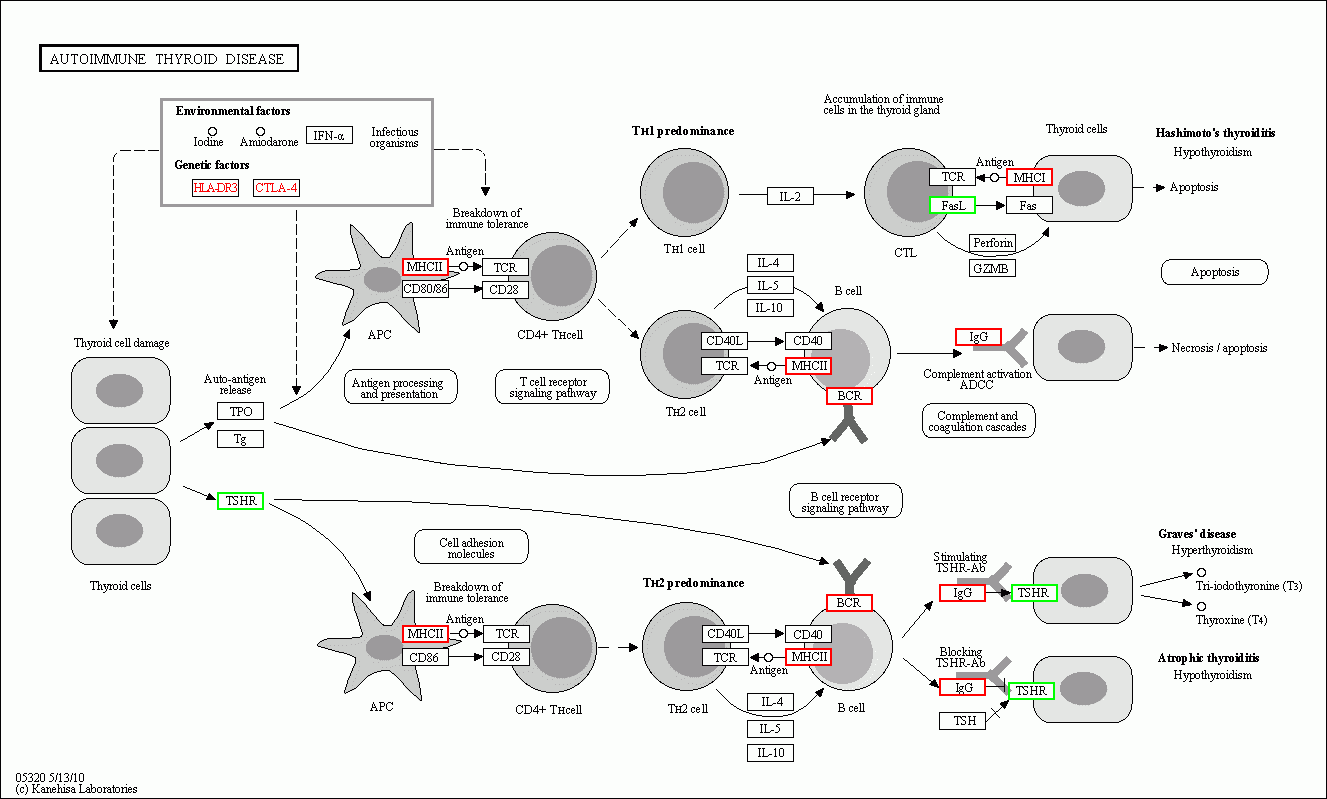

Supplement: S1 File — (ZIP) [file pone.0143219.s003.zip › pathway map/10 map05320.png]

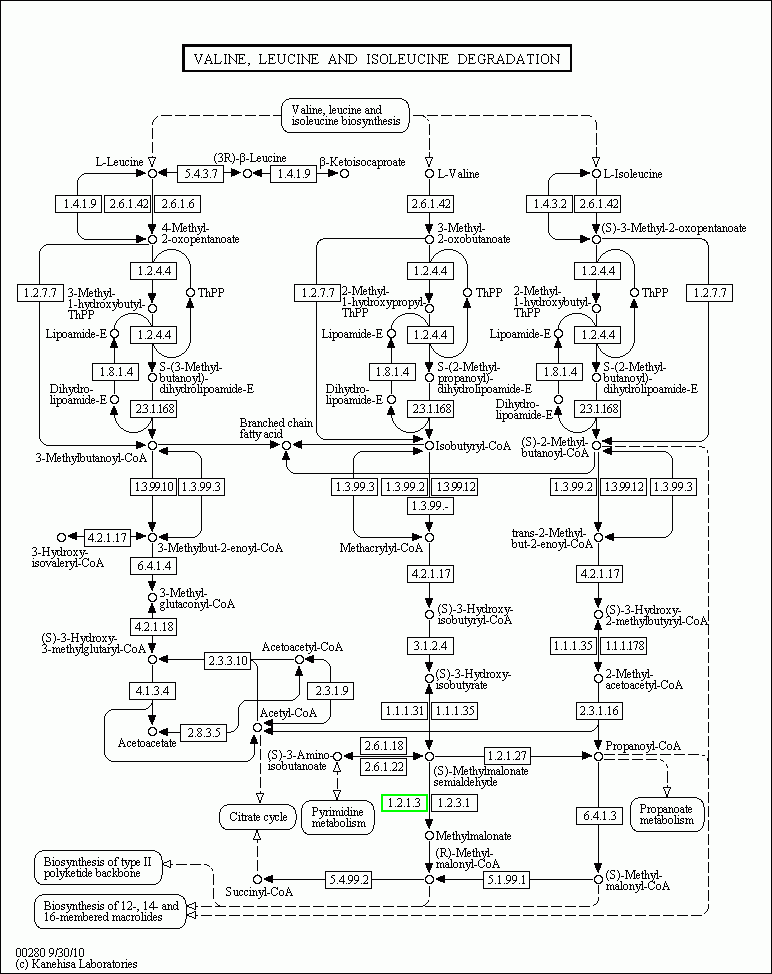

Supplement: S1 File — (ZIP) [file pone.0143219.s003.zip › pathway map/100 map00280.png]

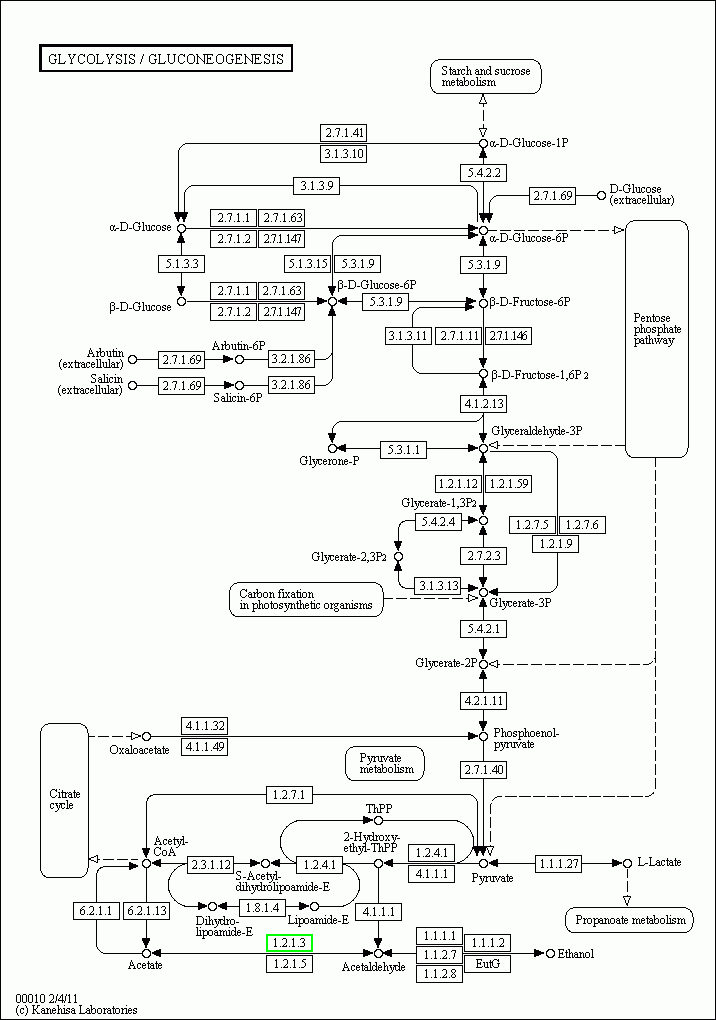

Supplement: S1 File — (ZIP) [file pone.0143219.s003.zip › pathway map/101 map00010.png]

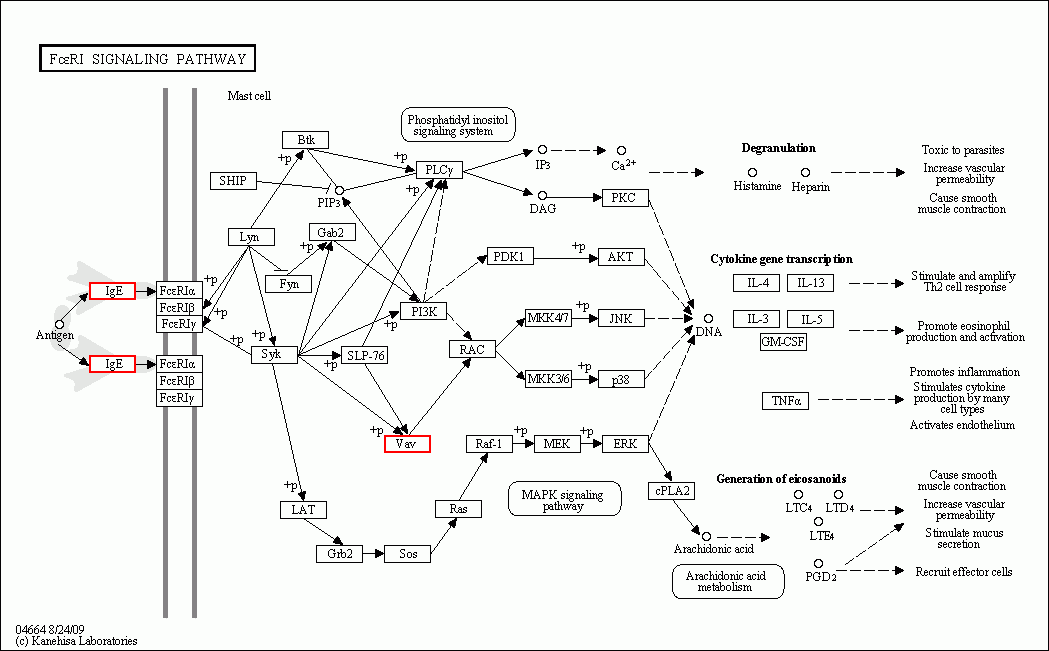

Supplement: S1 File — (ZIP) [file pone.0143219.s003.zip › pathway map/103 map04664.png]

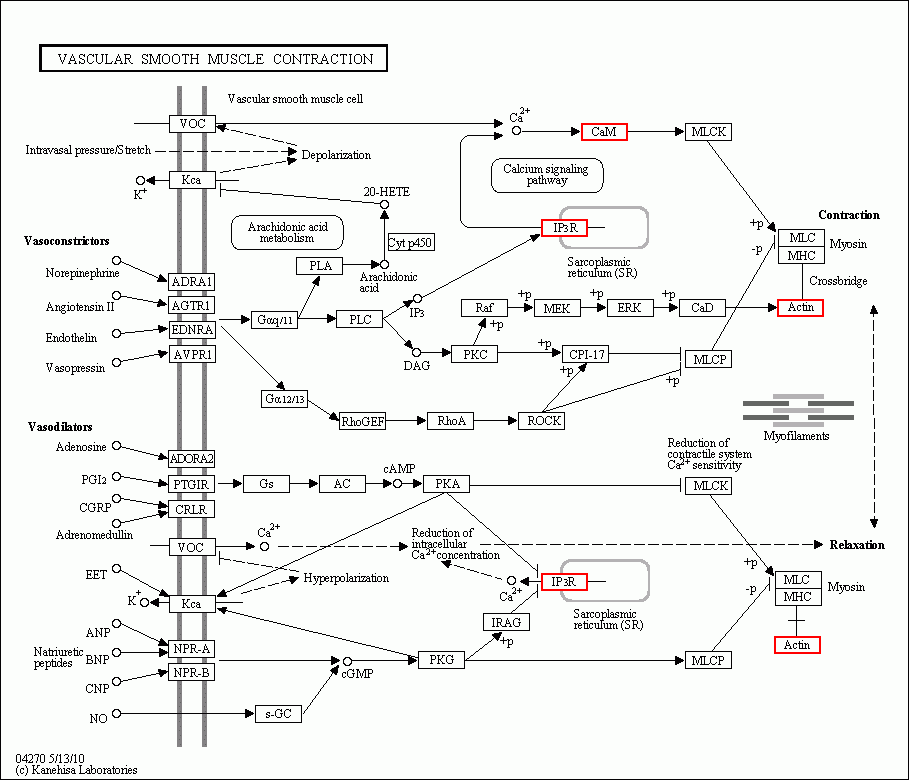

Supplement: S1 File — (ZIP) [file pone.0143219.s003.zip › pathway map/104 map04270.png]

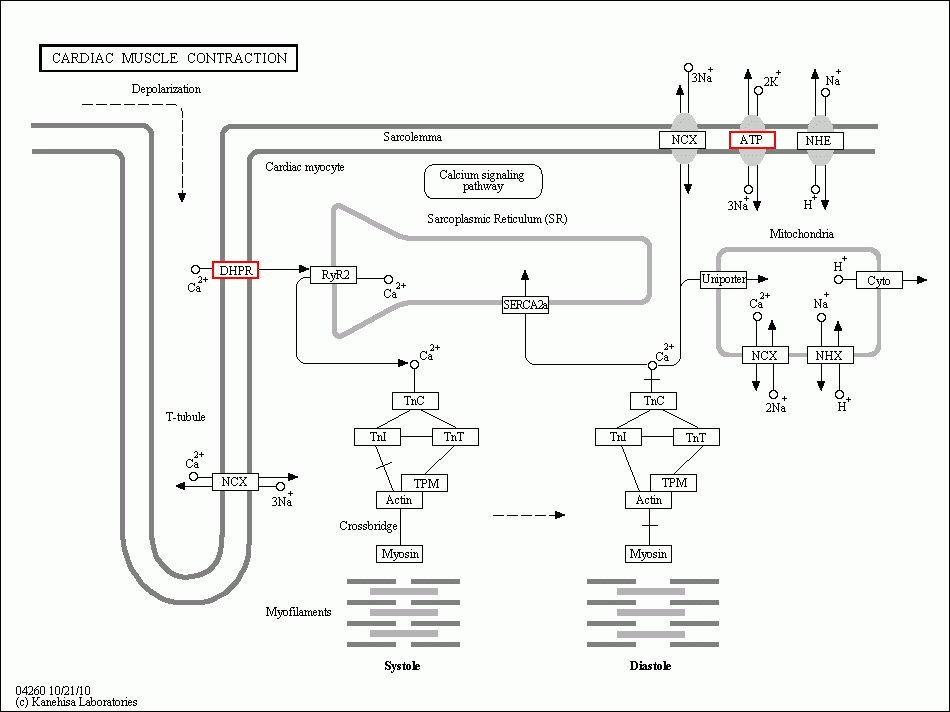

Supplement: S1 File — (ZIP) [file pone.0143219.s003.zip › pathway map/105 map04260.png]

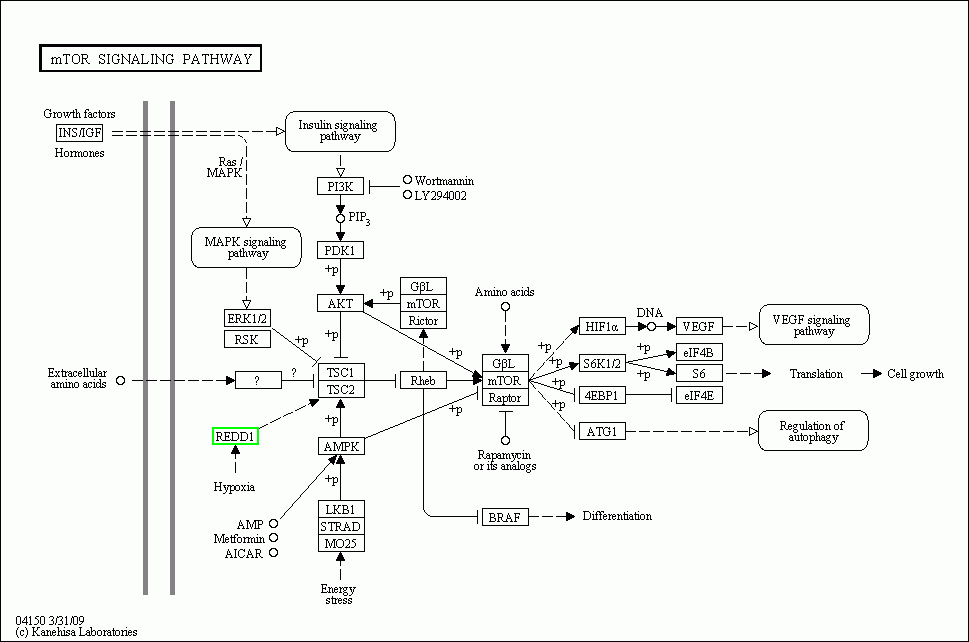

Supplement: S1 File — (ZIP) [file pone.0143219.s003.zip › pathway map/106 map04150.png]

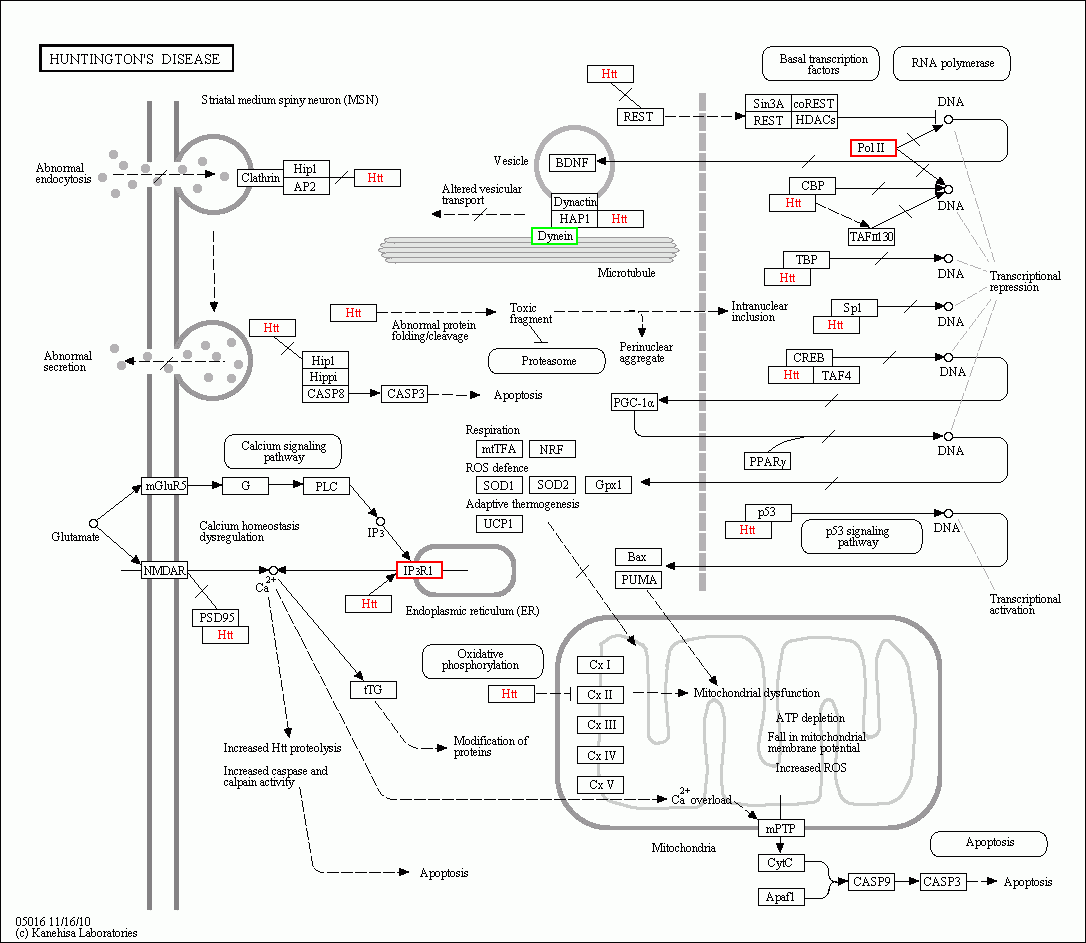

Supplement: S1 File — (ZIP) [file pone.0143219.s003.zip › pathway map/107 map05016.png]

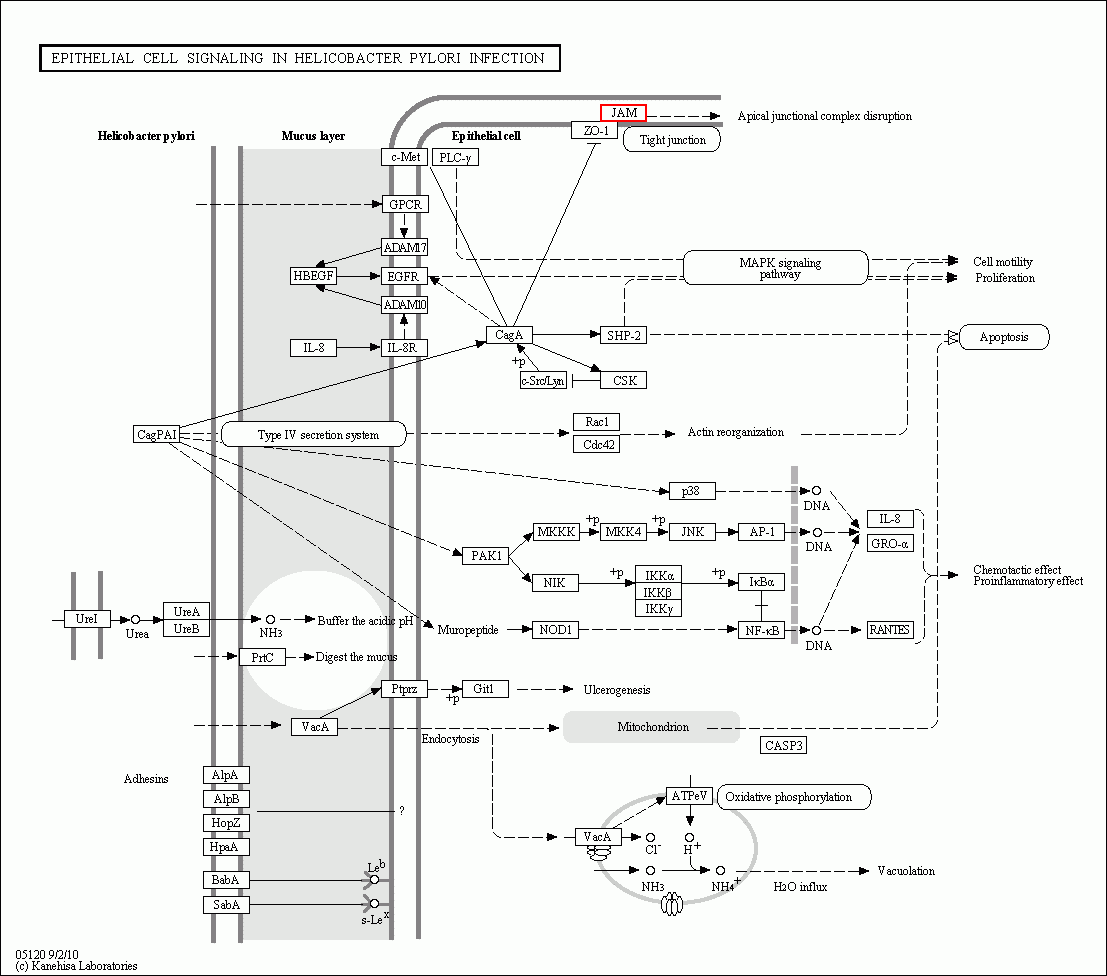

Supplement: S1 File — (ZIP) [file pone.0143219.s003.zip › pathway map/108 map05120.png]

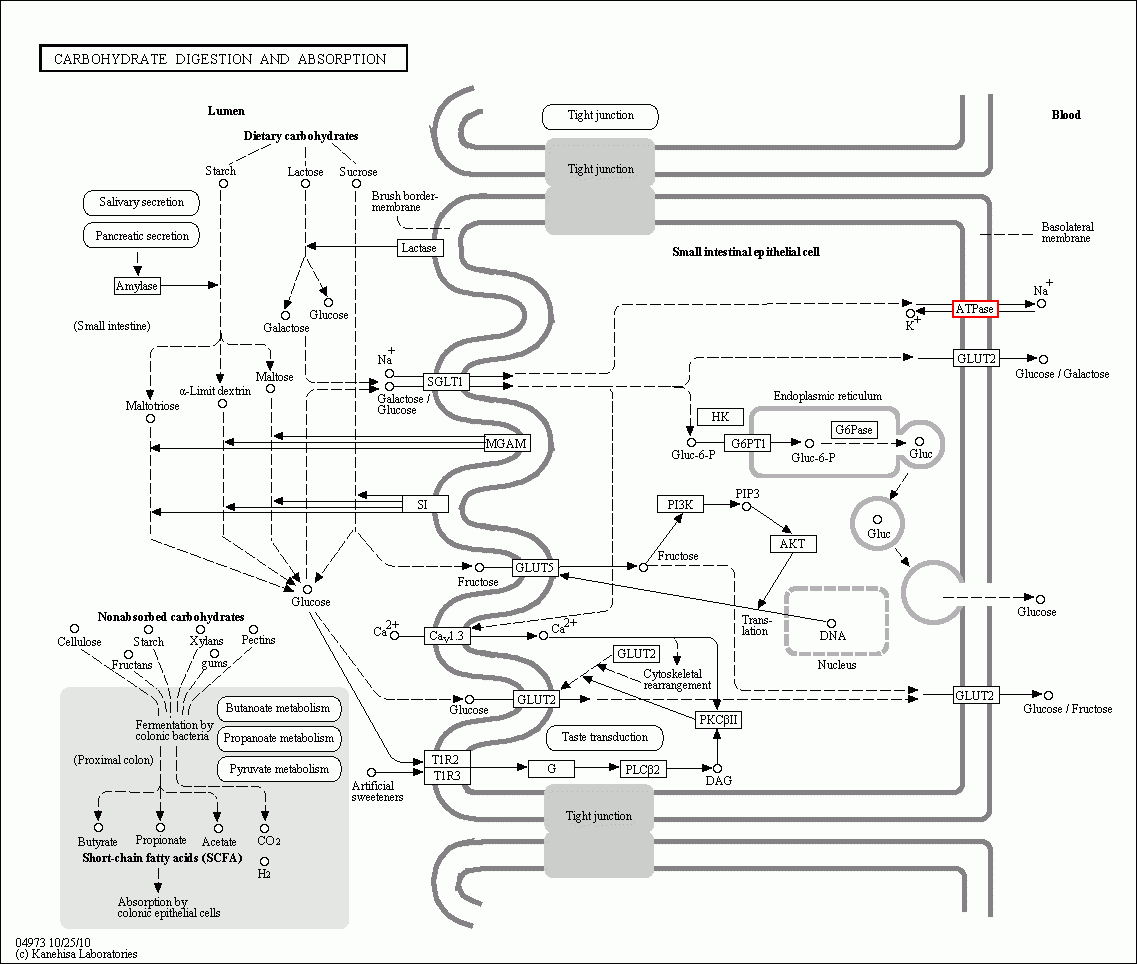

Supplement: S1 File — (ZIP) [file pone.0143219.s003.zip › pathway map/109 map04973.png]

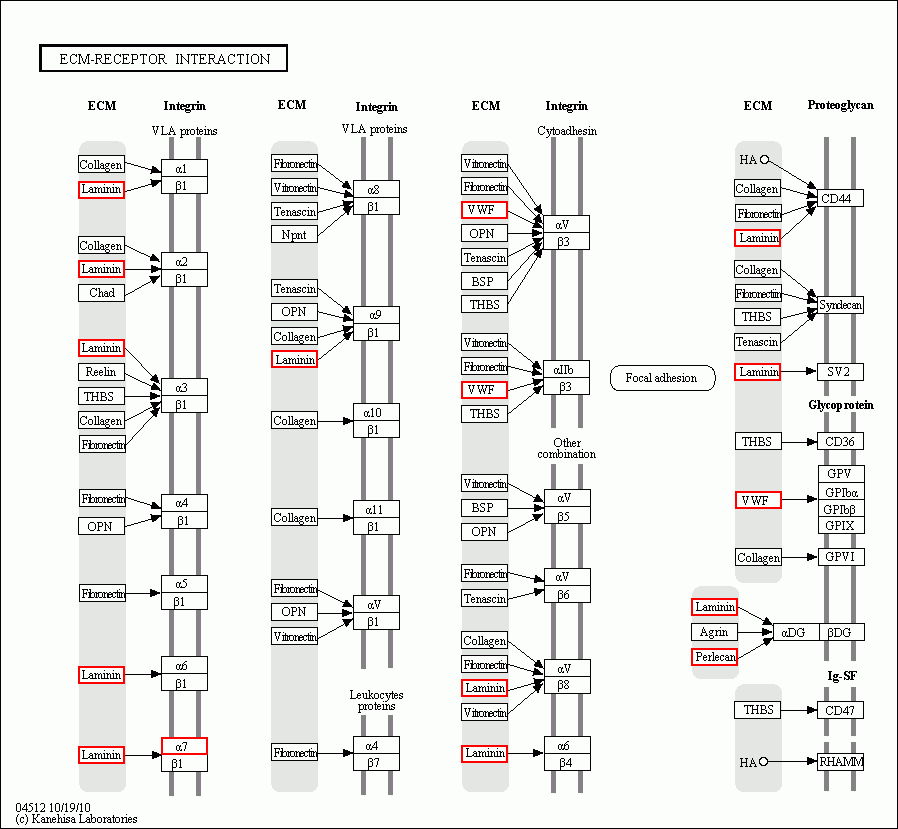

Supplement: S1 File — (ZIP) [file pone.0143219.s003.zip › pathway map/11 map04512.png]

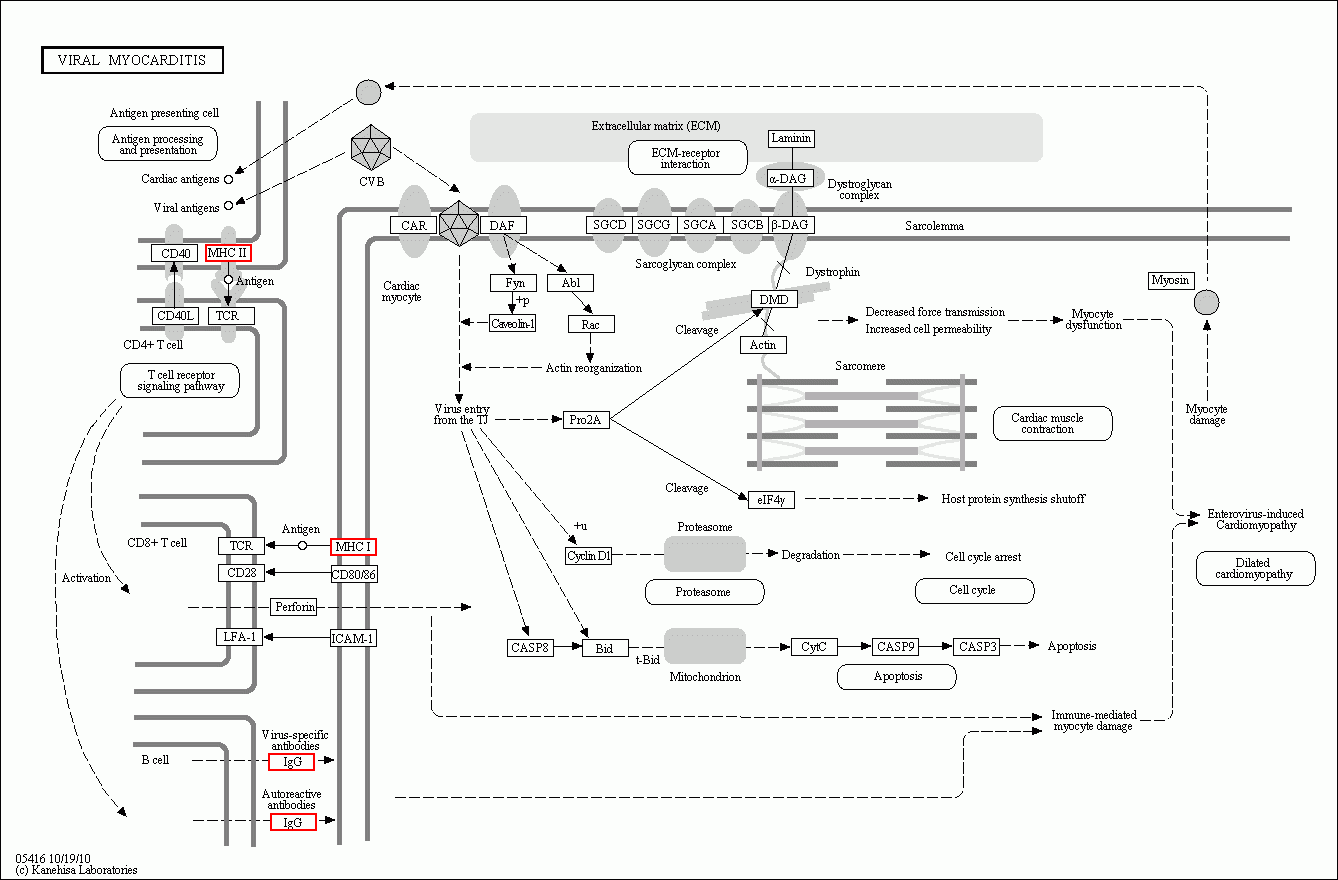

Supplement: S1 File — (ZIP) [file pone.0143219.s003.zip › pathway map/110 map05416.png]

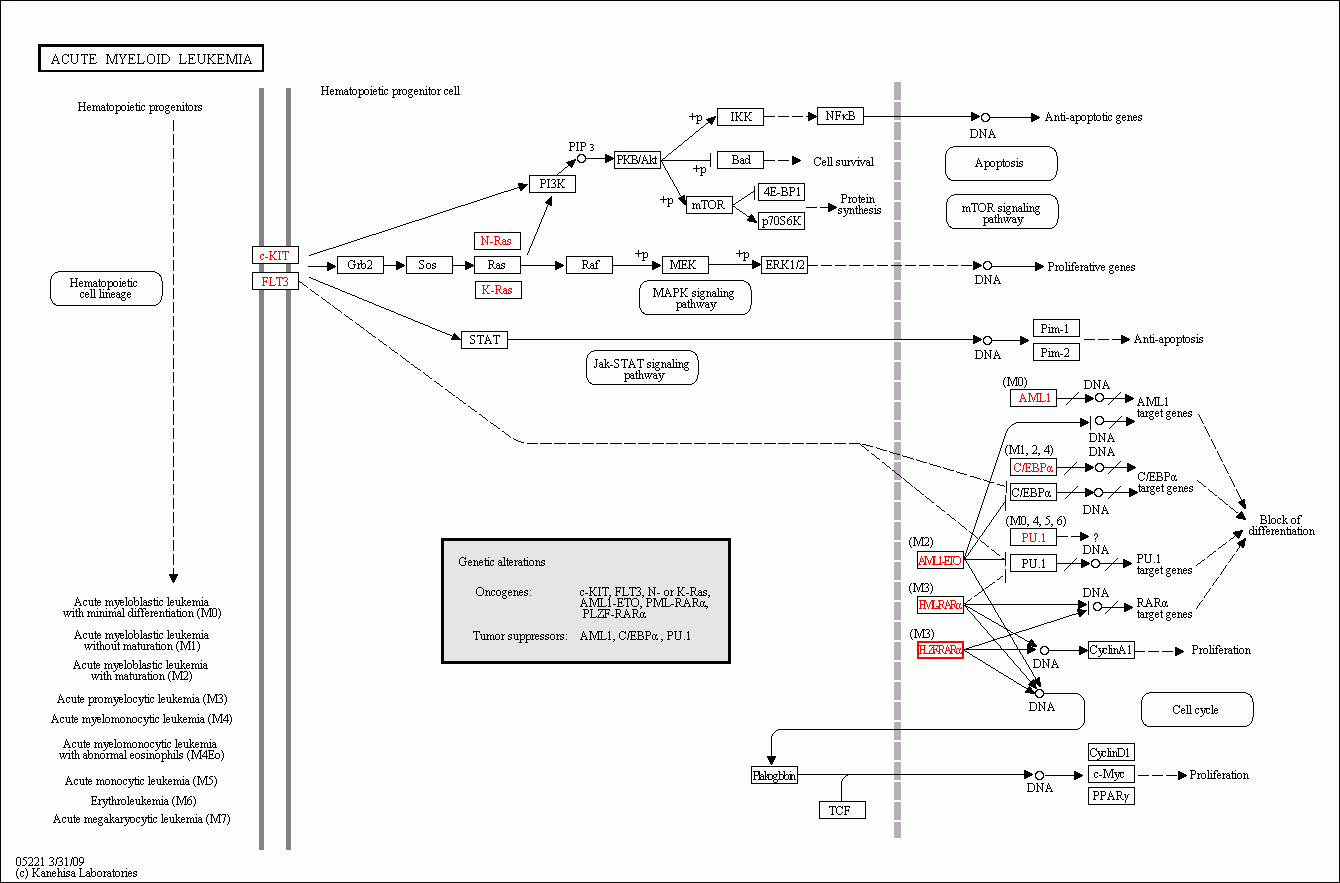

Supplement: S1 File — (ZIP) [file pone.0143219.s003.zip › pathway map/111 map05221.png]

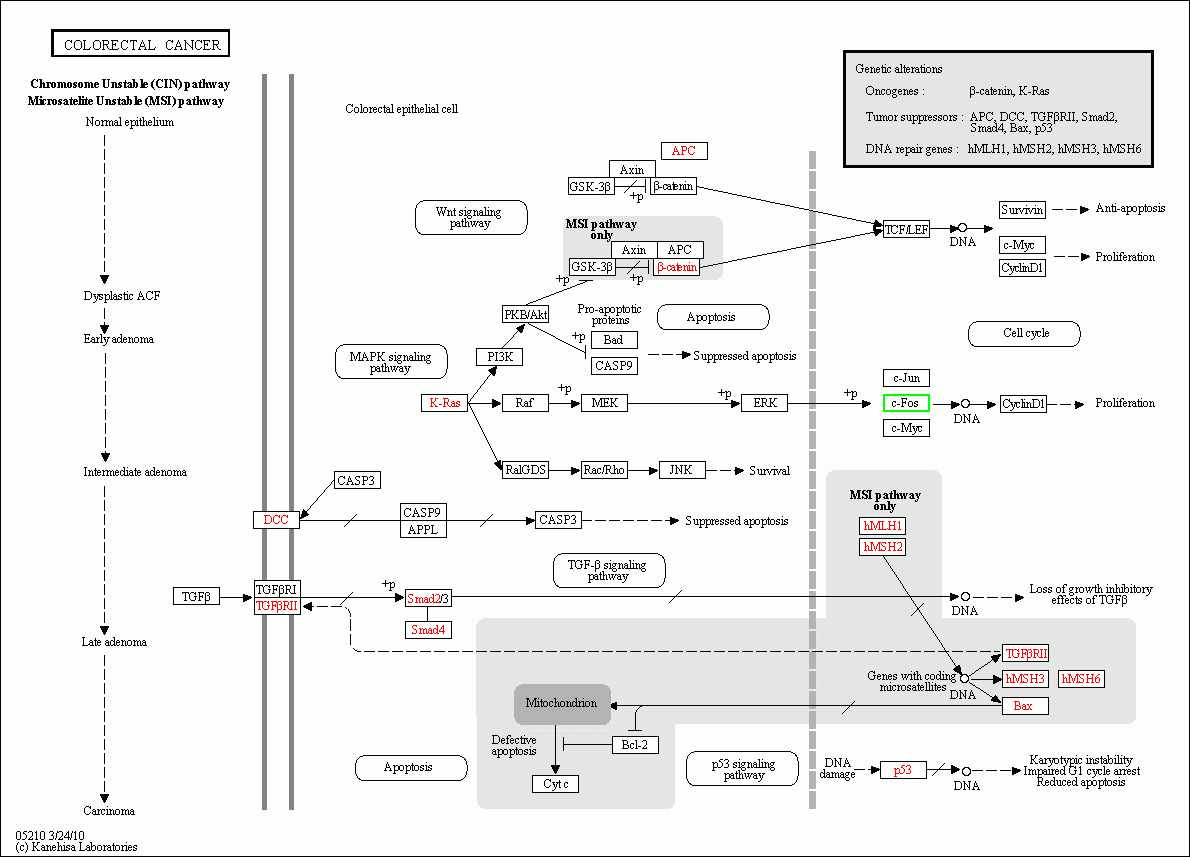

Supplement: S1 File — (ZIP) [file pone.0143219.s003.zip › pathway map/112 map05210.png]

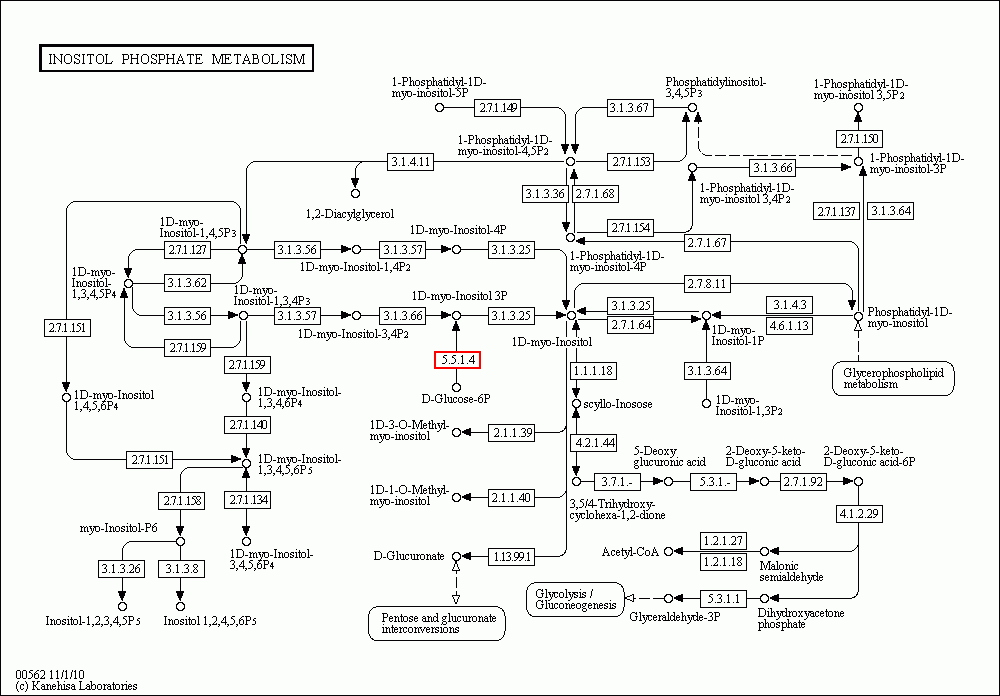

Supplement: S1 File — (ZIP) [file pone.0143219.s003.zip › pathway map/113 map00562.png]

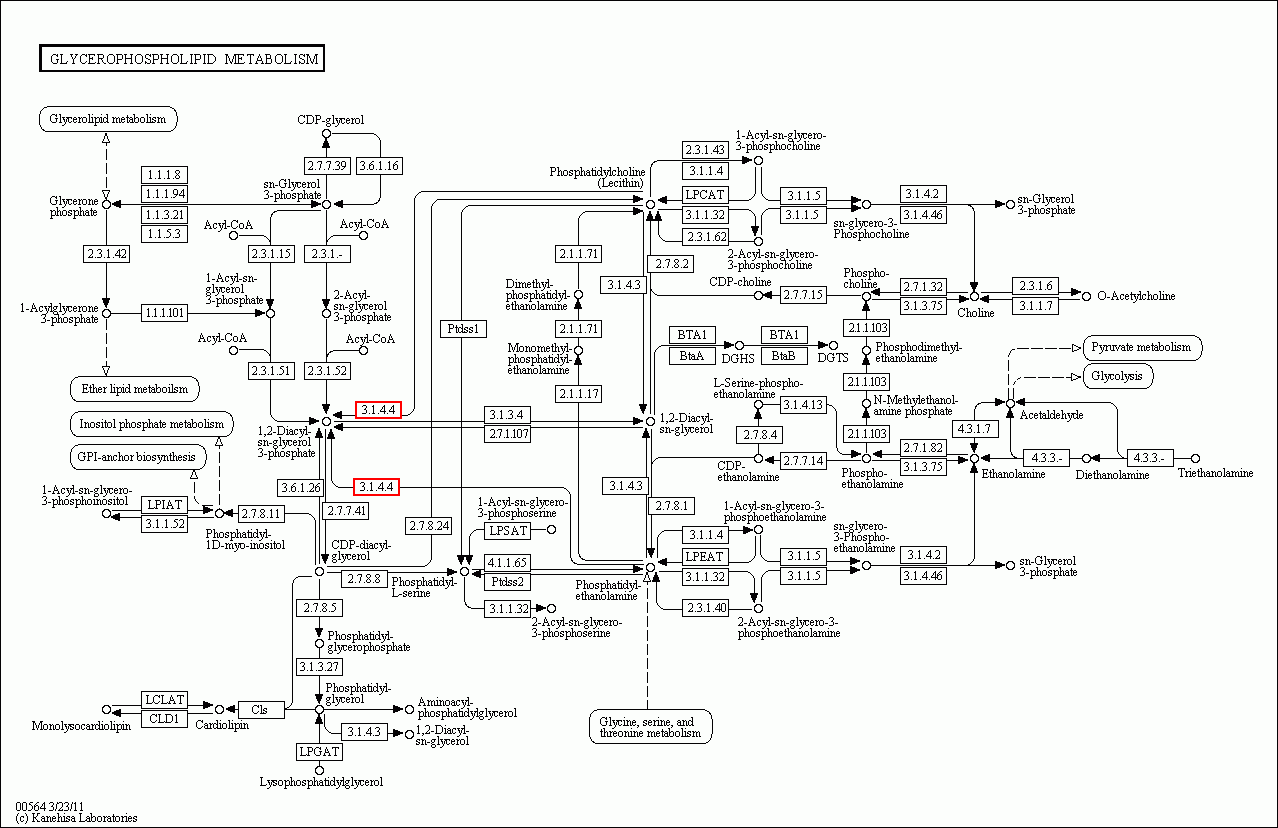

Supplement: S1 File — (ZIP) [file pone.0143219.s003.zip › pathway map/115 map00564.png]

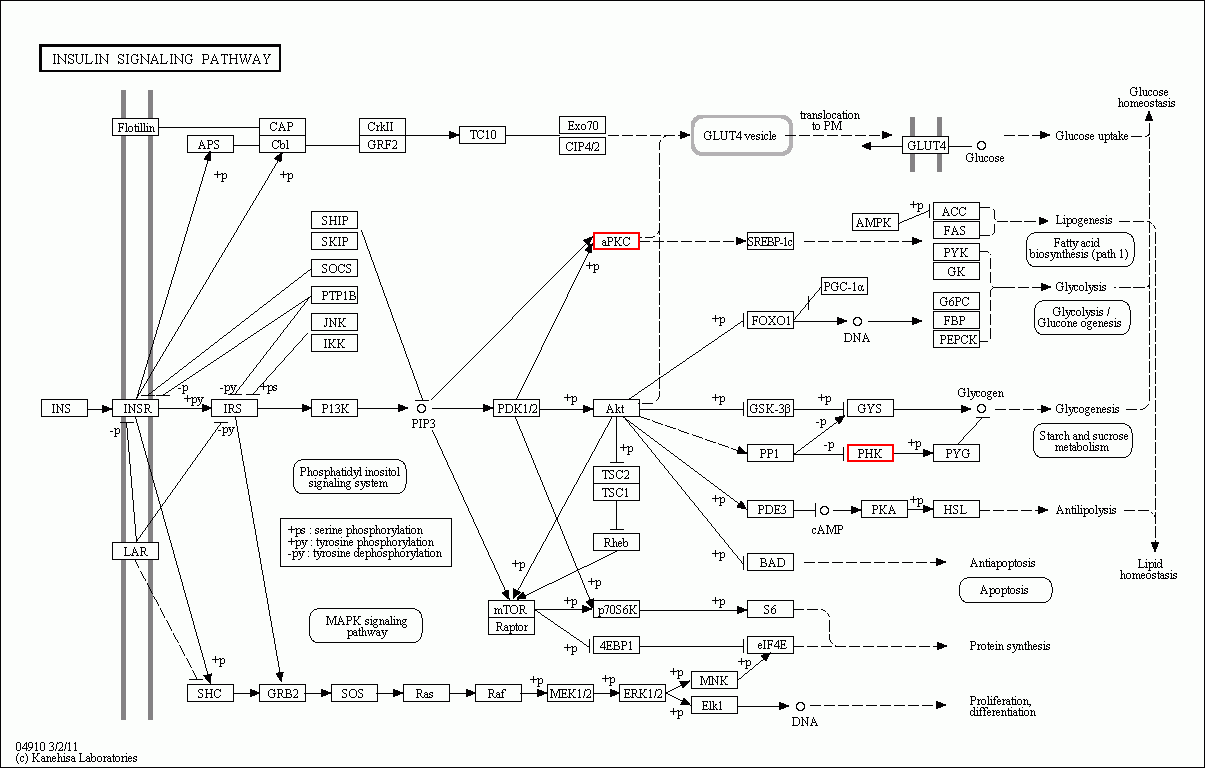

Supplement: S1 File — (ZIP) [file pone.0143219.s003.zip › pathway map/116 map04910.png]

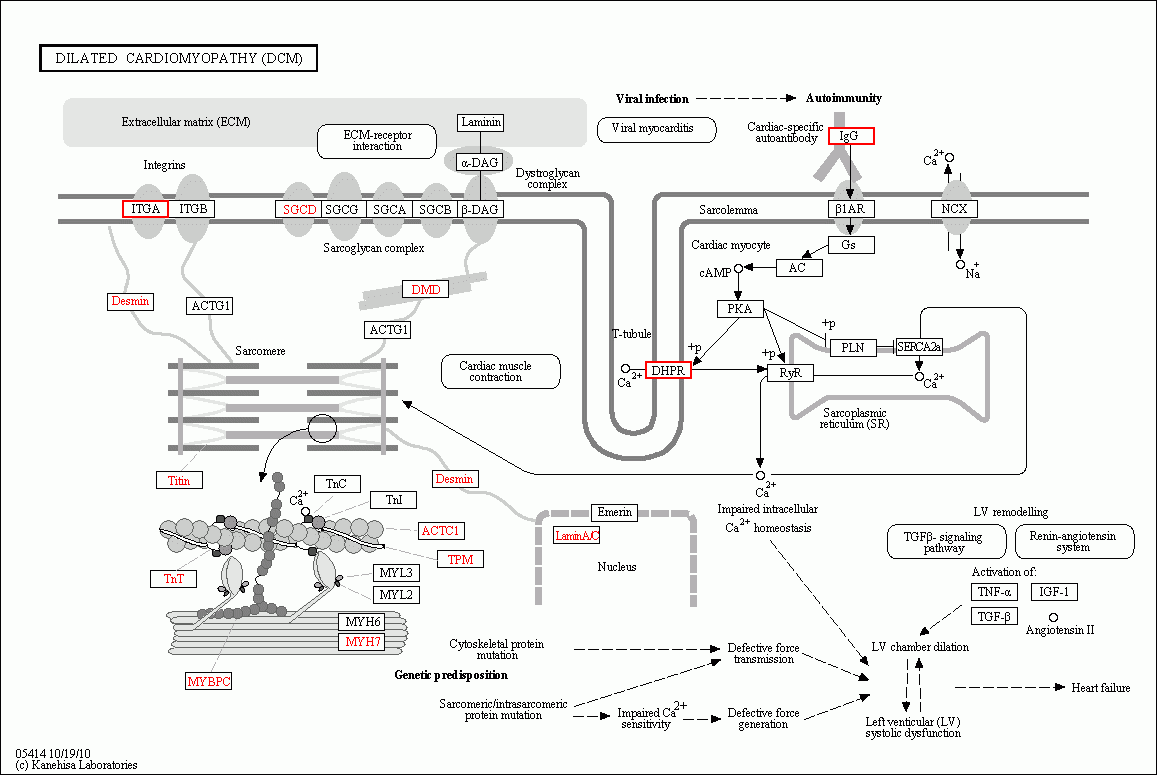

Supplement: S1 File — (ZIP) [file pone.0143219.s003.zip › pathway map/117 map05414.png]

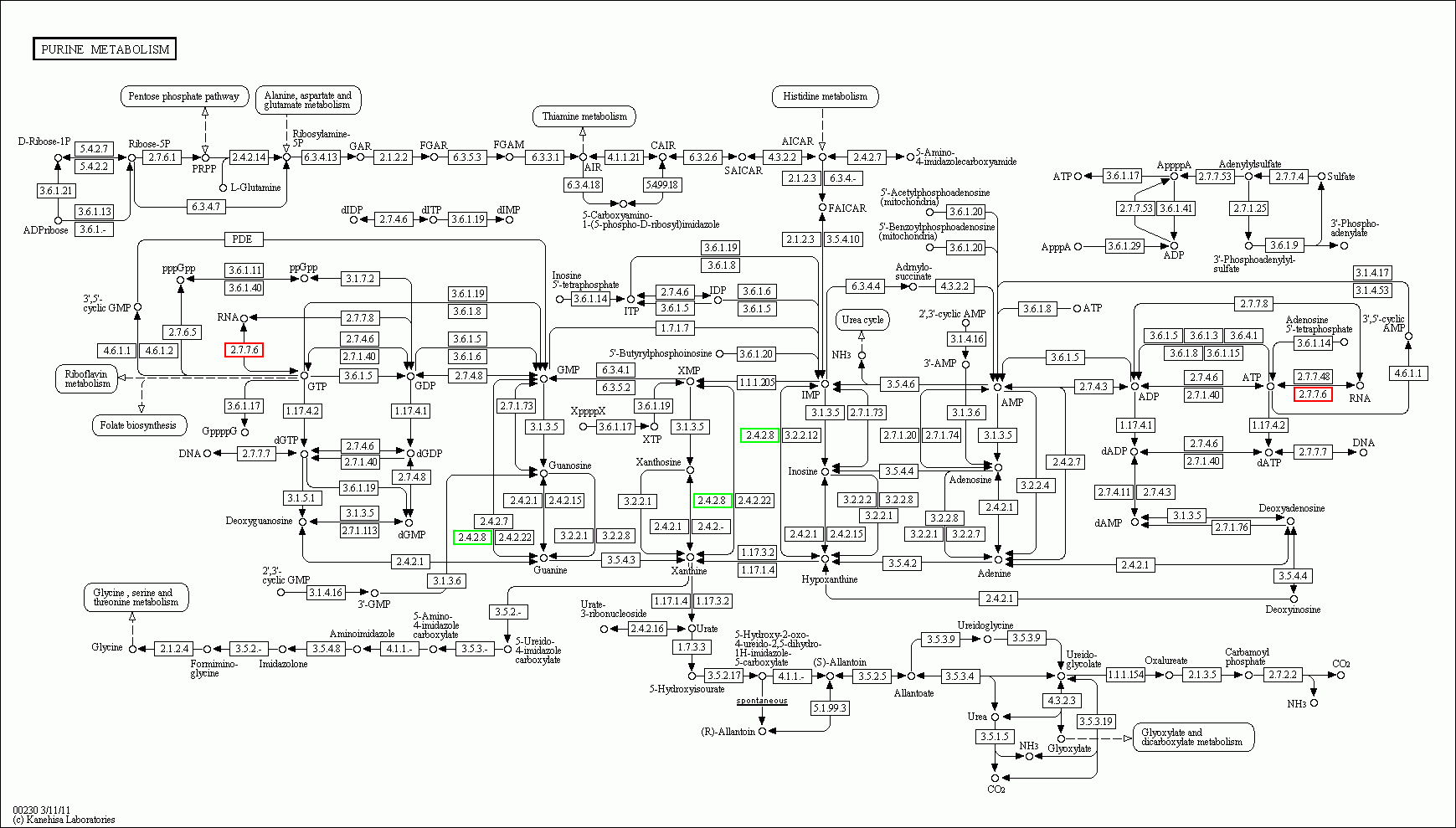

Supplement: S1 File — (ZIP) [file pone.0143219.s003.zip › pathway map/118 map00230.png]

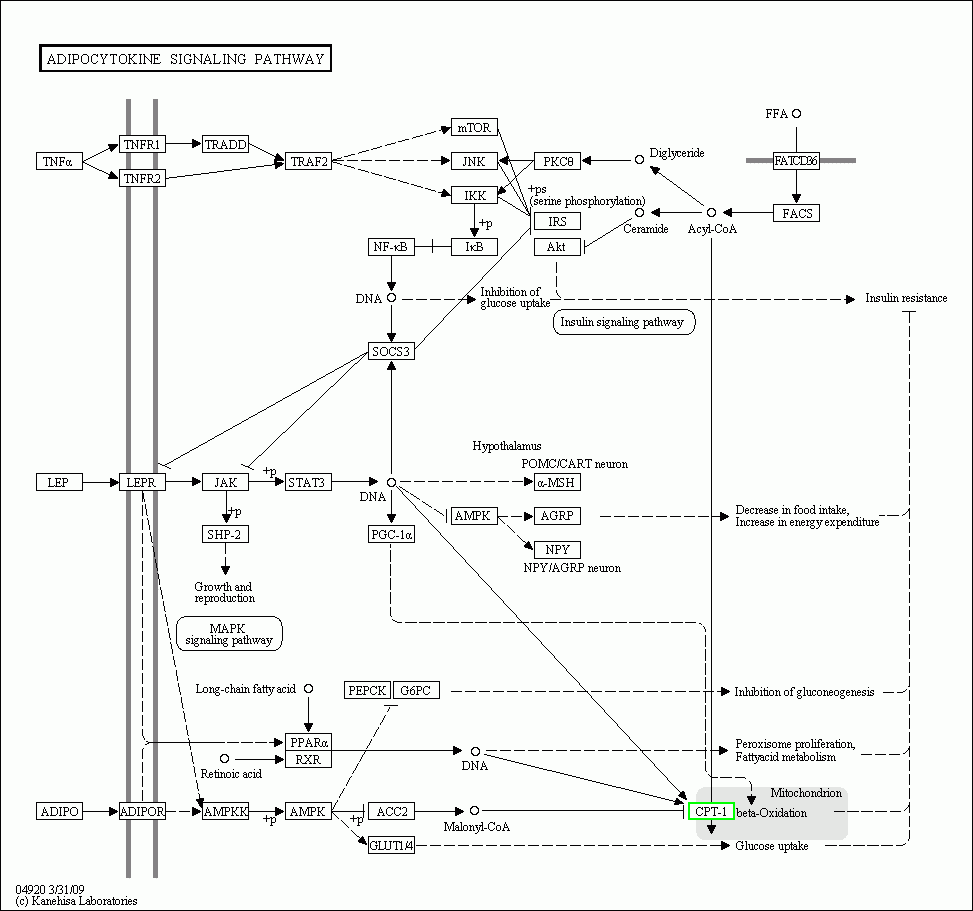

Supplement: S1 File — (ZIP) [file pone.0143219.s003.zip › pathway map/119 map04920.png]

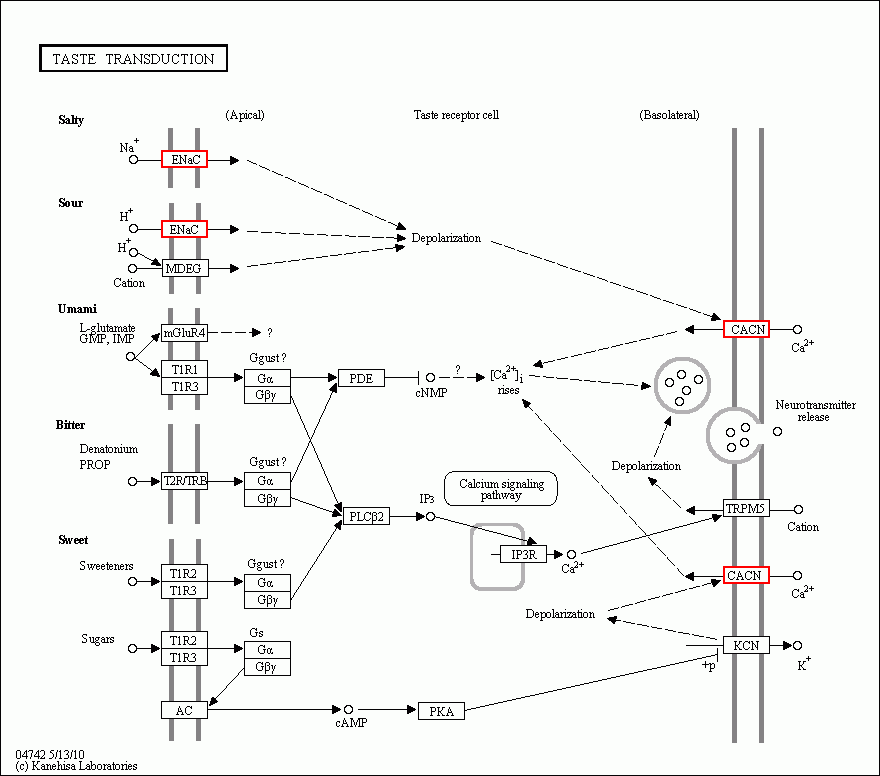

Supplement: S1 File — (ZIP) [file pone.0143219.s003.zip › pathway map/12 map04742.png]

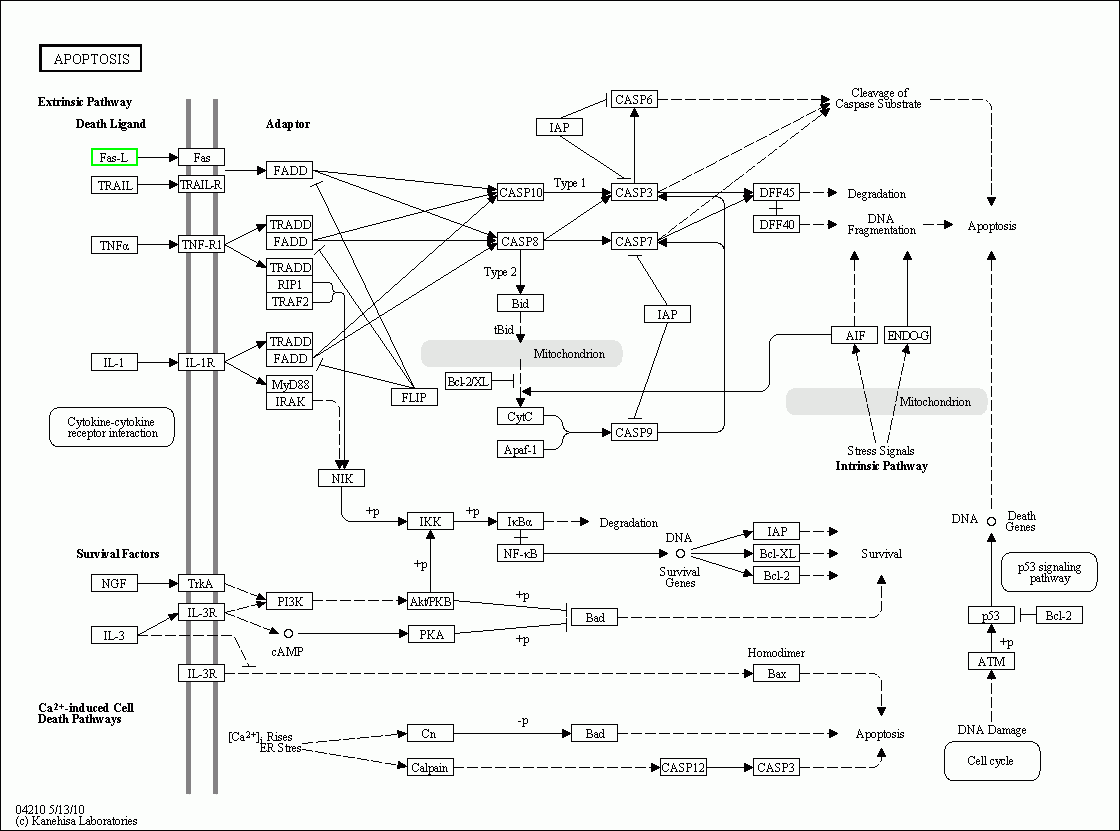

Supplement: S1 File — (ZIP) [file pone.0143219.s003.zip › pathway map/120 map04210.png]

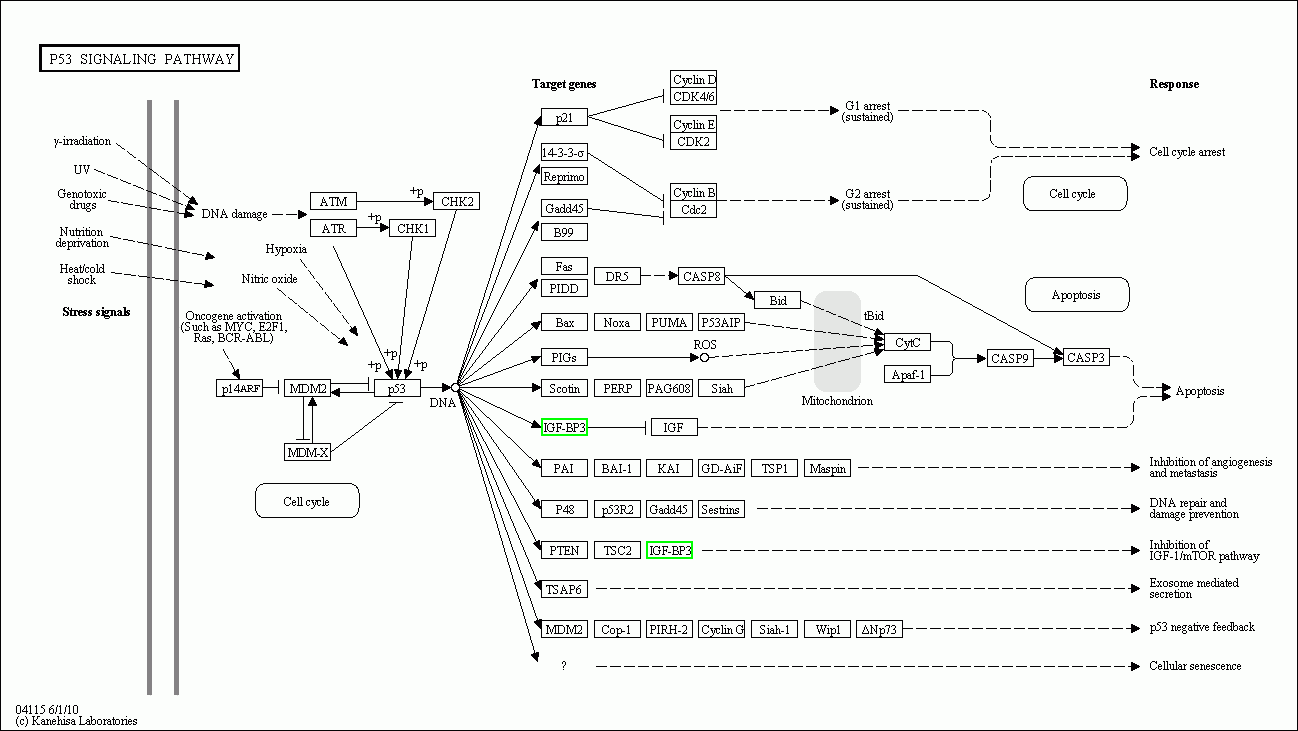

Supplement: S1 File — (ZIP) [file pone.0143219.s003.zip › pathway map/121 map04115.png]

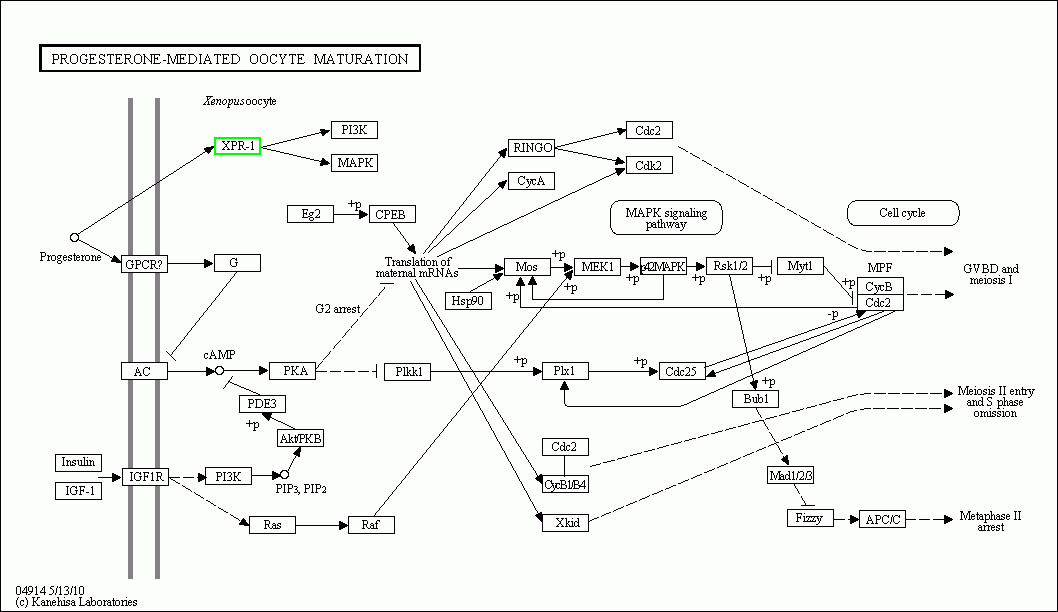

Supplement: S1 File — (ZIP) [file pone.0143219.s003.zip › pathway map/122 map04914.png]

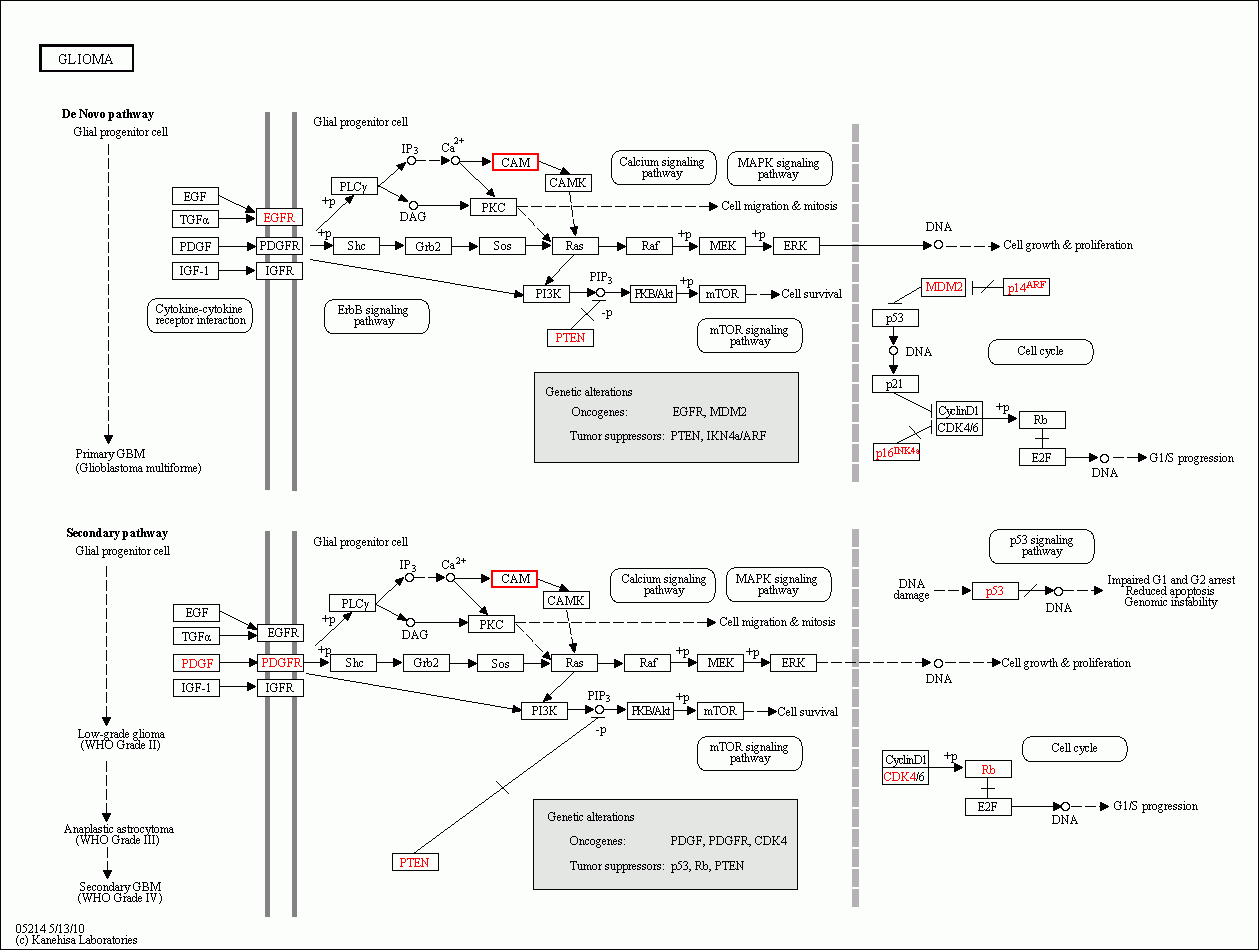

Supplement: S1 File — (ZIP) [file pone.0143219.s003.zip › pathway map/123 map05214.png]

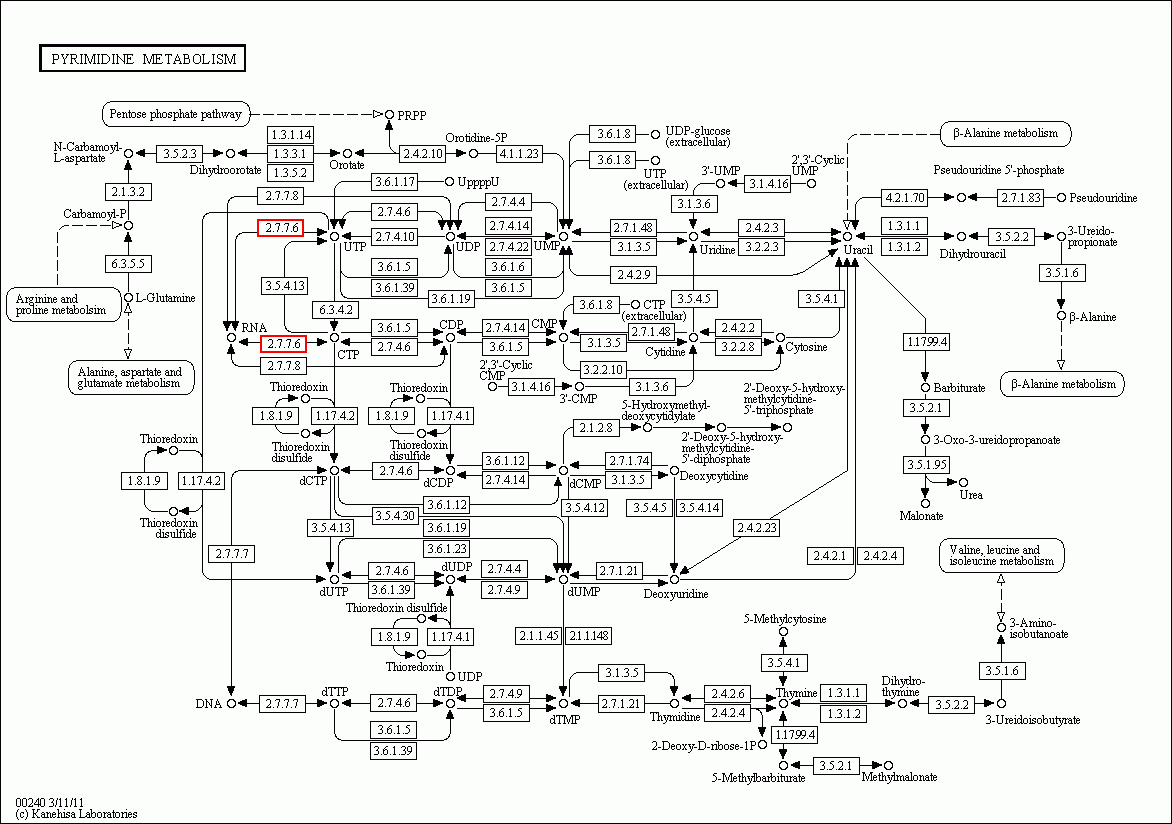

Supplement: S1 File — (ZIP) [file pone.0143219.s003.zip › pathway map/124 map00240.png]

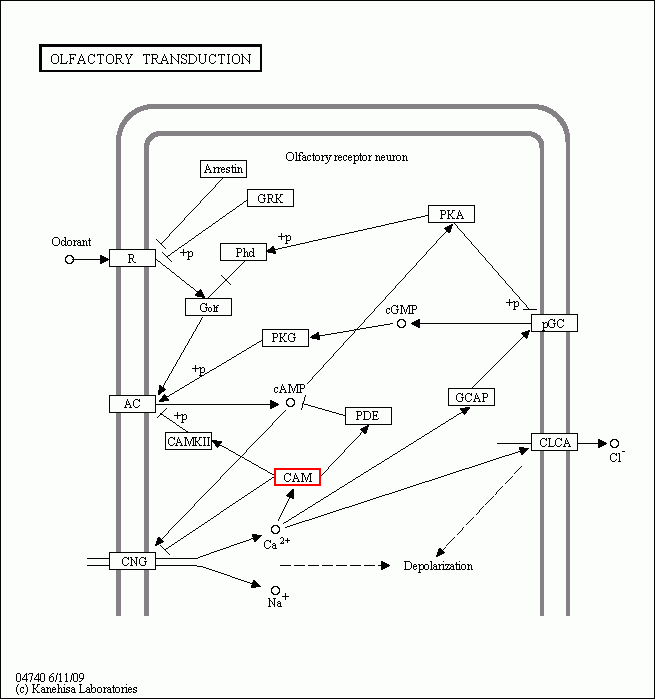

Supplement: S1 File — (ZIP) [file pone.0143219.s003.zip › pathway map/125 map04740.png]

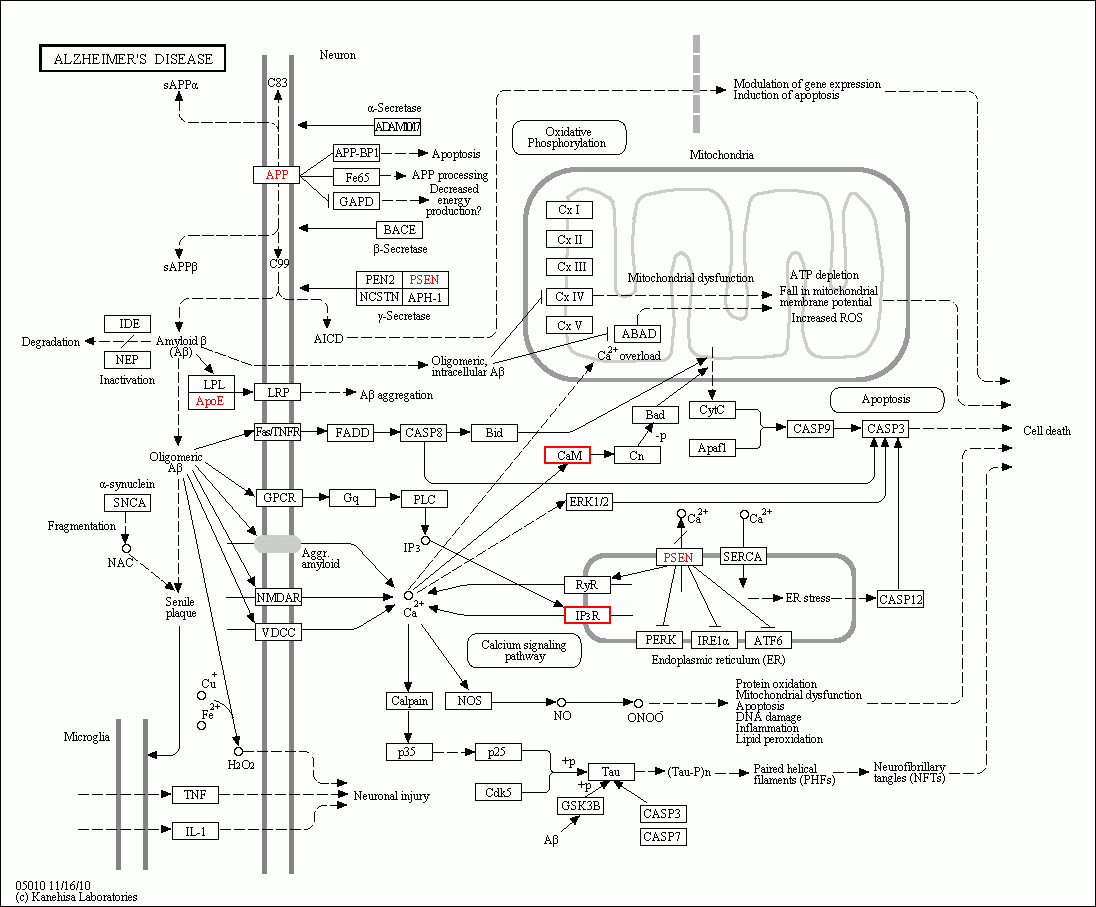

Supplement: S1 File — (ZIP) [file pone.0143219.s003.zip › pathway map/126 map05010.png]

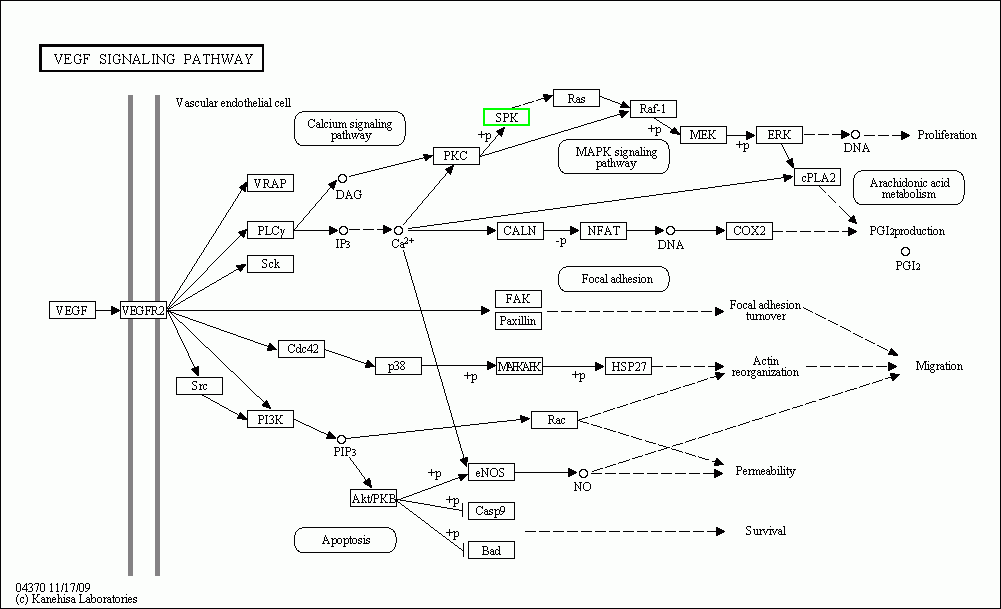

Supplement: S1 File — (ZIP) [file pone.0143219.s003.zip › pathway map/127 map04370.png]

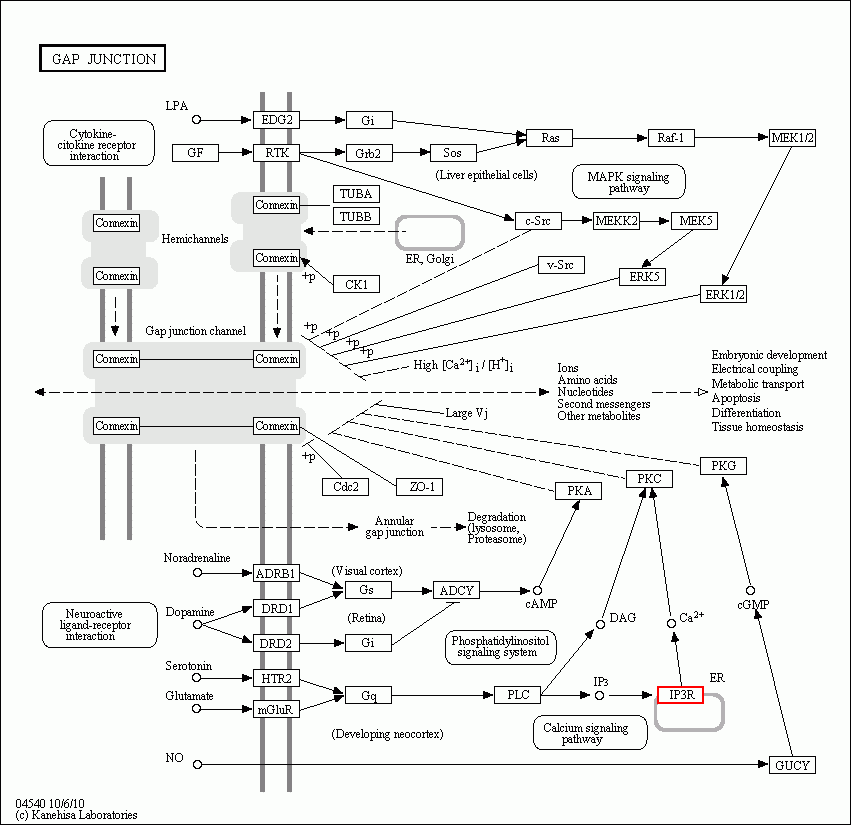

Supplement: S1 File — (ZIP) [file pone.0143219.s003.zip › pathway map/128 map04540.png]

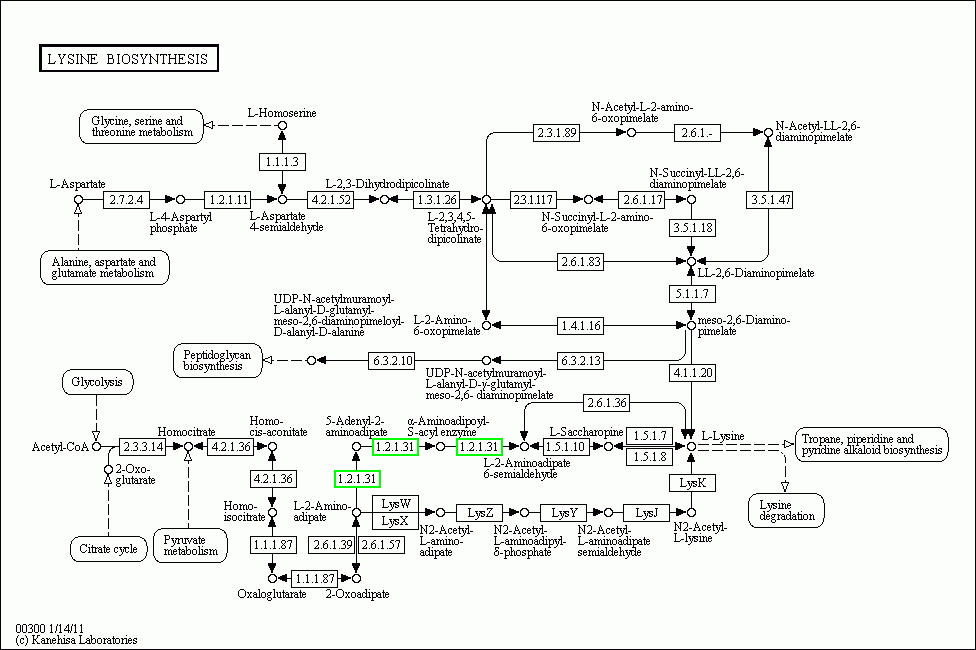

Supplement: S1 File — (ZIP) [file pone.0143219.s003.zip › pathway map/13 map00300.png]

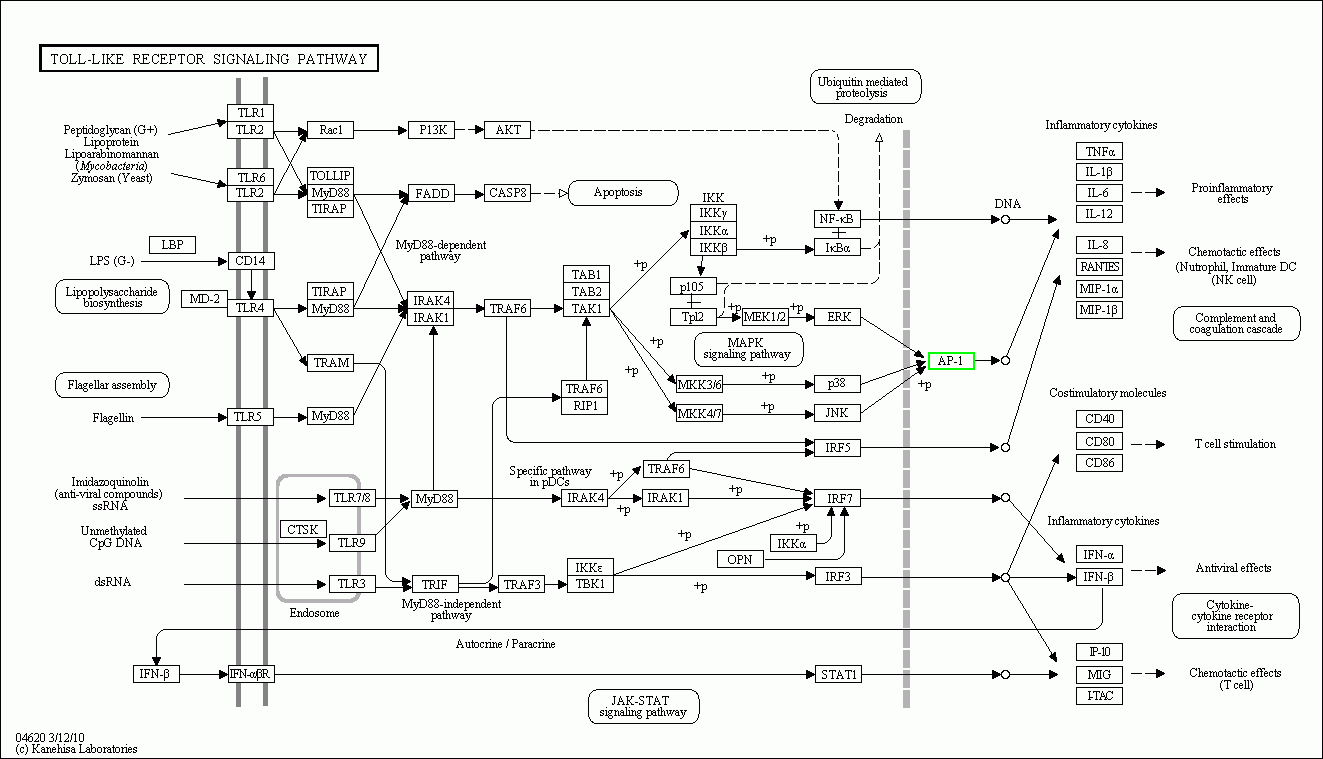

Supplement: S1 File — (ZIP) [file pone.0143219.s003.zip › pathway map/130 map04620.png]

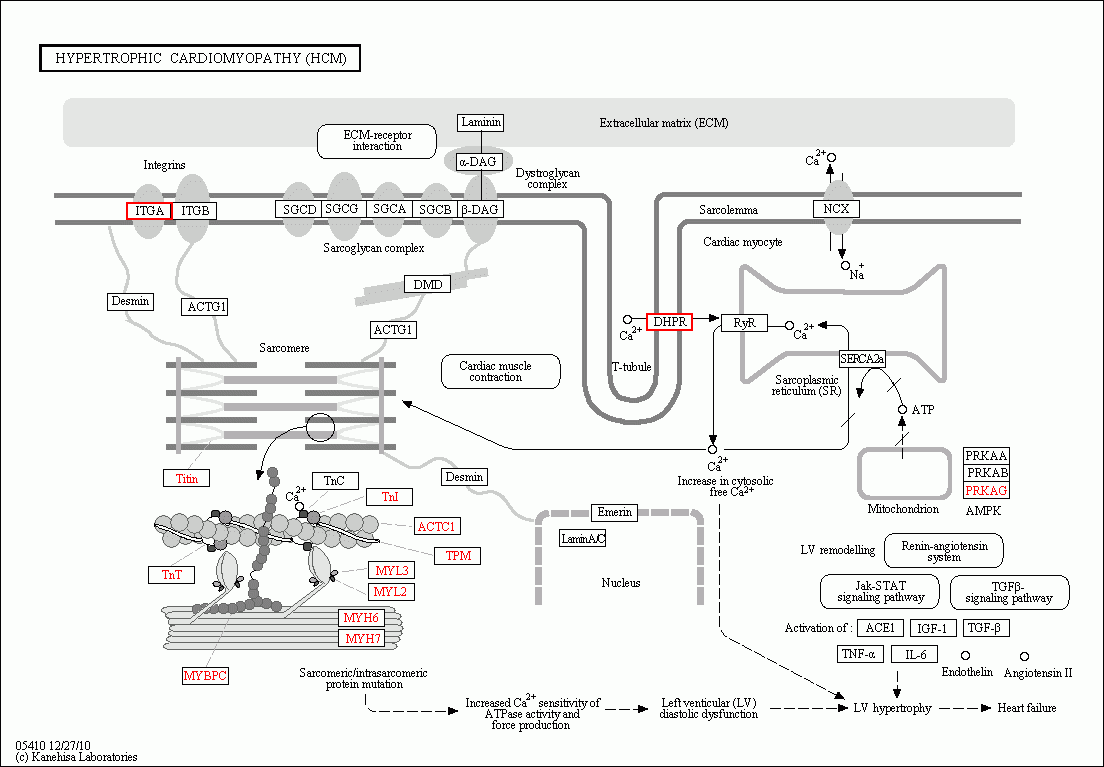

Supplement: S1 File — (ZIP) [file pone.0143219.s003.zip › pathway map/131 map05410.png]

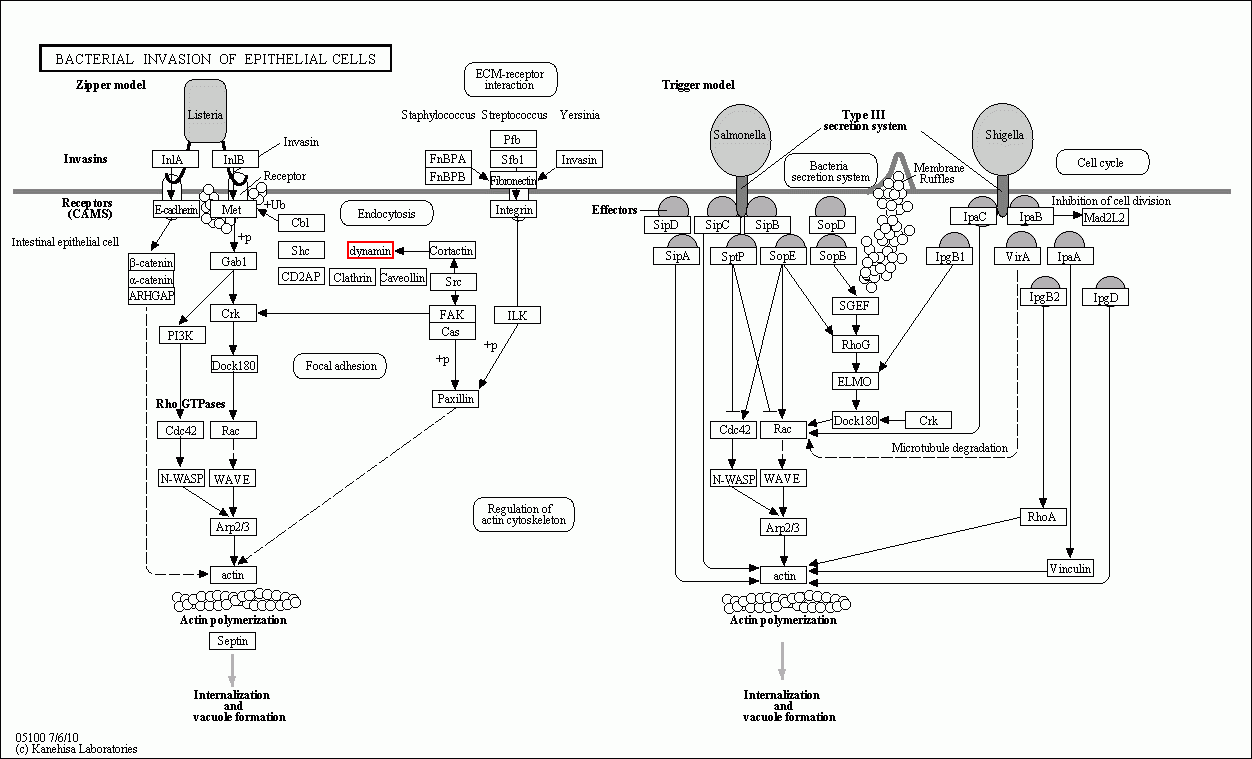

Supplement: S1 File — (ZIP) [file pone.0143219.s003.zip › pathway map/132 map05100.png]

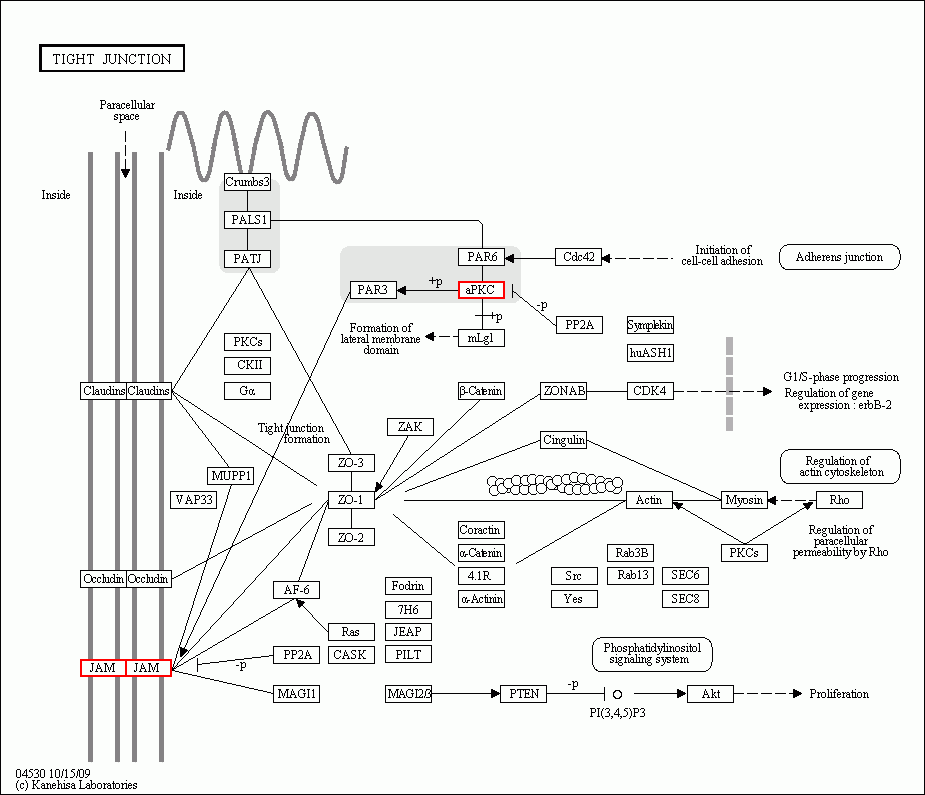

Supplement: S1 File — (ZIP) [file pone.0143219.s003.zip › pathway map/133 map04530.png]

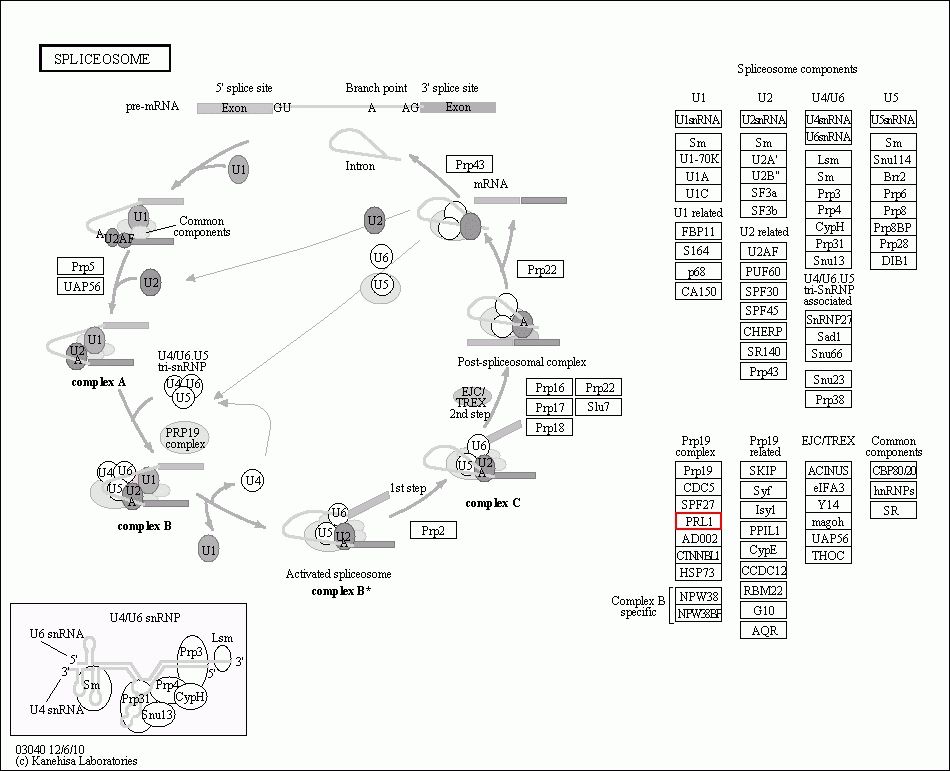

Supplement: S1 File — (ZIP) [file pone.0143219.s003.zip › pathway map/134 map03040.png]

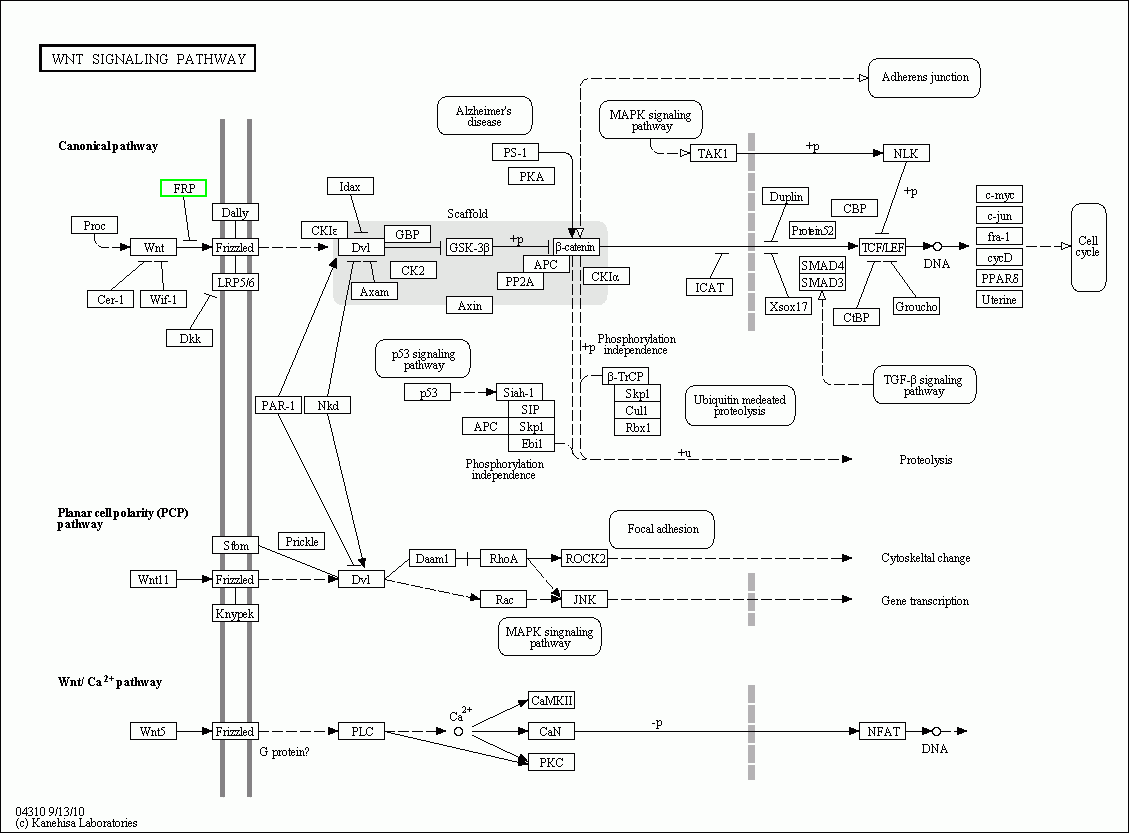

Supplement: S1 File — (ZIP) [file pone.0143219.s003.zip › pathway map/135 map04310.png]

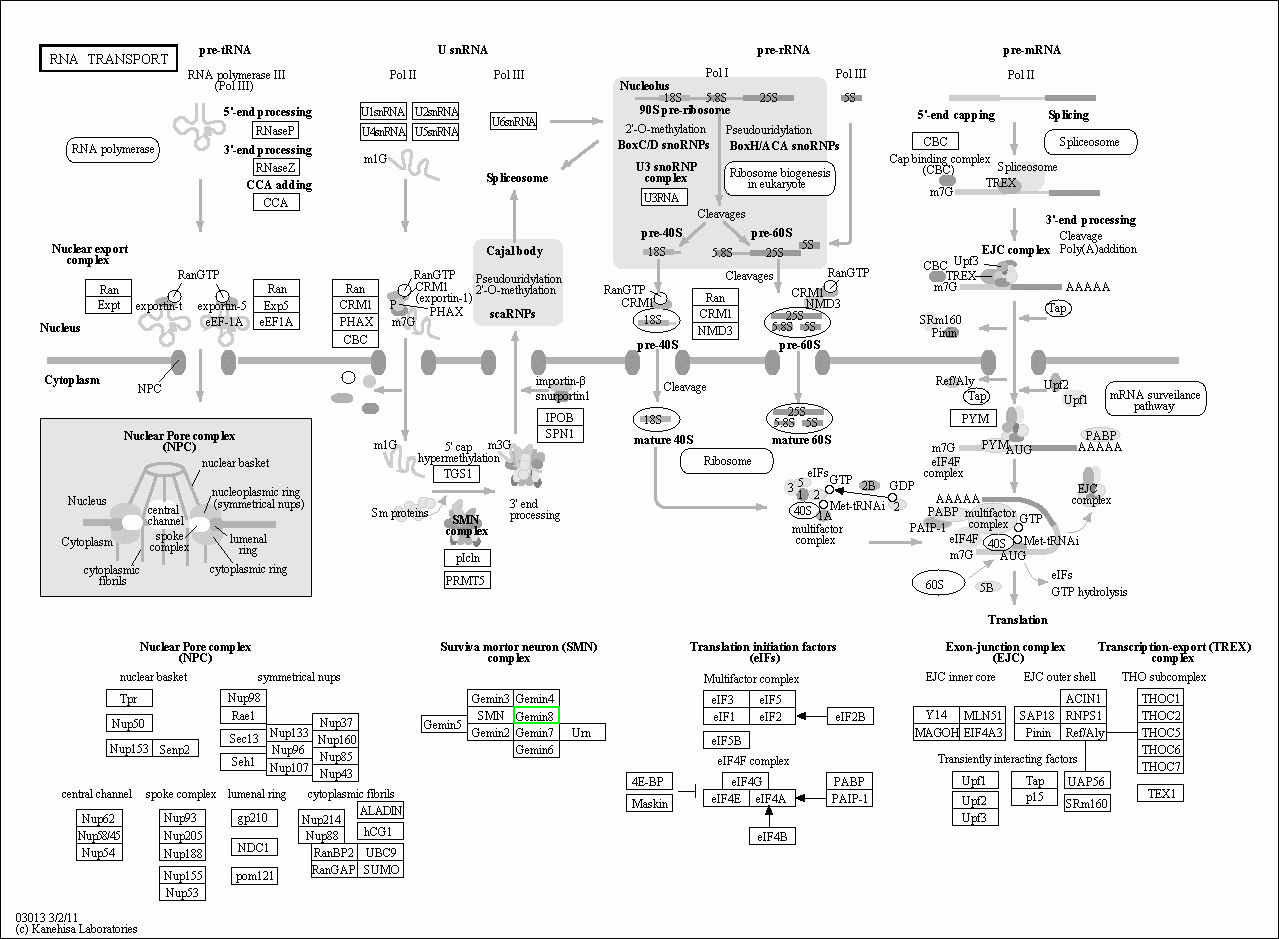

Supplement: S1 File — (ZIP) [file pone.0143219.s003.zip › pathway map/136 map03013.png]

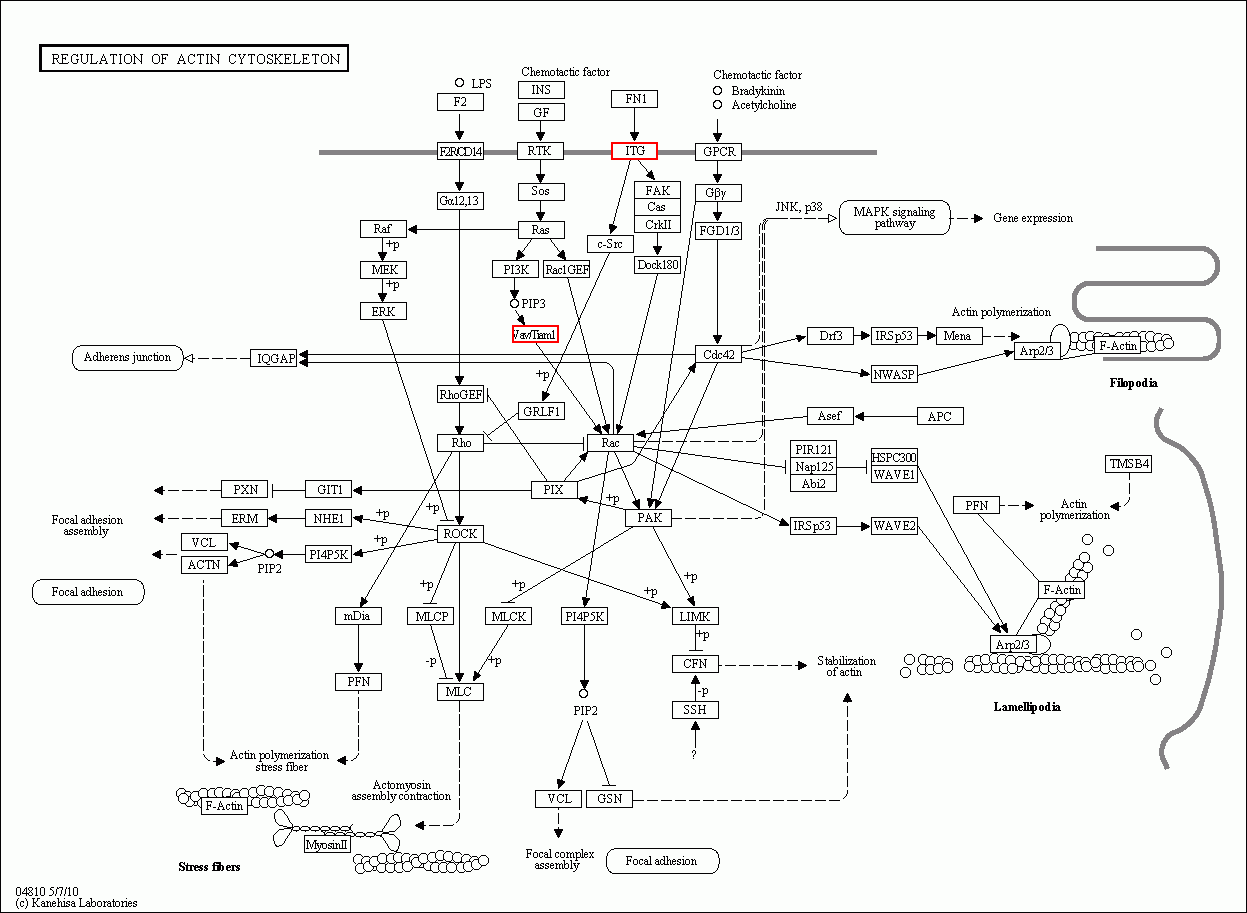

Supplement: S1 File — (ZIP) [file pone.0143219.s003.zip › pathway map/137 map04810.png]

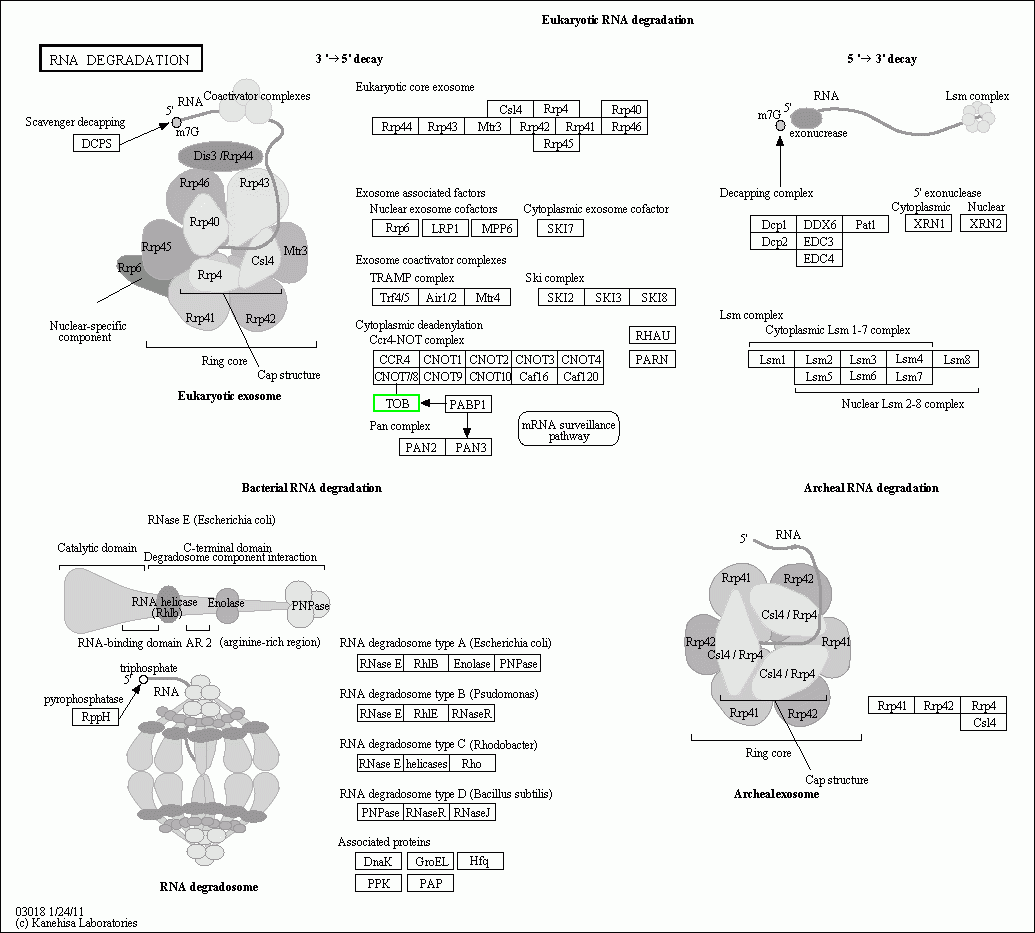

Supplement: S1 File — (ZIP) [file pone.0143219.s003.zip › pathway map/138 map03018.png]

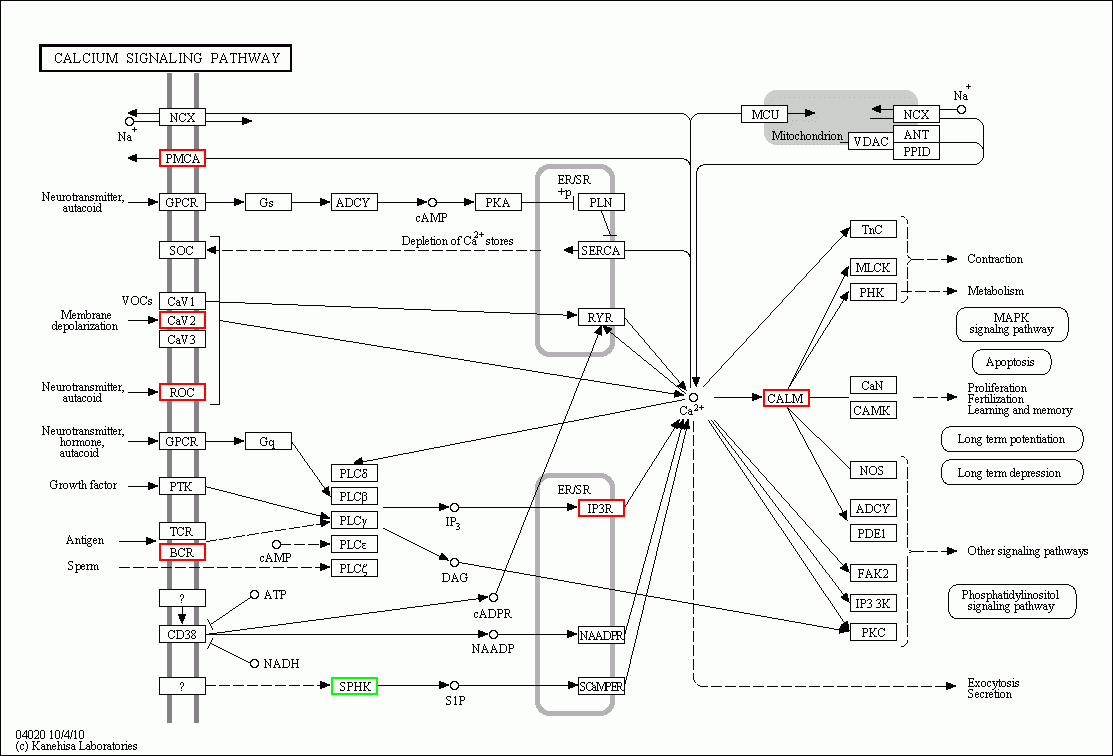

Supplement: S1 File — (ZIP) [file pone.0143219.s003.zip › pathway map/14 map04020.png]

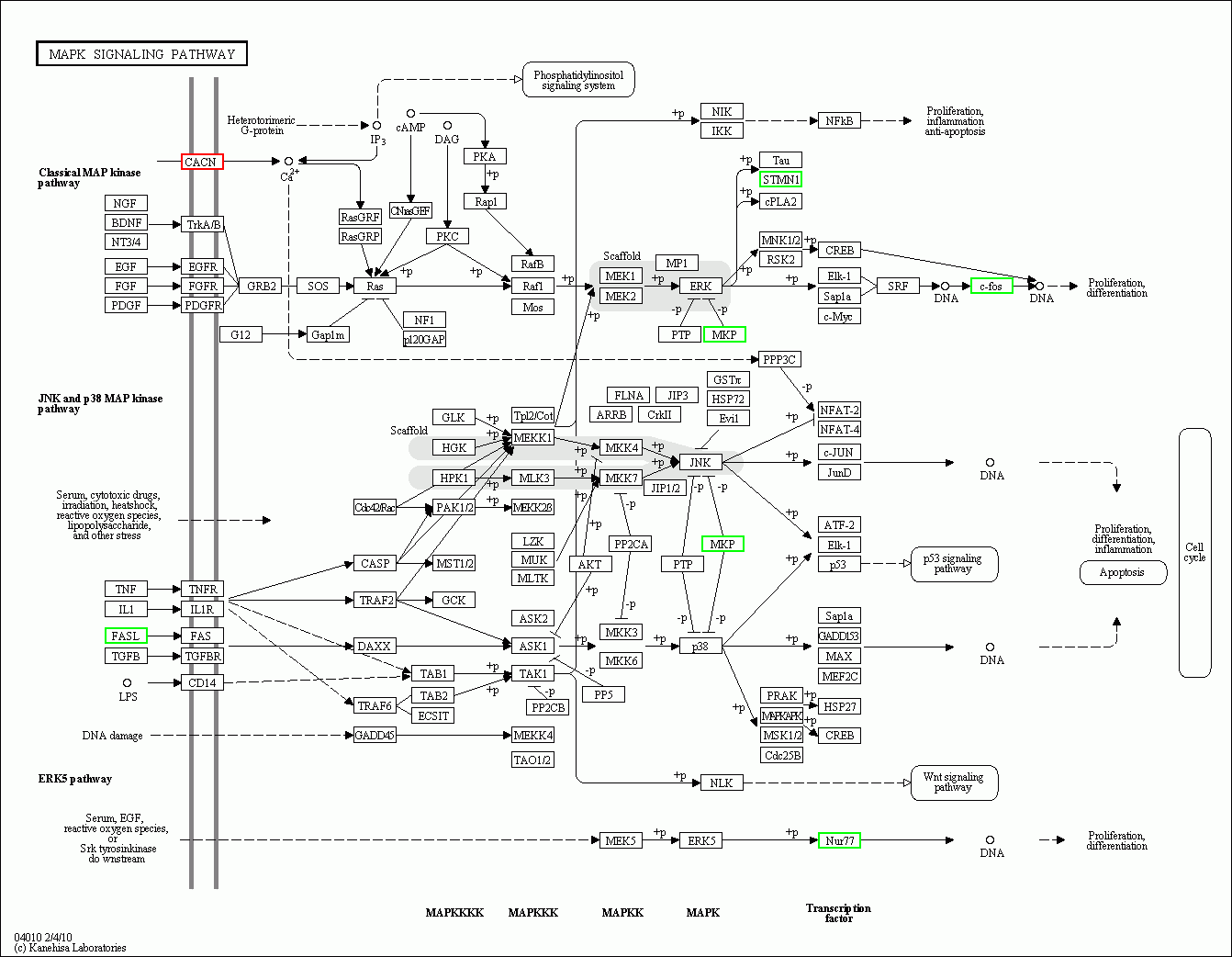

Supplement: S1 File — (ZIP) [file pone.0143219.s003.zip › pathway map/15 map04010.png]

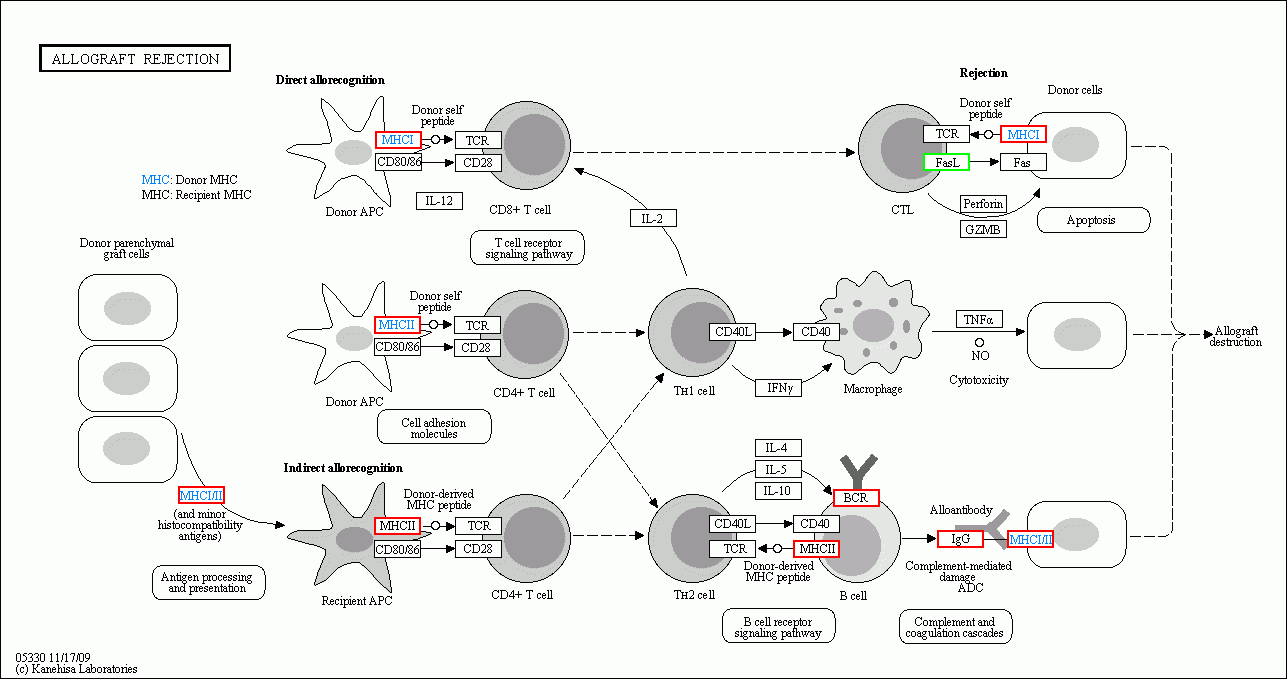

Supplement: S1 File — (ZIP) [file pone.0143219.s003.zip › pathway map/16 map05330.png]

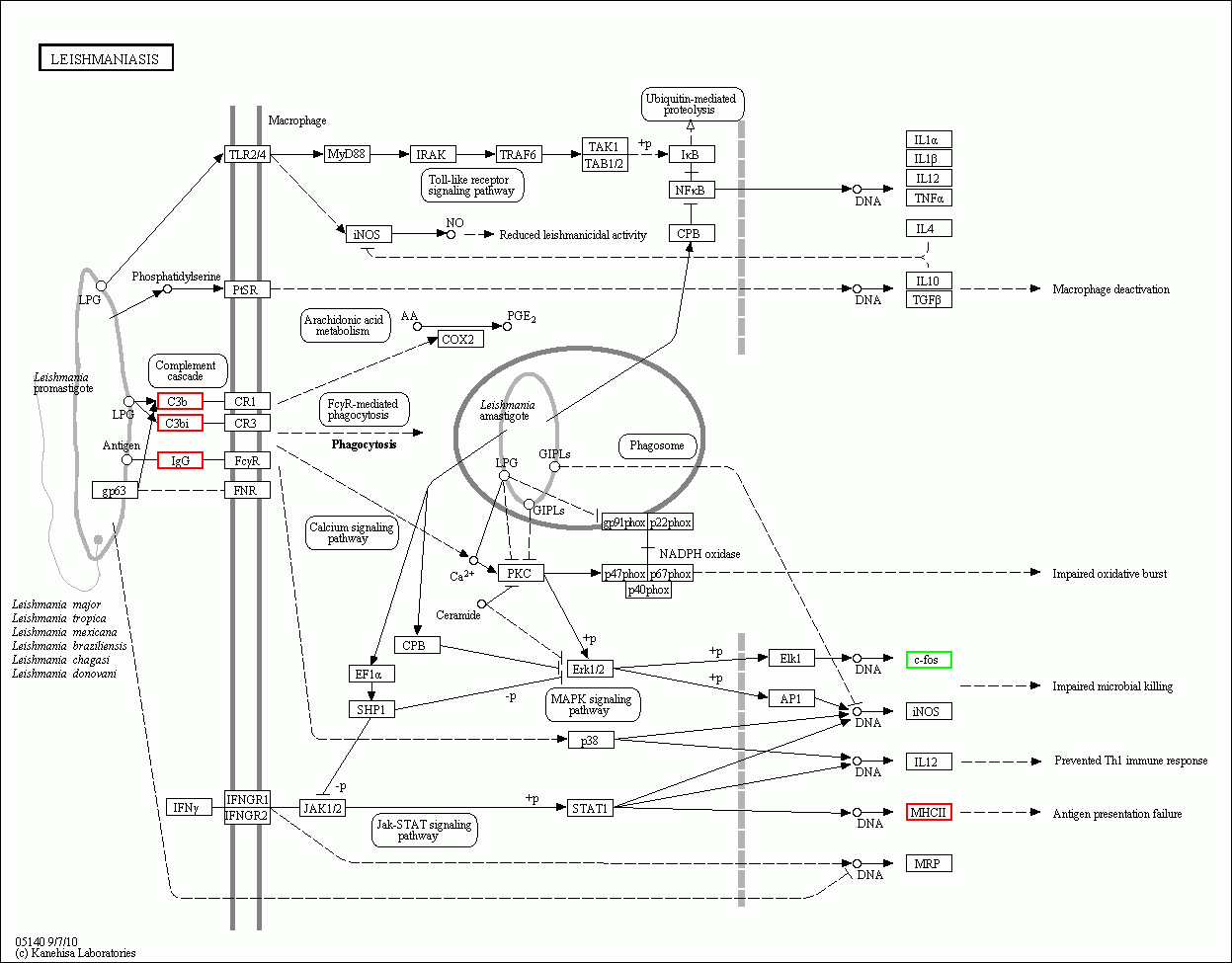

Supplement: S1 File — (ZIP) [file pone.0143219.s003.zip › pathway map/17 map05140.png]

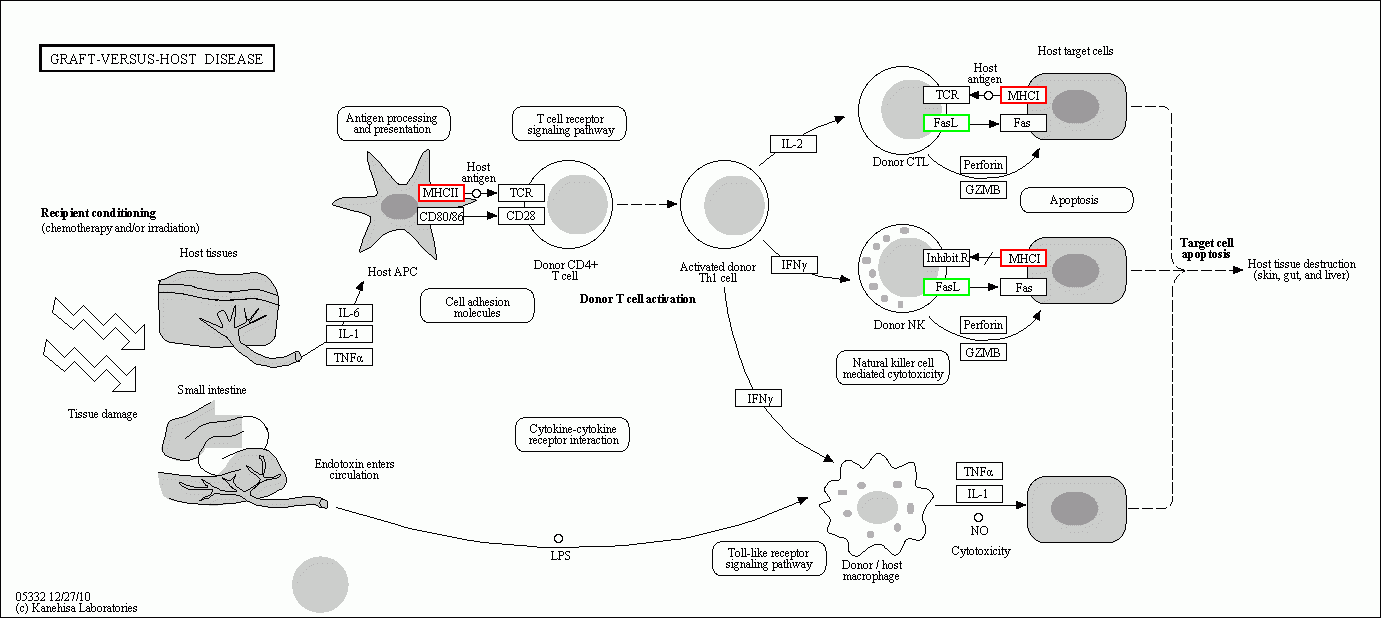

Supplement: S1 File — (ZIP) [file pone.0143219.s003.zip › pathway map/18 map05332.png]

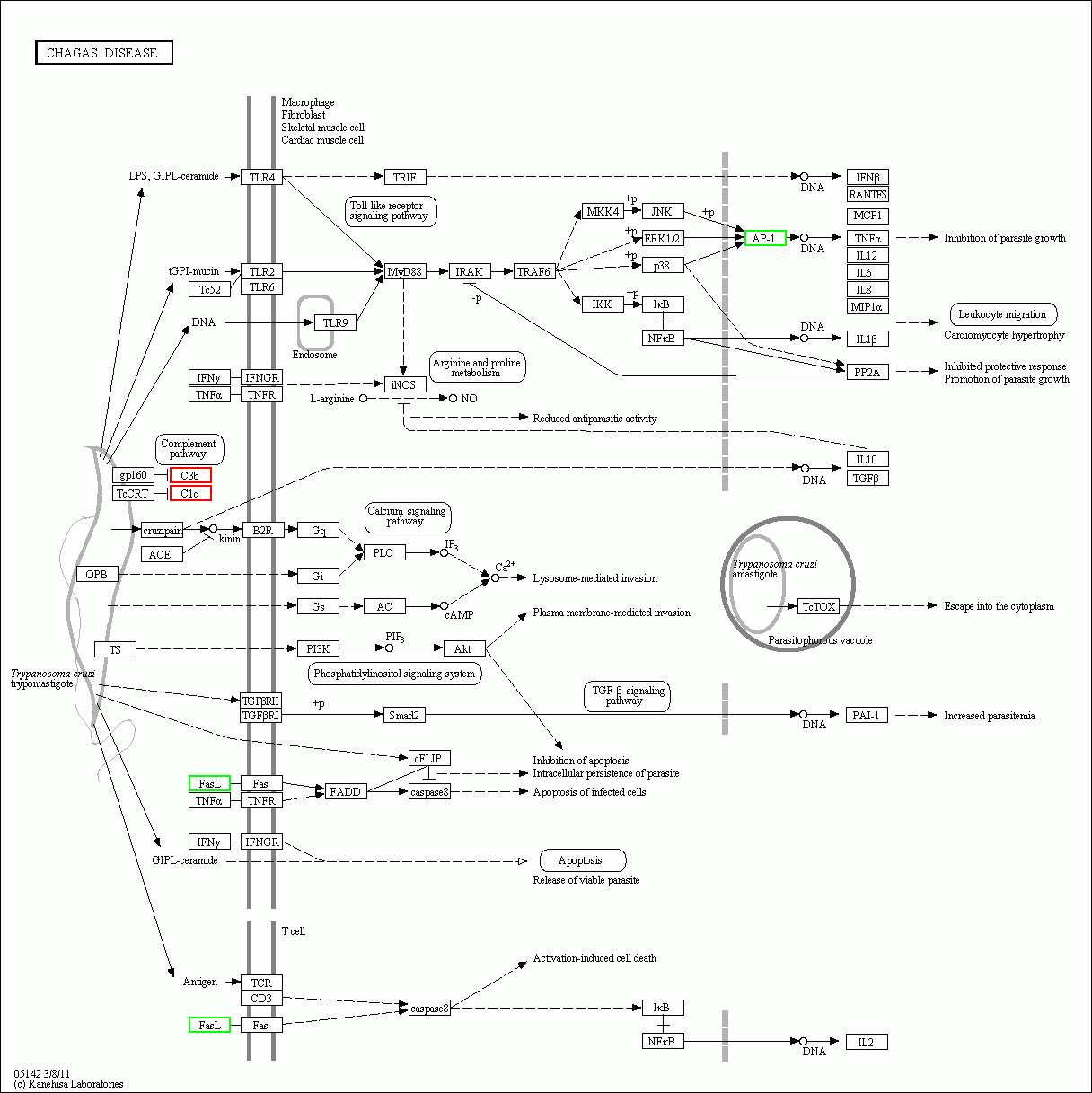

Supplement: S1 File — (ZIP) [file pone.0143219.s003.zip › pathway map/19 map05142.png]

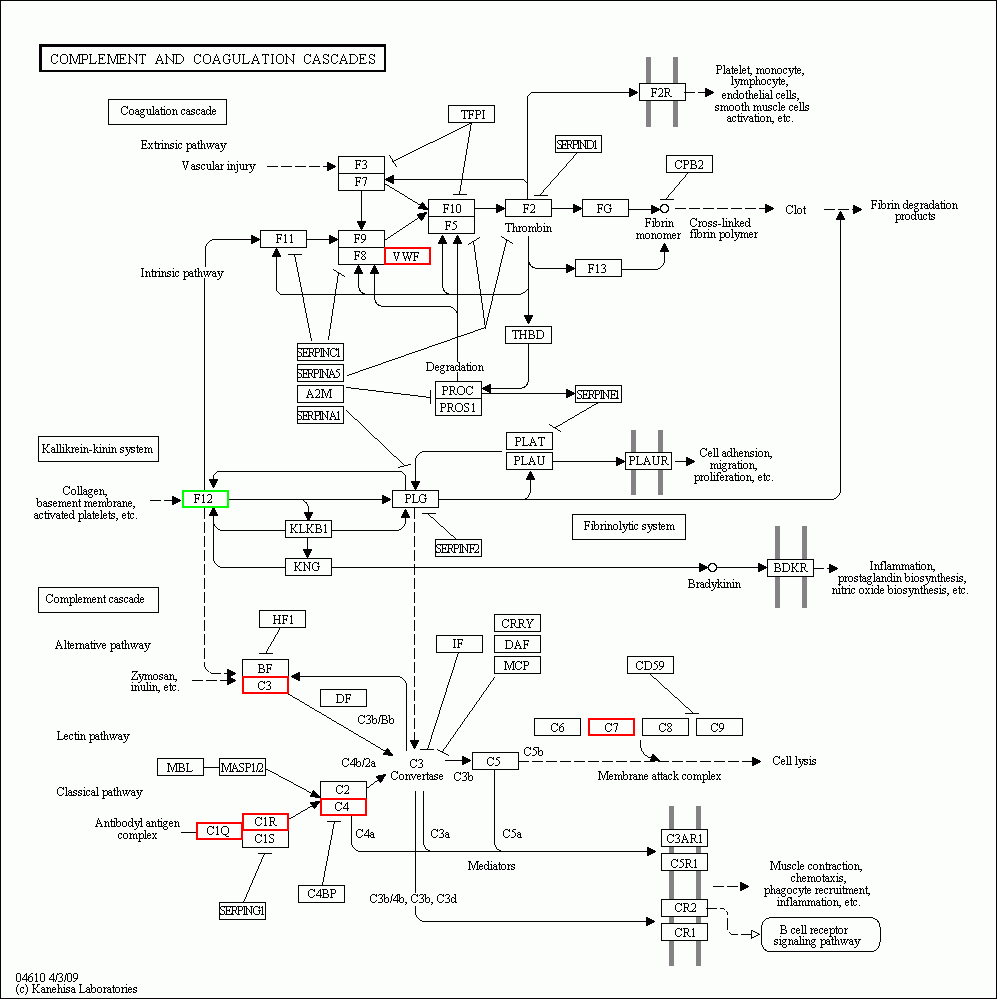

Supplement: S1 File — (ZIP) [file pone.0143219.s003.zip › pathway map/2 map04610.png]

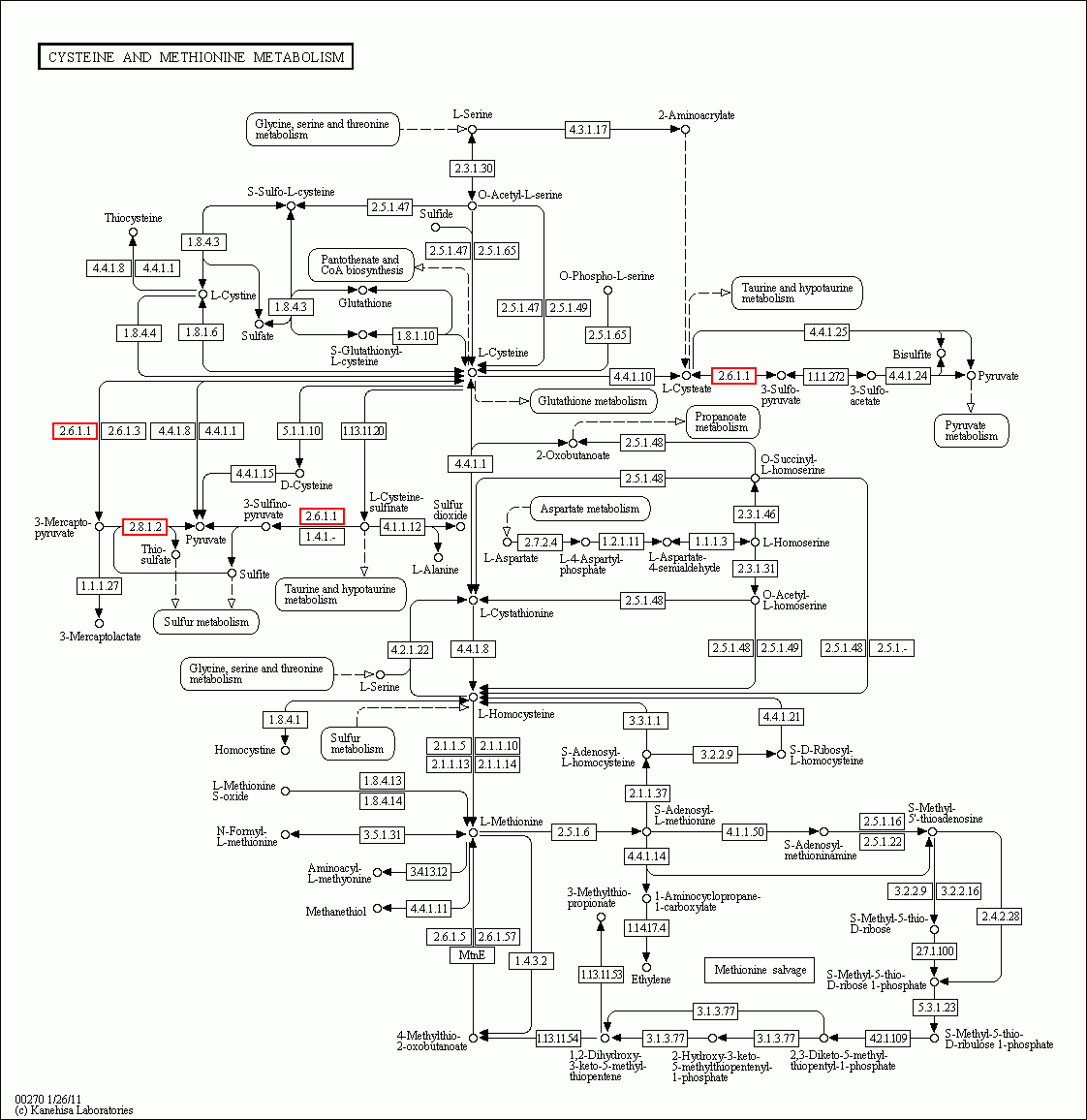

Supplement: S1 File — (ZIP) [file pone.0143219.s003.zip › pathway map/20 map00270.png]

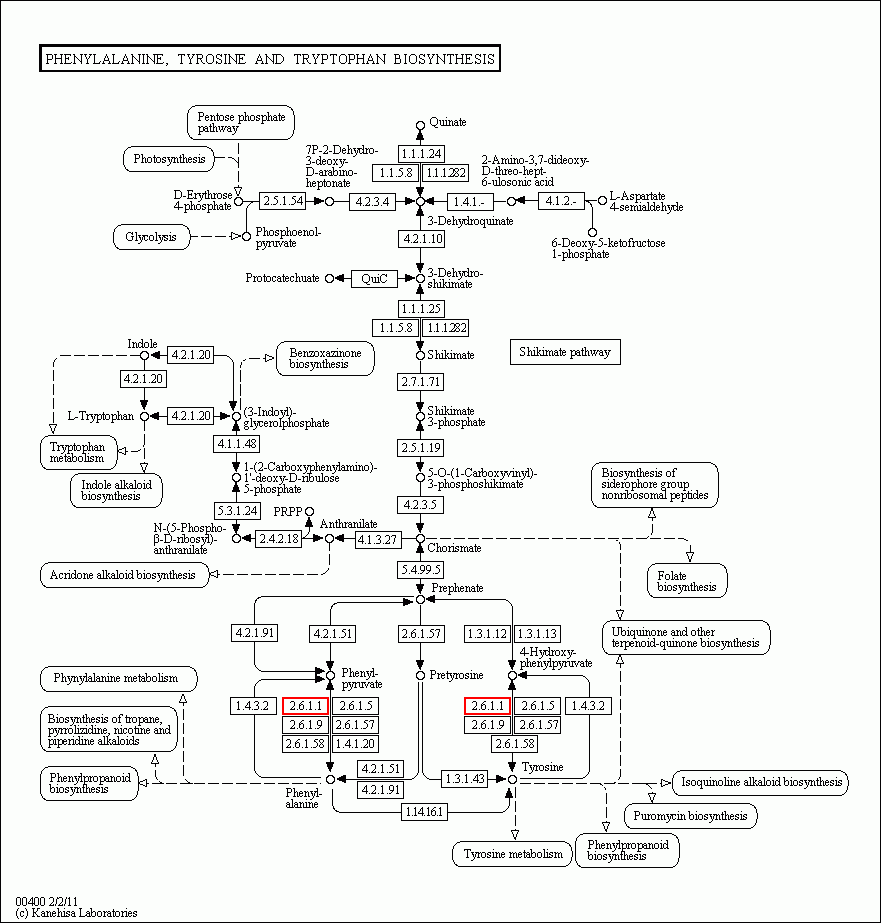

Supplement: S1 File — (ZIP) [file pone.0143219.s003.zip › pathway map/21 map00400.png]

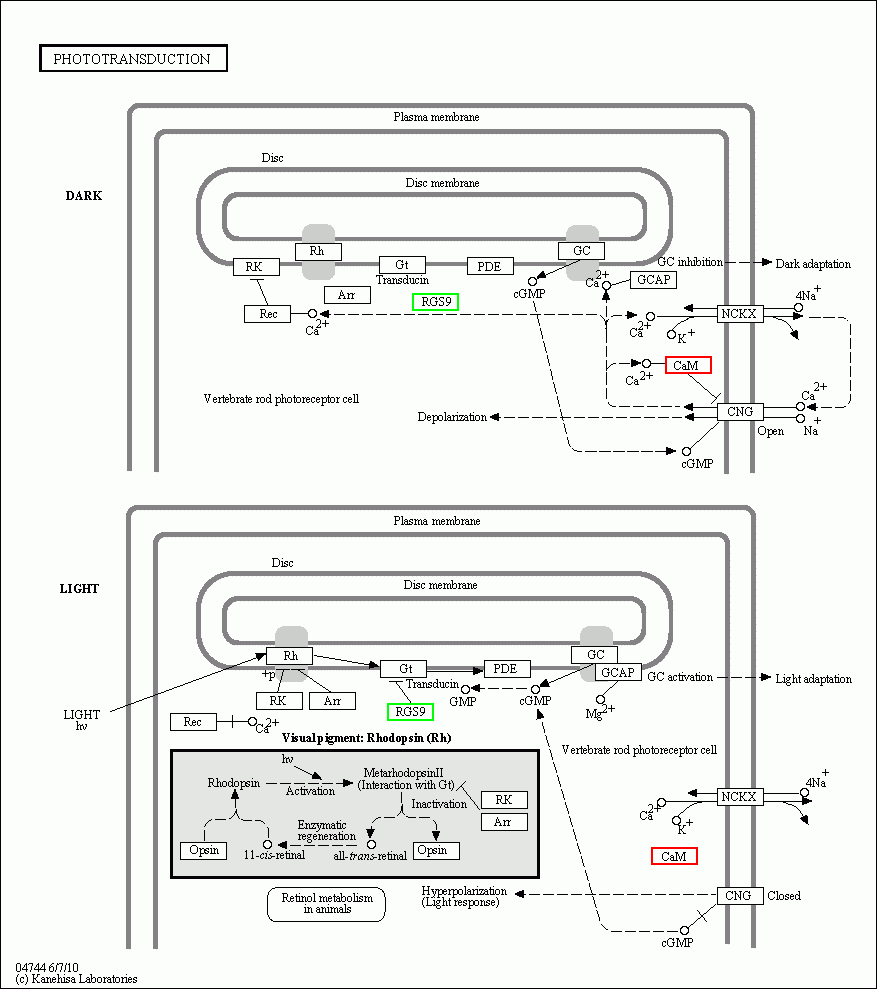

Supplement: S1 File — (ZIP) [file pone.0143219.s003.zip › pathway map/22 map04744.png]

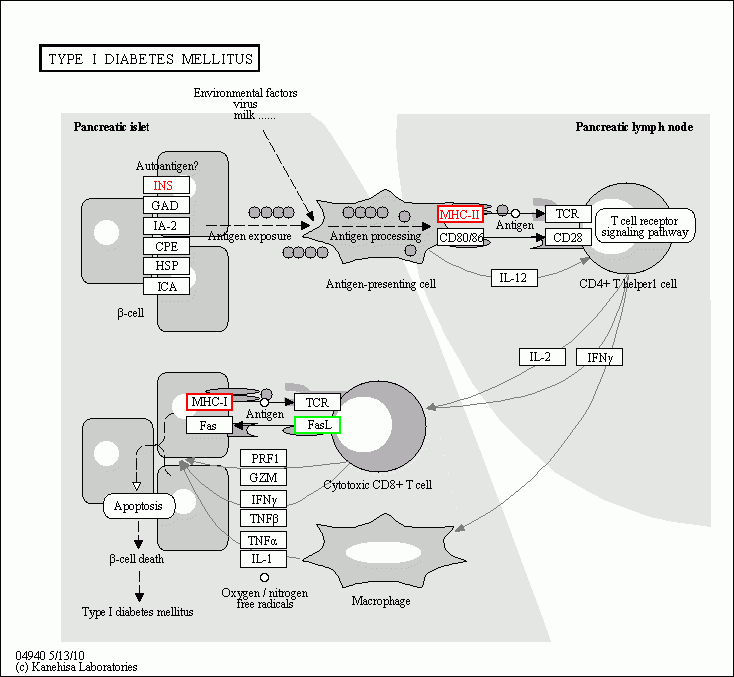

Supplement: S1 File — (ZIP) [file pone.0143219.s003.zip › pathway map/23 map04940.png]

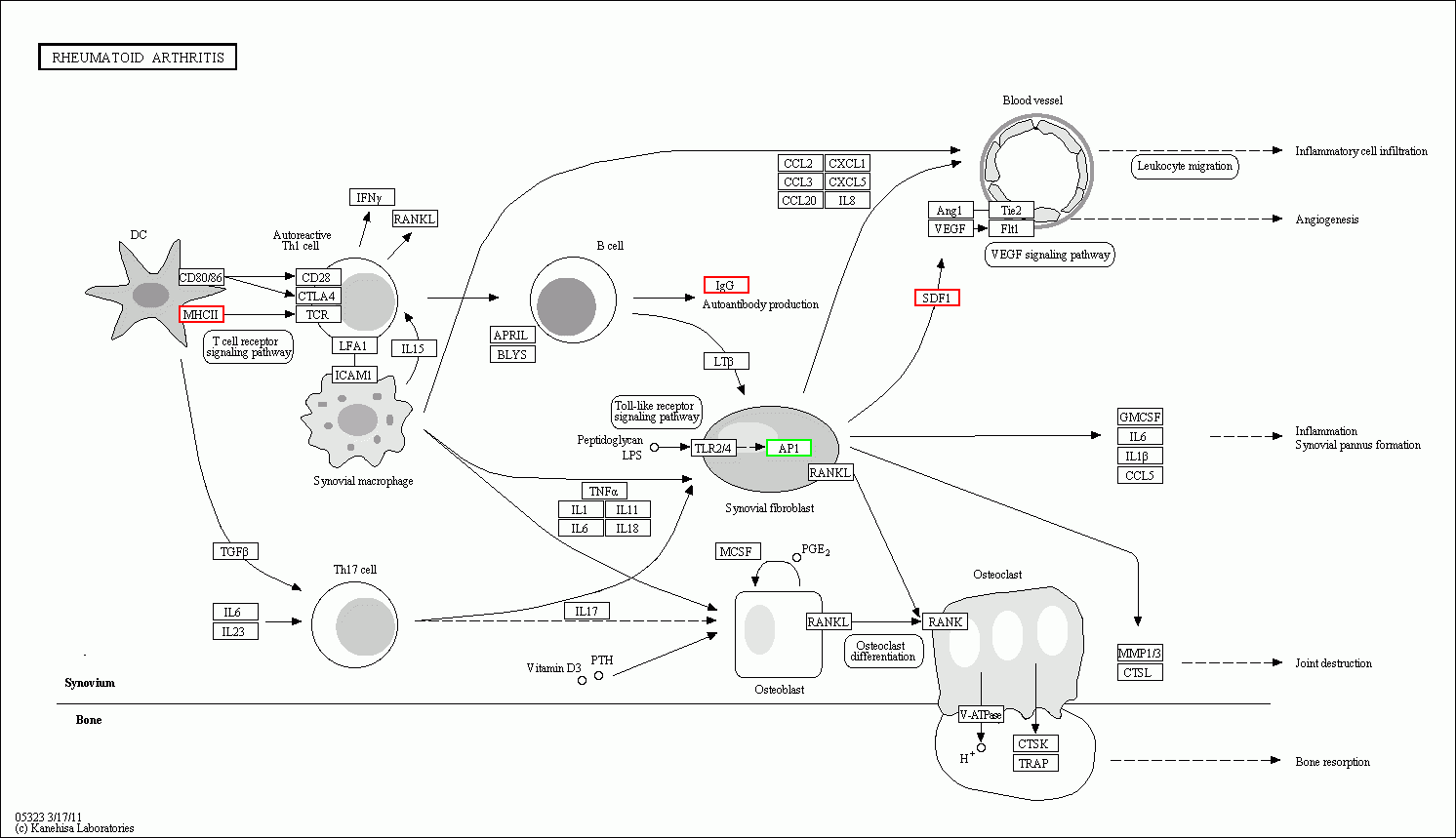

Supplement: S1 File — (ZIP) [file pone.0143219.s003.zip › pathway map/24 map05323.png]

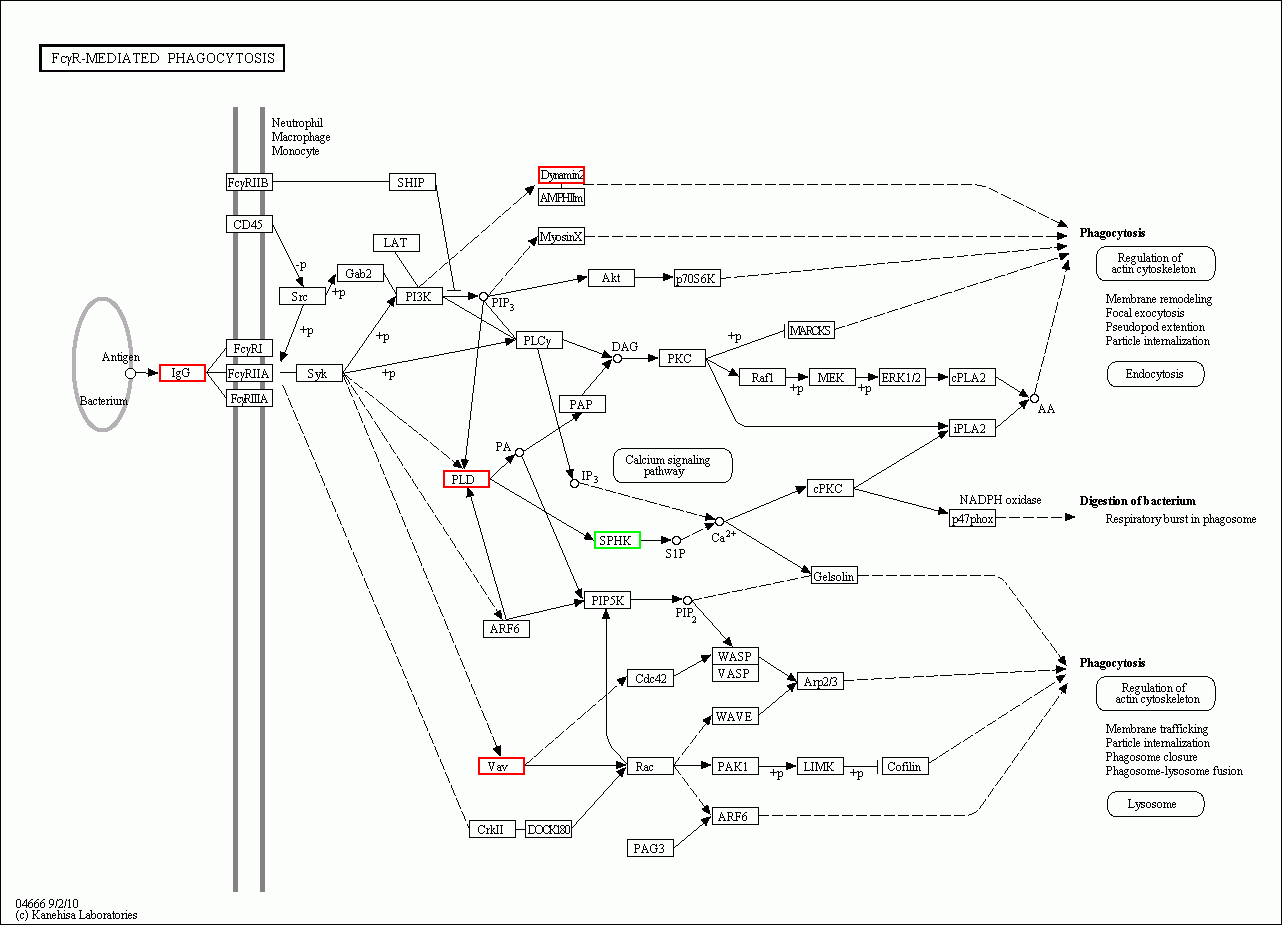

Supplement: S1 File — (ZIP) [file pone.0143219.s003.zip › pathway map/25 map04666.png]

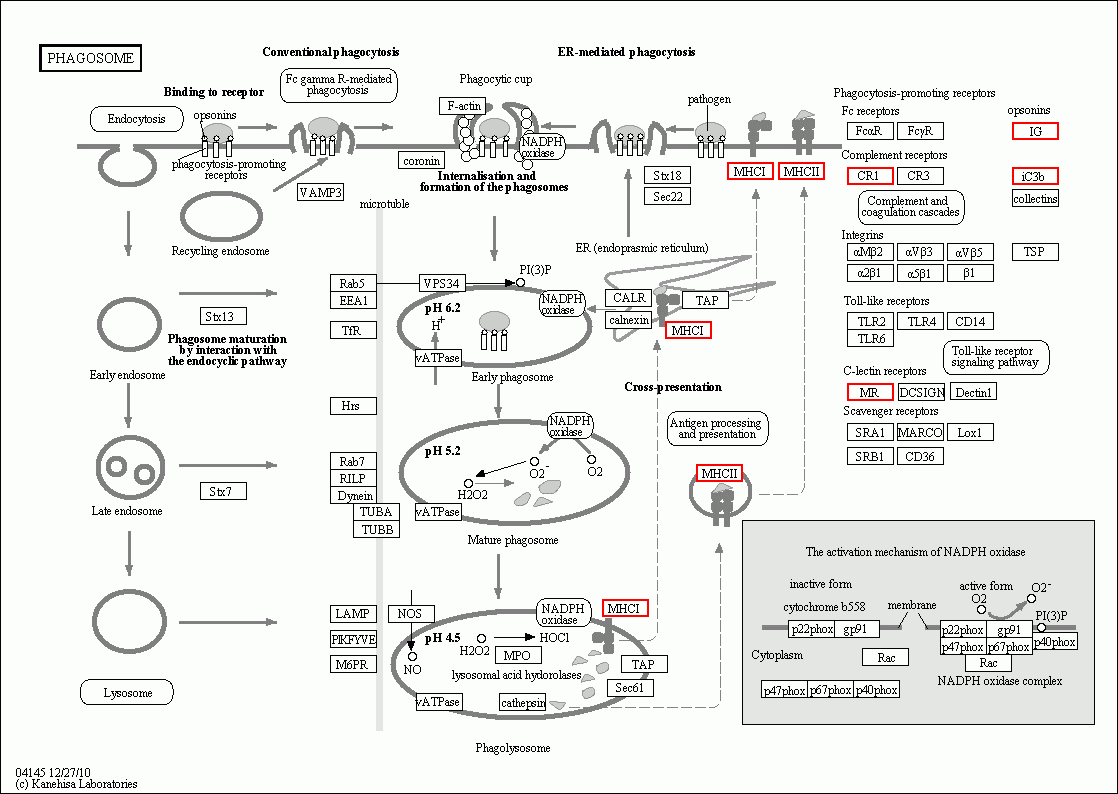

Supplement: S1 File — (ZIP) [file pone.0143219.s003.zip › pathway map/26 map04145.png]

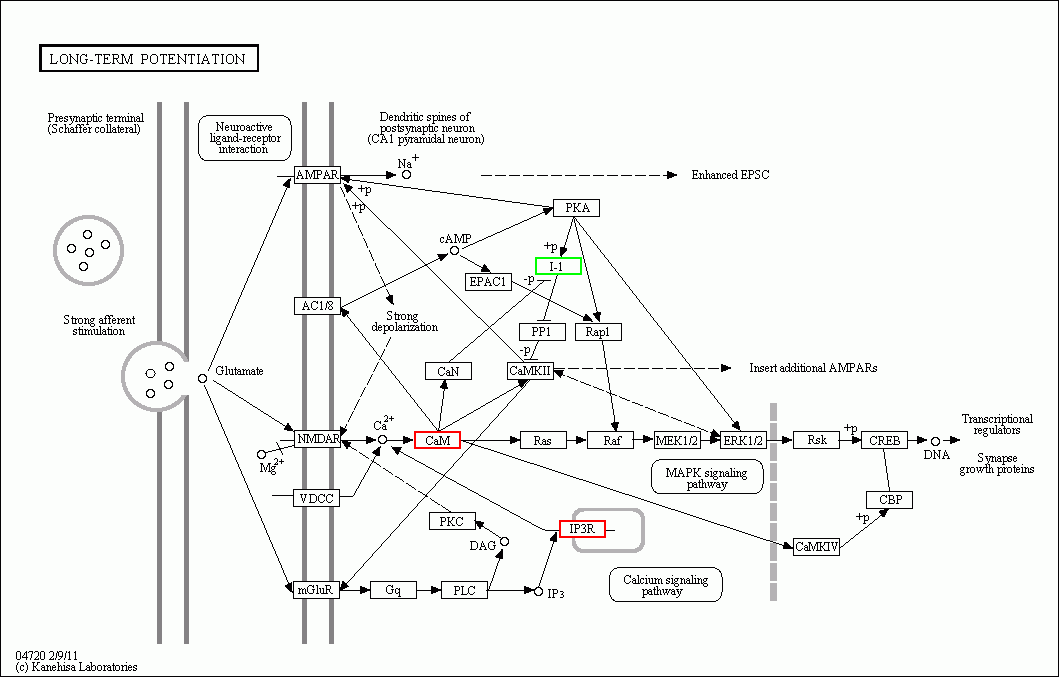

Supplement: S1 File — (ZIP) [file pone.0143219.s003.zip › pathway map/27 map04720.png]

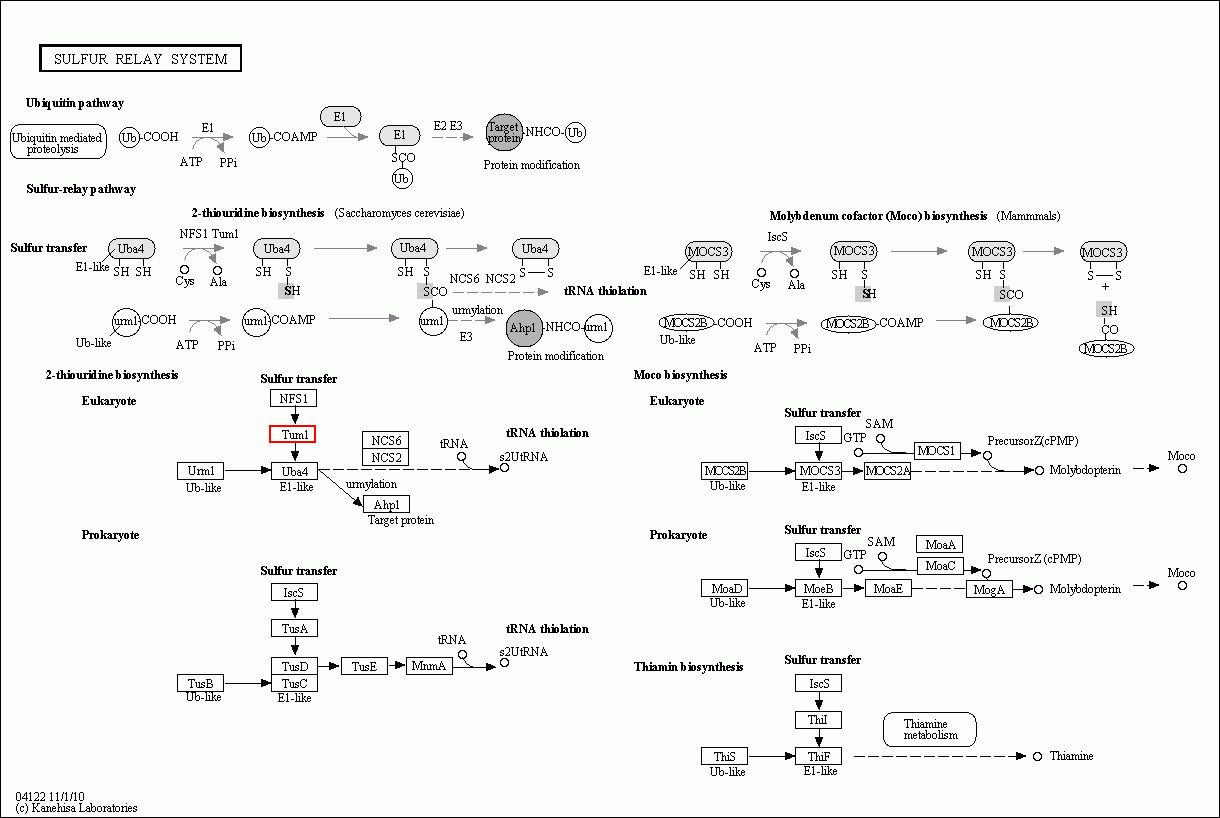

Supplement: S1 File — (ZIP) [file pone.0143219.s003.zip › pathway map/28 map04122.png]

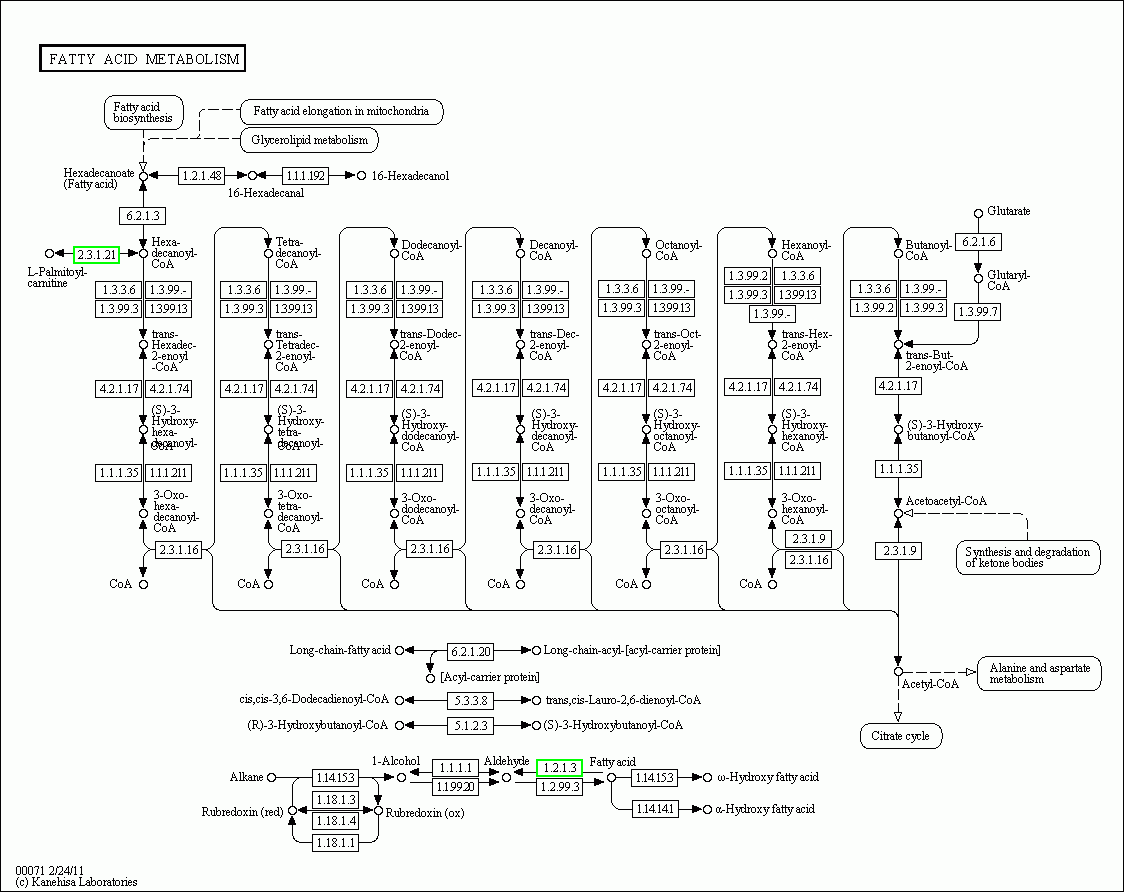

Supplement: S1 File — (ZIP) [file pone.0143219.s003.zip › pathway map/29 map00071.png]

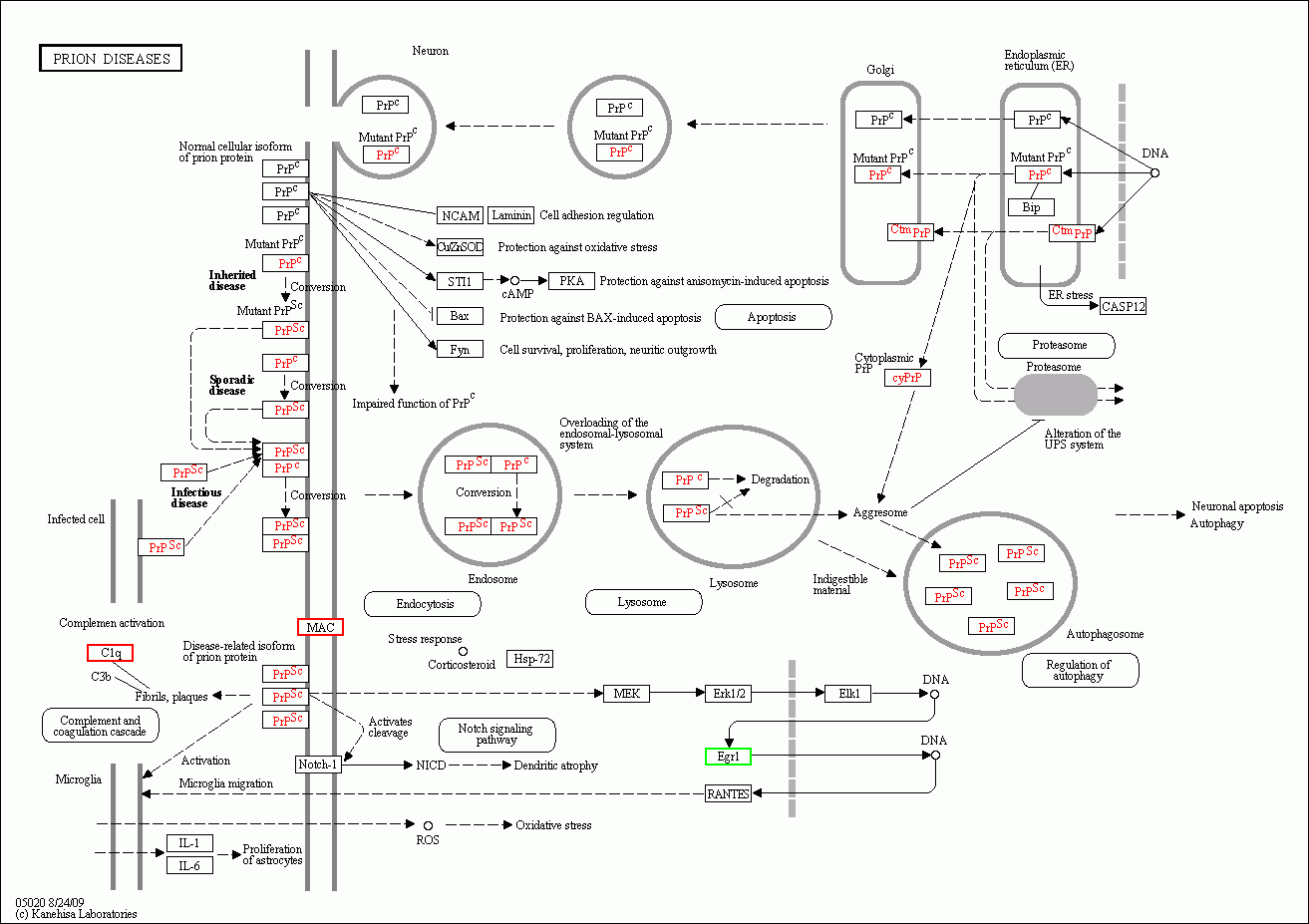

Supplement: S1 File — (ZIP) [file pone.0143219.s003.zip › pathway map/3 map05020.png]

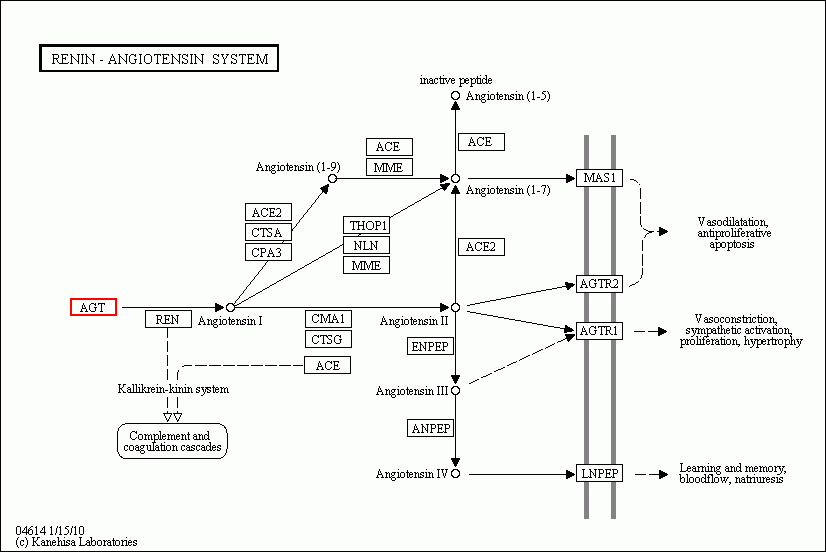

Supplement: S1 File — (ZIP) [file pone.0143219.s003.zip › pathway map/30 map04614.png]

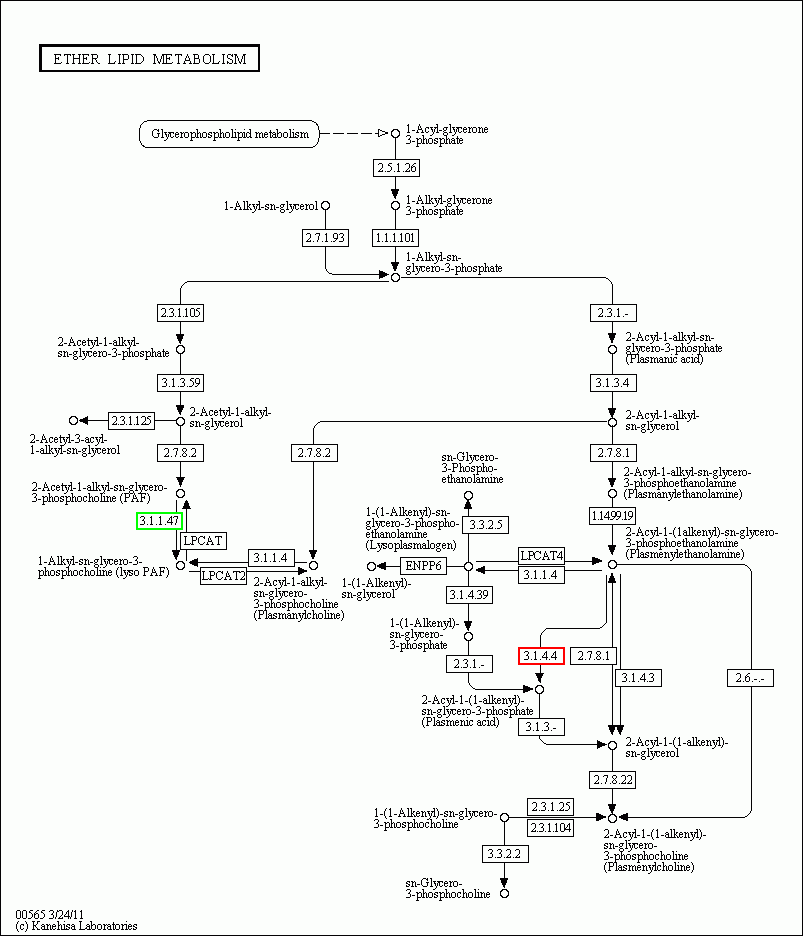

Supplement: S1 File — (ZIP) [file pone.0143219.s003.zip › pathway map/31 map00565.png]

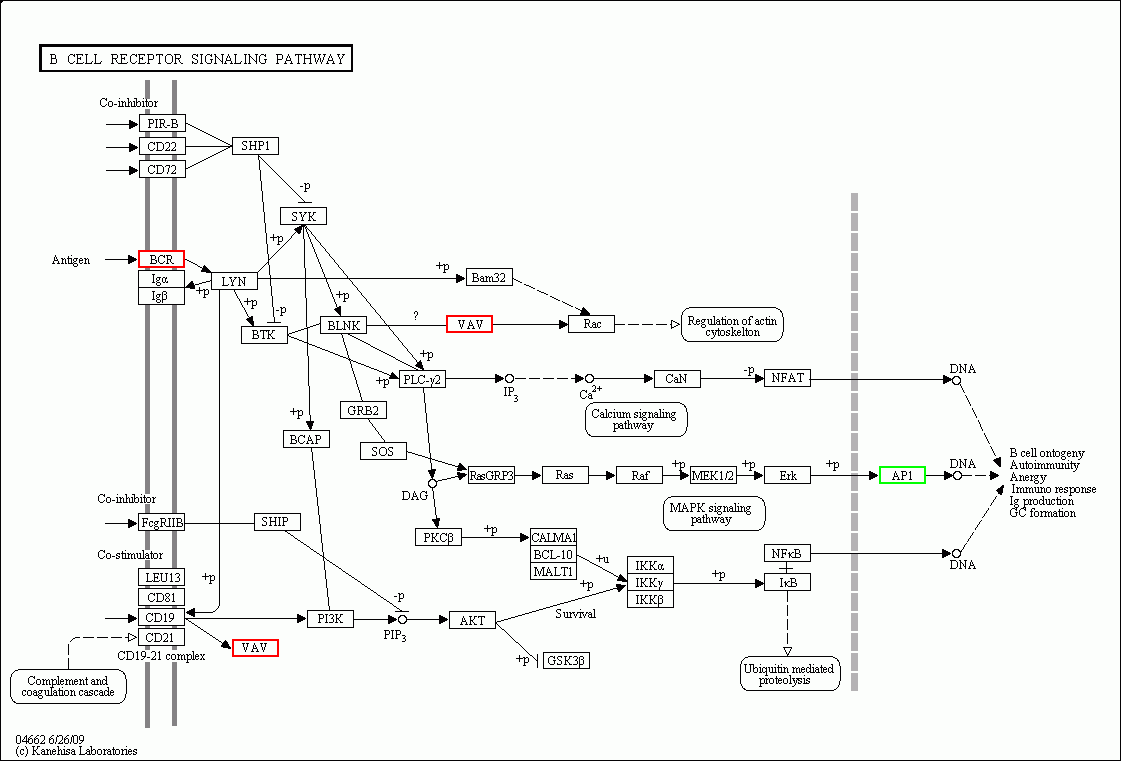

Supplement: S1 File — (ZIP) [file pone.0143219.s003.zip › pathway map/32 map04662.png]

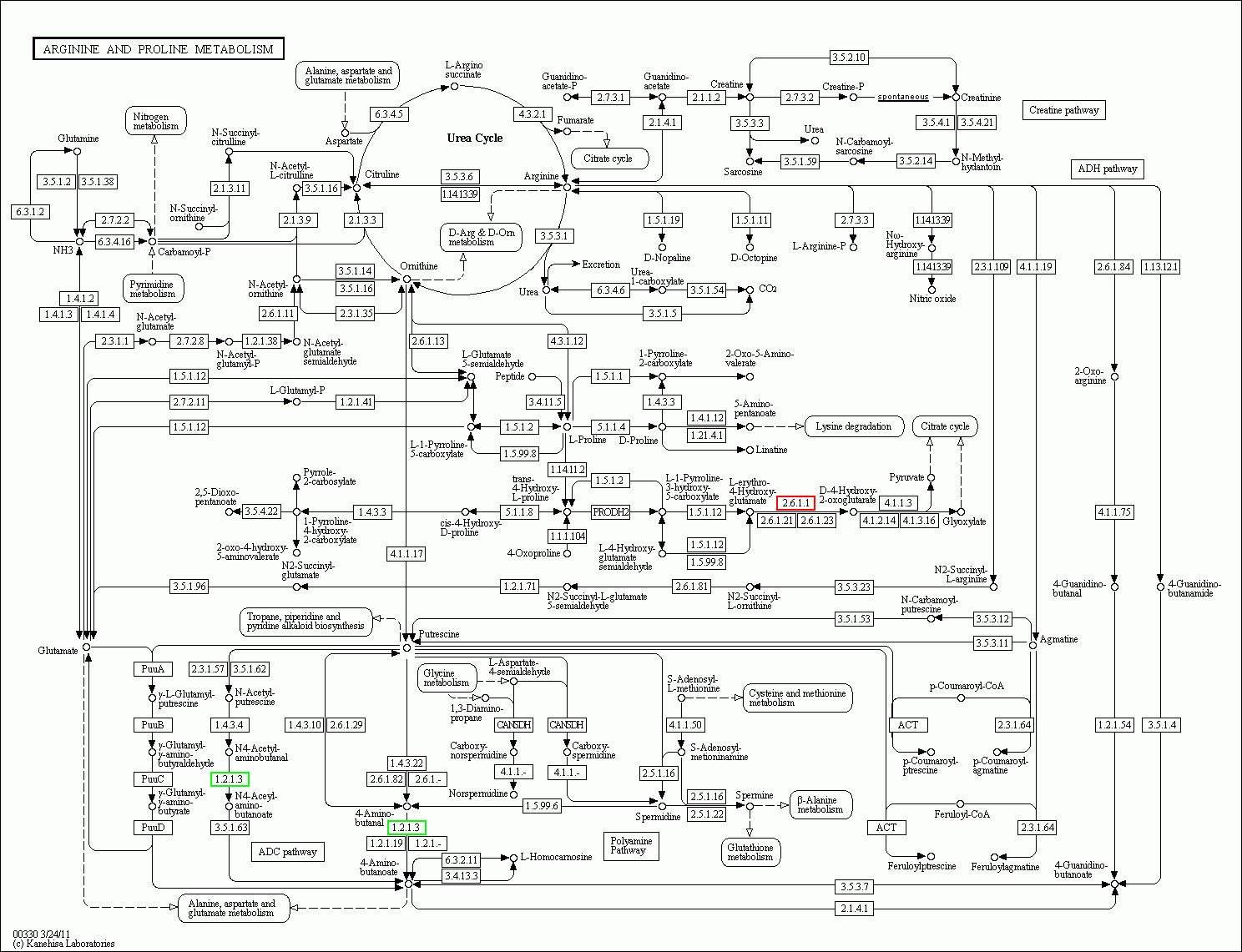

Supplement: S1 File — (ZIP) [file pone.0143219.s003.zip › pathway map/33 map00330.png]

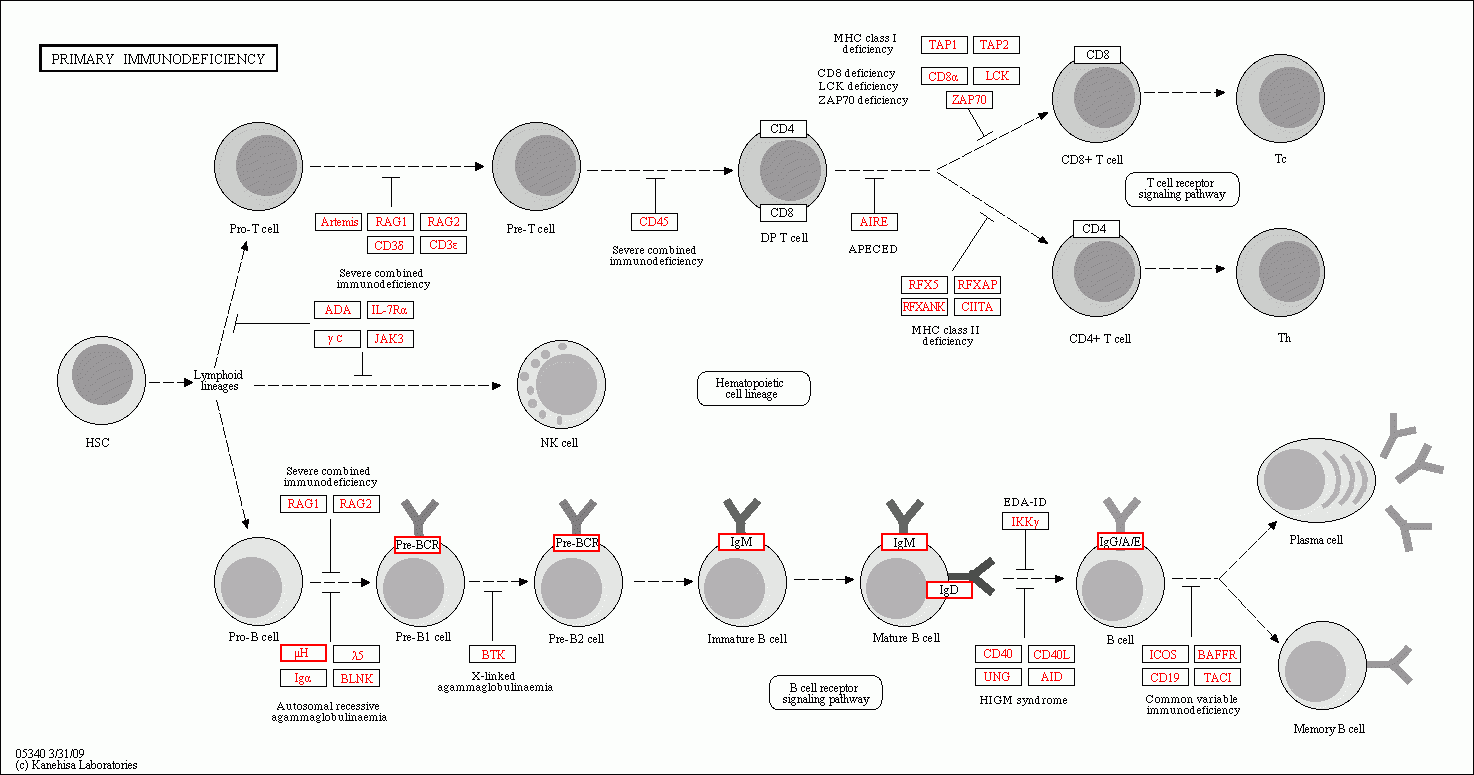

Supplement: S1 File — (ZIP) [file pone.0143219.s003.zip › pathway map/34 map05340.png]

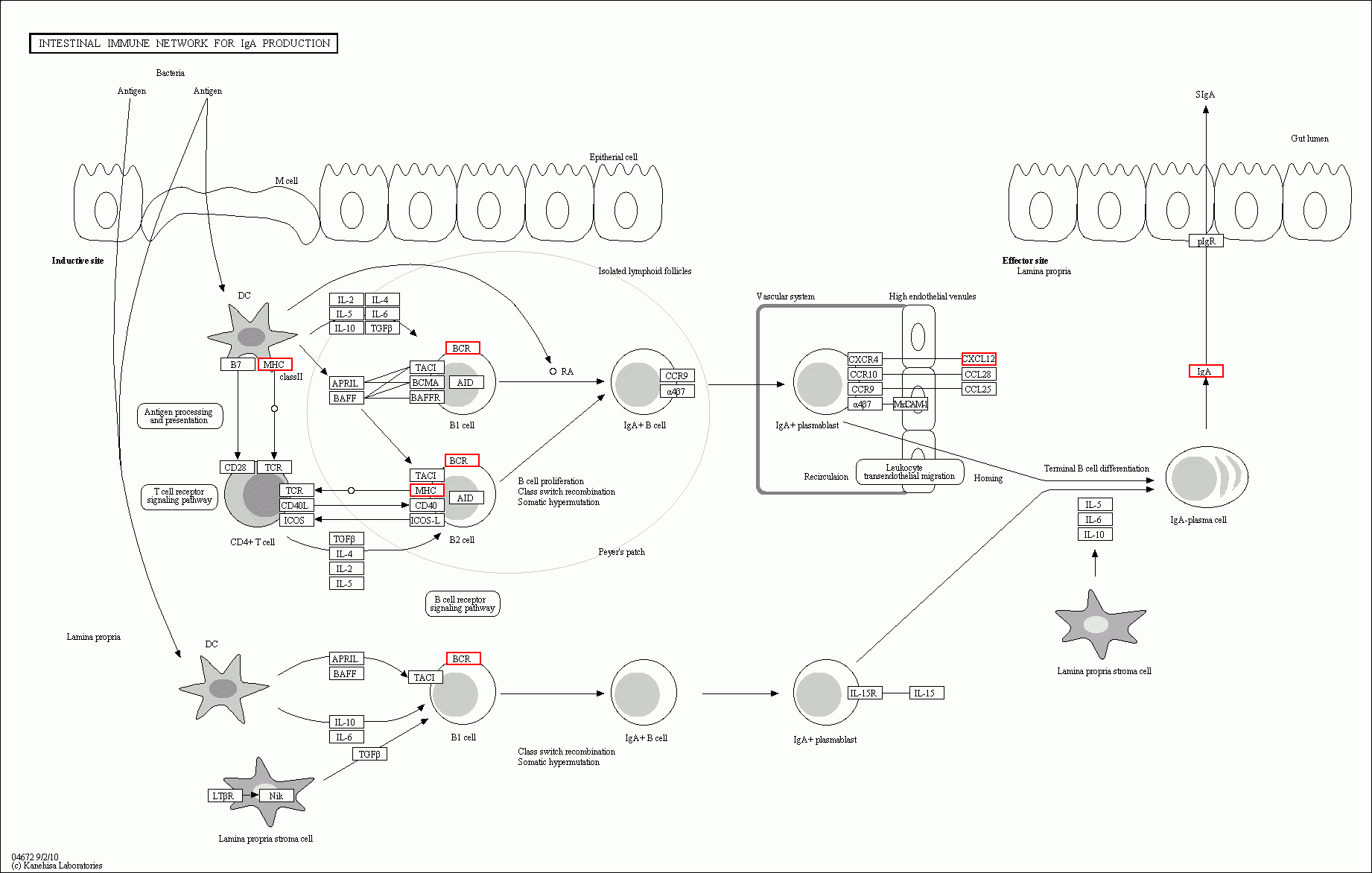

Supplement: S1 File — (ZIP) [file pone.0143219.s003.zip › pathway map/35 map04672.png]

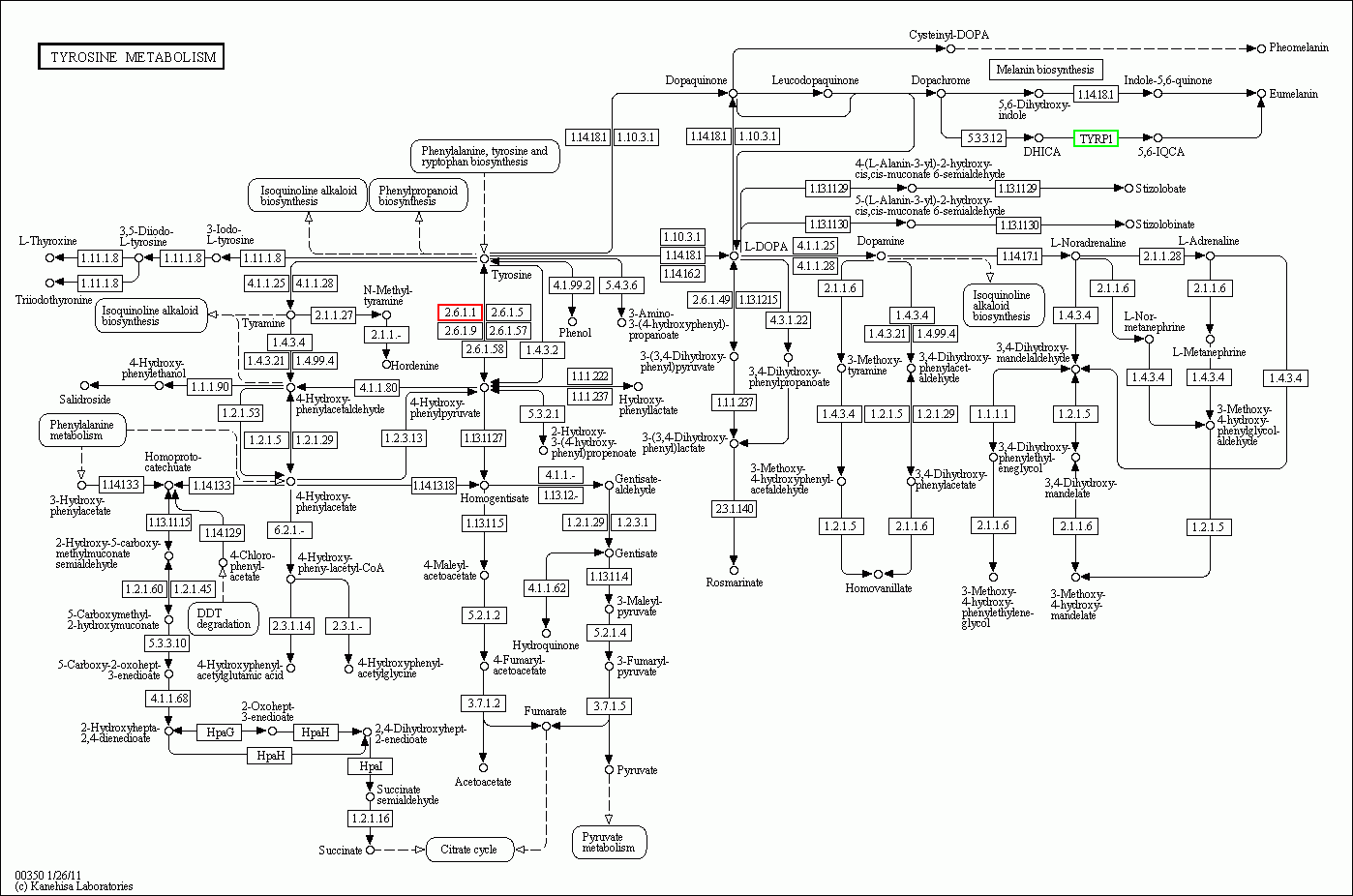

Supplement: S1 File — (ZIP) [file pone.0143219.s003.zip › pathway map/36 map00350.png]

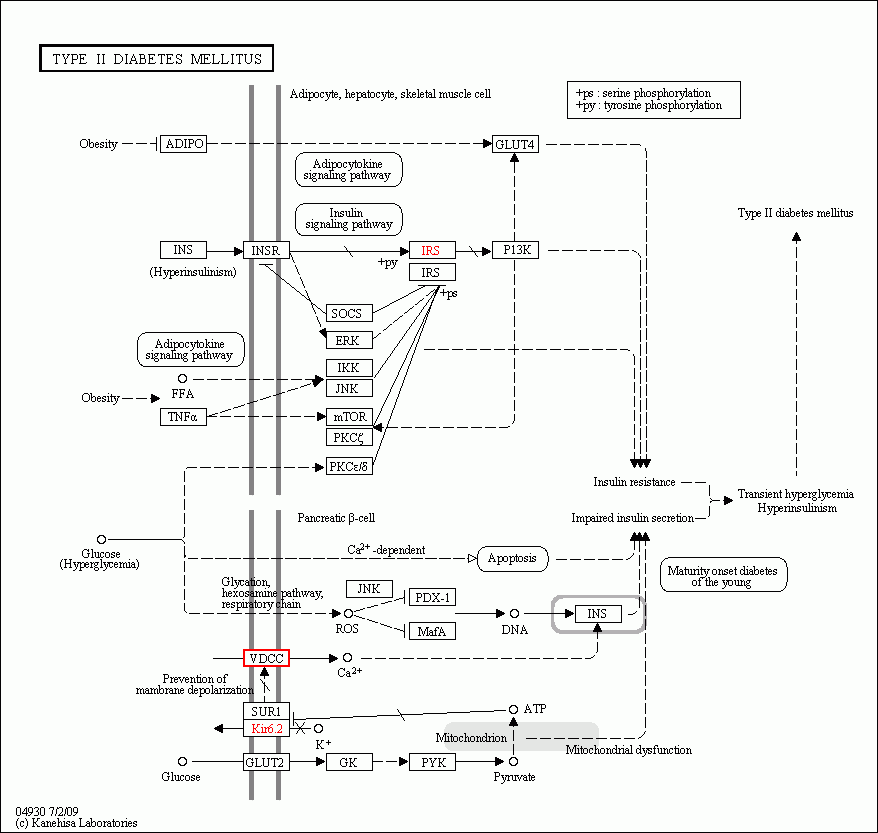

Supplement: S1 File — (ZIP) [file pone.0143219.s003.zip › pathway map/37 map04930.png]

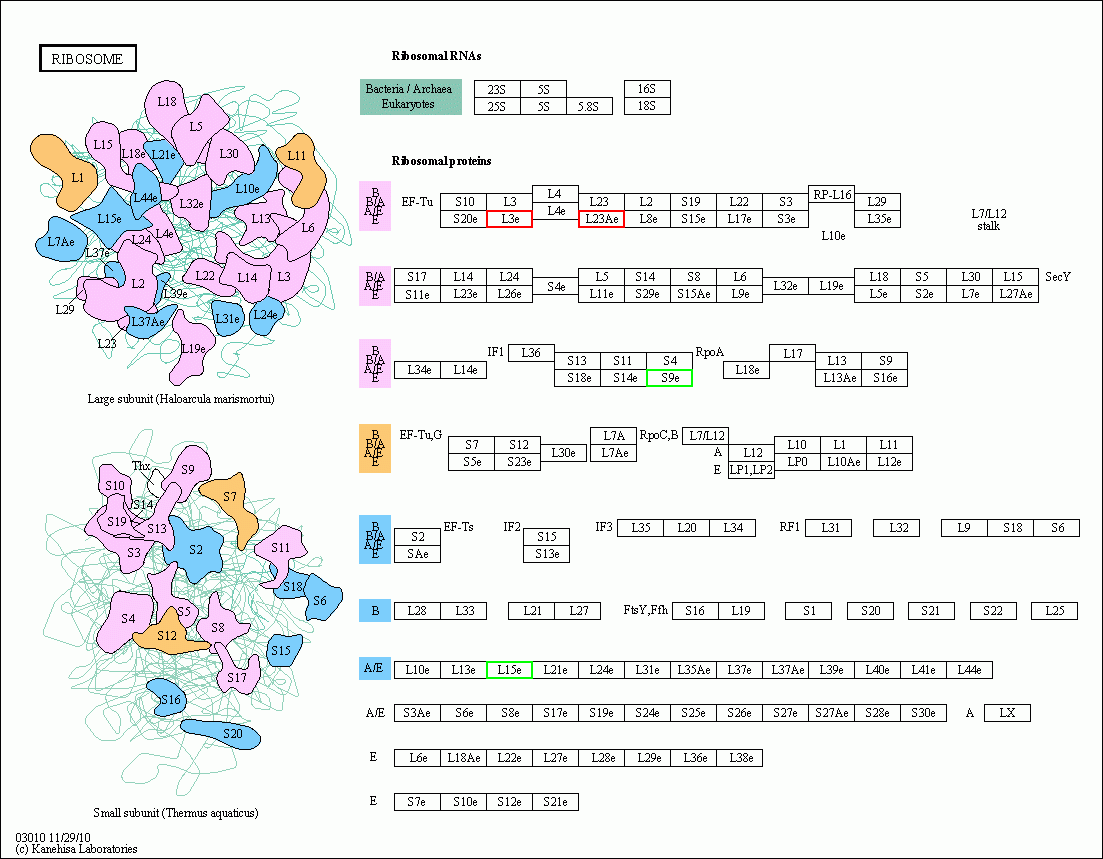

Supplement: S1 File — (ZIP) [file pone.0143219.s003.zip › pathway map/38 map03010.png]

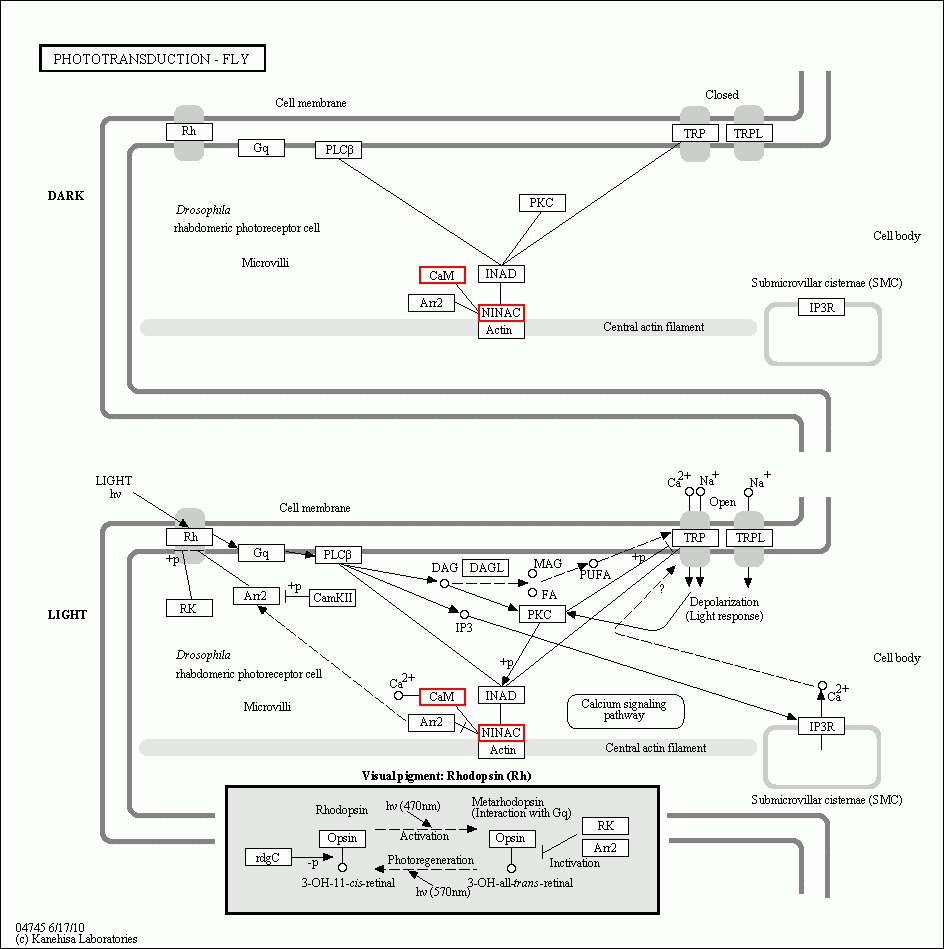

Supplement: S1 File — (ZIP) [file pone.0143219.s003.zip › pathway map/39 map04745.png]

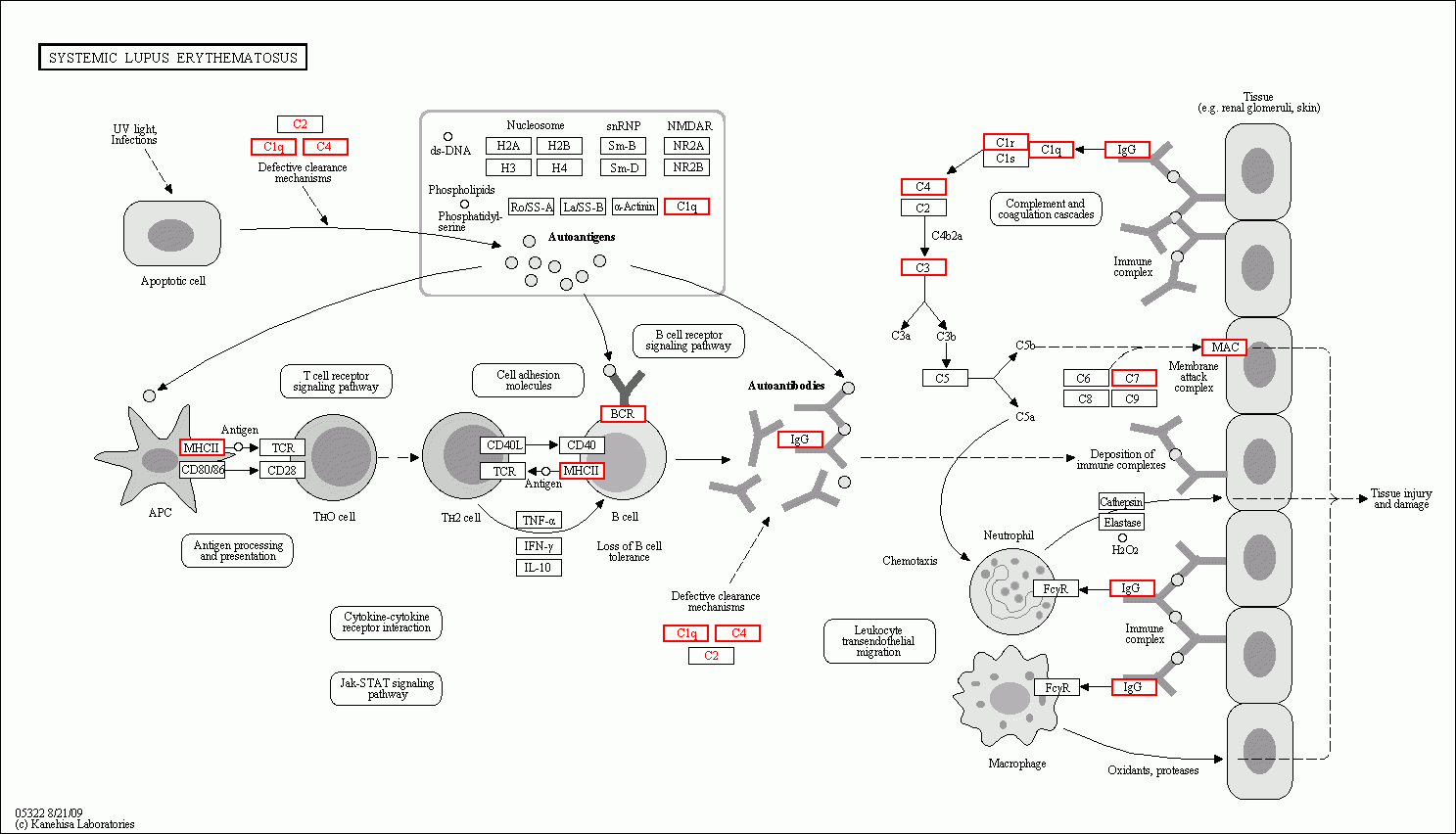

Supplement: S1 File — (ZIP) [file pone.0143219.s003.zip › pathway map/4 map05322.png]

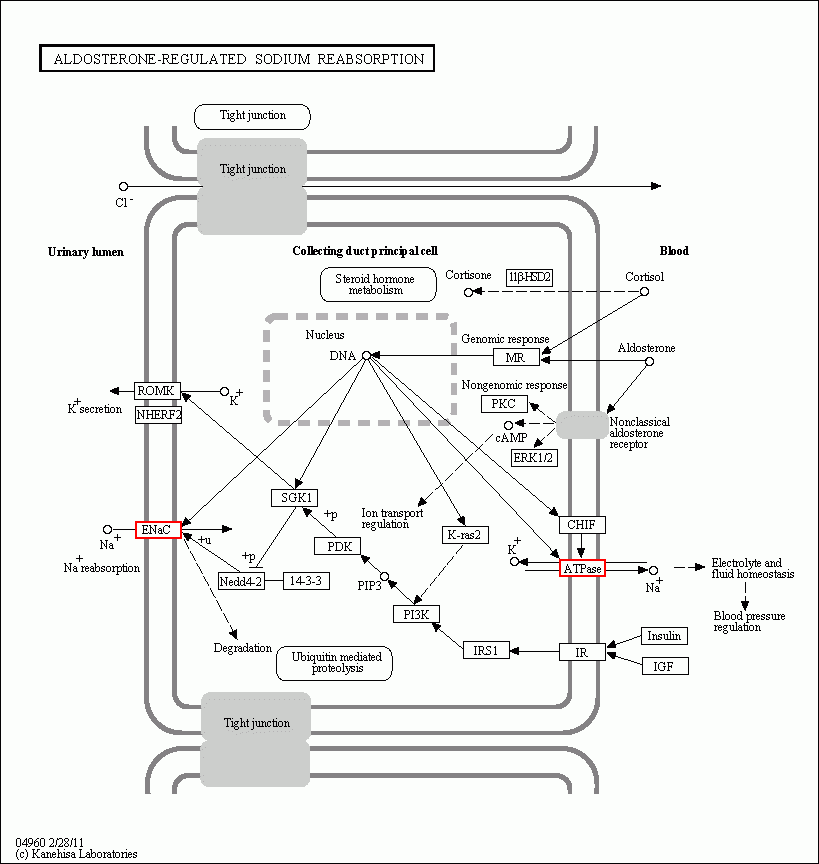

Supplement: S1 File — (ZIP) [file pone.0143219.s003.zip › pathway map/40 map04960.png]

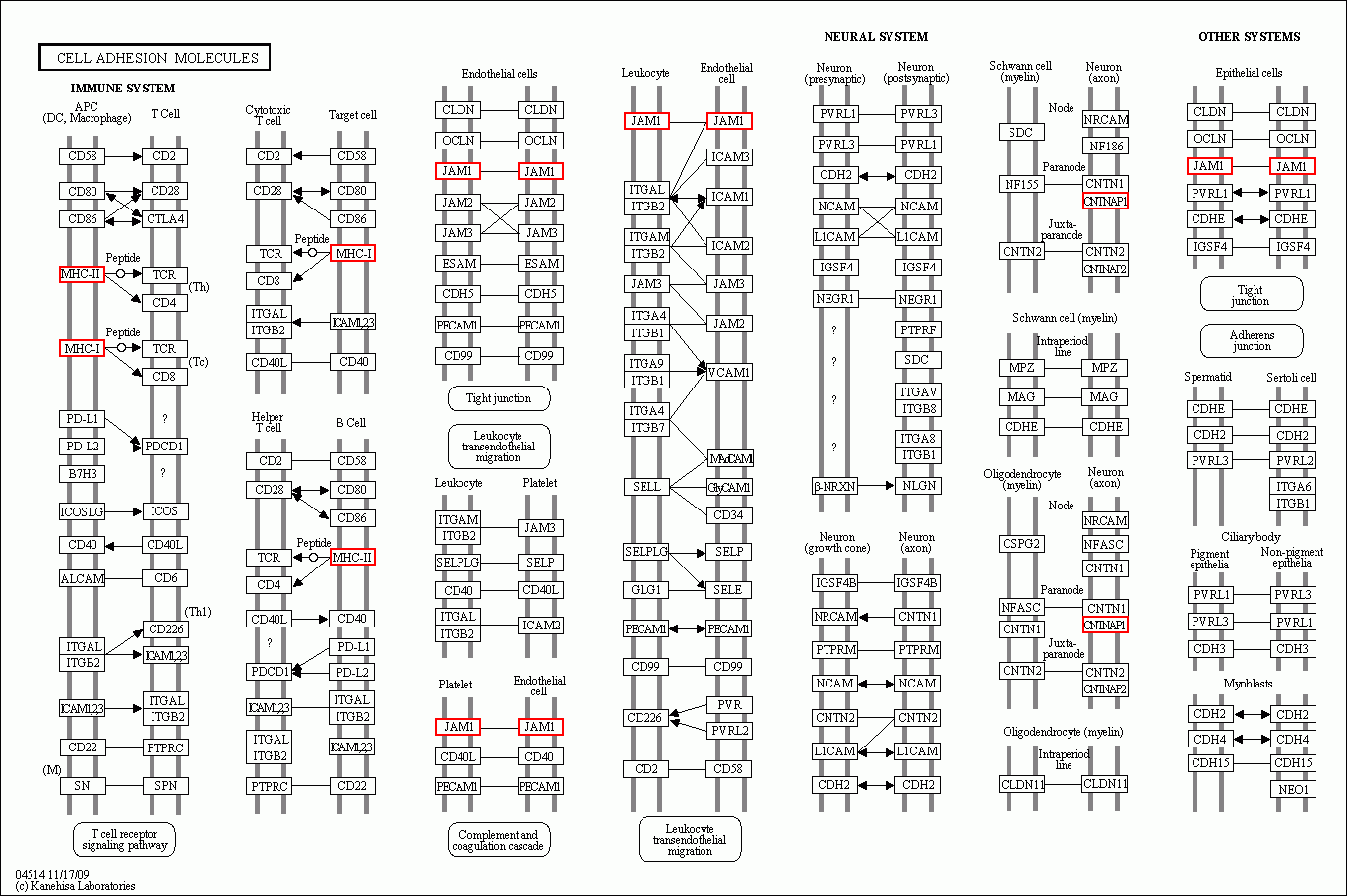

Supplement: S1 File — (ZIP) [file pone.0143219.s003.zip › pathway map/41 map04514.png]

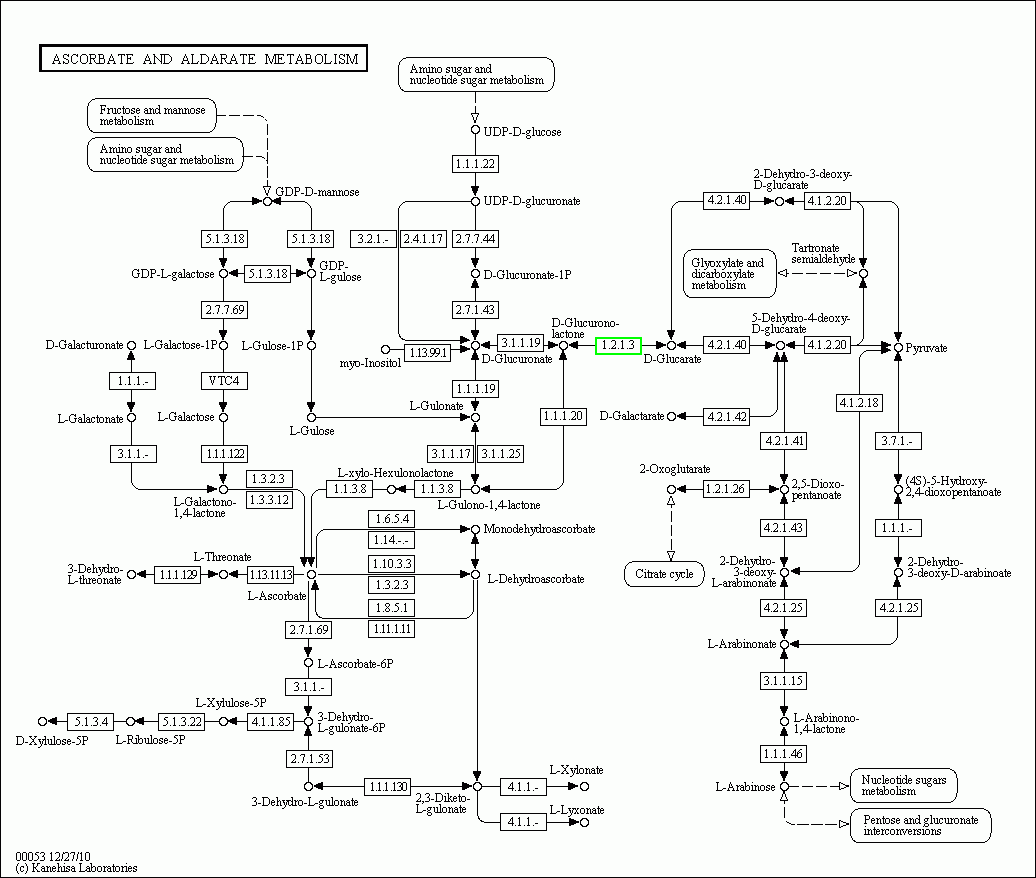

Supplement: S1 File — (ZIP) [file pone.0143219.s003.zip › pathway map/43 map00053.png]

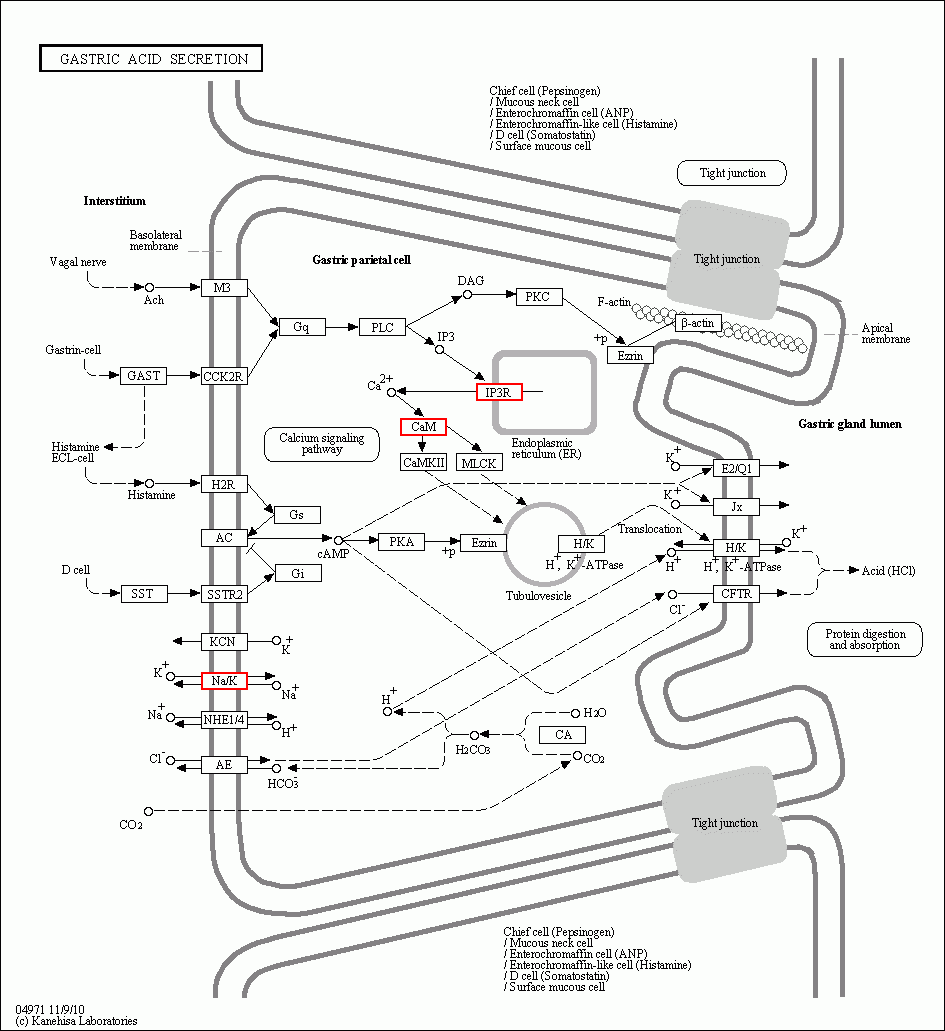

Supplement: S1 File — (ZIP) [file pone.0143219.s003.zip › pathway map/44 map04971.png]

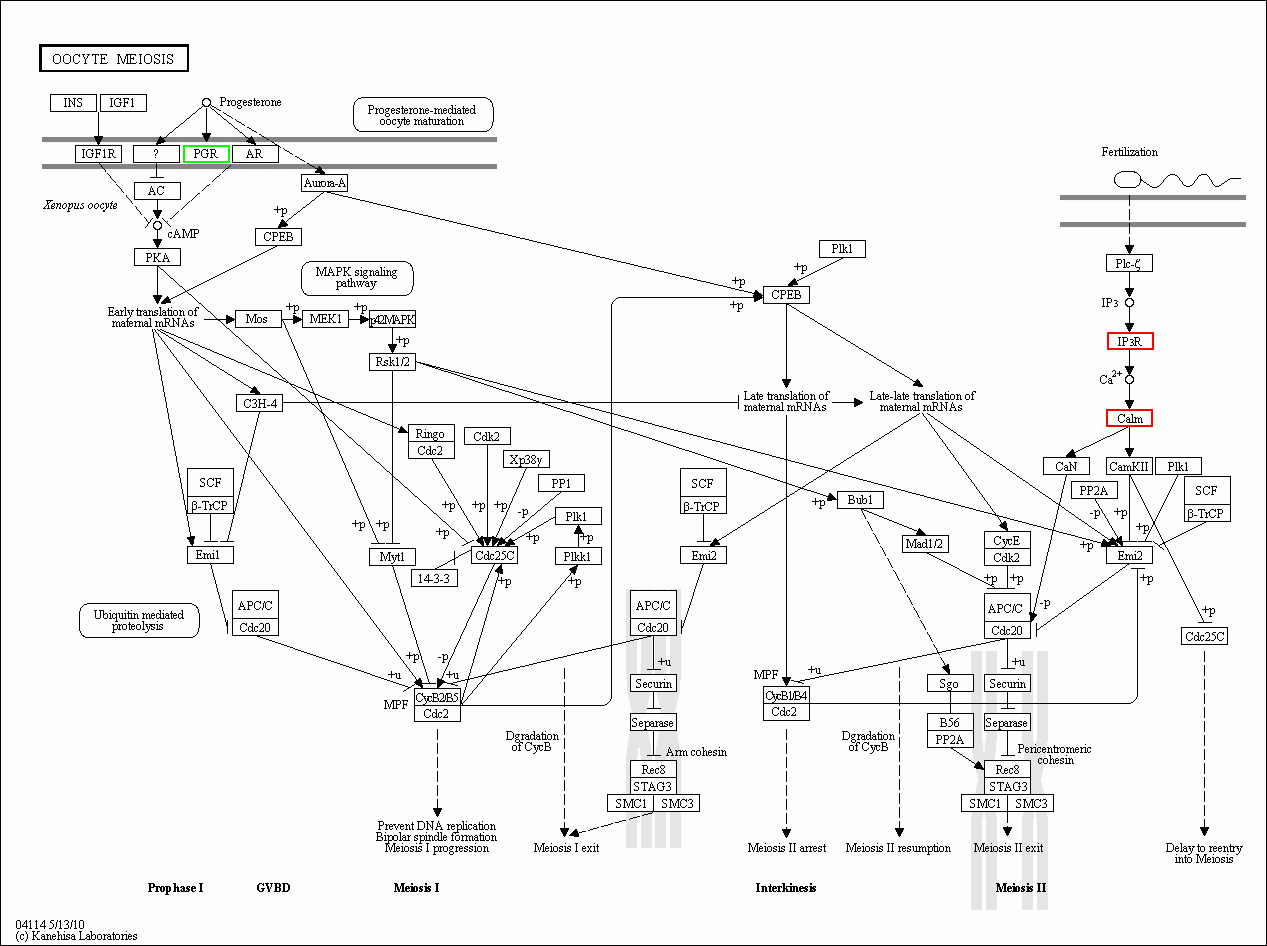

Supplement: S1 File — (ZIP) [file pone.0143219.s003.zip › pathway map/45 map04114.png]

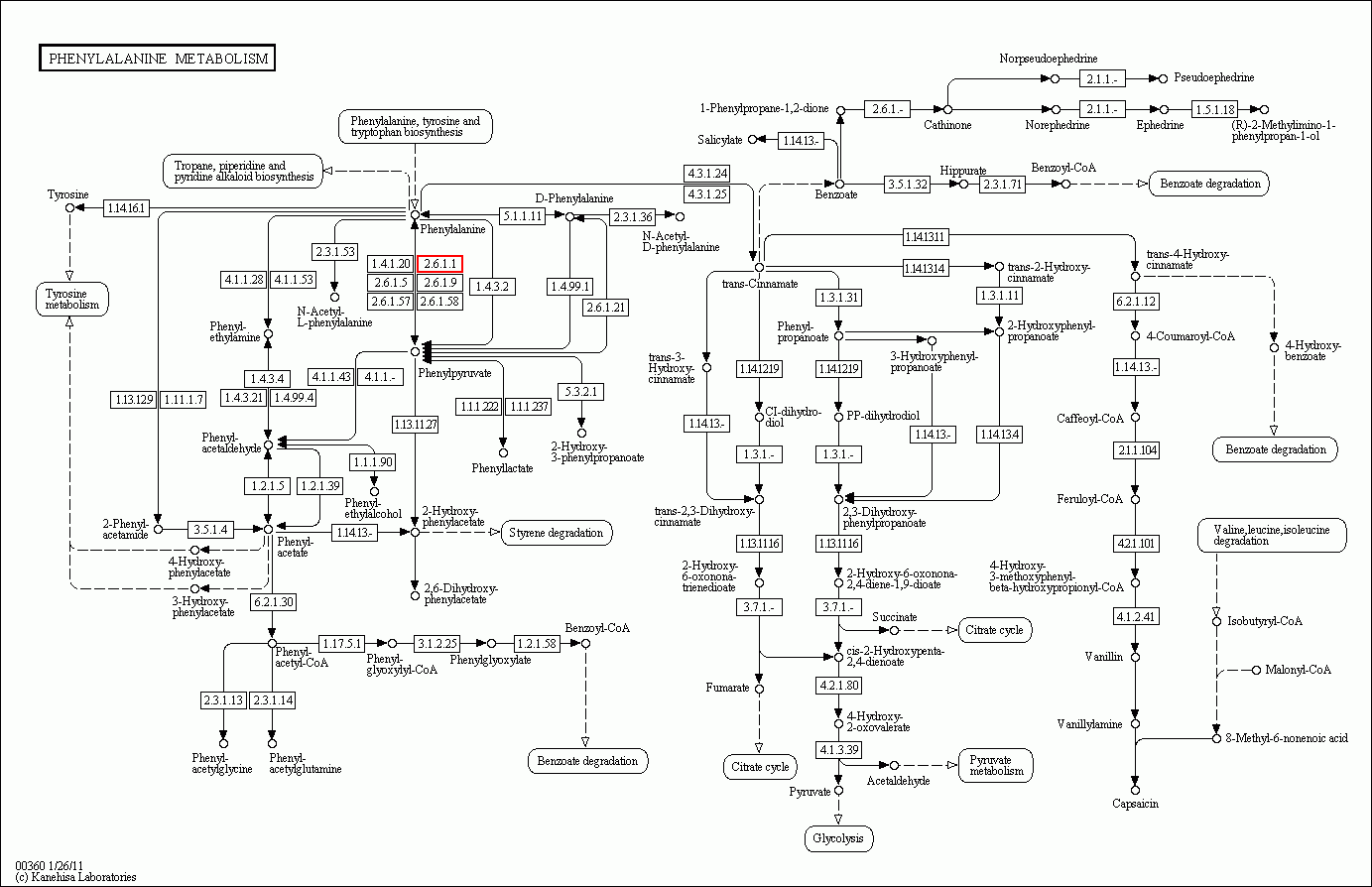

Supplement: S1 File — (ZIP) [file pone.0143219.s003.zip › pathway map/46 map00360.png]

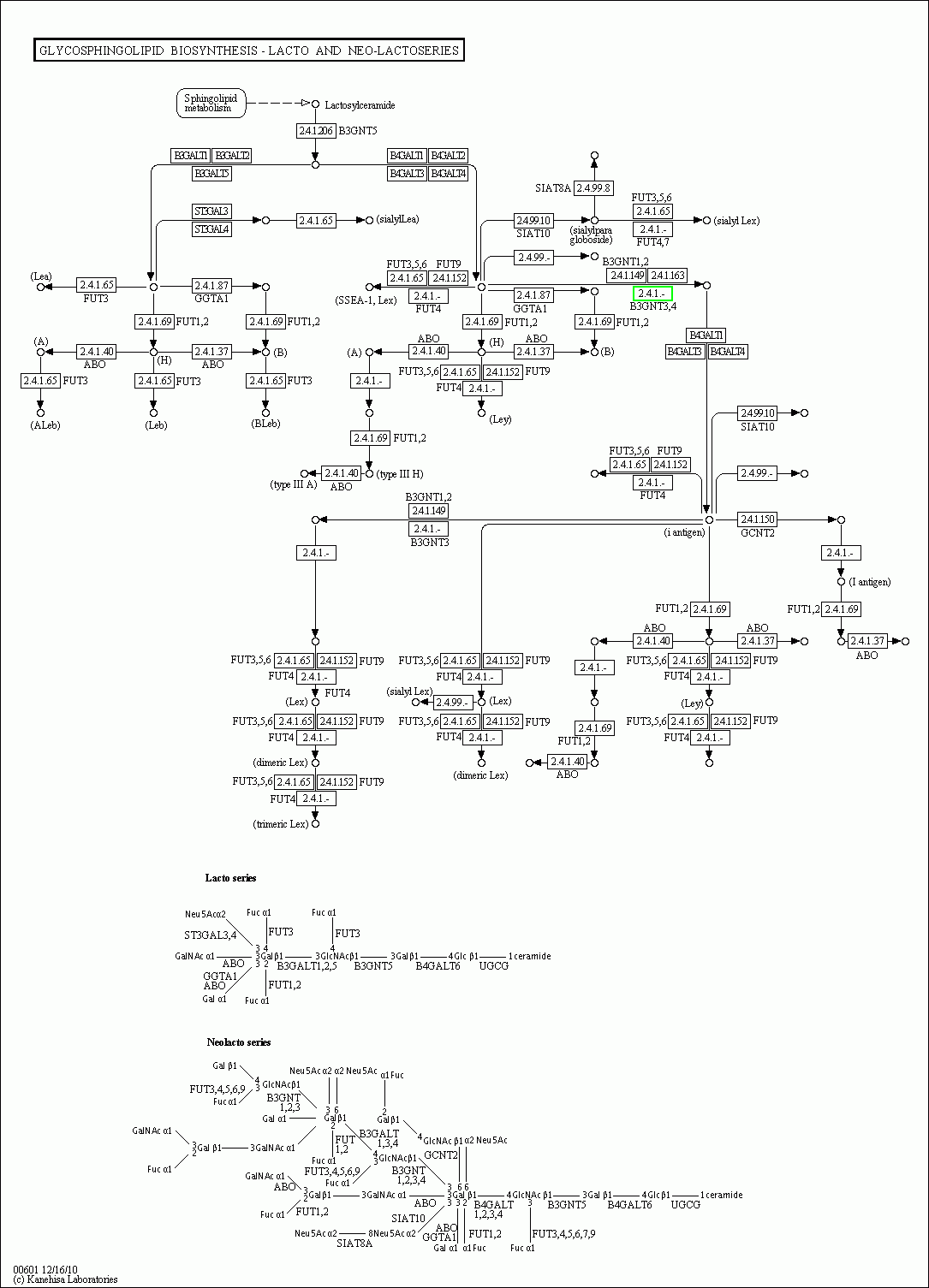

Supplement: S1 File — (ZIP) [file pone.0143219.s003.zip › pathway map/47 map00601.png]

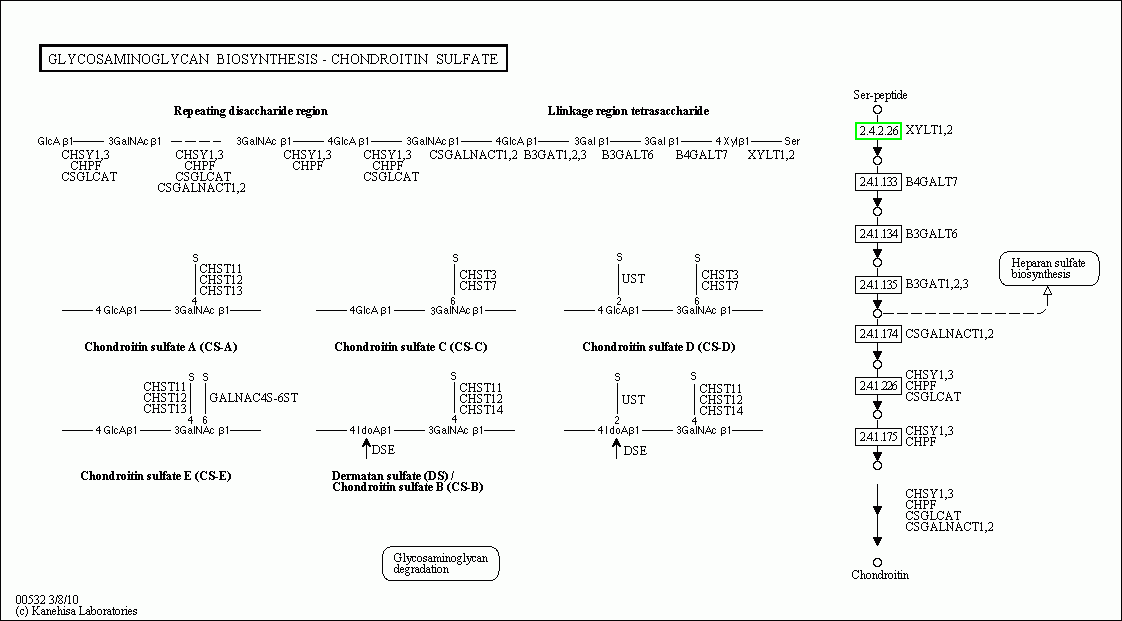

Supplement: S1 File — (ZIP) [file pone.0143219.s003.zip › pathway map/48 map00532.png]

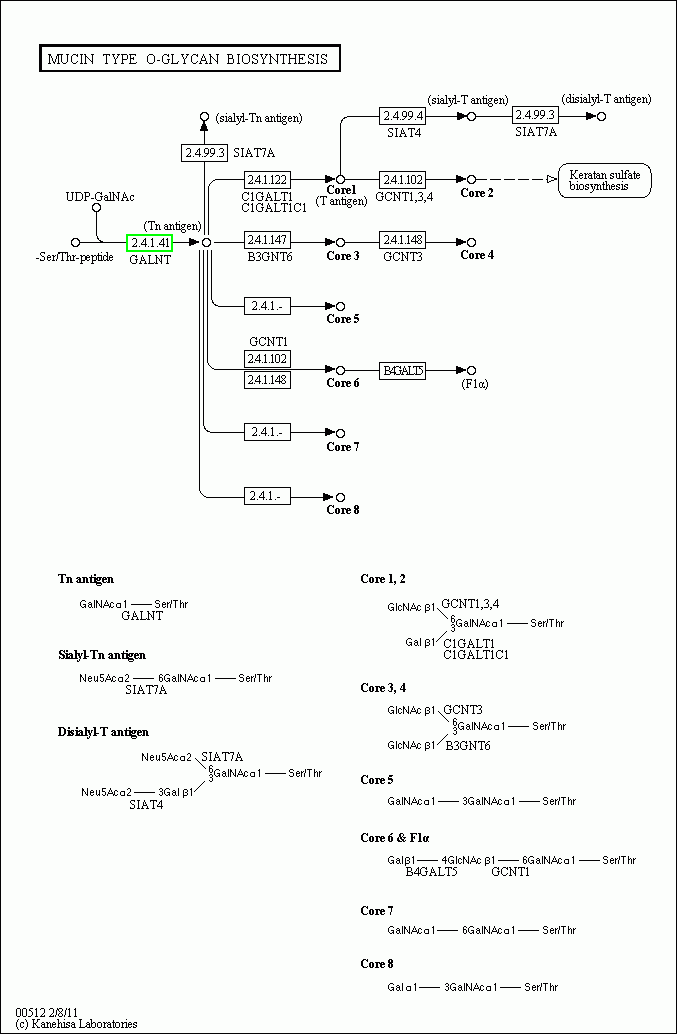

Supplement: S1 File — (ZIP) [file pone.0143219.s003.zip › pathway map/49 map00512.png]

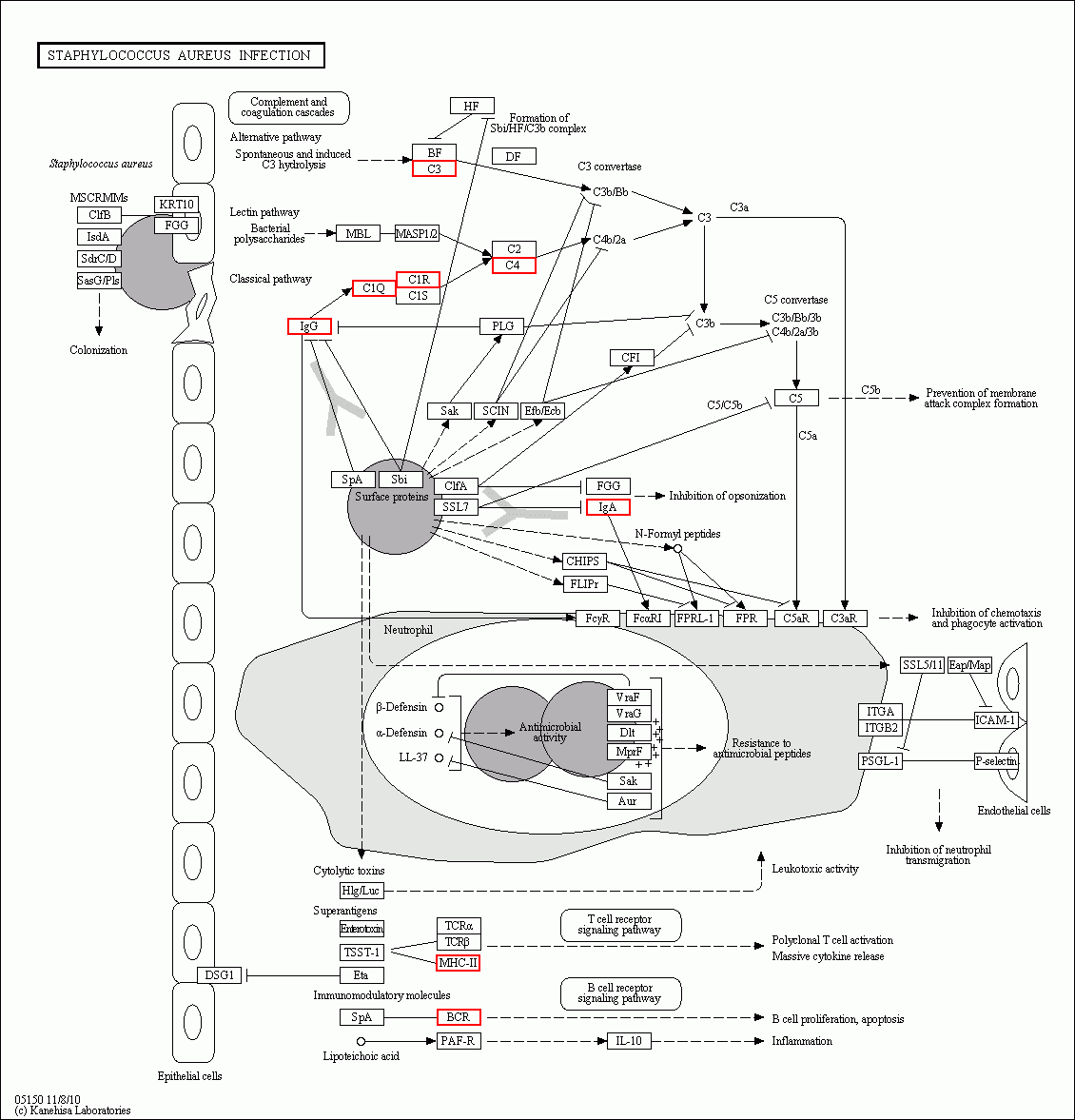

Supplement: S1 File — (ZIP) [file pone.0143219.s003.zip › pathway map/5 map05150.png]

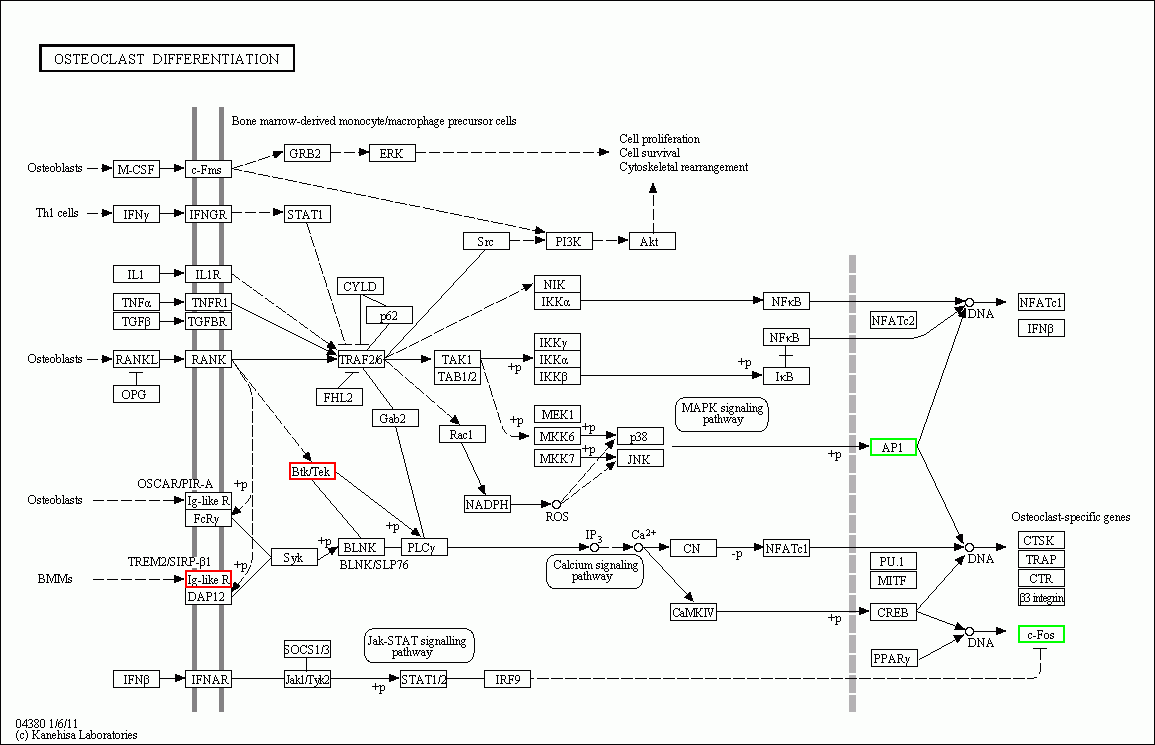

Supplement: S1 File — (ZIP) [file pone.0143219.s003.zip › pathway map/50 map04380.png]

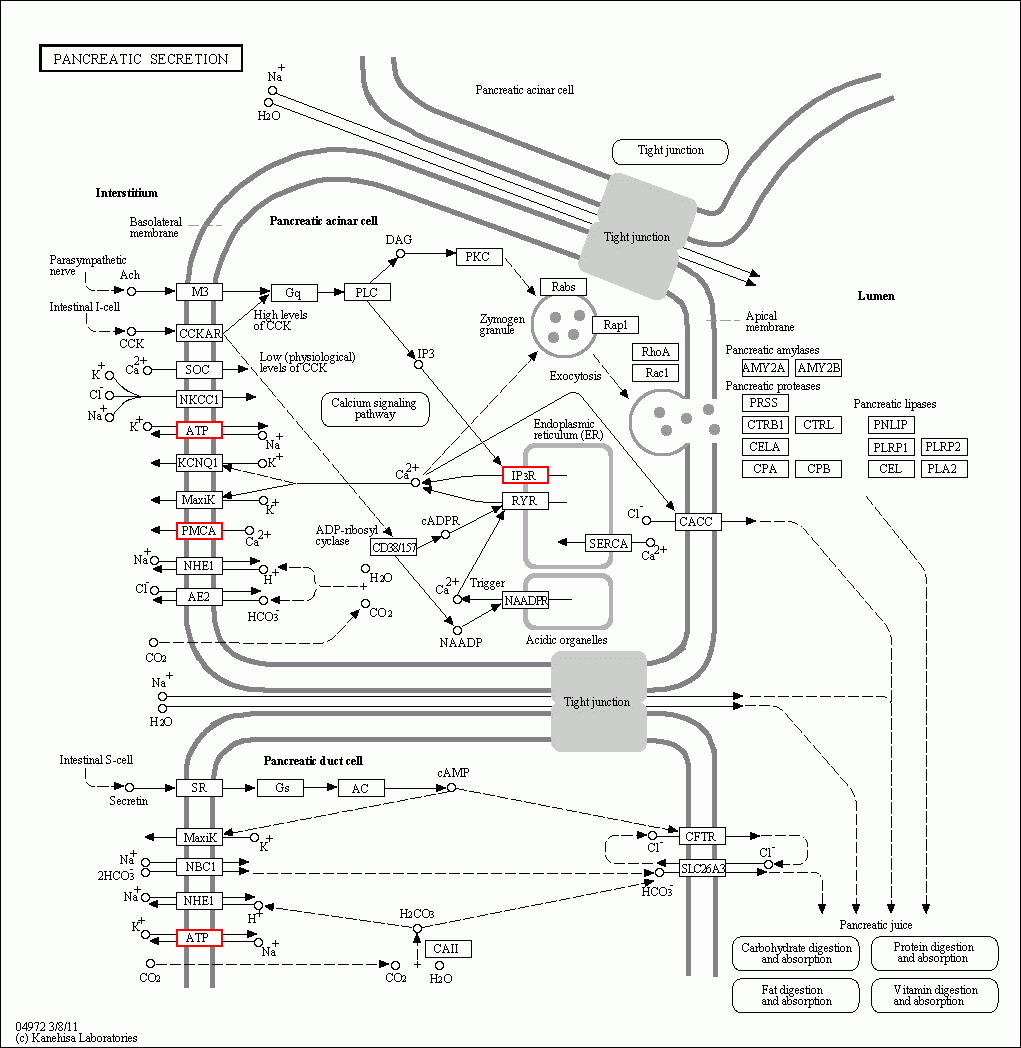

Supplement: S1 File — (ZIP) [file pone.0143219.s003.zip › pathway map/51 map04972.png]

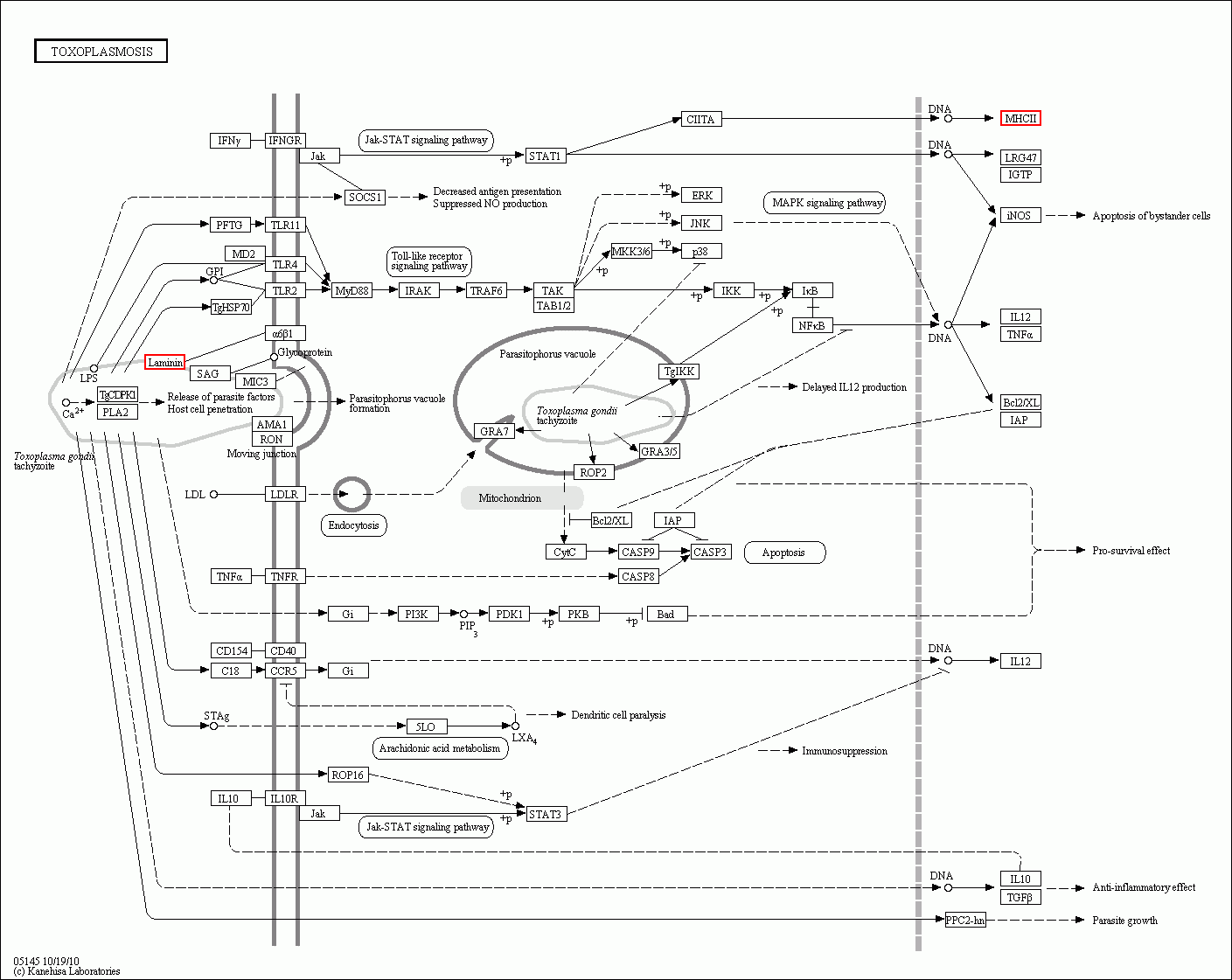

Supplement: S1 File — (ZIP) [file pone.0143219.s003.zip › pathway map/52 map05145.png]

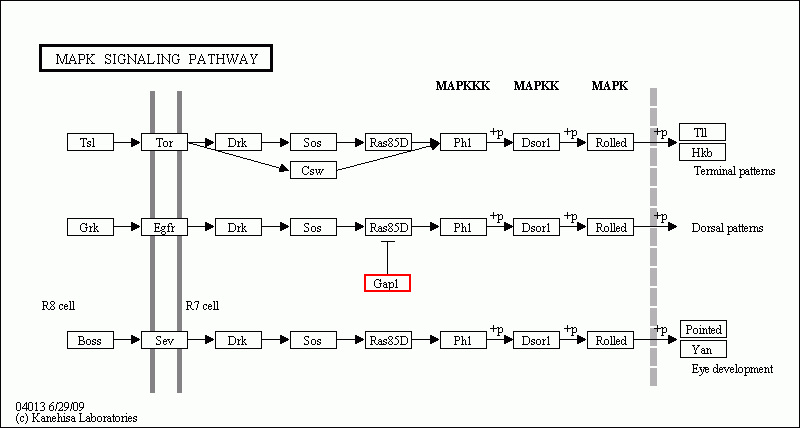

Supplement: S1 File — (ZIP) [file pone.0143219.s003.zip › pathway map/53 map04013.png]

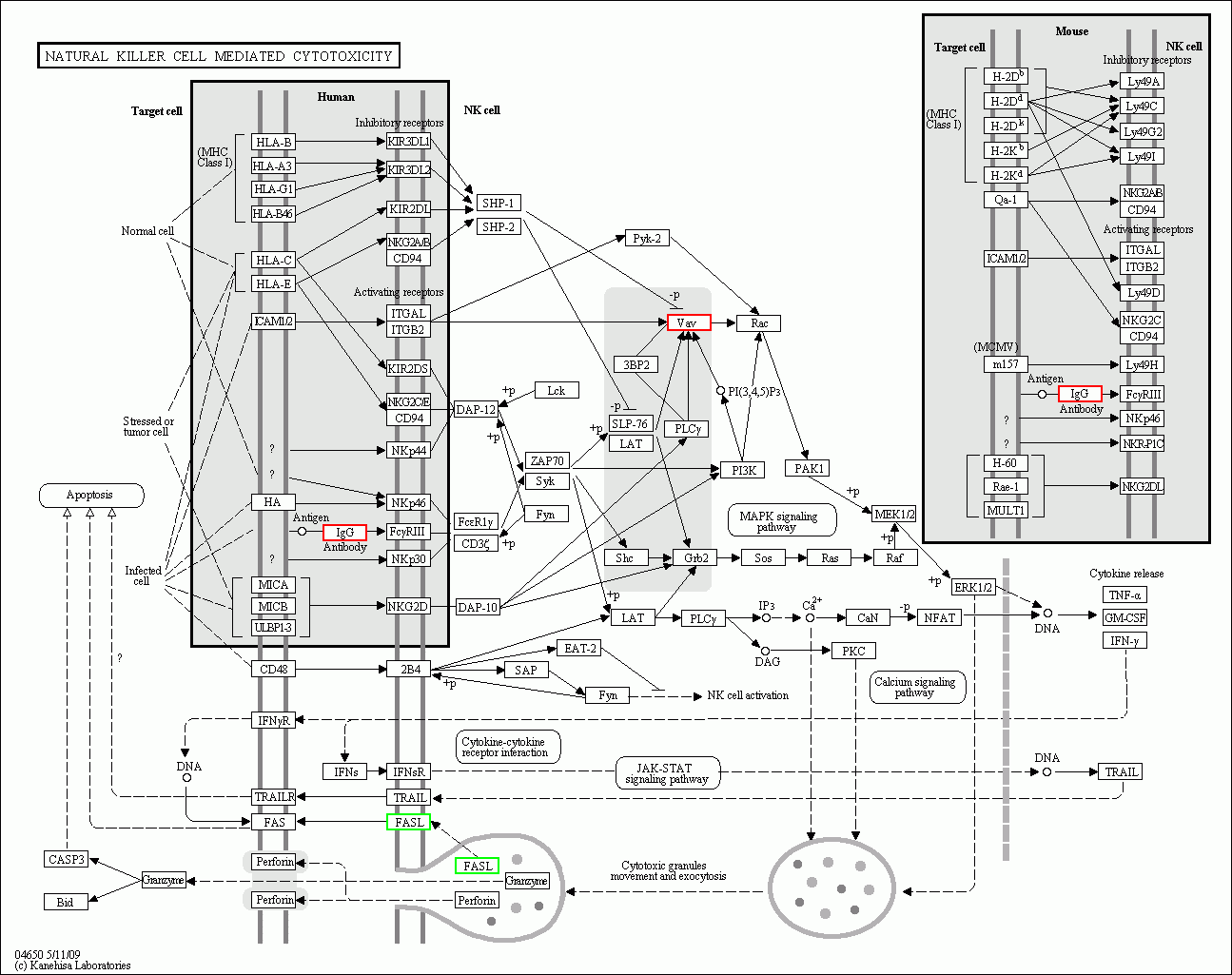

Supplement: S1 File — (ZIP) [file pone.0143219.s003.zip › pathway map/54 map04650.png]

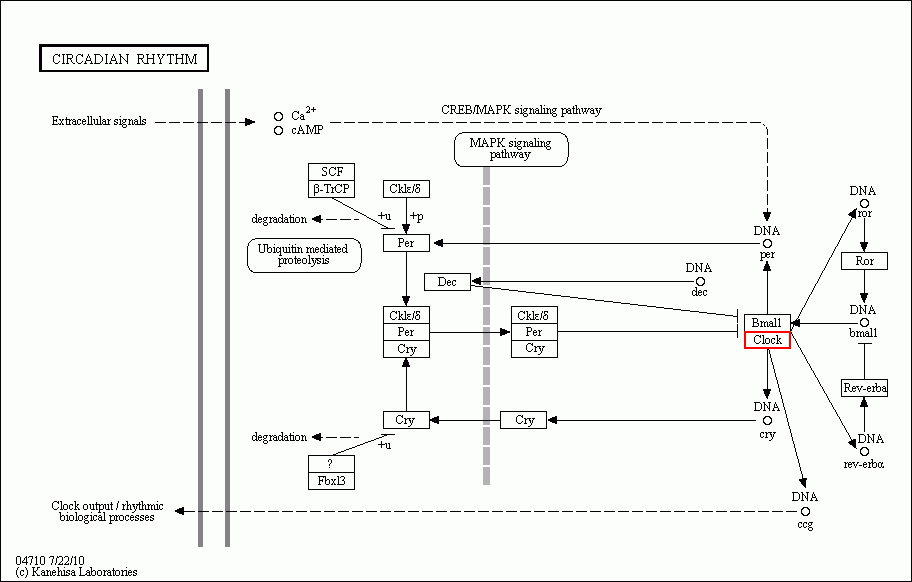

Supplement: S1 File — (ZIP) [file pone.0143219.s003.zip › pathway map/55 map04710.png]

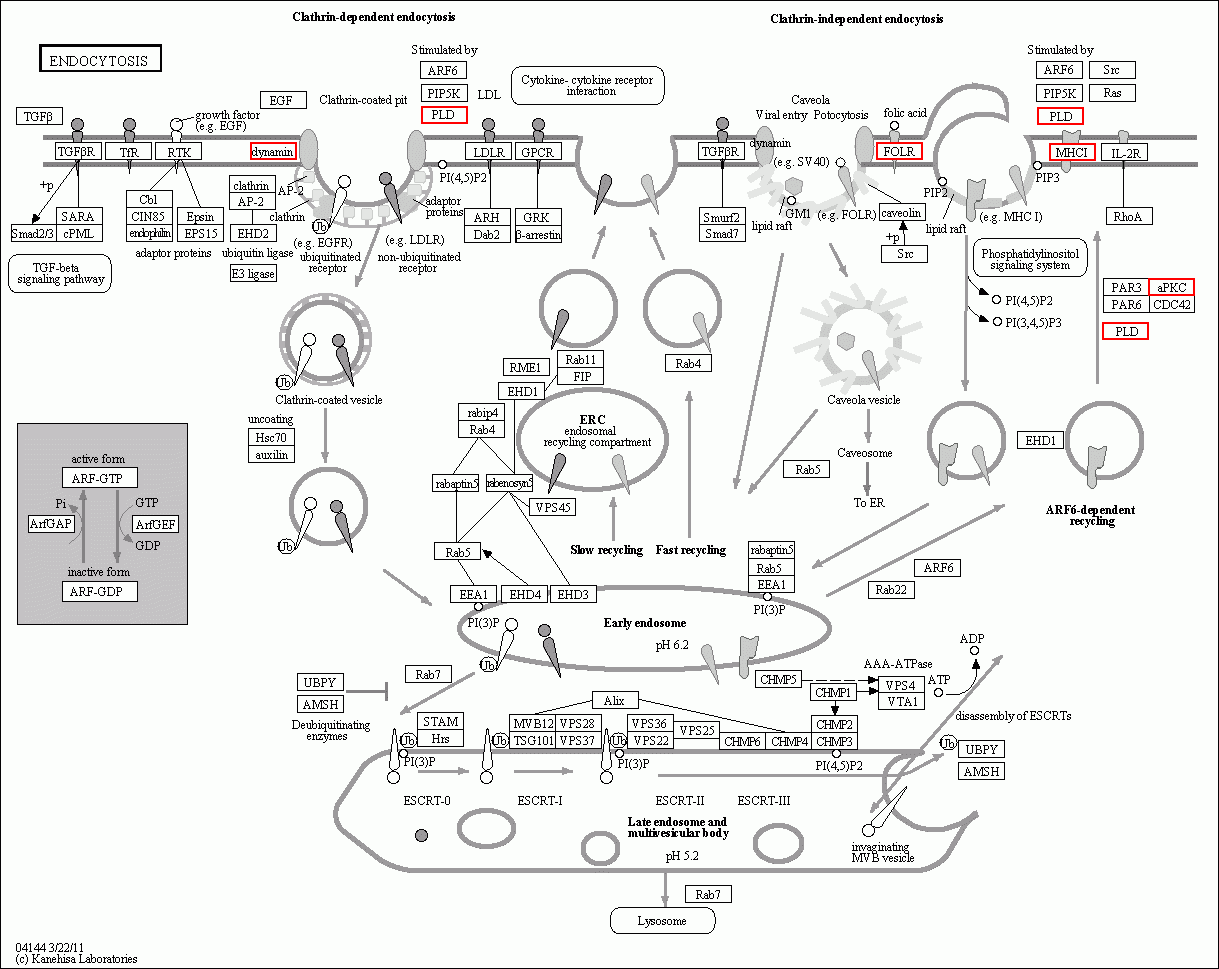

Supplement: S1 File — (ZIP) [file pone.0143219.s003.zip › pathway map/56 map04144.png]

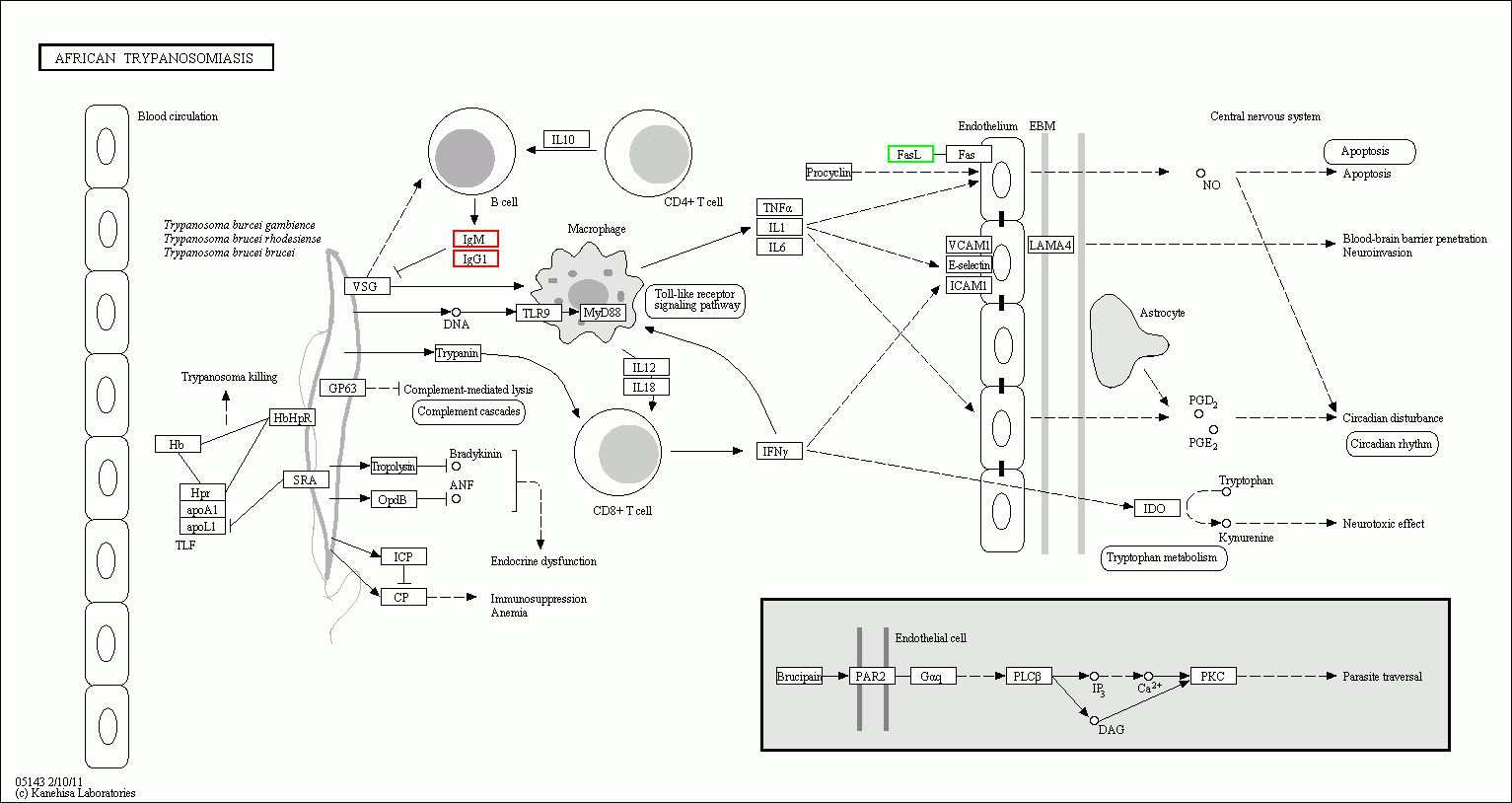

Supplement: S1 File — (ZIP) [file pone.0143219.s003.zip › pathway map/57 map05143.png]

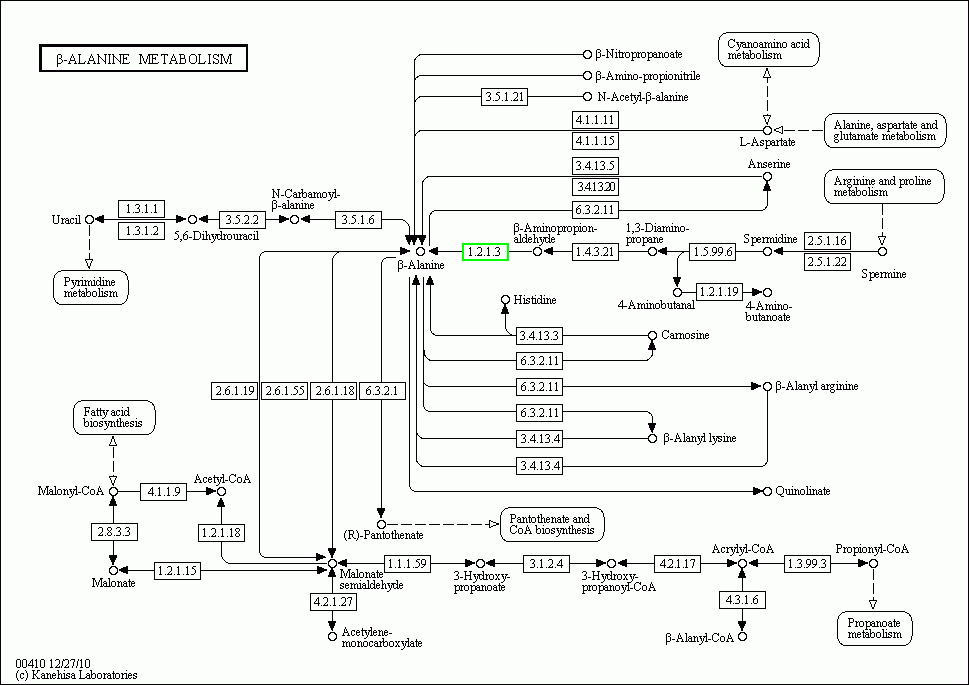

Supplement: S1 File — (ZIP) [file pone.0143219.s003.zip › pathway map/58 map00410.png]

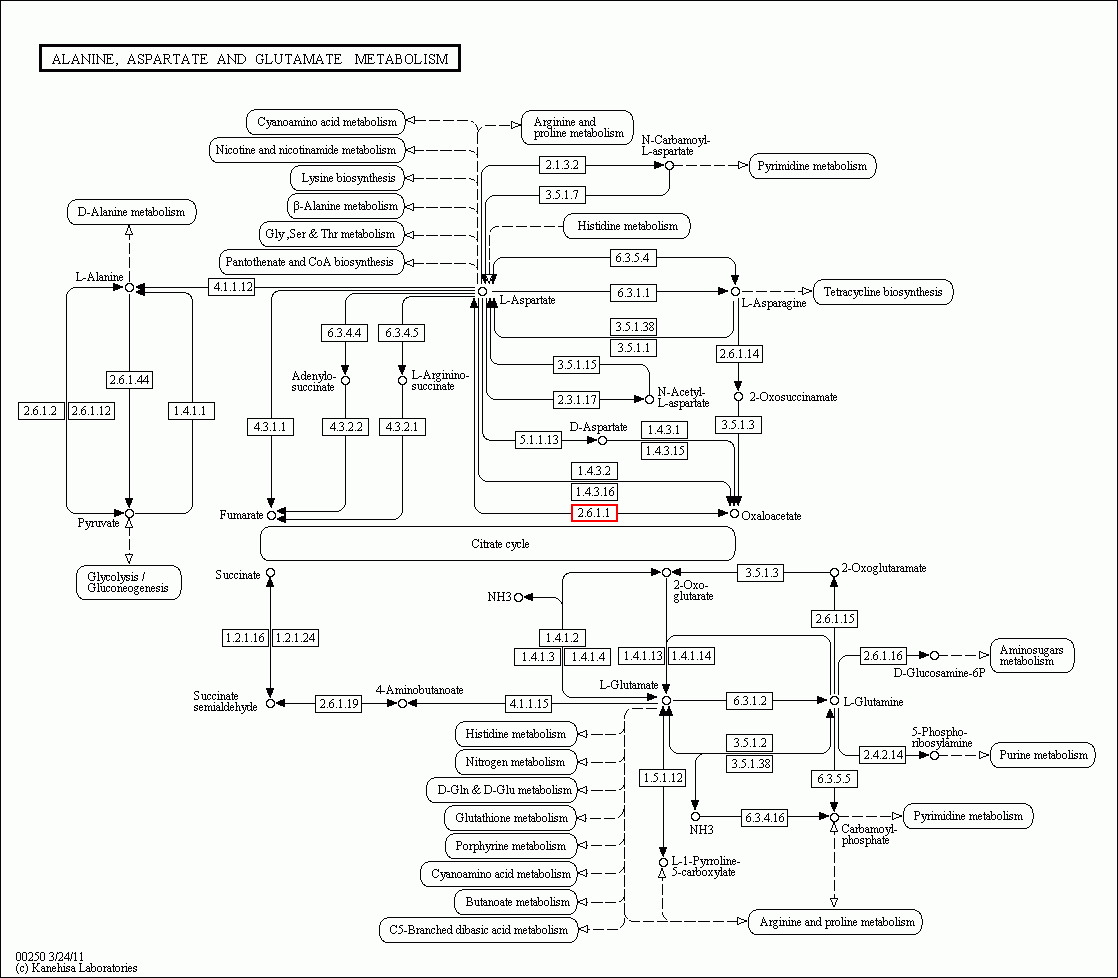

Supplement: S1 File — (ZIP) [file pone.0143219.s003.zip › pathway map/59 map00250.png]

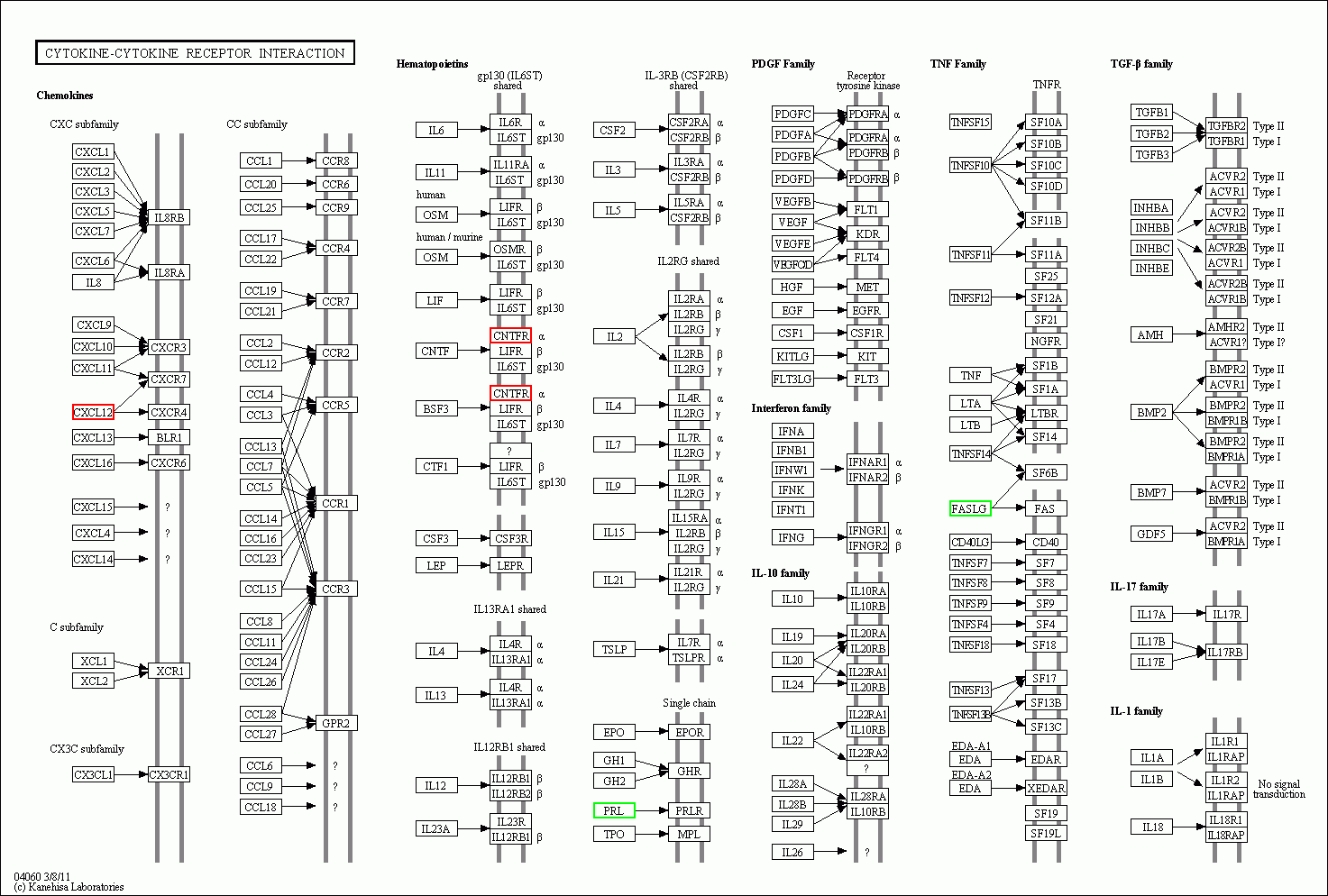

Supplement: S1 File — (ZIP) [file pone.0143219.s003.zip › pathway map/6 map04060.png]

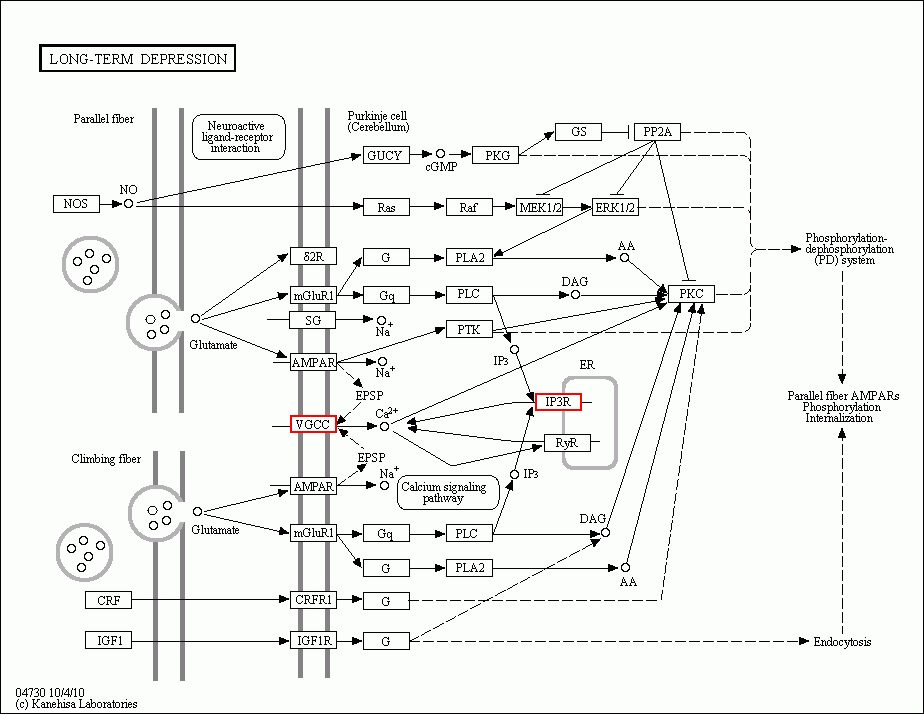

Supplement: S1 File — (ZIP) [file pone.0143219.s003.zip › pathway map/60 map04730.png]

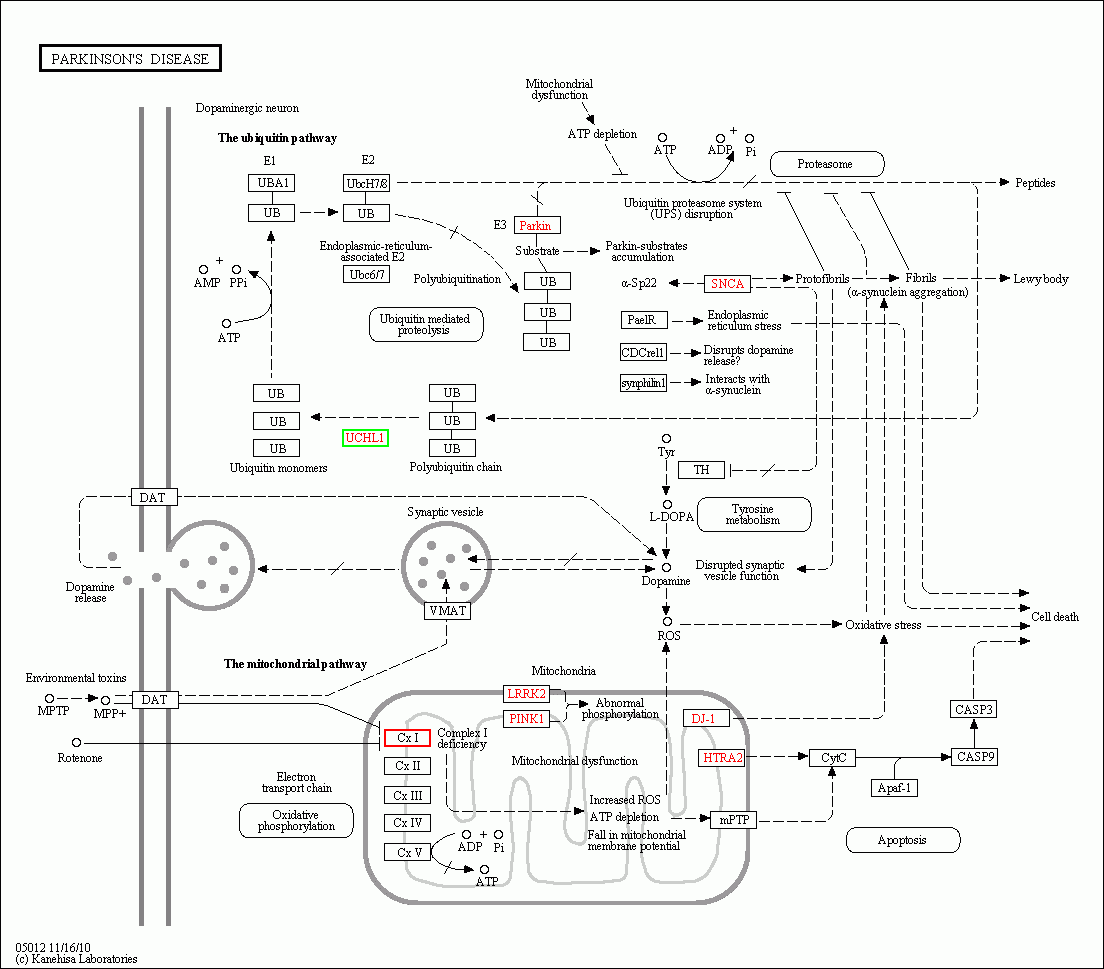

Supplement: S1 File — (ZIP) [file pone.0143219.s003.zip › pathway map/61 map05012.png]

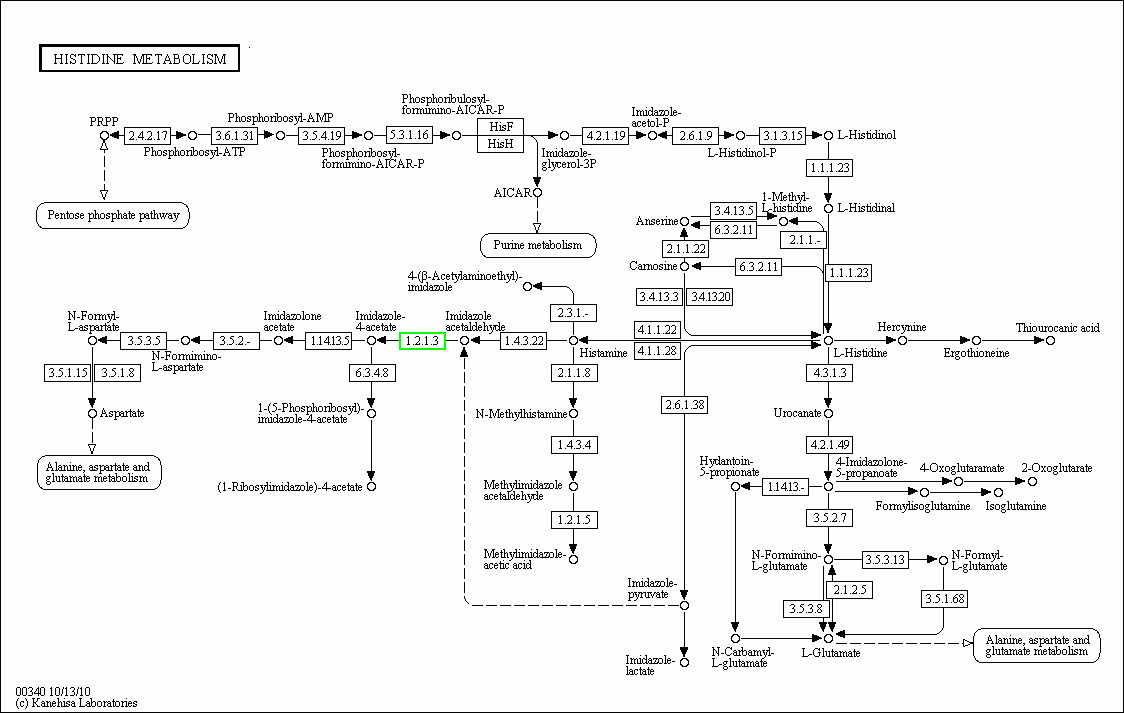

Supplement: S1 File — (ZIP) [file pone.0143219.s003.zip › pathway map/62 map00340.png]

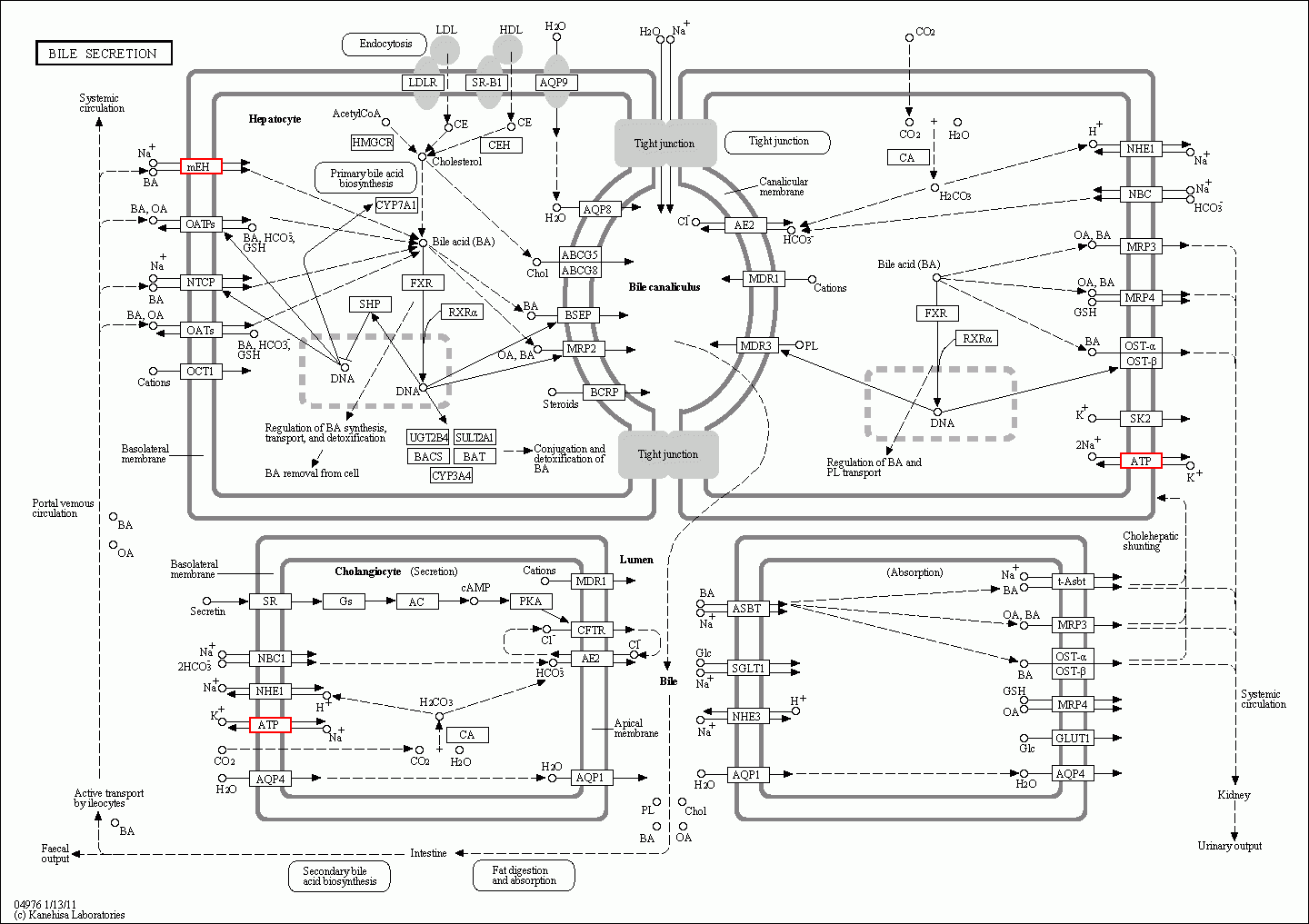

Supplement: S1 File — (ZIP) [file pone.0143219.s003.zip › pathway map/63 map04976.png]

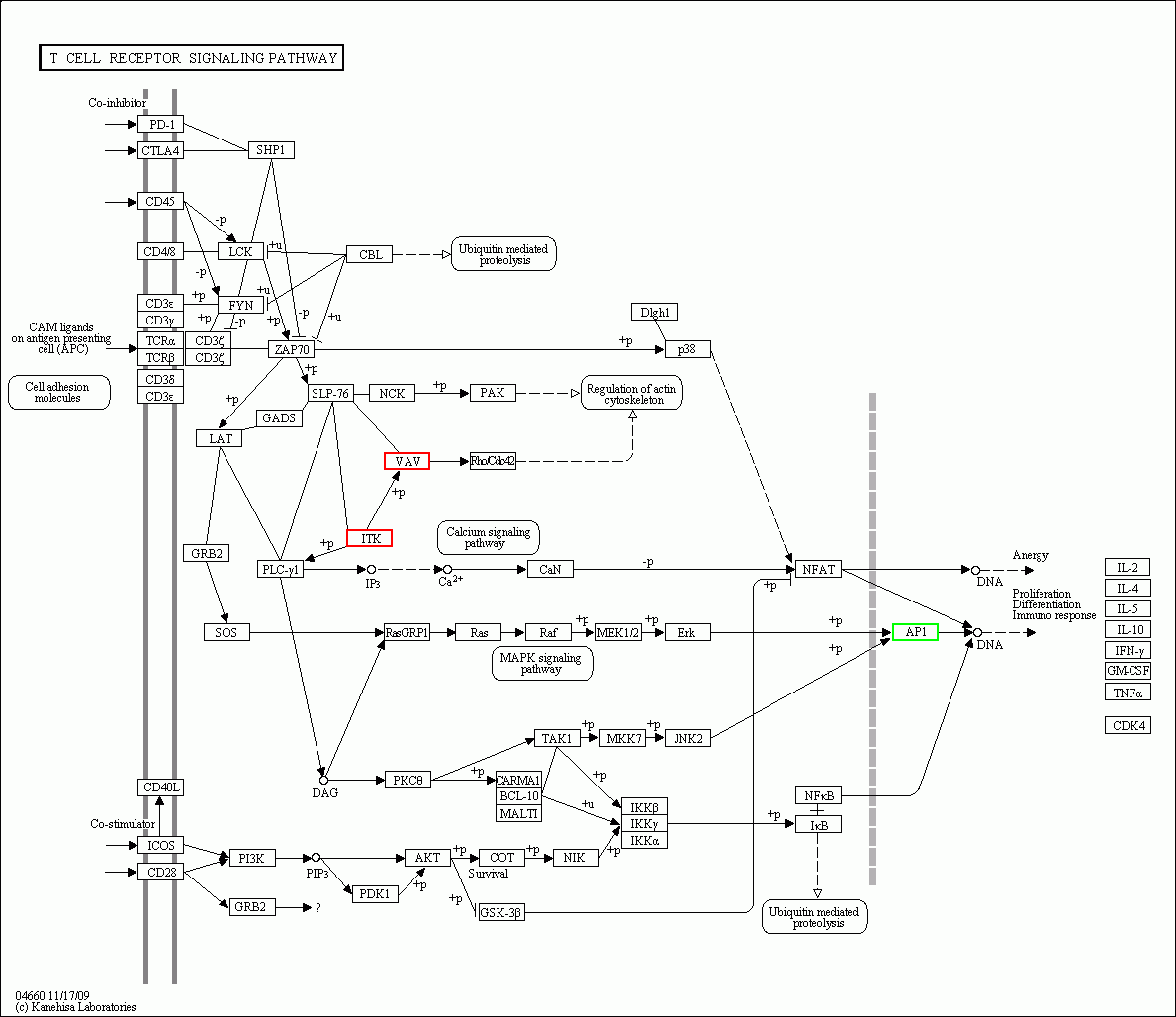

Supplement: S1 File — (ZIP) [file pone.0143219.s003.zip › pathway map/64 map04660.png]

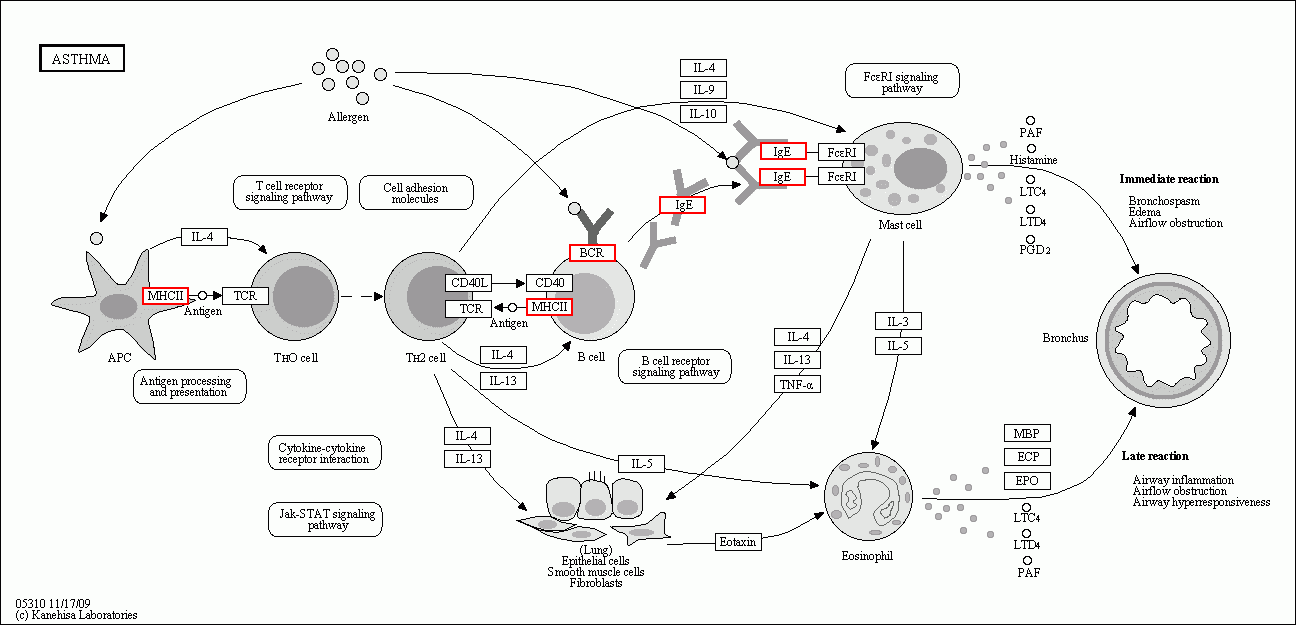

Supplement: S1 File — (ZIP) [file pone.0143219.s003.zip › pathway map/65 map05310.png]

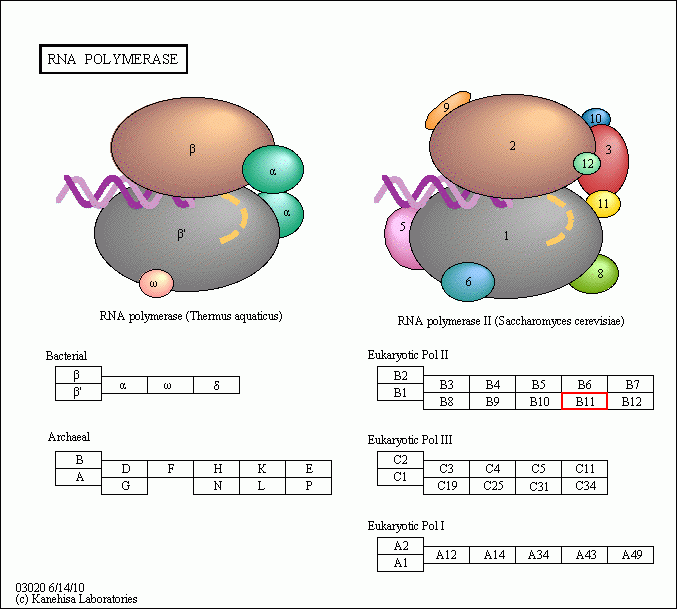

Supplement: S1 File — (ZIP) [file pone.0143219.s003.zip › pathway map/66 map03020.png]
