# Supplementary figures and images for: Reproductive Hormone and Transcriptomic Responses of Pituitary Tissue in Anestrus Gilts Induced by Nutrient Restriction (part 2 of 2)
Source: PLoS One. 2015 Nov 18;10(11):e0143219. doi: 10.1371/journal.pone.0143219 (PMC4651501; doi:10.1371/journal.pone.0143219)

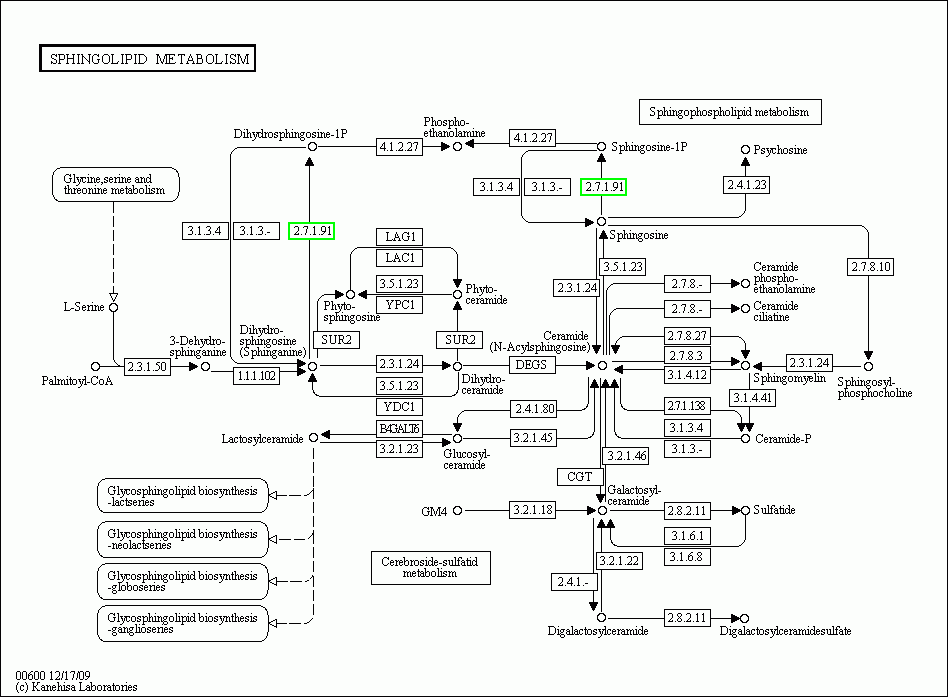

Supplement: S1 File — (ZIP) [file pone.0143219.s003.zip › pathway map/67 map00600.png]

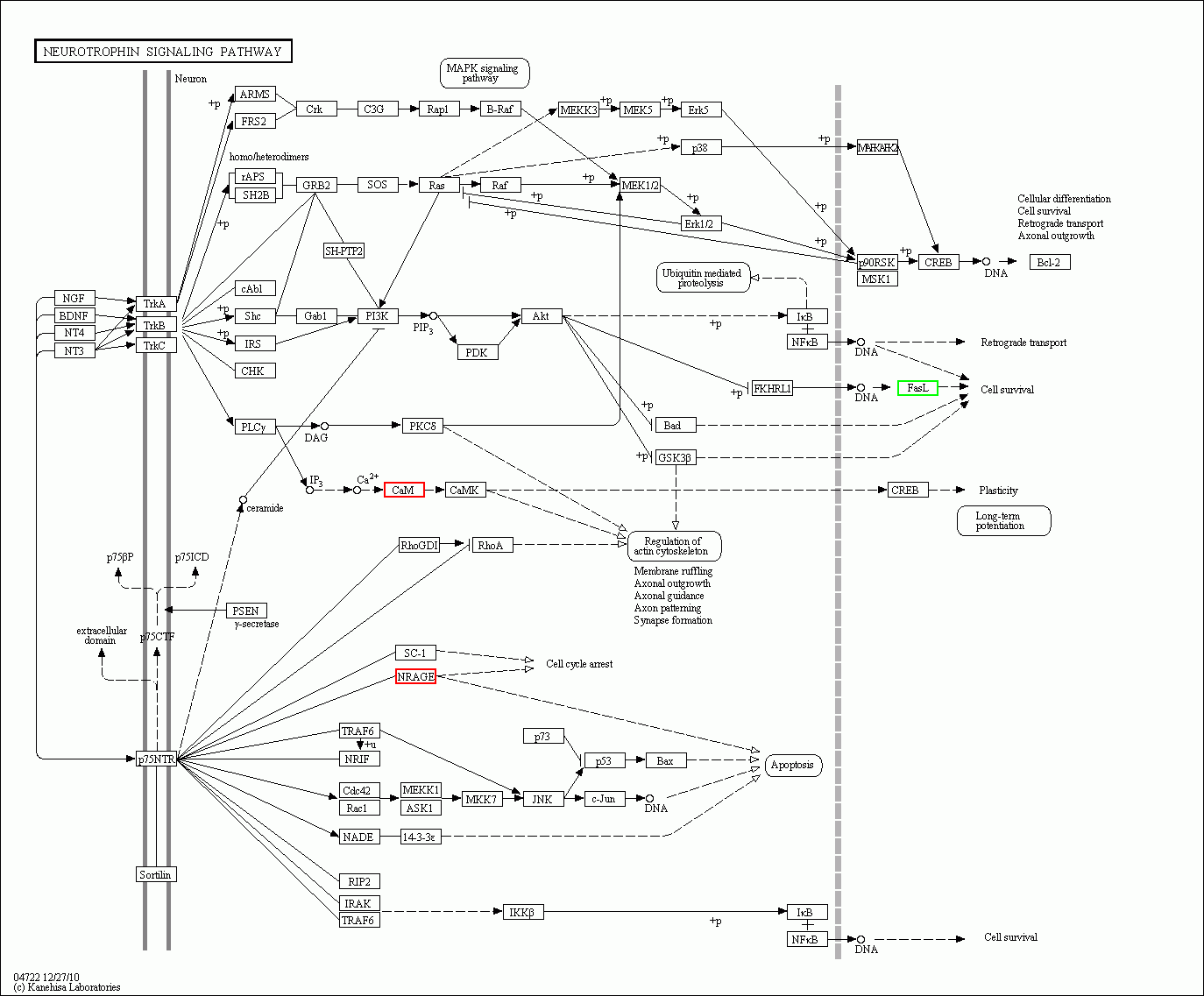

Supplement: S1 File — (ZIP) [file pone.0143219.s003.zip › pathway map/68 map04722.png]

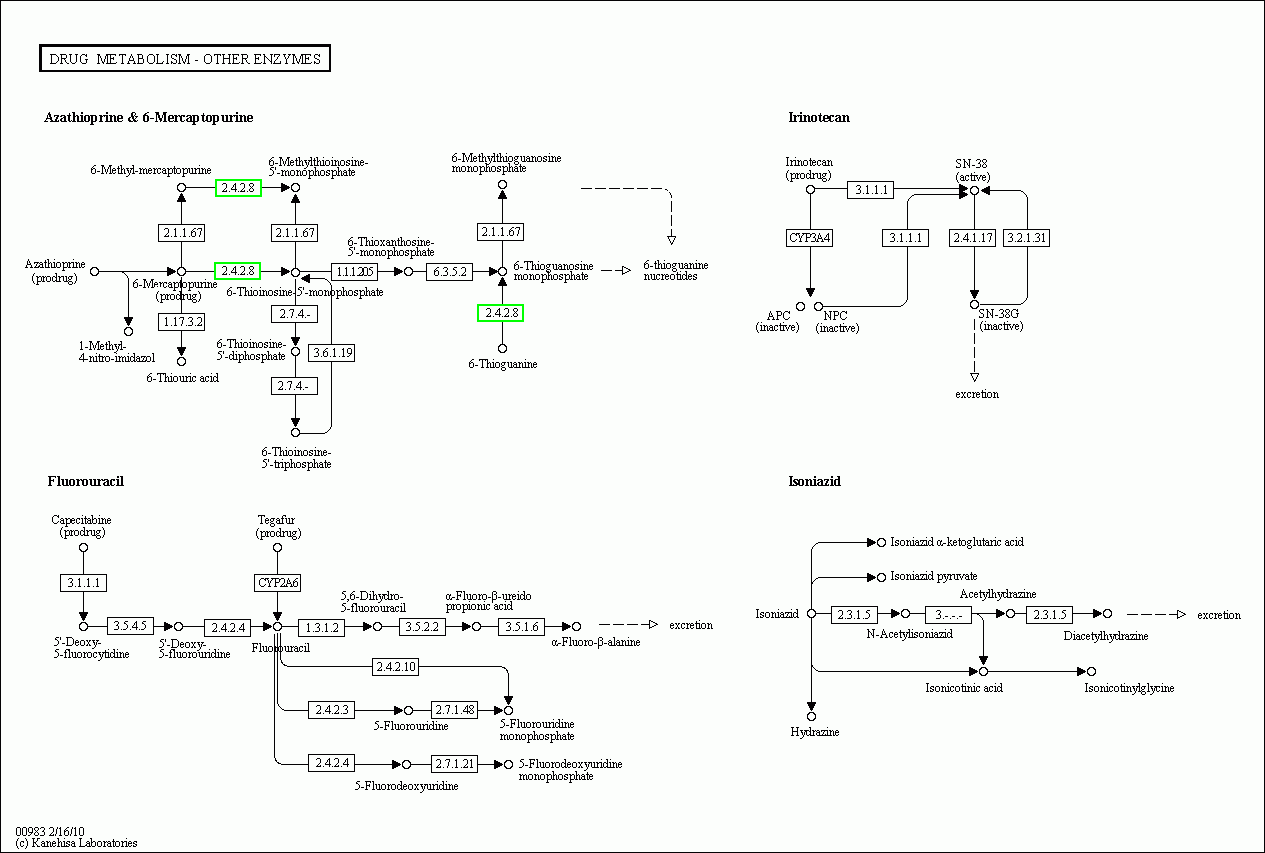

Supplement: S1 File — (ZIP) [file pone.0143219.s003.zip › pathway map/69 map00983.png]

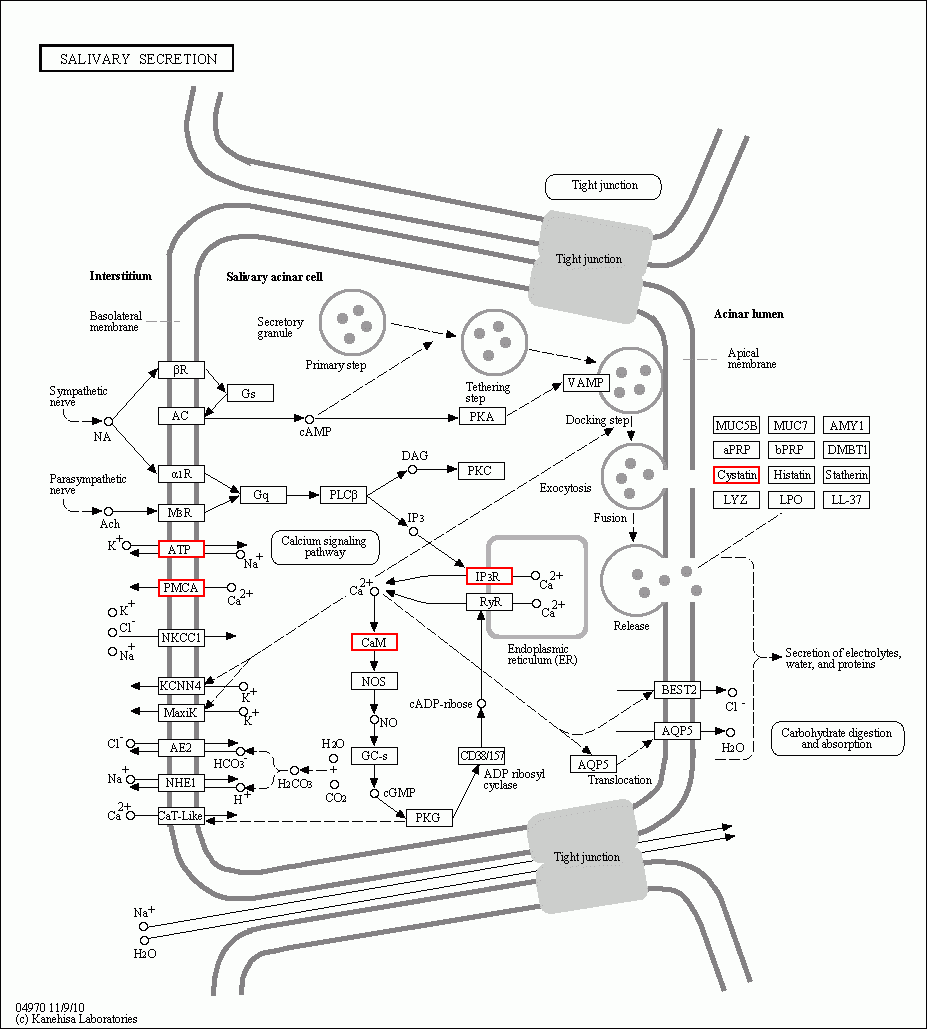

Supplement: S1 File — (ZIP) [file pone.0143219.s003.zip › pathway map/7 map04970.png]

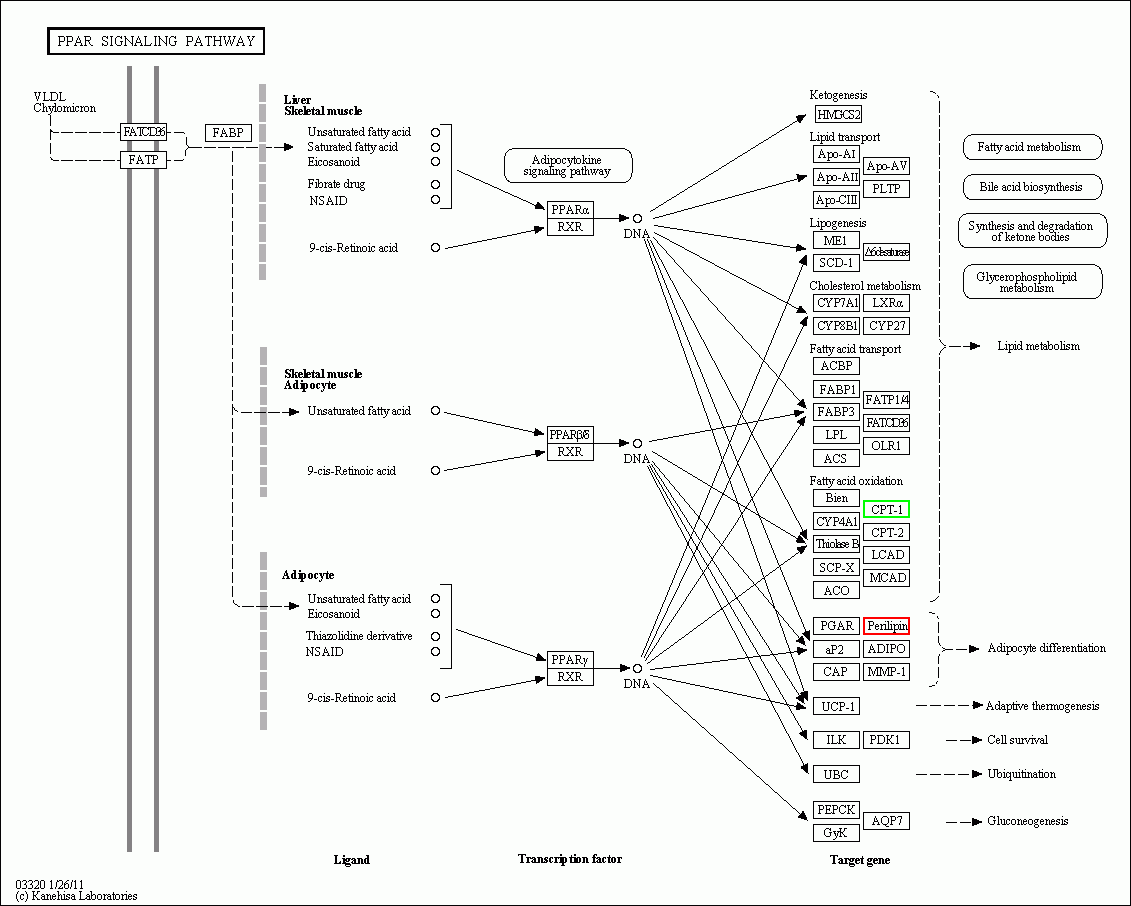

Supplement: S1 File — (ZIP) [file pone.0143219.s003.zip › pathway map/70 map03320.png]

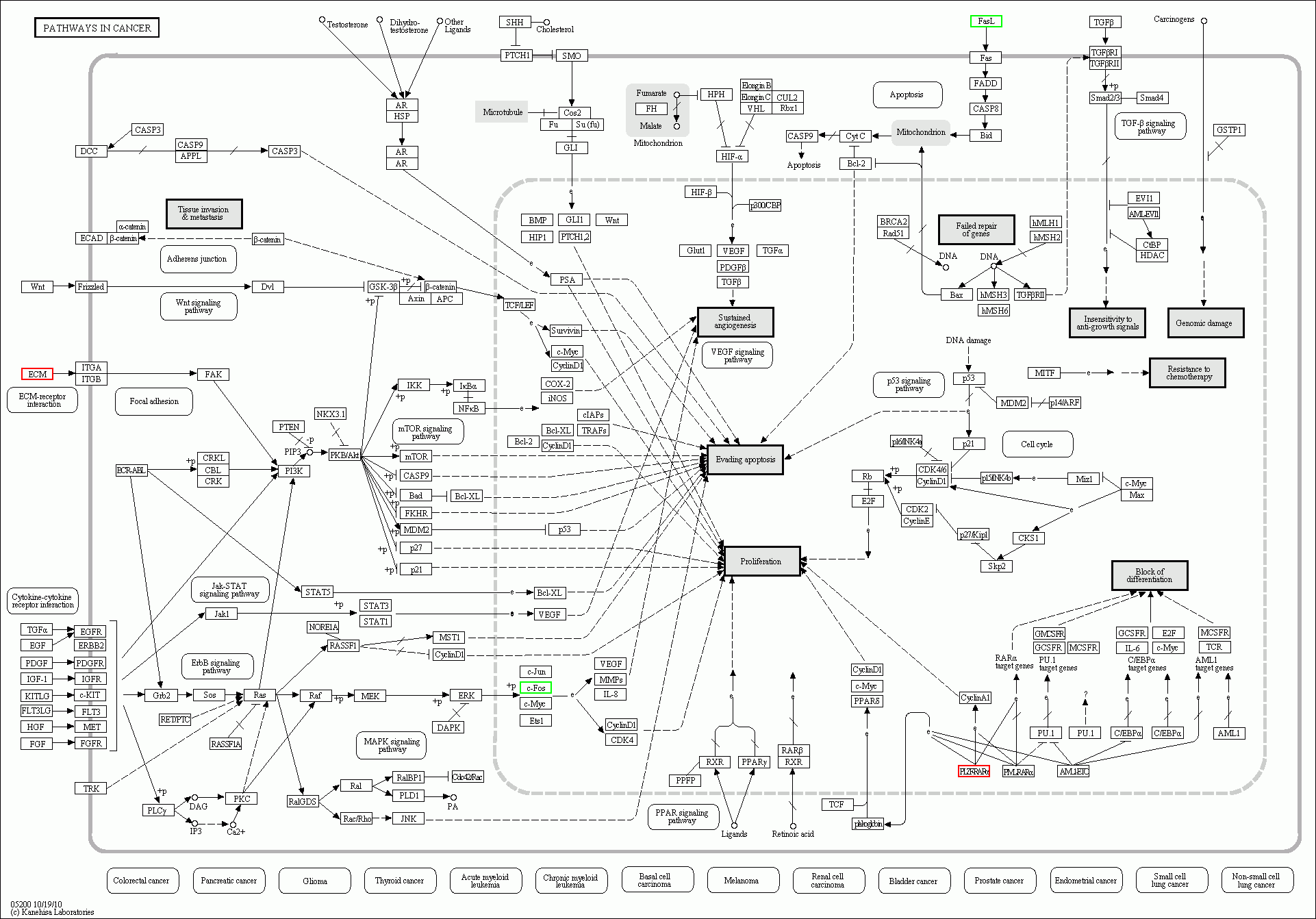

Supplement: S1 File — (ZIP) [file pone.0143219.s003.zip › pathway map/71 map05200.png]

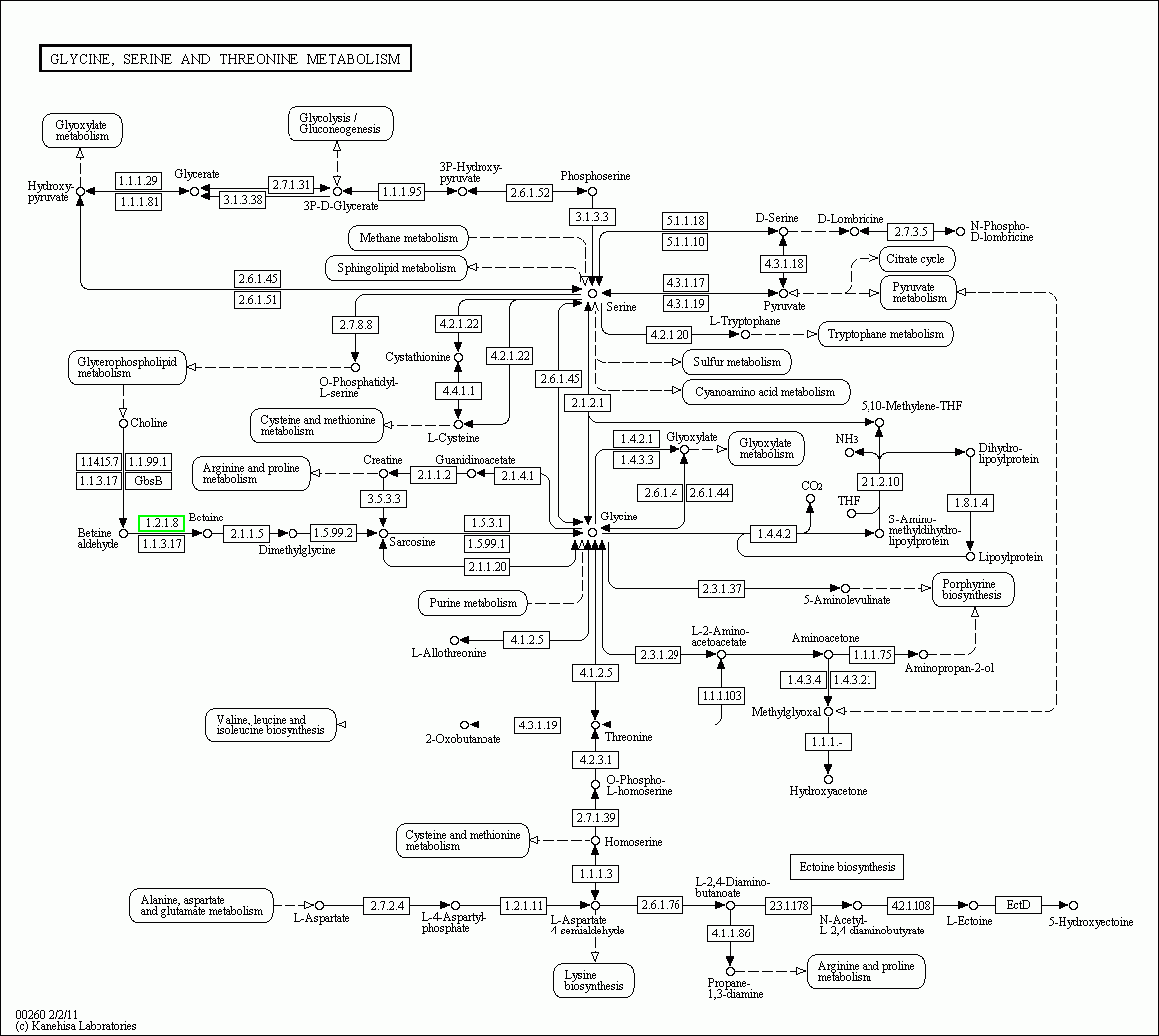

Supplement: S1 File — (ZIP) [file pone.0143219.s003.zip › pathway map/72 map00260.png]

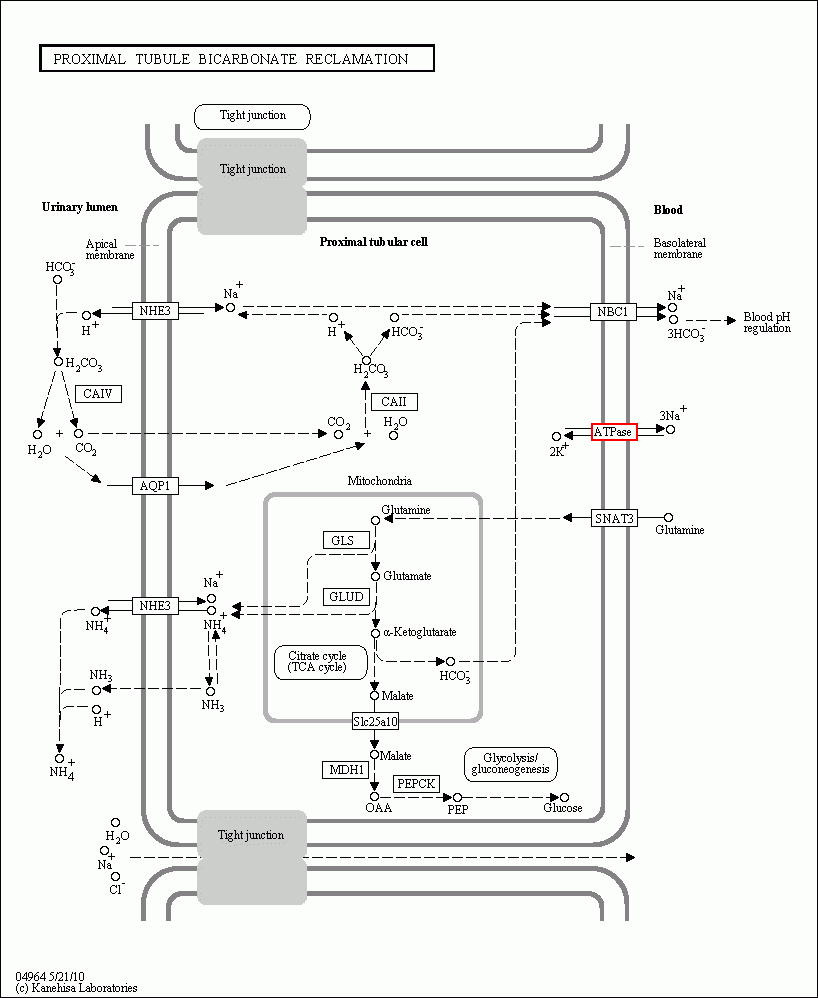

Supplement: S1 File — (ZIP) [file pone.0143219.s003.zip › pathway map/73 map04964.png]

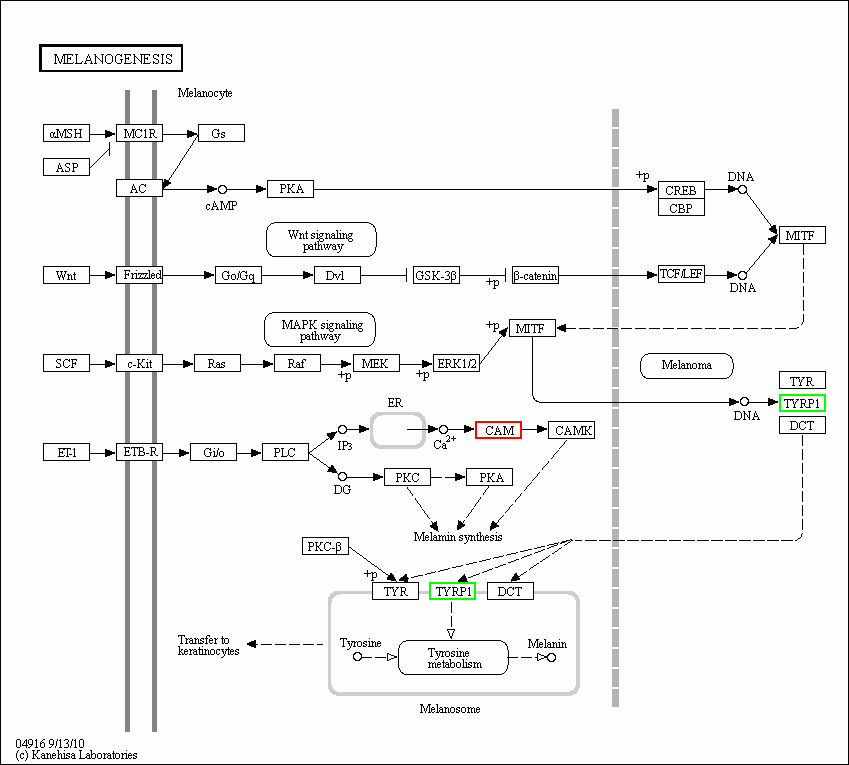

Supplement: S1 File — (ZIP) [file pone.0143219.s003.zip › pathway map/74 map04916.png]

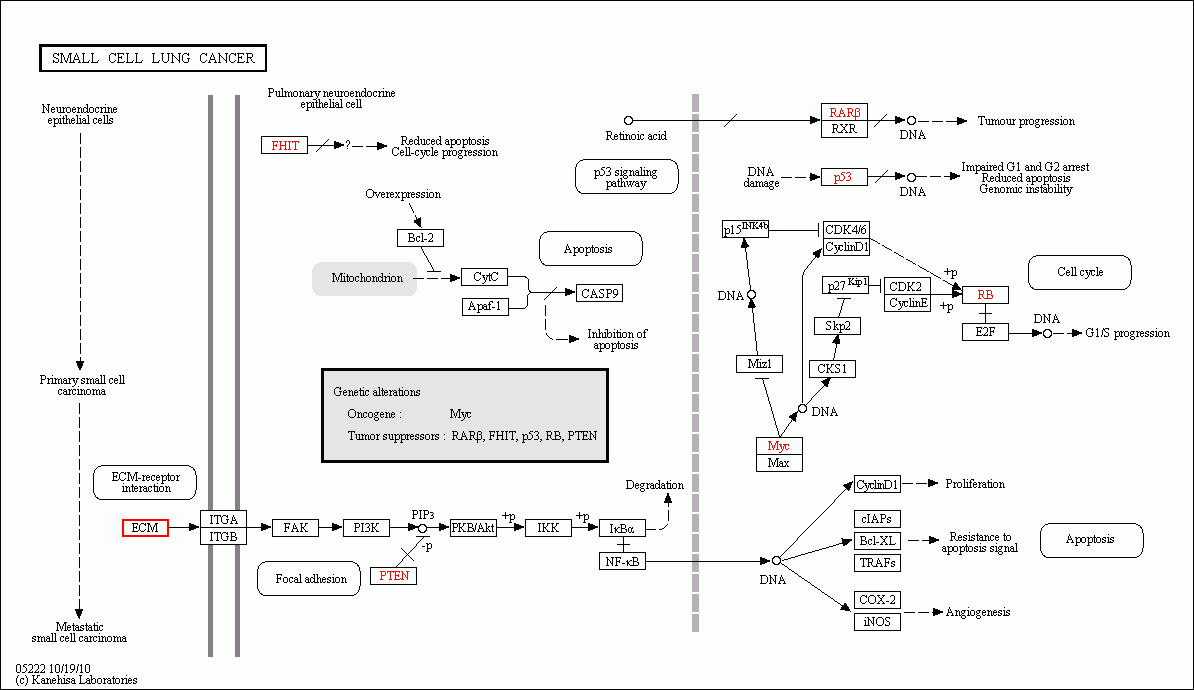

Supplement: S1 File — (ZIP) [file pone.0143219.s003.zip › pathway map/75 map05222.png]

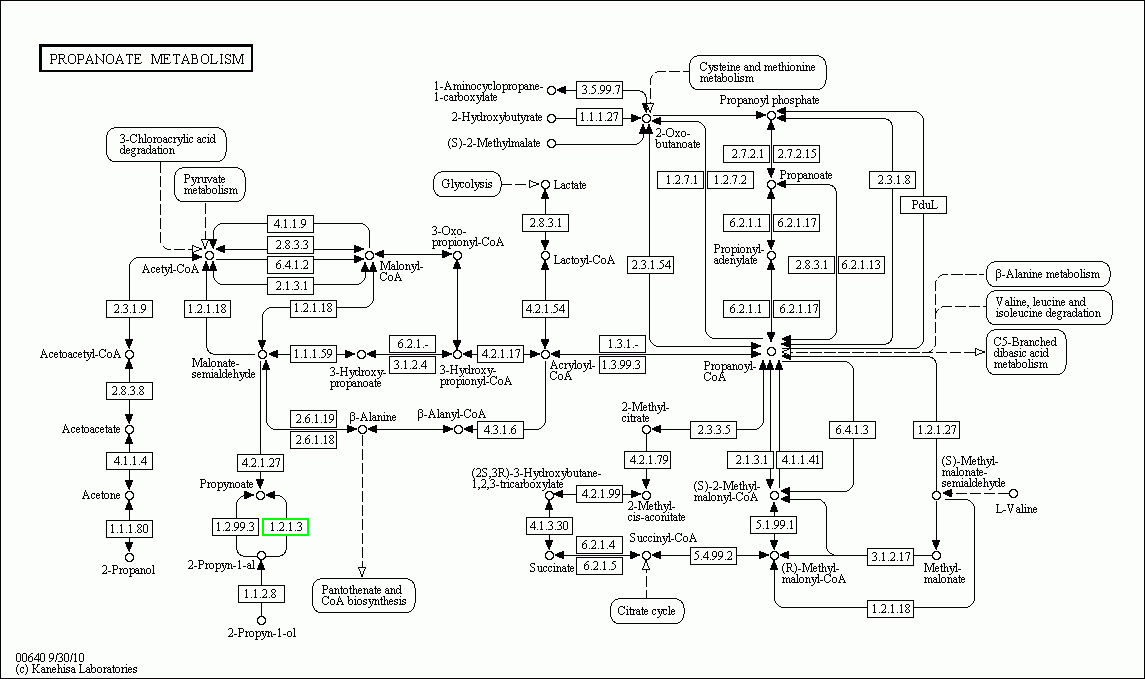

Supplement: S1 File — (ZIP) [file pone.0143219.s003.zip › pathway map/76 map00640.png]

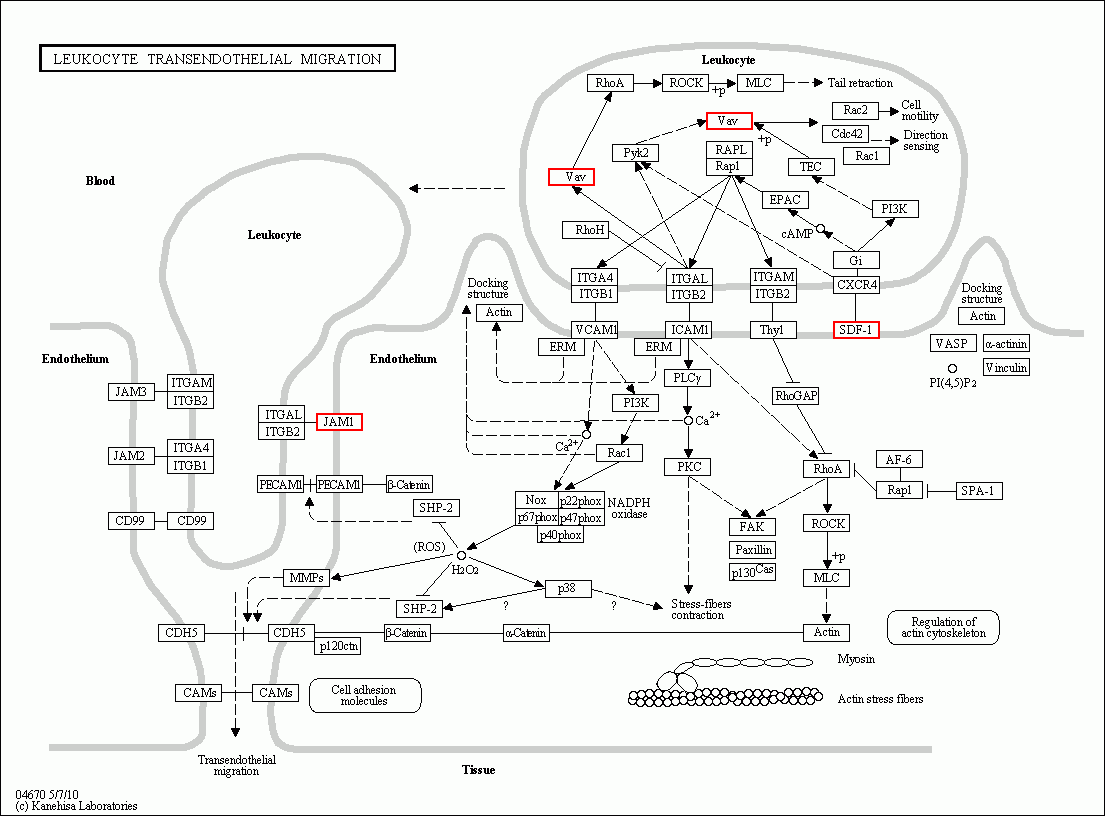

Supplement: S1 File — (ZIP) [file pone.0143219.s003.zip › pathway map/77 map04670.png]

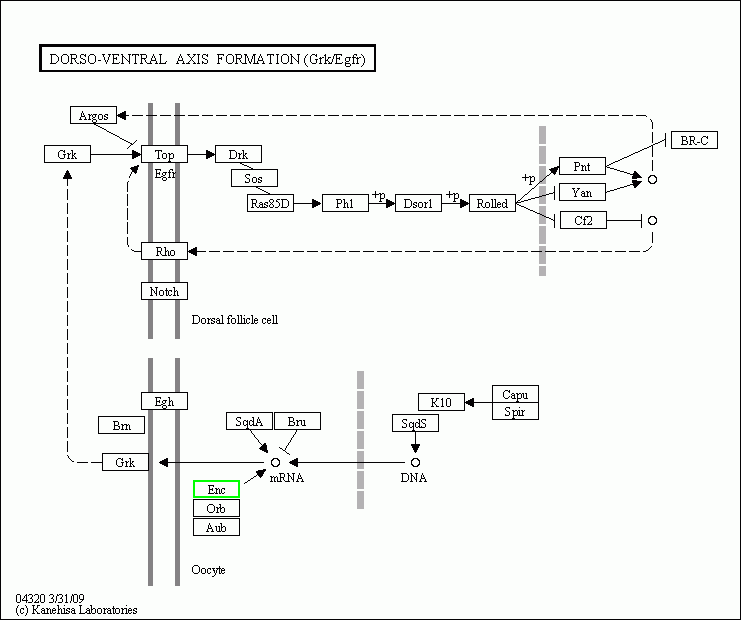

Supplement: S1 File — (ZIP) [file pone.0143219.s003.zip › pathway map/78 map04320.png]

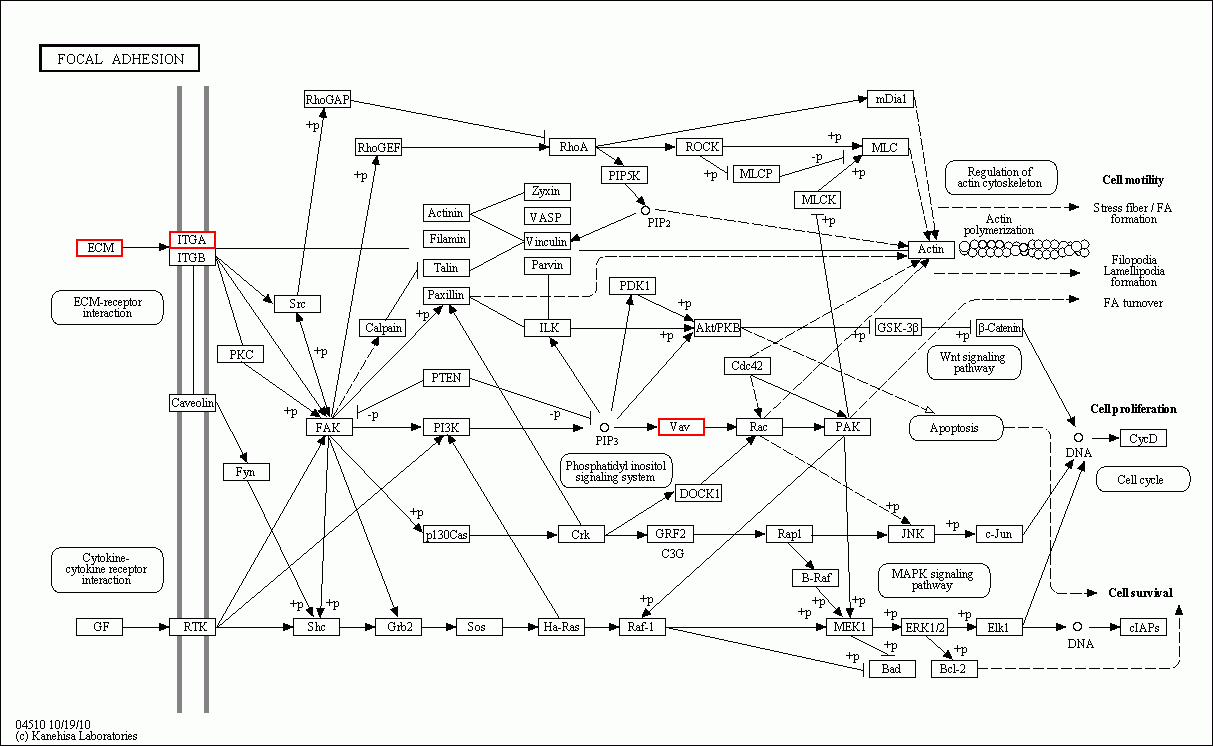

Supplement: S1 File — (ZIP) [file pone.0143219.s003.zip › pathway map/79 map04510.png]

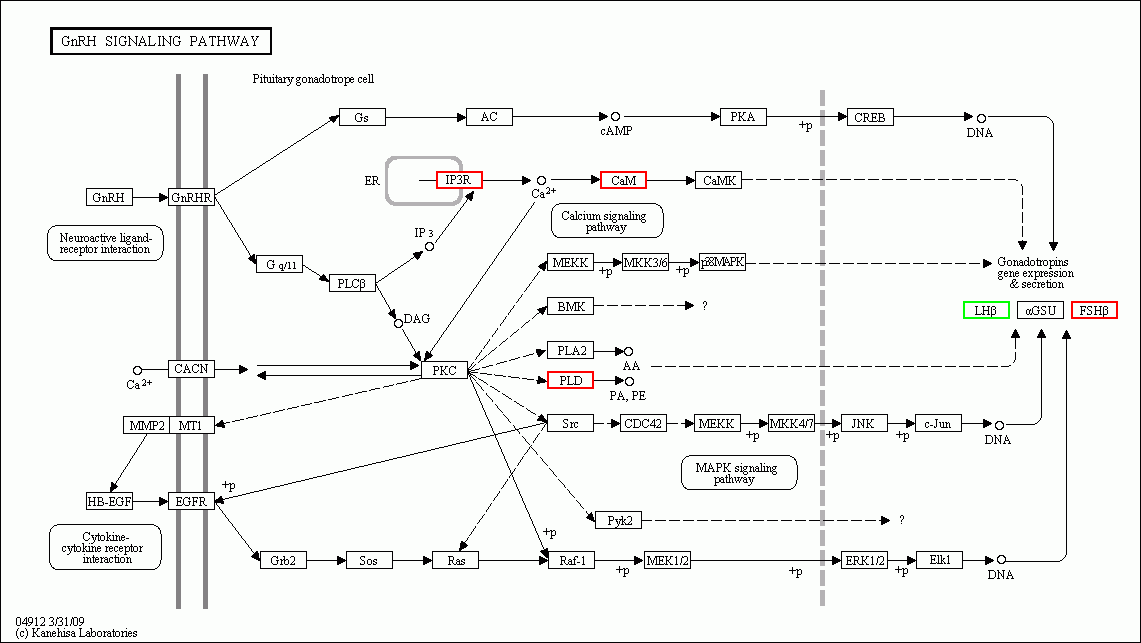

Supplement: S1 File — (ZIP) [file pone.0143219.s003.zip › pathway map/8 map04912.png]

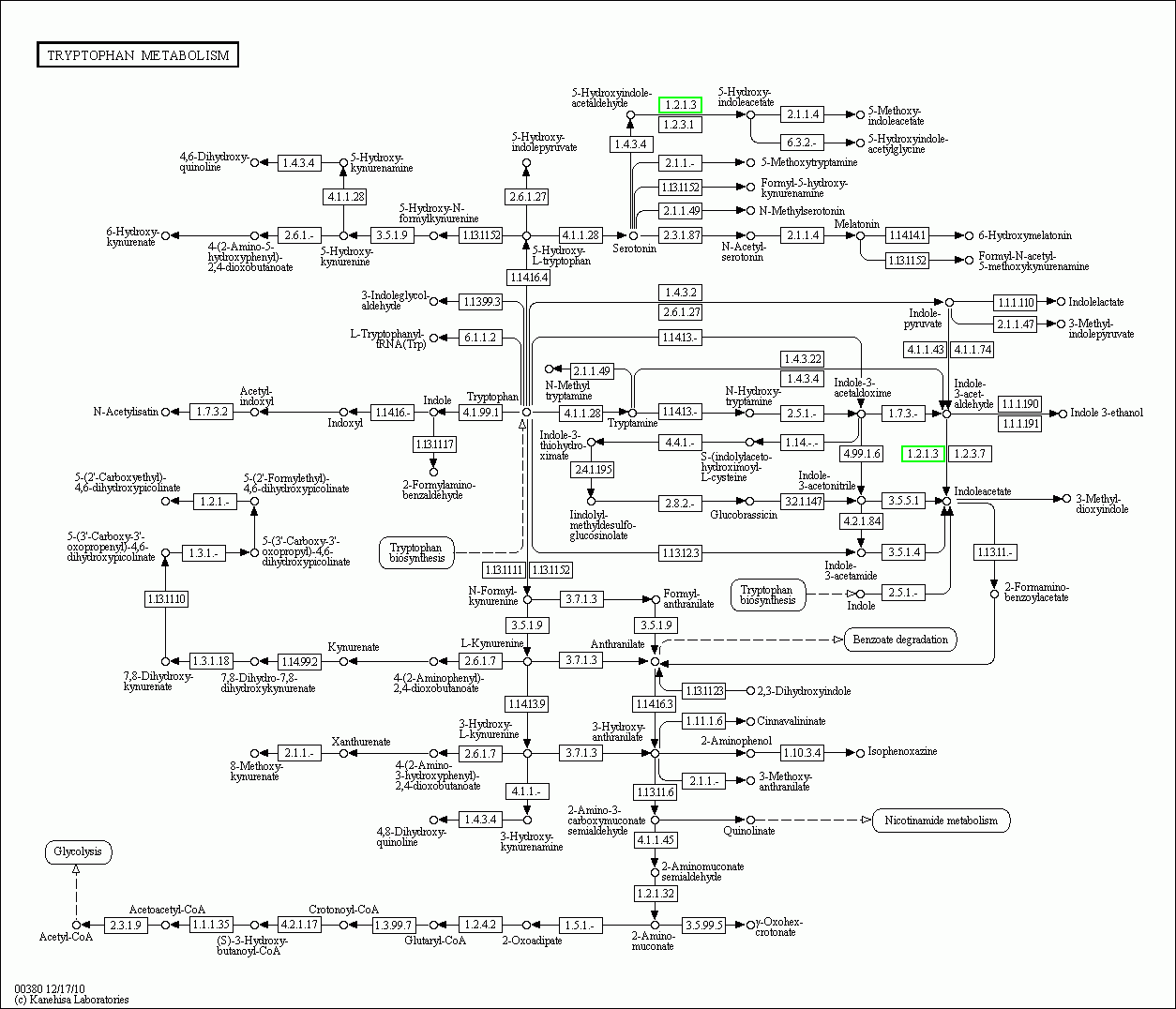

Supplement: S1 File — (ZIP) [file pone.0143219.s003.zip › pathway map/80 map00380.png]

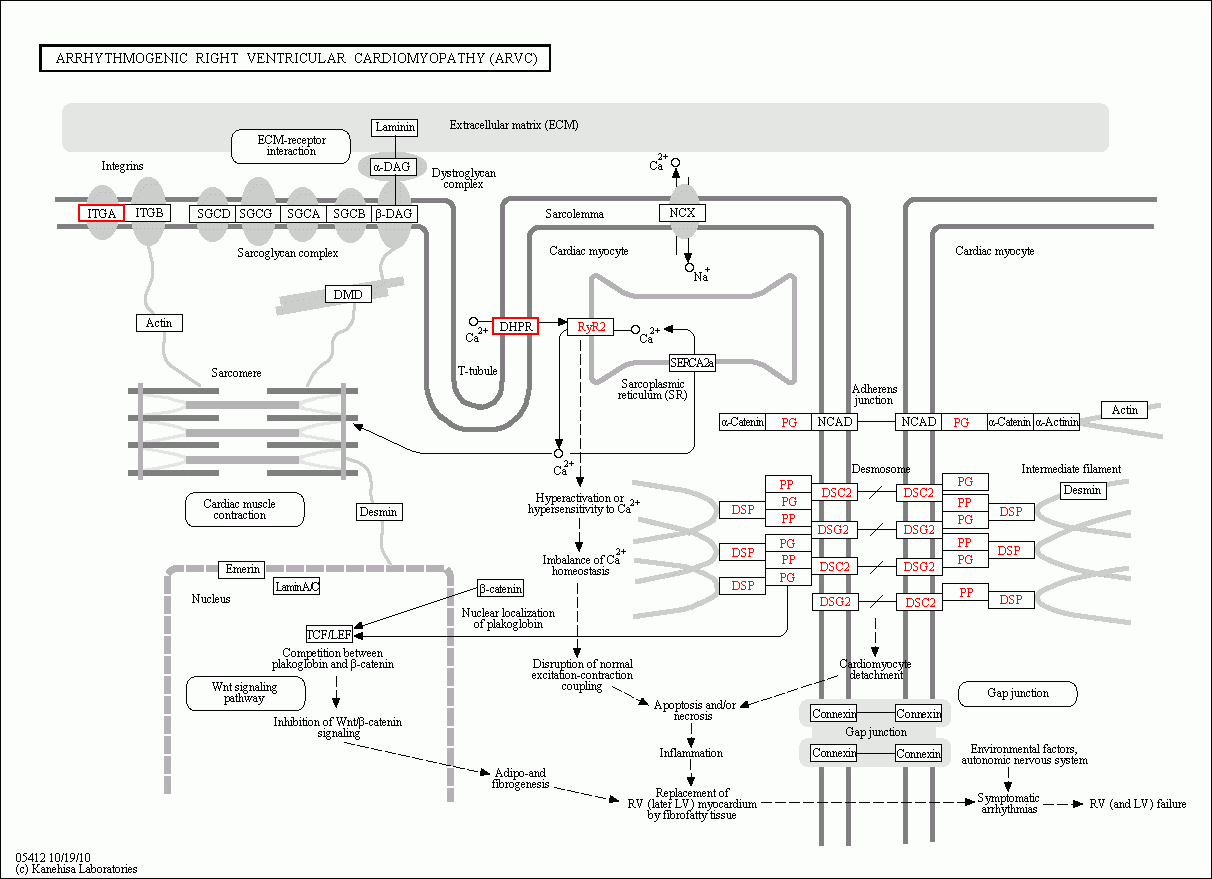

Supplement: S1 File — (ZIP) [file pone.0143219.s003.zip › pathway map/81 map05412.png]

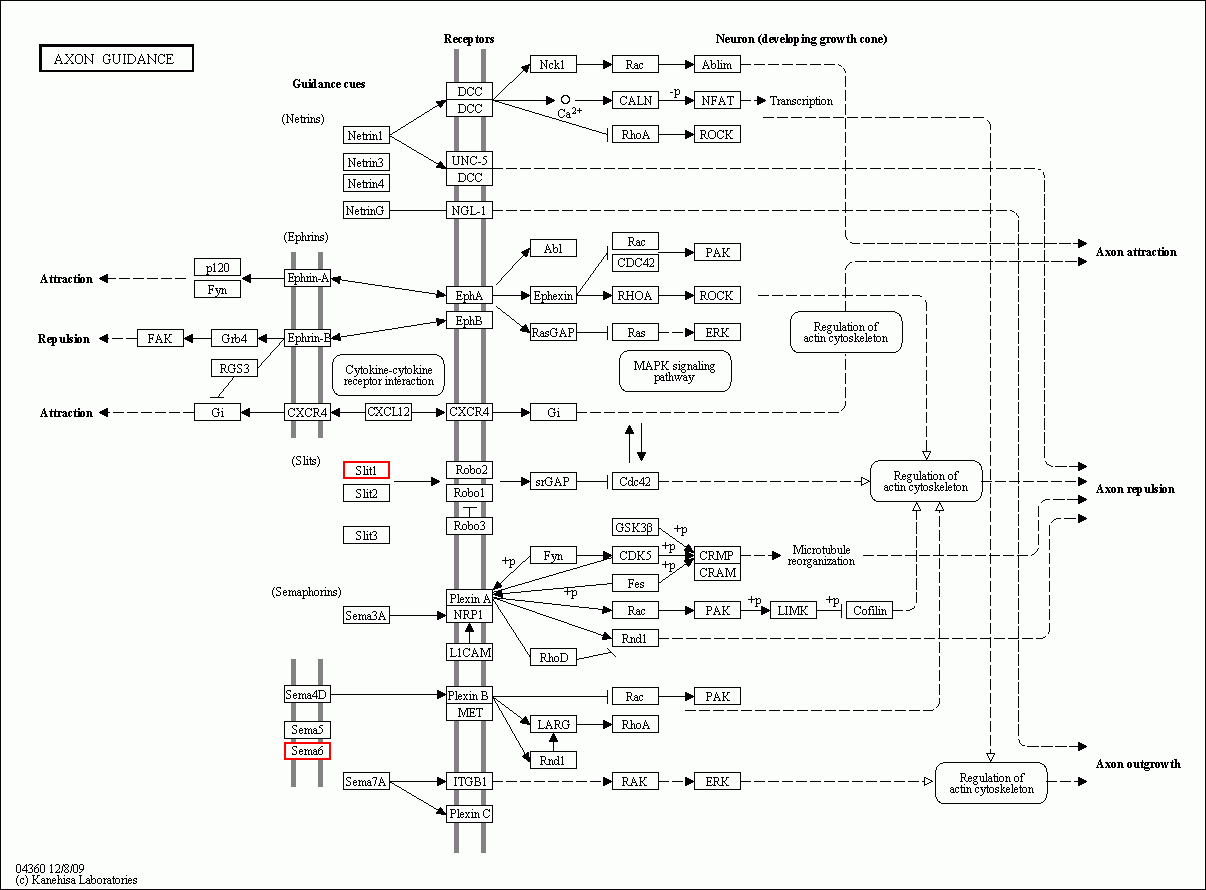

Supplement: S1 File — (ZIP) [file pone.0143219.s003.zip › pathway map/82 map04360.png]

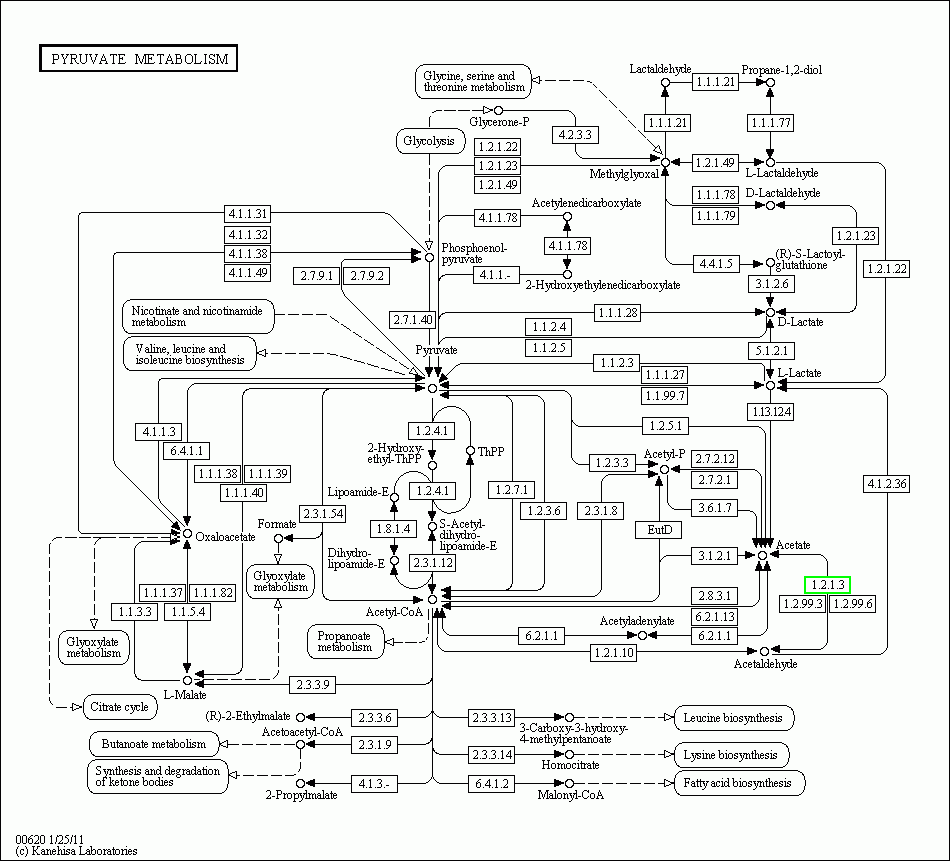

Supplement: S1 File — (ZIP) [file pone.0143219.s003.zip › pathway map/83 map00620.png]

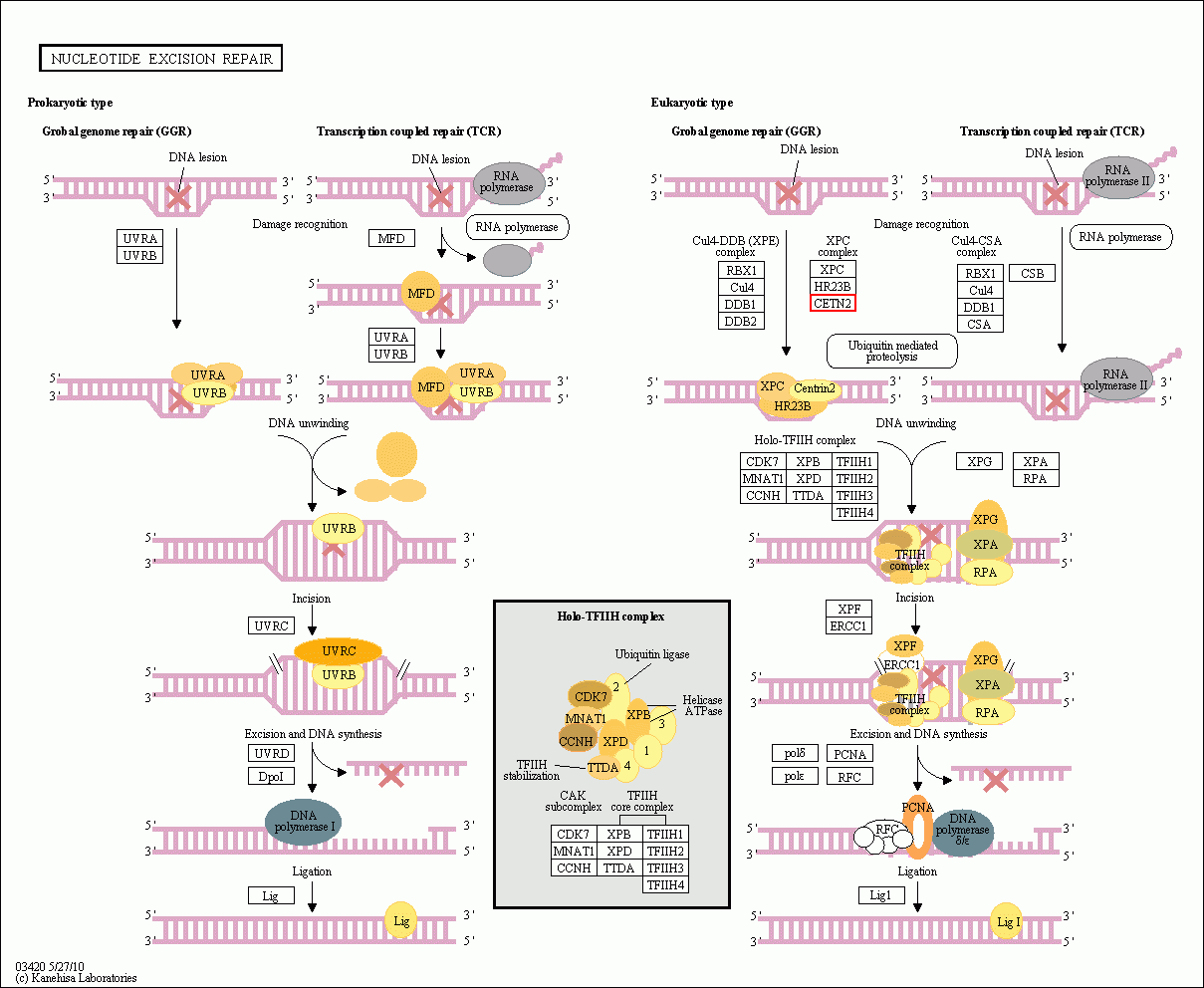

Supplement: S1 File — (ZIP) [file pone.0143219.s003.zip › pathway map/84 map03420.png]

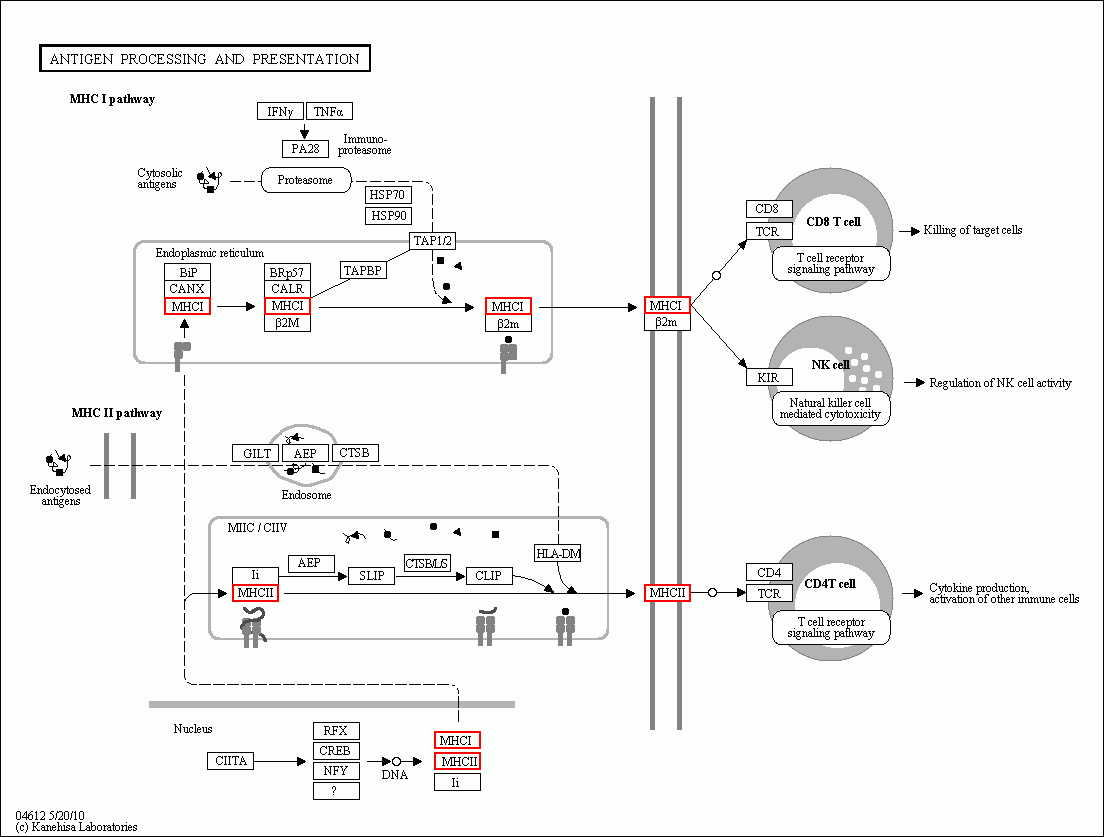

Supplement: S1 File — (ZIP) [file pone.0143219.s003.zip › pathway map/85 map04612.png]

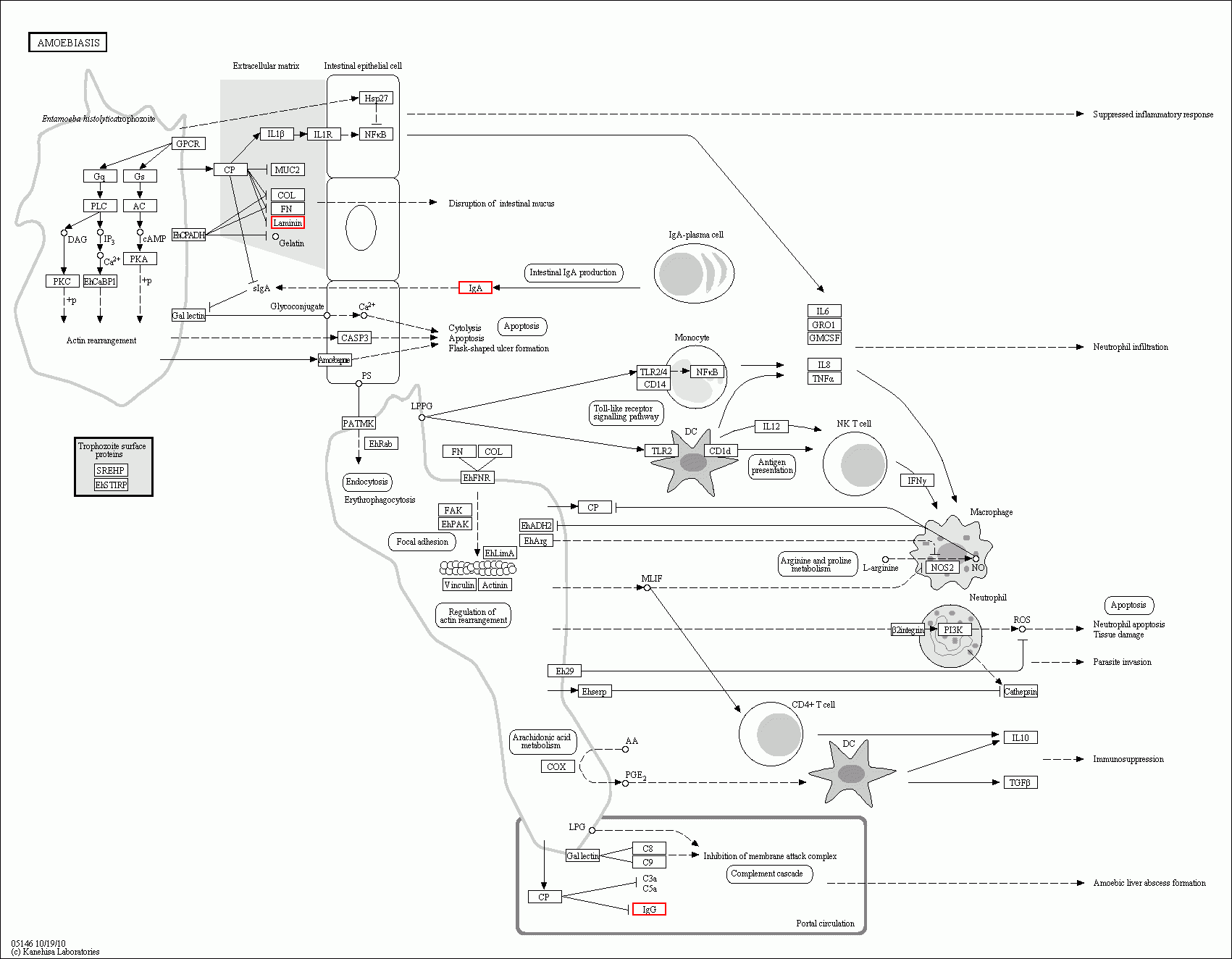

Supplement: S1 File — (ZIP) [file pone.0143219.s003.zip › pathway map/87 map05146.png]

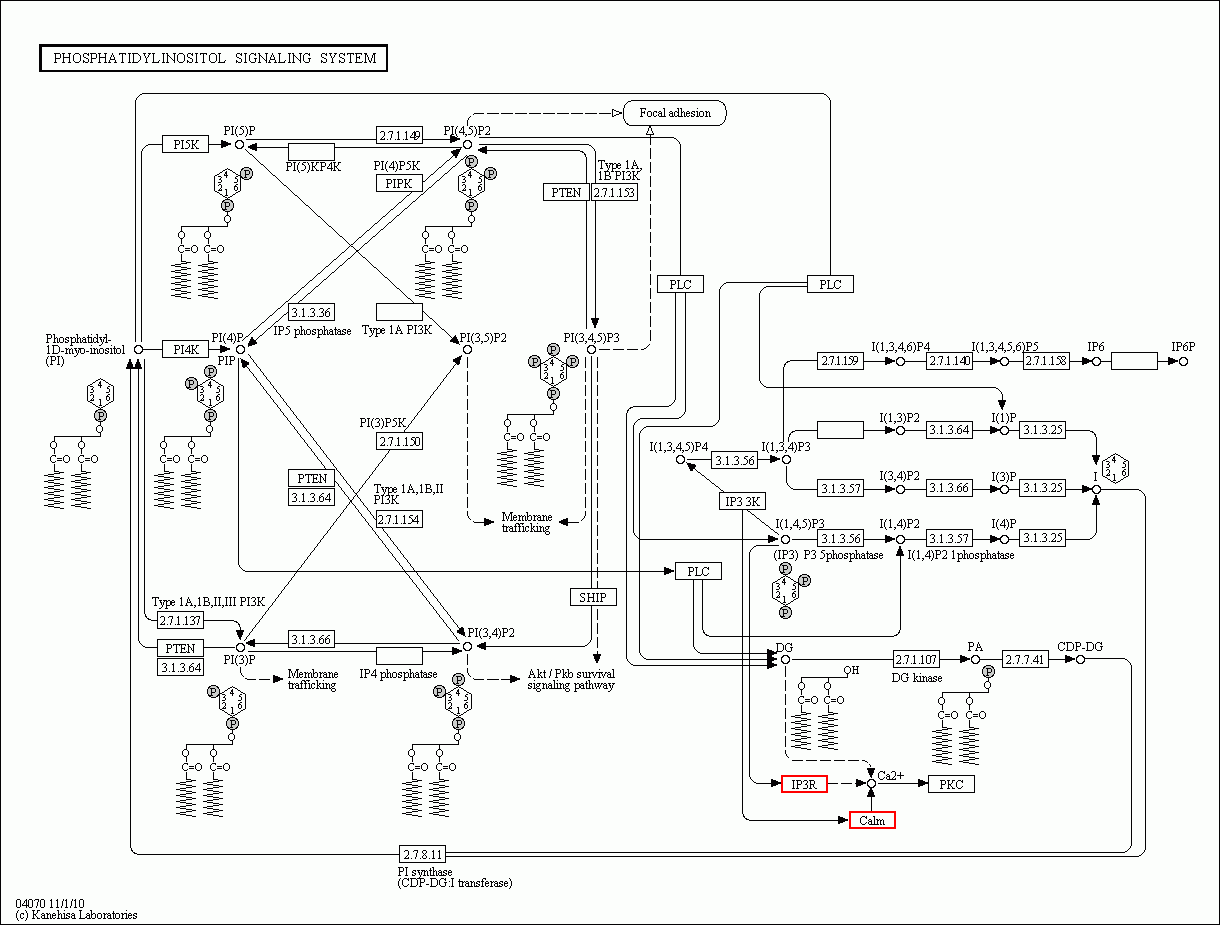

Supplement: S1 File — (ZIP) [file pone.0143219.s003.zip › pathway map/88 map04070.png]

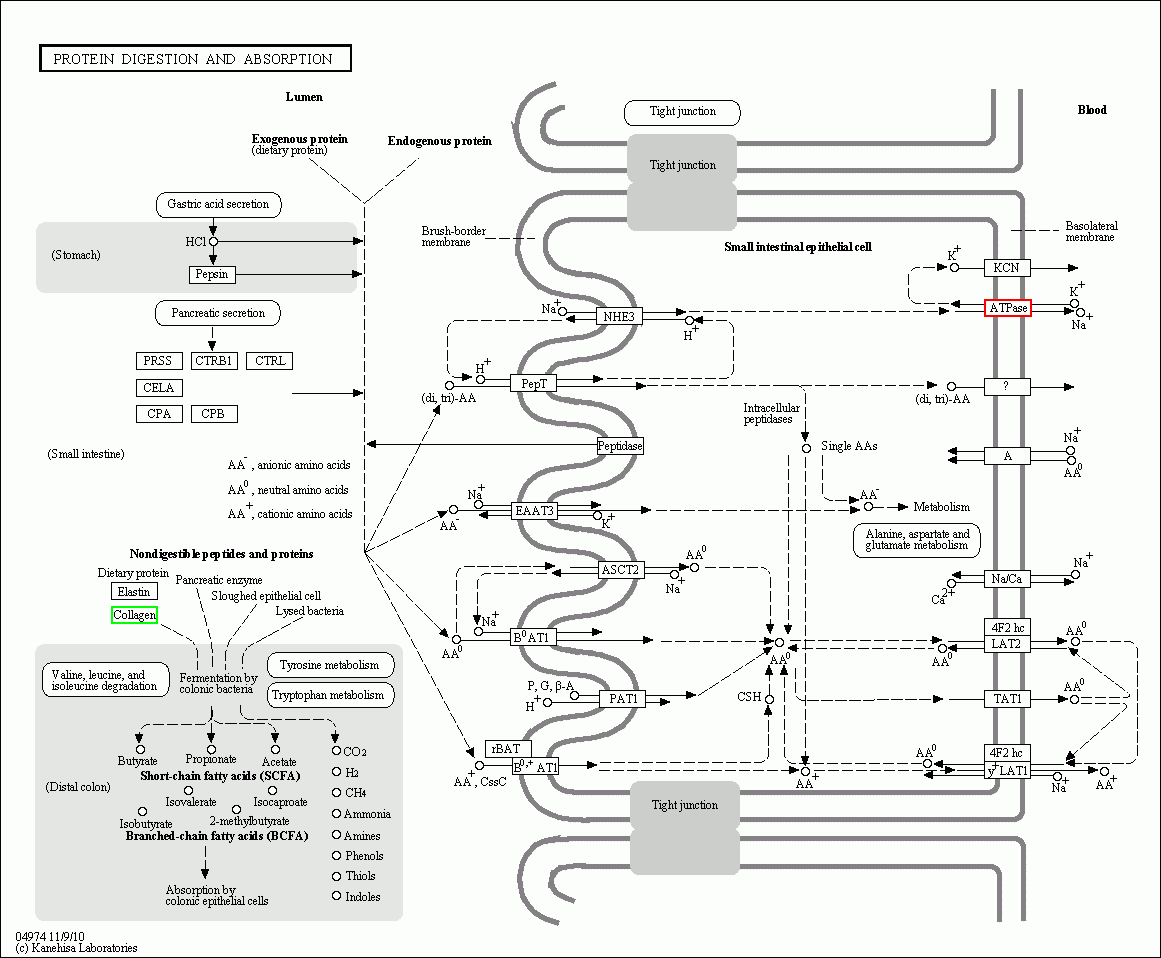

Supplement: S1 File — (ZIP) [file pone.0143219.s003.zip › pathway map/89 map04974.png]

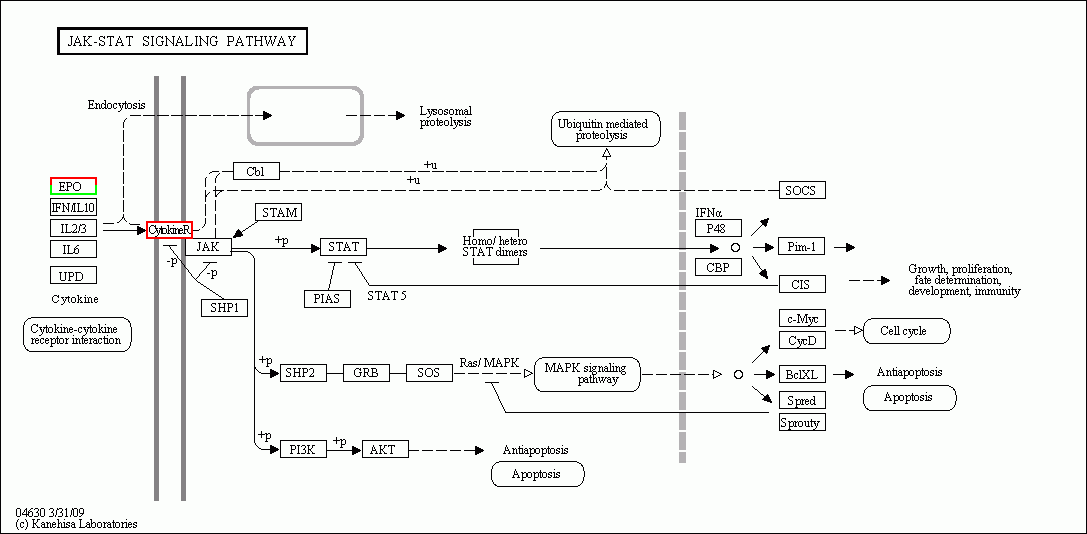

Supplement: S1 File — (ZIP) [file pone.0143219.s003.zip › pathway map/9 map04630.png]

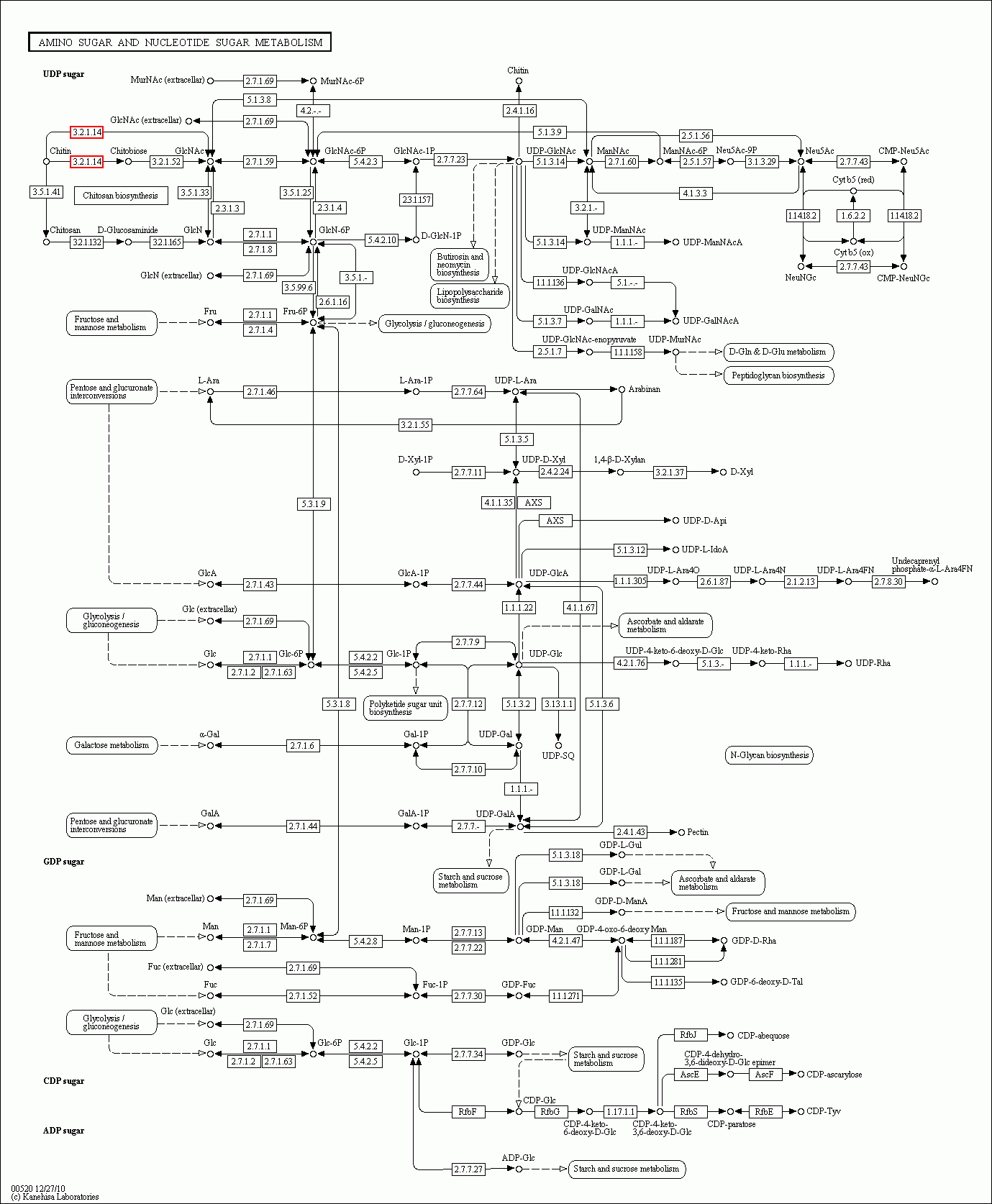

Supplement: S1 File — (ZIP) [file pone.0143219.s003.zip › pathway map/90 map00520.png]

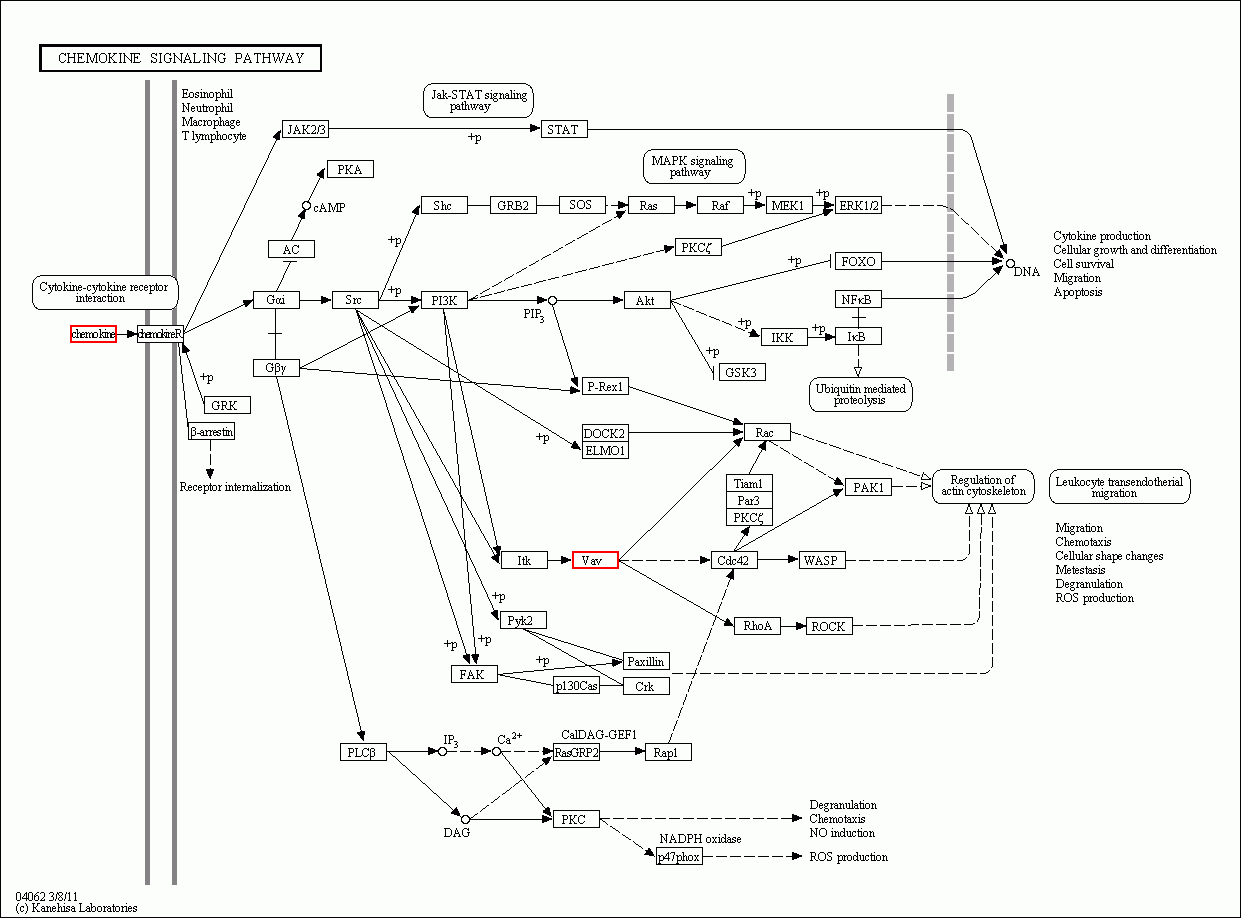

Supplement: S1 File — (ZIP) [file pone.0143219.s003.zip › pathway map/91 map04062.png]

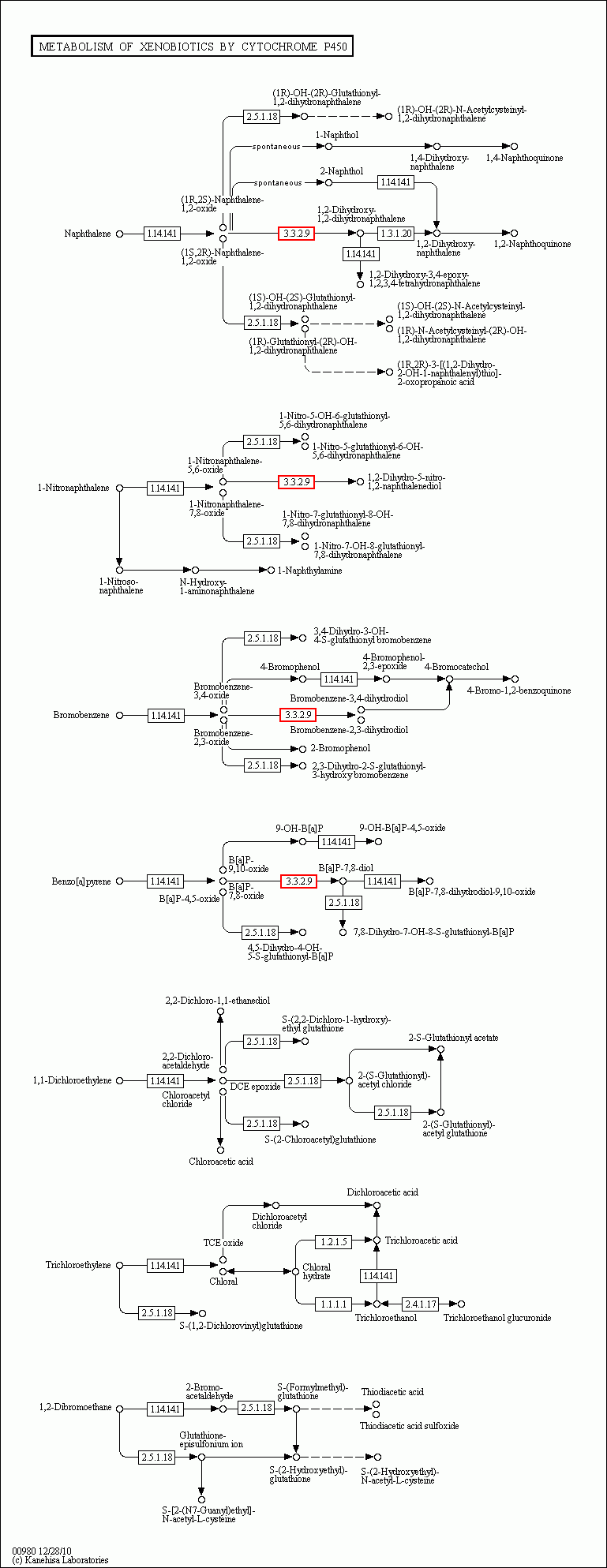

Supplement: S1 File — (ZIP) [file pone.0143219.s003.zip › pathway map/92 map00980.png]

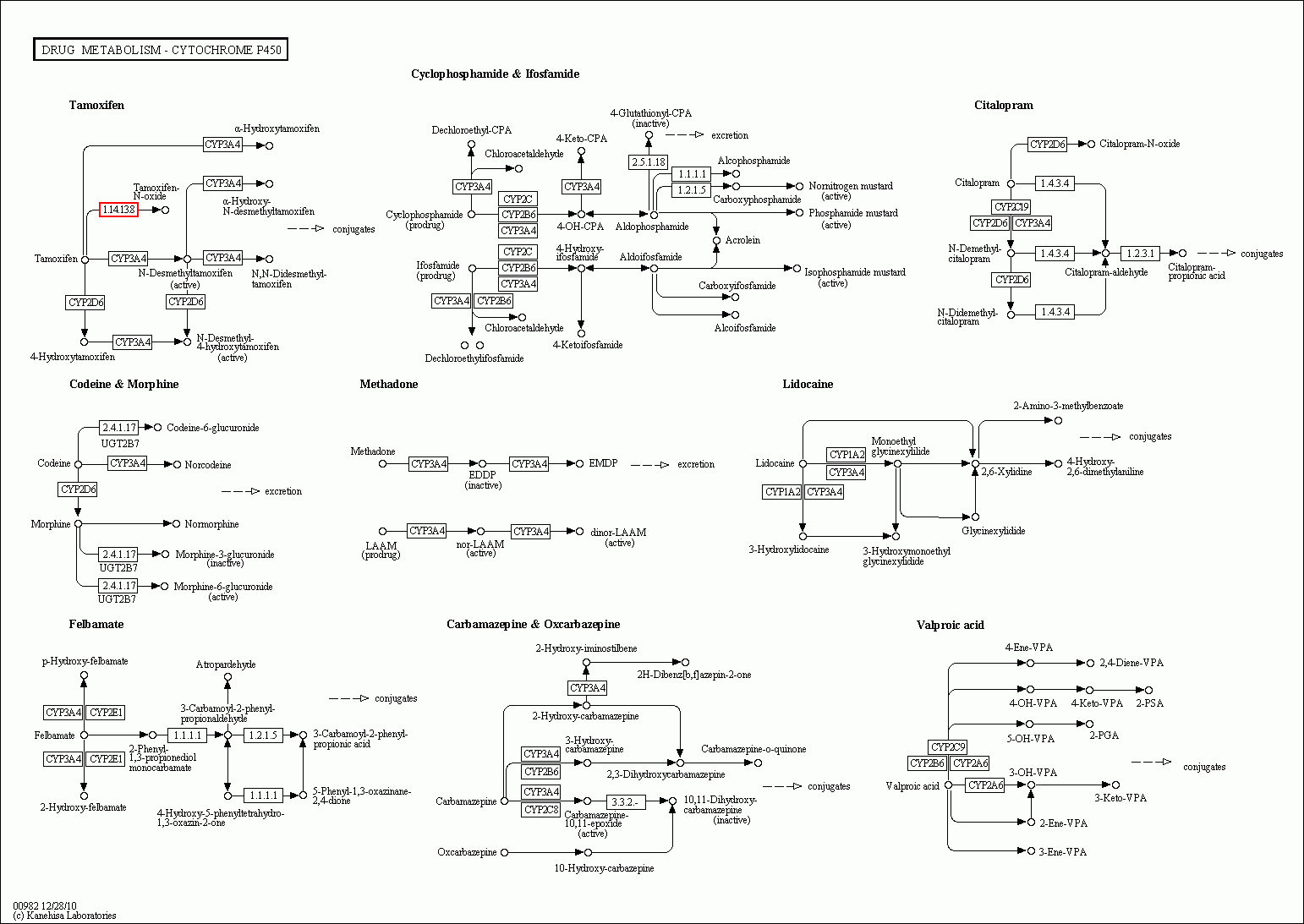

Supplement: S1 File — (ZIP) [file pone.0143219.s003.zip › pathway map/93 map00982.png]

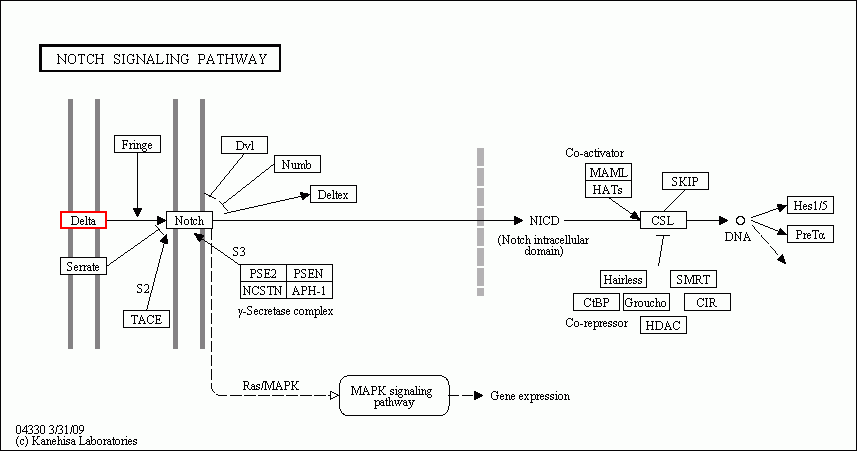

Supplement: S1 File — (ZIP) [file pone.0143219.s003.zip › pathway map/94 map04330.png]

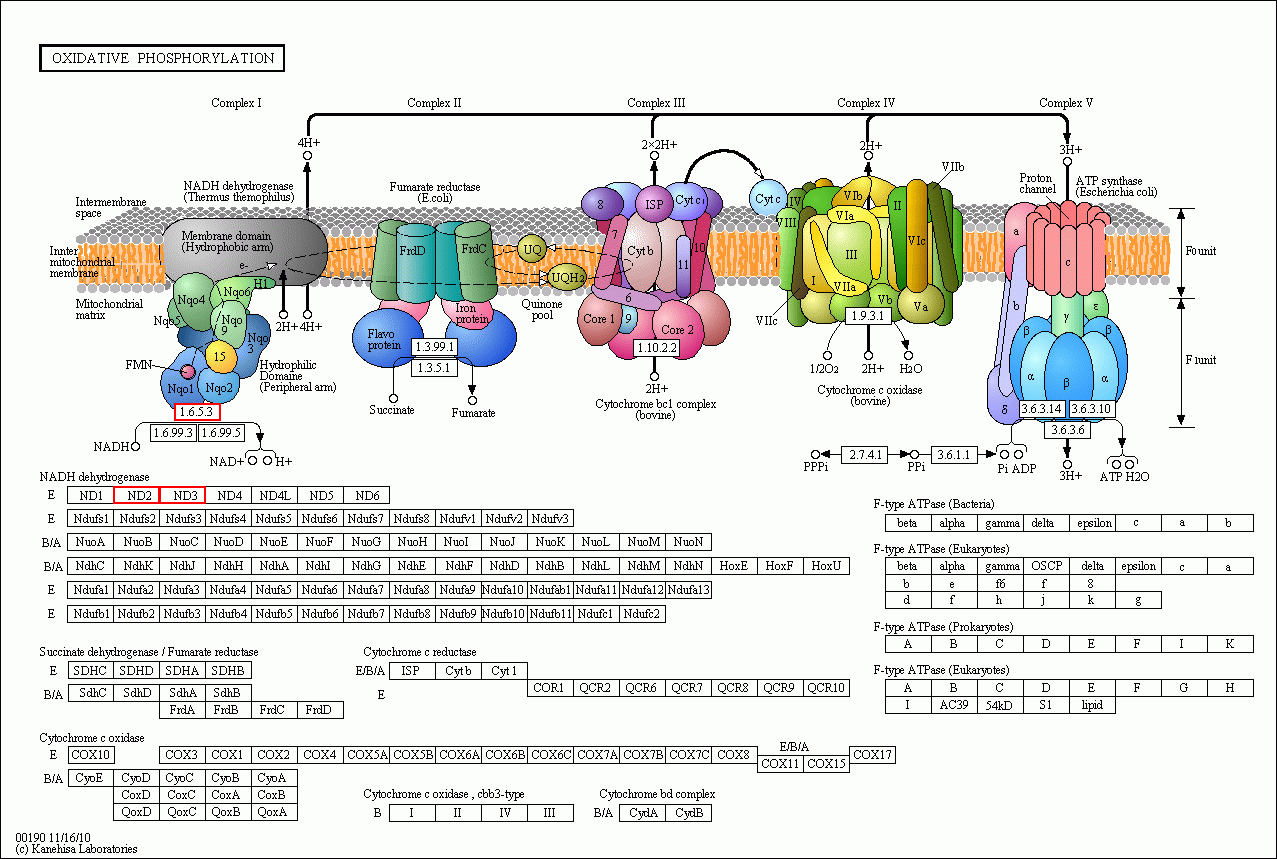

Supplement: S1 File — (ZIP) [file pone.0143219.s003.zip › pathway map/95 map00190.png]

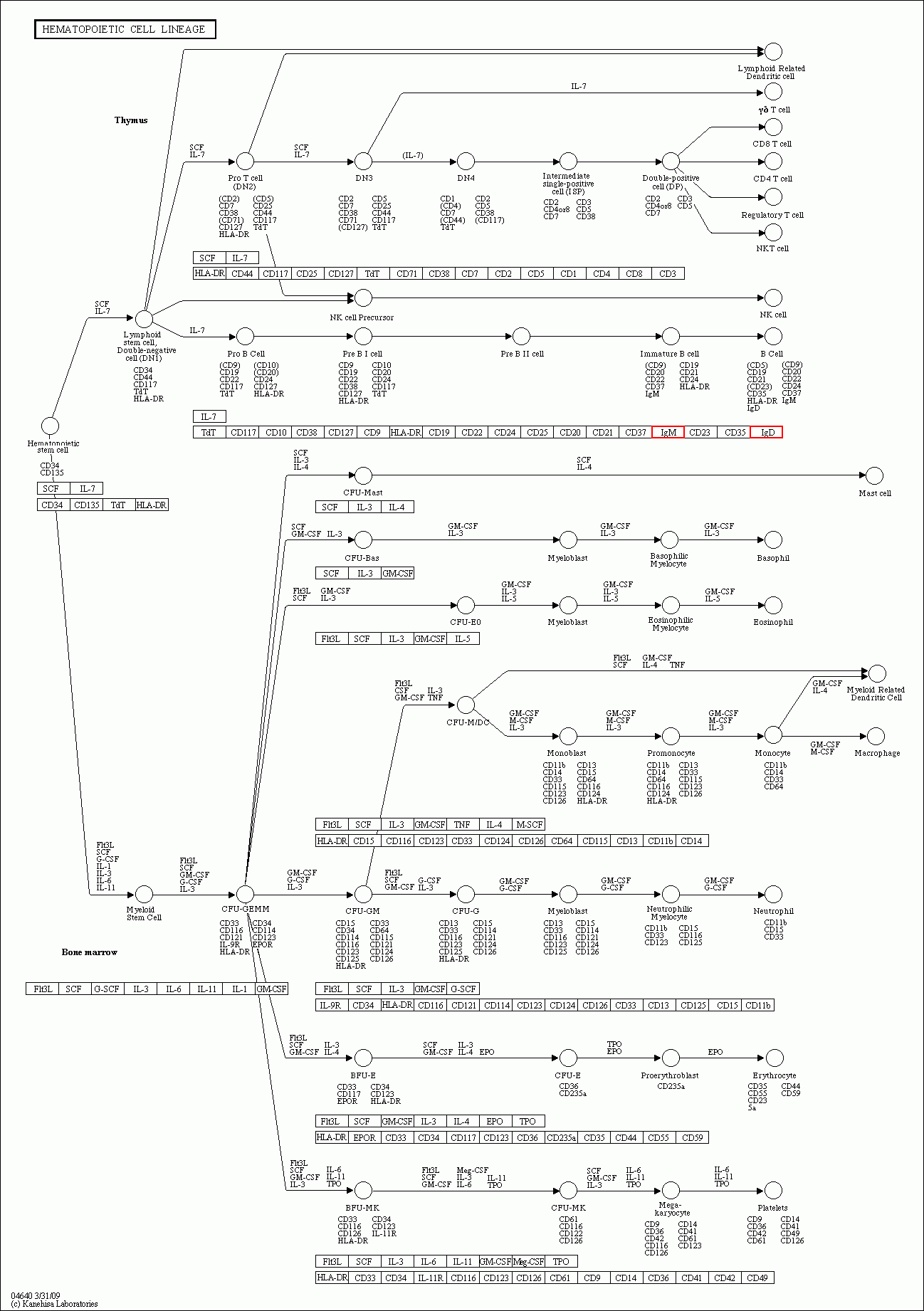

Supplement: S1 File — (ZIP) [file pone.0143219.s003.zip › pathway map/96 map04640.png]

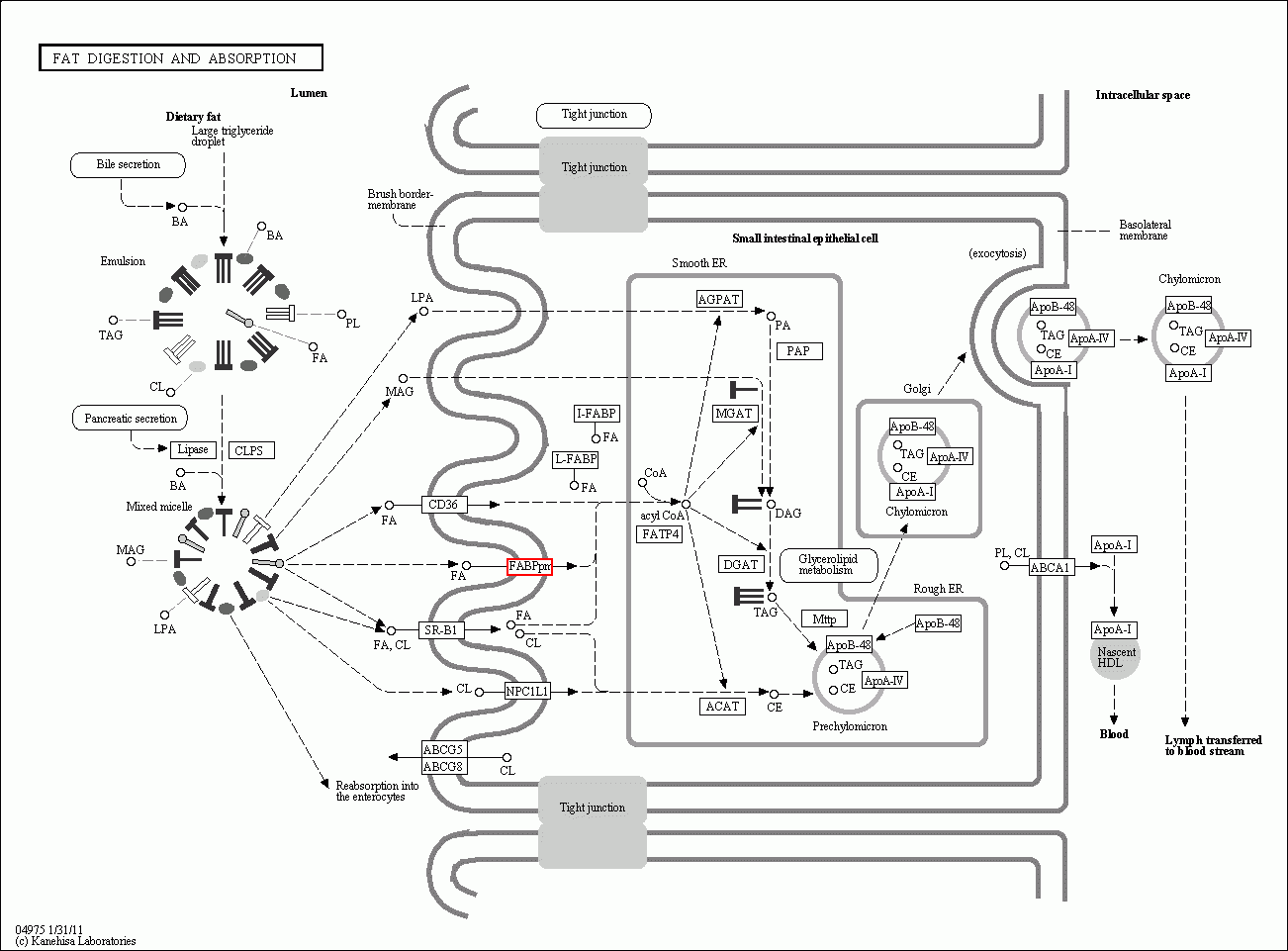

Supplement: S1 File — (ZIP) [file pone.0143219.s003.zip › pathway map/97 map04975.png]

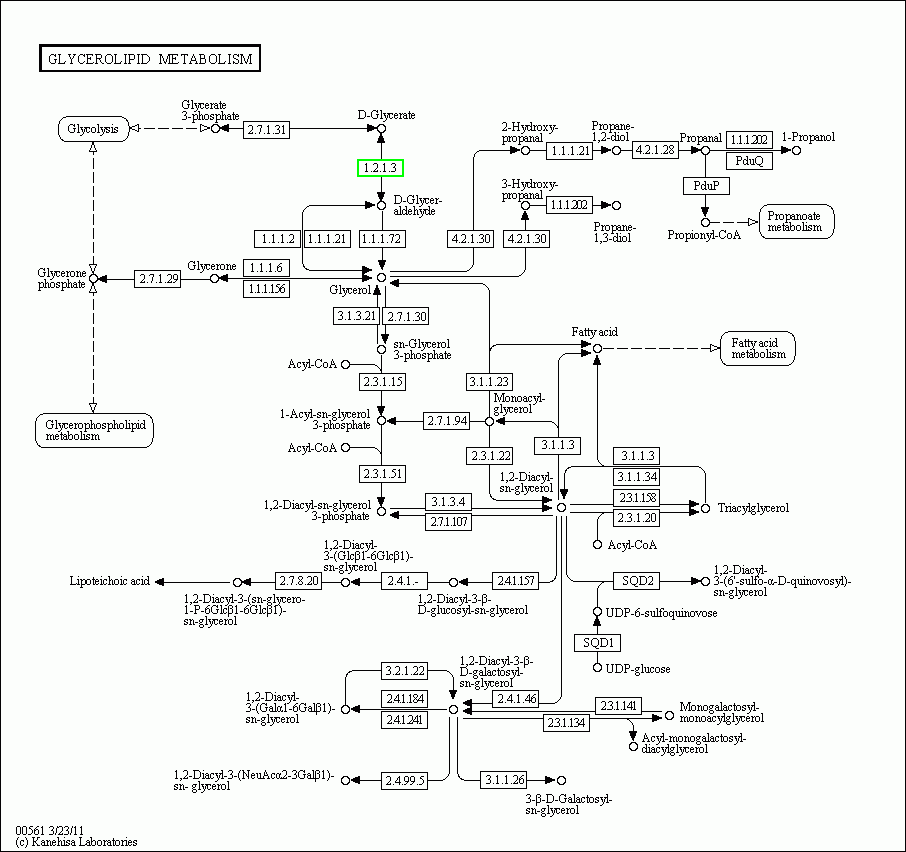

Supplement: S1 File — (ZIP) [file pone.0143219.s003.zip › pathway map/98 map00561.png]

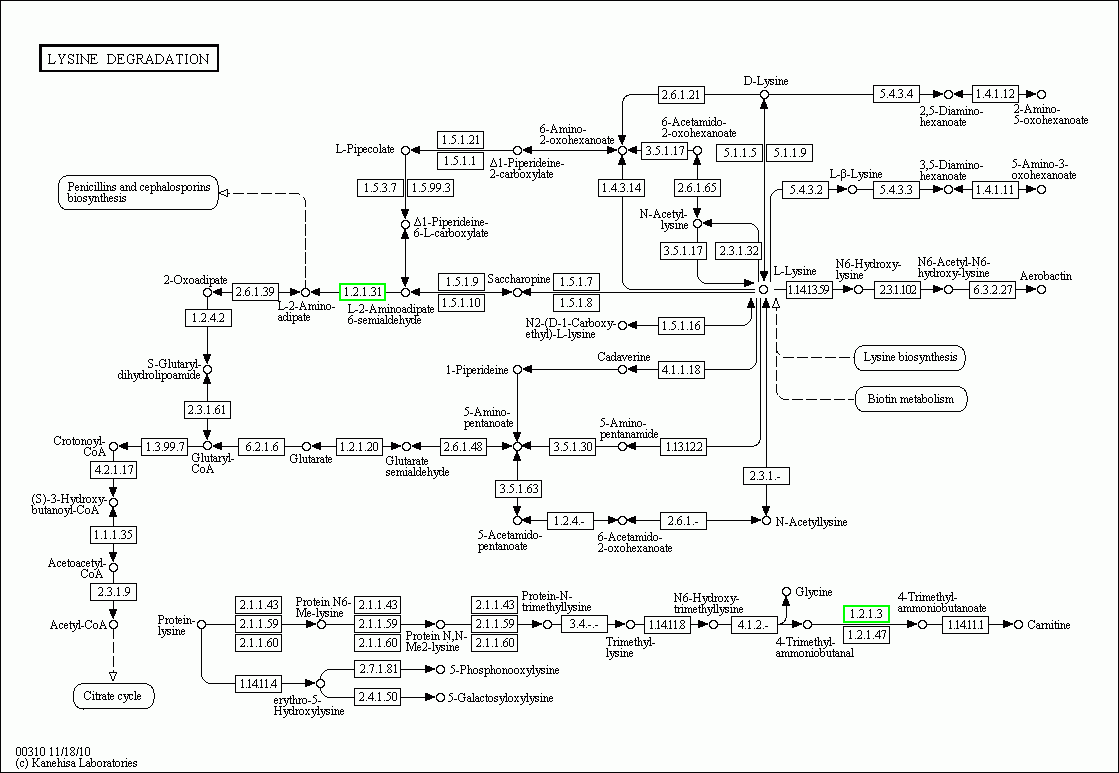

Supplement: S1 File — (ZIP) [file pone.0143219.s003.zip › pathway map/99 map00310.png]
