# Supplementary material for: Reproductive Hormone and Transcriptomic Responses of Pituitary Tissue in Anestrus Gilts Induced by Nutrient Restriction
Source: PLoS One. 2015 Nov 18;10(11):e0143219. doi: 10.1371/journal.pone.0143219 (PMC4651501; doi:10.1371/journal.pone.0143219)
Supplement: S4 Table — The Genes shown in red box were upregulated and those shown in green box were downregulated in CON relative to NR gilts in the hyperlink pathway figure. CON denotes normally fed gilts (2.86 kg/d) and NR denotes nutrient restricted gilts (1 kg/d). (DOCX) [file pone.0143219.s007.docx]

**S4 Table. The result of Solexa/Illumina sequencing for pathway analysis of differentially expressed genes based on the Kyoto Encyclopedia of Genes and Genomes (KEGG) database.**

|  | Pathway | **Differentially expressed genes** |
| --- | --- | --- |
| 1 | [Neuroactive ligand-receptor interaction](pathway%20map/1%20map04080.png) | gnl\|UG\|Ssc#S55325311, gnl\|UG\|Ssc#S55285749, gnl\|UG\|Ssc#S55325496, gi\|47523119\|ref\|NM_213869.1\|, gnl\|UG\|Ssc#S46876395, gnl\|UG\|Ssc#S55325364, gnl\|UG\|Ssc#S20116300, gnl\|UG\|Ssc#S6090246, gi\|47522633\|ref\|NM_213926.1\|, gnl\|UG\|Ssc#S55325522, gnl\|UG\|Ssc#S52891623 |
| 2 | [Complement and coagulation cascades](pathway%20map/2%20map04610.png) | gnl\|UG\|Ssc#S20116317, gnl\|UG\|Ssc#S58309878, gnl\|UG\|Ssc#S20116701, gnl\|UG\|Ssc#S6091069, gnl\|UG\|Ssc#S42951501, gnl\|UG\|Ssc#S18553219, gnl\|UG\|Ssc#S20116353 |
| 3 | [Prion diseases](pathway%20map/3%20map05020.png) | gnl\|UG\|Ssc#S20116317, gnl\|UG\|Ssc#S18553219, gnl\|UG\|Ssc#S58312433, gnl\|UG\|Ssc#S58312428 |
| 4 | [Systemic lupus erythematosus](pathway%20map/4%20map05322.png) | gnl\|UG\|Ssc#S55321680, gnl\|UG\|Ssc#S14766117, gnl\|UG\|Ssc#S20116317, gnl\|UG\|Ssc#S58309878, gnl\|UG\|Ssc#S20116701, gnl\|UG\|Ssc#S42951501, gnl\|UG\|Ssc#S18553219 |
| 5 | [Staphylococcus aureus infection](pathway%20map/5%20map05150.png) | gnl\|UG\|Ssc#S55321680, gnl\|UG\|Ssc#S14766117, gnl\|UG\|Ssc#S58309878, gnl\|UG\|Ssc#S20116701, gnl\|UG\|Ssc#S42951501, gnl\|UG\|Ssc#S18553219 |
| 6 | [Cytokine-cytokine receptor interaction](pathway%20map/6%20map04060.png) | gnl\|UG\|Ssc#S35171399, gnl\|UG\|Ssc#S55325496, gi\|47523119\|ref\|NM_213869.1\|, gnl\|UG\|Ssc#S58305733, gi\|47522633\|ref\|NM_213926.1\|, gnl\|UG\|Ssc#S55325522, gnl\|UG\|Ssc#S34273825 |
| 7 | [Salivary secretion](pathway%20map/7%20map04970.png) | gnl\|UG\|Ssc#S56448572, gnl\|UG\|Ssc#S55327620, gnl\|UG\|Ssc#S55283891, gnl\|UG\|Ssc#S58304058, gnl\|UG\|Ssc#S58300922 |
| 8 | [GnRH signaling pathway](pathway%20map/8%20map04912.png) | gnl\|UG\|Ssc#S56448572, gnl\|UG\|Ssc#S55325311, gnl\|UG\|Ssc#S58304058, gnl\|UG\|Ssc#S55318348, gnl\|UG\|Ssc#S52891623 |
| 9 | [Jak-STAT signaling pathway](pathway%20map/9%20map04630.png) | gnl\|UG\|Ssc#S55325496, gi\|47523119\|ref\|NM_213869.1\|, gnl\|UG\|Ssc#S58305733, gi\|47522633\|ref\|NM_213926.1\|, gnl\|UG\|Ssc#S55325522 |
| 10 | [Autoimmune thyroid disease](pathway%20map/10%20map05320.png) | gnl\|UG\|Ssc#S55321680, gnl\|UG\|Ssc#S14766117, gnl\|UG\|Ssc#S16592156, gnl\|UG\|Ssc#S20116300, gnl\|UG\|Ssc#S34273825 |
| 11 | [ECM-receptor interaction](pathway%20map/11%20map04512.png) | gnl\|UG\|Ssc#S58310151, gnl\|UG\|Ssc#S58308537, gnl\|UG\|Ssc#S43495858, gnl\|UG\|Ssc#S46875662, gnl\|UG\|Ssc#S6091069 |
| 12 | [Taste transduction](pathway%20map/12%20map04742.png) | gnl\|UG\|Ssc#S58313056, gnl\|UG\|Ssc#S58309851 |
| 13 | [Lysine biosynthesis](pathway%20map/13%20map00300.png) | gnl\|UG\|Ssc#S35328054 |
| 14 | [Calcium signaling pathway](pathway%20map/14%20map04020.png) | gnl\|UG\|Ssc#S55321680, gnl\|UG\|Ssc#S56448572, gnl\|UG\|Ssc#S58313056, gnl\|UG\|Ssc#S46876395, gnl\|UG\|Ssc#S58304058, gnl\|UG\|Ssc#S58300922, gnl\|UG\|Ssc#S58305222 |
| 15 | [MAPK signaling pathway](pathway%20map/15%20map04010.png) | gnl\|UG\|Ssc#S58313056, gnl\|UG\|Ssc#S58314746, gnl\|UG\|Ssc#S55327735, gnl\|UG\|Ssc#S35166542, gnl\|UG\|Ssc#S55326563, gnl\|UG\|Ssc#S46879502, gnl\|UG\|Ssc#S34273825 |
| 16 | [Allograft rejection](pathway%20map/16%20map05330.png) | gnl\|UG\|Ssc#S55321680, gnl\|UG\|Ssc#S14766117, gnl\|UG\|Ssc#S16592156, gnl\|UG\|Ssc#S34273825 |
| 17 | [Leishmaniasis](pathway%20map/17%20map05140.png) | gnl\|UG\|Ssc#S55321680, gnl\|UG\|Ssc#S14766117, gnl\|UG\|Ssc#S20116701, gnl\|UG\|Ssc#S35166542 |
| 18 | [Graft-versus-host disease](pathway%20map/18%20map05332.png) | gnl\|UG\|Ssc#S14766117, gnl\|UG\|Ssc#S16592156, gnl\|UG\|Ssc#S34273825 |
| 19 | [Chagas disease (American trypanosomiasis)](pathway%20map/19%20map05142.png) | gnl\|UG\|Ssc#S20116701, gnl\|UG\|Ssc#S18553219, gnl\|UG\|Ssc#S35166542, gnl\|UG\|Ssc#S34273825 |
| 20 | [Cysteine and methionine metabolism](pathway%20map/20%20map00270.png) | gnl\|UG\|Ssc#S56448572, gnl\|UG\|Ssc#S46879456 |
| 21 | [Phenylalanine, tyrosine and tryptophan biosynthesis](pathway%20map/21%20map00400.png) | gnl\|UG\|Ssc#S56448572 |
| 22 | [Phototransduction](pathway%20map/22%20map04744.png) | gnl\|UG\|Ssc#S56448572, gnl\|UG\|Ssc#S58312740 |
| 23 | [Type I diabetes mellitus](pathway%20map/23%20map04940.png) | gnl\|UG\|Ssc#S14766117, gnl\|UG\|Ssc#S16592156, gnl\|UG\|Ssc#S34273825 |
| 24 | [Rheumatoid arthritis](pathway%20map/24%20map05323.png) | gnl\|UG\|Ssc#S55321680, gnl\|UG\|Ssc#S14766117, gnl\|UG\|Ssc#S35171399, gnl\|UG\|Ssc#S35166542 |
| 25 | [Fc gamma R-mediated phagocytosis](pathway%20map/25%20map04666.png) | gnl\|UG\|Ssc#S55321680, gnl\|UG\|Ssc#S58306512, gnl\|UG\|Ssc#S55318348, gnl\|UG\|Ssc#S46881169, gnl\|UG\|Ssc#S58305222 |
| 26 | [Phagosome](pathway%20map/26%20map04145.png) | gnl\|UG\|Ssc#S55321680, gnl\|UG\|Ssc#S14766117, gnl\|UG\|Ssc#S16592156, gnl\|UG\|Ssc#S35167297, gnl\|UG\|Ssc#S58309878, gnl\|UG\|Ssc#S20116701 |
| 27 | [Long-term potentiation](pathway%20map/27%20map04720.png) | gnl\|UG\|Ssc#S56448572, gnl\|UG\|Ssc#S58304058, gnl\|UG\|Ssc#S18357620 |
| 28 | [Sulfur relay system](pathway%20map/28%20map04122.png) | gnl\|UG\|Ssc#S46879456 |
| 29 | [Fatty acid metabolism](pathway%20map/29%20map00071.png) | gnl\|UG\|Ssc#S35328054, gnl\|UG\|Ssc#S55325470 |
| 30 | [Renin-angiotensin system](pathway%20map/30%20map04614.png) | gnl\|UG\|Ssc#S46876867 |
| 31 | [Ether lipid metabolism](pathway%20map/31%20map00565.png) | gnl\|UG\|Ssc#S55318348, gnl\|UG\|Ssc#S23212902 |
| 32 | [B cell receptor signaling pathway](pathway%20map/32%20map04662.png) | gnl\|UG\|Ssc#S55321680, gnl\|UG\|Ssc#S58306512, gnl\|UG\|Ssc#S58312169, gnl\|UG\|Ssc#S35166542 |
| 33 | [Arginine and proline metabolism](pathway%20map/33%20map00330.png) | gnl\|UG\|Ssc#S56448572, gnl\|UG\|Ssc#S35328054 |
| 34 | [Primary immunodeficiency](pathway%20map/34%20map05340.png) | gnl\|UG\|Ssc#S55242440, gnl\|UG\|Ssc#S55321680, gnl\|UG\|Ssc#S35172157 |
| 35 | [Intestinal immune network for IgA production](pathway%20map/35%20map04672.png) | gnl\|UG\|Ssc#S55321680, gnl\|UG\|Ssc#S14766117, gnl\|UG\|Ssc#S35171399 |
| 36 | [Tyrosine metabolism](pathway%20map/36%20map00350.png) | gnl\|UG\|Ssc#S56448572, gnl\|UG\|Ssc#S25665854 |
| 37 | [Type II diabetes mellitus](pathway%20map/37%20map04930.png) | gnl\|UG\|Ssc#S58313056, gnl\|UG\|Ssc#S58313090 |
| 38 | [Ribosome](pathway%20map/38%20map03010.png) | gnl\|UG\|Ssc#S39884104, gnl\|UG\|Ssc#S35164089, gnl\|UG\|Ssc#S40379983, gnl\|UG\|Ssc#S35324522 |
| 39 | [Phototransduction - fly](pathway%20map/39%20map04745.png) | gnl\|UG\|Ssc#S56448572, gnl\|UG\|Ssc#S58305647 |
| 40 | [Aldosterone-regulated sodium reabsorption](pathway%20map/40%20map04960.png) | gnl\|UG\|Ssc#S55327620, gnl\|UG\|Ssc#S58309851 |
| 41 | [Cell adhesion molecules (CAMs)](pathway%20map/41%20map04514.png) | gnl\|UG\|Ssc#S14766117, gnl\|UG\|Ssc#S16592156, gnl\|UG\|Ssc#S58305017, gnl\|UG\|Ssc#S46875662 |
| 42 | Glycosaminoglycan biosynthesis - heparan sulfate (no map in kegg database) | gnl\|UG\|Ssc#S58304506 |
| 43 | [Ascorbate and aldarate metabolism](pathway%20map/43%20map00053.png) | gnl\|UG\|Ssc#S35328054 |
| 44 | [Gastric acid secretion](pathway%20map/44%20map04971.png) | gnl\|UG\|Ssc#S56448572, gnl\|UG\|Ssc#S55327620, gnl\|UG\|Ssc#S58304058 |
| 45 | [Oocyte meiosis](pathway%20map/45%20map04114.png) | gnl\|UG\|Ssc#S56448572, gnl\|UG\|Ssc#S58304058, gnl\|UG\|Ssc#S55327595 |
| 46 | [Phenylalanine metabolism](pathway%20map/46%20map00360.png) | gnl\|UG\|Ssc#S56448572 |
| 47 | [Glycosphingolipid biosynthesis - lacto and neolacto series](pathway%20map/47%20map00601.png) | gnl\|UG\|Ssc#S55319682 |
| 48 | [Glycosaminoglycan biosynthesis - chondroitin sulfate](pathway%20map/48%20map00532.png) | gnl\|UG\|Ssc#S58304506 |
| 49 | [Mucin type O-Glycan biosynthesis](pathway%20map/49%20map00512.png) | gnl\|UG\|Ssc#S58311970 |
| 50 | [Osteoclast differentiation](pathway%20map/50%20map04380.png) | gnl\|UG\|Ssc#S55321680, gnl\|UG\|Ssc#S58307436, gnl\|UG\|Ssc#S35166542 |
| 51 | [Pancreatic secretion](pathway%20map/51%20map04972.png) | gnl\|UG\|Ssc#S55327620, gnl\|UG\|Ssc#S58304058, gnl\|UG\|Ssc#S58300922 |
| 52 | [Toxoplasmosis](pathway%20map/52%20map05145.png) | gnl\|UG\|Ssc#S14766117, gnl\|UG\|Ssc#S58308537, gnl\|UG\|Ssc#S43495858 |
| 53 | [MAPK signaling pathway - fly](pathway%20map/53%20map04013.png) | gnl\|UG\|Ssc#S58303491 |
| 54 | [Natural killer cell mediated cytotoxicity](pathway%20map/54%20map04650.png) | gnl\|UG\|Ssc#S55321680, gnl\|UG\|Ssc#S16592156, gnl\|UG\|Ssc#S58306512, gnl\|UG\|Ssc#S34273825 |
| 55 | [Circadian rhythm - mammal](pathway%20map/55%20map04710.png) | gnl\|UG\|Ssc#S58311511 |
| 56 | [Endocytosis](pathway%20map/56%20map04144.png) | gnl\|UG\|Ssc#S16592156, gnl\|UG\|Ssc#S35164870, gnl\|UG\|Ssc#S58313090, gnl\|UG\|Ssc#S55318348, gnl\|UG\|Ssc#S46881169 |
| 57 | [African trypanosomiasis](pathway%20map/57%20map05143.png) | gnl\|UG\|Ssc#S55321680, gnl\|UG\|Ssc#S34273825 |
| 58 | [beta-Alanine metabolism](pathway%20map/58%20map00410.png) | gnl\|UG\|Ssc#S35328054 |
| 59 | [Alanine, aspartate and glutamate metabolism](pathway%20map/59%20map00250.png) | gnl\|UG\|Ssc#S56448572 |
| 60 | [Long-term depression](pathway%20map/60%20map04730.png) | gnl\|UG\|Ssc#S58313056, gnl\|UG\|Ssc#S58304058 |
| 61 | [Parkinson's disease](pathway%20map/61%20map05012.png) | gnl\|UG\|Ssc#S40474453, gnl\|UG\|Ssc#S19548913, gnl\|UG\|Ssc#S6054441 |
| 62 | [Histidine metabolism](pathway%20map/62%20map00340.png) | gnl\|UG\|Ssc#S35328054 |
| 63 | [Bile secretion](pathway%20map/63%20map04976.png) | gnl\|UG\|Ssc#S55327620, gnl\|UG\|Ssc#S35167355 |
| 64 | [T cell receptor signaling pathway](pathway%20map/64%20map04660.png) | gnl\|UG\|Ssc#S58307436, gnl\|UG\|Ssc#S58306512, gnl\|UG\|Ssc#S35166542 |
| 65 | [Asthma](pathway%20map/65%20map05310.png) | gnl\|UG\|Ssc#S55321680, gnl\|UG\|Ssc#S14766117 |
| 66 | [RNA polymerase](pathway%20map/66%20map03020.png) | gnl\|UG\|Ssc#S58303491 |
| 67 | [Sphingolipid metabolism](pathway%20map/67%20map00600.png) | gnl\|UG\|Ssc#S58305222 |
| 68 | [Neurotrophin signaling pathway](pathway%20map/68%20map04722.png) | gnl\|UG\|Ssc#S58300959, gnl\|UG\|Ssc#S56448572, gnl\|UG\|Ssc#S34273825 |
| 69 | [Drug metabolism - other enzymes](pathway%20map/69%20map00983.png) | gnl\|UG\|Ssc#S58305630 |
| 70 | [PPAR signaling pathway](pathway%20map/70%20map03320.png) | gnl\|UG\|Ssc#S43178458, gnl\|UG\|Ssc#S55325470 |
| 71 | [Pathways in cancer](pathway%20map/71%20map05200.png) | gnl\|UG\|Ssc#S55323247, gnl\|UG\|Ssc#S58308537, gnl\|UG\|Ssc#S43495858, gnl\|UG\|Ssc#S55318348, gnl\|UG\|Ssc#S35166542, gnl\|UG\|Ssc#S34273825 |
| 72 | [Glycine, serine and threonine metabolism](pathway%20map/72%20map00260.png) | gnl\|UG\|Ssc#S35328054 |
| 73 | [Proximal tubule bicarbonate reclamation](pathway%20map/73%20map04964.png) | gnl\|UG\|Ssc#S55327620 |
| 74 | [Melanogenesis](pathway%20map/74%20map04916.png) | gnl\|UG\|Ssc#S56448572, gnl\|UG\|Ssc#S25665854 |
| 75 | [Small cell lung cancer](pathway%20map/75%20map05222.png) | gnl\|UG\|Ssc#S58308537, gnl\|UG\|Ssc#S43495858 |
| 76 | [Propanoate metabolism](pathway%20map/76%20map00640.png) | gnl\|UG\|Ssc#S35328054 |
| 77 | [Leukocyte transendothelial migration](pathway%20map/77%20map04670.png) | gnl\|UG\|Ssc#S35171399, gnl\|UG\|Ssc#S46875662, gnl\|UG\|Ssc#S58306512 |
| 78 | [Dorso-ventral axis formation](pathway%20map/78%20map04320.png) | gnl\|UG\|Ssc#S58304300 |
| 79 | [Focal adhesion](pathway%20map/79%20map04510.png) | gnl\|UG\|Ssc#S58310151, gnl\|UG\|Ssc#S58308537, gnl\|UG\|Ssc#S43495858, gnl\|UG\|Ssc#S6091069, gnl\|UG\|Ssc#S58306512 |
| 80 | [Tryptophan metabolism](pathway%20map/80%20map00380.png) | gnl\|UG\|Ssc#S35328054 |
| 81 | [Arrhythmogenic right ventricular cardiomyopathy (ARVC)](pathway%20map/81%20map05412.png) | gnl\|UG\|Ssc#S58310151, gnl\|UG\|Ssc#S58314746 |
| 82 | [Axon guidance](pathway%20map/82%20map04360.png) | gnl\|UG\|Ssc#S58300957, gnl\|UG\|Ssc#S35171399, gnl\|UG\|Ssc#S58312543 |
| 83 | [Pyruvate metabolism](pathway%20map/83%20map00620.png) | gnl\|UG\|Ssc#S35328054 |
| 84 | [Nucleotide excision repair](pathway%20map/84%20map03420.png) | gnl\|UG\|Ssc#S56448572 |
| 85 | [Antigen processing and presentation](pathway%20map/85%20map04612.png) | gnl\|UG\|Ssc#S14766117, gnl\|UG\|Ssc#S16592156 |
| 86 | Other types of O-glycan biosynthesis (no map in kegg database) | gnl\|UG\|Ssc#S46878343 |
| 87 | [Amoebiasis](pathway%20map/87%20map05146.png) | gnl\|UG\|Ssc#S55321680, gnl\|UG\|Ssc#S58308537, gnl\|UG\|Ssc#S43495858 |
| 88 | [Phosphatidylinositol signaling system](pathway%20map/88%20map04070.png) | gnl\|UG\|Ssc#S56448572, gnl\|UG\|Ssc#S58304058 |
| 89 | [Protein digestion and absorption](pathway%20map/89%20map04974.png) | gnl\|UG\|Ssc#S55327620, gnl\|UG\|Ssc#S46880230 |
| 90 | [Amino sugar and nucleotide sugar metabolism](pathway%20map/90%20map00520.png) | gnl\|UG\|Ssc#S20347643 |
| 91 | [Chemokine signaling pathway](pathway%20map/91%20map04062.png) | gnl\|UG\|Ssc#S35171399, gnl\|UG\|Ssc#S58313090, gnl\|UG\|Ssc#S58306512 |
| 92 | [Metabolism of xenobiotics by cytochrome P450](pathway%20map/92%20map00980.png) | gnl\|UG\|Ssc#S35167355 |
| 93 | [Drug metabolism - cytochrome P450](pathway%20map/93%20map00982.png) | gnl\|UG\|Ssc#S35167394 |
| 94 | [Notch signaling pathway](pathway%20map/94%20map04330.png) | gnl\|UG\|Ssc#S51750135 |
| 95 | [Oxidative phosphorylation](pathway%20map/95%20map00190.png) | gnl\|UG\|Ssc#S40474453, gnl\|UG\|Ssc#S19548913 |
| 96 | [Hematopoietic cell lineage](pathway%20map/96%20map04640.png) | gnl\|UG\|Ssc#S55321680, gnl\|UG\|Ssc#S14766117 |
| 97 | [Fat digestion and absorption](pathway%20map/97%20map04975.png) | gnl\|UG\|Ssc#S56448572 |
| 98 | [Glycerolipid metabolism](pathway%20map/98%20map00561.png) | gnl\|UG\|Ssc#S35328054 |
| 99 | [Lysine degradation](pathway%20map/99%20map00310.png) | gnl\|UG\|Ssc#S35328054 |
| 100 | [Valine, leucine and isoleucine degradation](pathway%20map/100%20map00280.png) | gnl\|UG\|Ssc#S35328054 |
| 101 | [Glycolysis / Gluconeogenesis](pathway%20map/101%20map00010.png) | gnl\|UG\|Ssc#S35328054 |
| 102 | Metabolic pathways (no map in kegg database) | gnl\|UG\|Ssc#S56448572, gnl\|UG\|Ssc#S46879456, gnl\|UG\|Ssc#S55327261, gnl\|UG\|Ssc#S40474453, gnl\|UG\|Ssc#S58303491, gnl\|UG\|Ssc#S19548913, gnl\|UG\|Ssc#S55318348, gnl\|UG\|Ssc#S55319682, gnl\|UG\|Ssc#S25665854, gnl\|UG\|Ssc#S35328054, gnl\|UG\|Ssc#S58305222, gnl\|UG\|Ssc#S58305630, gnl\|UG\|Ssc#S23212902, gnl\|UG\|Ssc#S58304506, gnl\|UG\|Ssc#S58311970 |
| 103 | [Fc epsilon RI signaling pathway](pathway%20map/103%20map04664.png) | gnl\|UG\|Ssc#S55321680, gnl\|UG\|Ssc#S58306512 |
| 104 | [Vascular smooth muscle contraction](pathway%20map/104%20map04270.png) | gnl\|UG\|Ssc#S56448572, gnl\|UG\|Ssc#S58304058, gnl\|UG\|Ssc#S35164008 |
| 105 | [Cardiac muscle contraction](pathway%20map/105%20map04260.png) | gnl\|UG\|Ssc#S55327620, gnl\|UG\|Ssc#S58314746 |
| 106 | [mTOR signaling pathway](pathway%20map/106%20map04150.png) | gnl\|UG\|Ssc#S58307095 |
| 107 | [Huntington's disease](pathway%20map/107%20map05016.png) | gnl\|UG\|Ssc#S58304058, gnl\|UG\|Ssc#S58303491, gnl\|UG\|Ssc#S58302838 |
| 108 | [Epithelial cell signaling in Helicobacter pylori infection](pathway%20map/108%20map05120.png) | gnl\|UG\|Ssc#S46875662 |
| 109 | [Carbohydrate digestion and absorption](pathway%20map/109%20map04973.png) | gnl\|UG\|Ssc#S55327620 |
| 110 | [Viral myocarditis](pathway%20map/110%20map05416.png) | gnl\|UG\|Ssc#S55321680, gnl\|UG\|Ssc#S14766117, gnl\|UG\|Ssc#S16592156 |
| 111 | [Acute myeloid leukemia](pathway%20map/111%20map05221.png) | gnl\|UG\|Ssc#S55323247 |
| 112 | [Colorectal cancer](pathway%20map/112%20map05210.png) | gnl\|UG\|Ssc#S35166542 |
| 113 | [Inositol phosphate metabolism](pathway%20map/113%20map00562.png) | gnl\|UG\|Ssc#S55327261 |
| 114 | Pancreatic cancer (no map in kegg database) | gnl\|UG\|Ssc#S55318348 |
| 115 | [Glycerophospholipid metabolism](pathway%20map/115%20map00564.png) | gnl\|UG\|Ssc#S55318348 |
| 116 | [Insulin signaling pathway](pathway%20map/116%20map04910.png) | gnl\|UG\|Ssc#S56448572, gnl\|UG\|Ssc#S58313090 |
| 117 | [Dilated cardiomyopathy](pathway%20map/117%20map05414.png) | gnl\|UG\|Ssc#S55321680, gnl\|UG\|Ssc#S58310151, gnl\|UG\|Ssc#S58314746 |
| 118 | [Purine metabolism](pathway%20map/118%20map00230.png) | gnl\|UG\|Ssc#S58303491, gnl\|UG\|Ssc#S58305630 |
| 119 | [Adipocytokine signaling pathway](pathway%20map/119%20map04920.png) | gnl\|UG\|Ssc#S55325470 |
| 120 | [Apoptosis](pathway%20map/120%20map04210.png) | gnl\|UG\|Ssc#S34273825 |
| 121 | [p53 signaling pathway](pathway%20map/121%20map04115.png) | gnl\|UG\|Ssc#S16849920 |
| 122 | [Progesterone-mediated oocyte maturation](pathway%20map/122%20map04914.png) | gnl\|UG\|Ssc#S55327595 |
| 123 | [Glioma](pathway%20map/126%20map05010.png) | gnl\|UG\|Ssc#S56448572 |
| 124 | [Pyrimidine metabolism](pathway%20map/124%20map00240.png) | gnl\|UG\|Ssc#S58303491 |
| 125 | [Olfactory transduction](pathway%20map/125%20map04740.png) | gnl\|UG\|Ssc#S56448572 |
| 126 | [Alzheimer's disease](pathway%20map/126%20map05010.png) | gnl\|UG\|Ssc#S56448572, gnl\|UG\|Ssc#S58304058 |
| 127 | [VEGF signaling pathway](pathway%20map/127%20map04370.png) | gnl\|UG\|Ssc#S58305222 |
| 128 | [Gap junction](pathway%20map/128%20map04540.png) | gnl\|UG\|Ssc#S58304058 |
| 129 | TGF-beta signaling pathway (no map in kegg database) | gnl\|UG\|Ssc#S50208755 |
| 130 | [Toll-like receptor signaling pathway](pathway%20map/130%20map04620.png) | gnl\|UG\|Ssc#S35166542 |
| 131 | [Hypertrophic cardiomyopathy (HCM)](pathway%20map/131%20map05410.png) | gnl\|UG\|Ssc#S58310151, gnl\|UG\|Ssc#S58314746 |
| 132 | [Bacterial invasion of epithelial cells](pathway%20map/132%20map05100.png) | gnl\|UG\|Ssc#S46881169 |
| 133 | [Tight junction](pathway%20map/133%20map04530.png) | gnl\|UG\|Ssc#S46875662, gnl\|UG\|Ssc#S58313090 |
| 134 | [Spliceosome](pathway%20map/134%20map03040.png) | gnl\|UG\|Ssc#S46876994 |
| 135 | [Wnt signaling pathway](pathway%20map/135%20map04310.png) | gnl\|UG\|Ssc#S43495801 |
| 136 | [RNA transport](pathway%20map/136%20map03013.png) | gnl\|UG\|Ssc#S58305376 |
| 137 | [Regulation of actin cytoskeleton](pathway%20map/137%20map04810.png) | gnl\|UG\|Ssc#S58310151, gnl\|UG\|Ssc#S58306512 |
| 138 | [RNA degradation](pathway%20map/138%20map03018.png) | gnl\|UG\|Ssc#S42528236 |
